# Synthesis of monodisperse inorganic polyphosphate polyP_10_*via* a photocaging strategy†

**DOI:** 10.1039/d5sc04037j

**Published:** 2025-07-10

**Authors:** Sandra Moser, Gloria Hans, Jiahui Ma, Thomas Haas, Nikolaus Jork, Felix Bauer, Bernhard Breit, Henning J. Jessen

**Affiliations:** a Institute of Organic Chemistry, Albert-Ludwigs-Universität Freiburg Albertstraße 21 79104 Freiburg im Breisgau Germany henning.jessen@oc.uni-freiburg.de; b CIBSS – Centre for Integrative Biological Signalling Studies, Albert-Ludwigs-Universität Freiburg Schänzlestraße 18 79104 Freiburg im Breisgau Germany

## Abstract

Inorganic polyphosphate (polyP), a linear biopolymer composed only of orthophosphate units, has emerged as a molecule of critical biological importance across species. While commercially available polyPs are polydisperse mixtures – irrespective of their origin (chemical, biochemical) – recent strategies have focused on the bottom-up synthesis of monodisperse polyPs that have distinct advantages in mechanistic studies. However, until now, syntheses have been limited to defined chains of up to eight phosphate units due to challenges in deprotection-associated degradation and purification. Here, we disclose a new strategy based on two terminal coumarin photocages to synthesize the longest monodisperse polyP chain available to date: polyP_10_. The photoremovable protecting groups facilitate purification and enable efficient deprotection with light. By tuning the photocage, we achieve control over uncaging wavelengths, integrate targeting modifications and incorporate ^18^O-labels. This is the first example of a photouncaging strategy in which an ^18^O-labeled photocage is specifically designed to release an ^18^O-labeled metabolite for downstream applications. During the uncaging, we observe an unprecedented aromatic substitution reaction from a cleaved coumarin photocage cation onto the second photocage that is still attached to the polyP chain. This suggests a π-stacking facilitated loop-like arrangement of caged polyP in water that is supported by DFT calculations.

## Abstract 2

This work presents a strategy to synthesize monodisperse polyP_10_ using photocages that enable purification, light-triggered deprotection and ^18^O labeling. The photorelease uncovers a quasi-intramolecular aromatic substitution enabled by loop formation.

## Graphical abstract

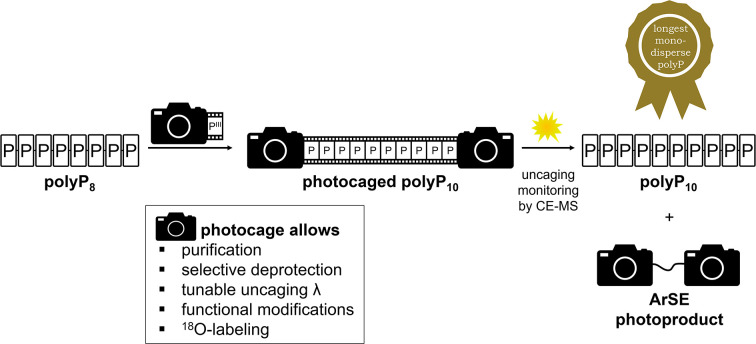

## Introduction

Inorganic polyphosphate (polyP), a linear polymer composed of three up to thousands of orthophosphates, has evolved from its former status as a “forgotten polymer” 30 years ago^1^ to a molecule of critical biological and technological importance. Today it is known that this conserved biopolymer is involved in fundamental cellular processes^2^ such as energy metabolism,^3^ stress response^4^ and DNA damage repair.^5^ Additionally, it holds biomedical relevance^6^ as it plays a role in blood clotting,^7^ inflammation,^8^ bone regeneration^9^ and bacterial virulence.^10^ PolyP has been shown to covalently and non-covalently bind to certain protein domains.^11^ Commercially available polyP is manufactured through two main methods: chemical synthesis and enzymatic synthesis.

In the chemical process, sodium monophosphate is heated to 700–1000 °C with subsequent rapid cooling.^12^ This technique yields a glass-like mixture of polyphosphates with different chain lengths,^13^ known as Graham's salt or – misguiding because of its linear structure – as sodium hexametaphosphate.^14^ By varying the temperature and vapor pressure, the average chain length can be adjusted.^12^ Moreover, different modifications have been obtained in the solid state, usually showing a helical arrangement of the polymer; based on these reports, such arrangements have also been suggested in solution.^15^

PolyP can also be synthesized enzymatically. Certain organisms, including yeast, bacteria and algae store high quantities of polyphosphates, which can be isolated through methods like phenol/chloroform extraction.^12^ Recently, an optimized extraction protocol has also become available for mammalian cells.^16^ While not yet commercially available, innovative biotechnological methods have demonstrated the potential to use *Saccharomyces cerevisiae* to convert phosphate-rich wastewater^17^ or de-oiled seeds and bran^18^ into sodium polyphosphate. These production methods have all in common, that they provide polyP samples with mixed chain lengths, making it hard to apply precise analytical techniques like mass spectrometry.

**Scheme 1:**
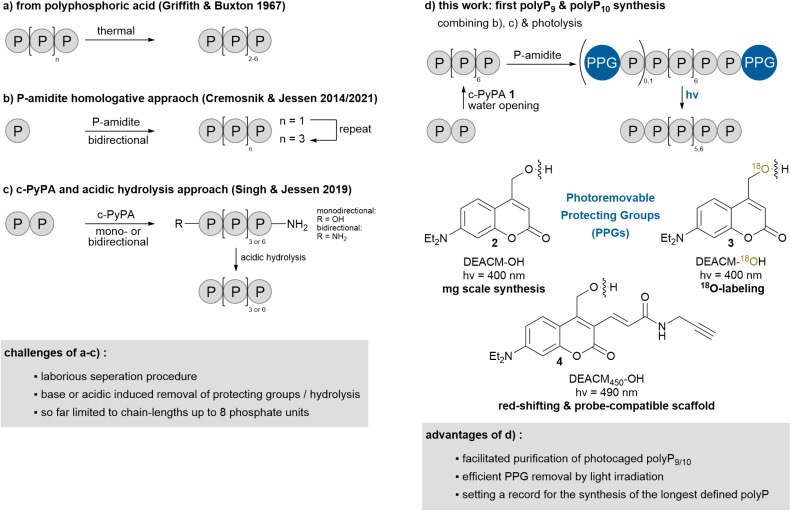
Overview of chemical synthesis strategies for defined unmodified short-chain polyPs.

**Scheme 2:**
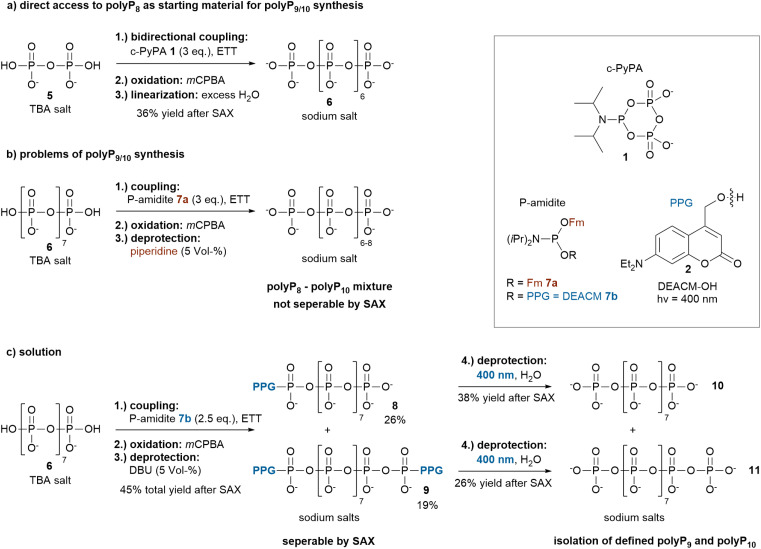
Syntheses of polyP_8_6, polyP_9_10 and polyP_10_11. (a) One-step synthesis of polyP_8_6*via* the bidirectional c-PyPA approach with water-induced ring opening. (b) Attempted synthesis of polyP_9_ and polyP_10_ using the bidirectional P-amidite method with a standard P-amidite 7a followed by piperidine deprotection,^20*b*^ yielding a non-separable polyP_8_, polyP_9_ and polyP_10_ mixture using SAX. (c) Successful synthesis of polyP_9_10 and polyP_10_11*via* photolysis of their photocaged derivatives 8 and 9, which are separable by SAX, employing a photocaged P-amidite 7b. Abbreviations: TBA: tetrabutylammonium, ETT: 5-(ethylthio)-1*H*-tetrazole, *m*CPBA: *meta*-chloroperbenzoic acid, Fm: fluorenylmethyl, PPG: photoremovable protecting group, DBU: 1,8-diazabicyclo[5.4.0]undec-7-ene.

The isolation of polyPs with a defined chain-length is currently not possible with the methods described above, but recent advances have enabled the synthesis of short-chain polyPs up to eight phosphate units on mg to g scale (Scheme 1). Pure tetra- to octapolyphosphates are accessible by heating polyphosphoric acid, resulting in a polyP mixture with an average chain length of around five (Scheme 1a).^19^ Separation is achieved through multiple extraction steps combined with cation- and anion-exchange chromatography making this process highly labor-intensive.^19^ The bottom-up synthesis of defined polyPs is possible using a P-amidite homologative approach (Scheme 1b).^20^ This process builds on three steps: activation/coupling, oxidation and base-induced deprotection, which can be performed in a single flask, and can be repeated iteratively.^20*a*,21^ To enhance efficiency, an improved approach was developed using the triphosphorylation reagent cyclic pyrophosphoryl P-amidite 1 (c-PyPA, Scheme 1c and 2).^20*b*,22^

This reagent enables the simultaneous incorporation of three phosphate units in a monodirectional approach or six in a bidirectional approach. The three-steps – activation/coupling, oxidation and linearization by nucleophiles – can be carried out in one pot as well. Amines are particularly effective for linearization. Starting from pyrophosphate, one then has direct access to symmetrical polyP_8_-diamidates, which can be hydrolyzed in acidic conditions to yield unmodified monodisperse polyP_8_.^22*a*,23^ While additional polyphosphorylation reagents exist for the synthesis of terminally modified oligophosphates,^24^ including nucleoside tetra- to heptaphosphates,^25^ dinucleoside tetra- and penta-phosphates^24*c*,25*a*,26^ as well as oligophosphorylated peptides,^27^ none of them have yet been used to synthesize unmodified polyP. To date, no monodisperse polyPs with chain lengths longer than eight units have been successfully synthesized. Achieving this would be an important step towards covering additional biological polyP structures, thus enabling more precise analysis^28^ and enhancing our understanding of their metabolic functions and topology. Defined longer chains will serve for precise analytical assignments and can help to understand and quantify polyP binding to proteins.

The synthetic limitation arises from the complexity of acquiring suitably long phosphate precursors and the increasing difficulty in purifying and isolating well-defined, elongated, non-UV active polyphosphates.^29^

Herein, we address these challenges by developing a novel strategy, which extends one-step-synthesized polyP_8_ to monodisperse and unmodified polyP_9_ and polyP_10_*via* photocaged polyP_9_ and polyP_10_ (Scheme 1d). The photoremovable protecting groups are crucial for their separation and enable efficient deprotection by light irradiation, avoiding decomposition observed with chemically triggered deprotection. Additionally, by tuning the photoremovable protecting groups, our approach allows for adjustment of the uncaging wavelength, the addition of clickable residues for probe development and the incorporation of ^18^O-labels to create heavy derivatives of polyP_9_ and polyP_10_ underlining the versatility of the approach. During the photouncaging experiments, we observed an unprecedented aromatic substitution on one photocage by the primary coumarin cation of another, indicating the formation of a loop-like structure in the polyP chain, likely stabilized by π-stacking interactions between the coumarin cages, aligning them in close proximity.

## Results and discussion

### Chemical synthesis of polyP_9_ and polyP_10_

PolyP_8_6 was synthesized in a single step starting from pyrophosphate 5, using the bidirectional approach with c-PyPA 1 (Scheme 2a). Direct water-mediated ring opening to polyP_8_6 has been low-yielding previously, while amine-induced linearization followed by acidic hydrolysis was more effective.^22*a*^ The modified approach described herein provides direct access to unmodified polyP_8_ by quenching the reaction mixture into excess of water, resulting in clean linearization to polyP_8_6.

To extend this readily available polyP_8_ (235 mg synthesized in a single step) to polyP_10_, the bidirectional P-amidite approach can be used.^20*b*^ However, this strategy presents two main challenges: first, the basic conditions required to remove the fluorenylmethyl (Fm) protecting groups from the newly introduced terminal phosphates can lead to partial degradation of the polyP chain. Second, the resulting mixture of polyP_8_, polyP_9_ and polyP_10_ is difficult to separate effectively by strong anion exchange chromatography (SAX) and hard to assign analytically (Scheme 2b). To overcome these limitations, a P-amidite bearing only one Fm group and a photoremovable protecting group (photocage) was employed enabling milder and orthogonal cleavage (Scheme 2c). The coumarin derivative DEACM-OH 2 was chosen due to its well-established photocleavage mechanism and its straightforward three-step synthesis.^30^ Reaction of the P-amidite 7b (2.5 eq.) with polyP_8_6 followed by oxidation and careful Fm removal gave a mixture of mono-photocaged polyP_9_8 (26% yield) and bis-photocaged polyP_10_9 (19% yield) in a ratio of approx. 1.4 : 1. Increasing the amount of P-amidite 7b to 3 eq. shifted the product distribution strongly in favour of bis-photocaged polyP_10_9 with a product ratio of 8 to 9 of approx. 0.1 : 1. The two products were readily separated by SAX. UV-activity of these compounds greatly facilitated purification. While their proton NMR chemical shifts are very similar, ^31^P-NMR provided a clear distinction: unsymmetrically mono-caged polyP_9_8 shows an integration ratio of 1 : 1 : 7, whereas 9 shows a 2 : 8 pattern. This confirms the symmetrical dual-caged structure of the latter (ESI,† for a ^31^P-NMR chemical shift table for condensed phosphates see Accounts Chem. Res.20b). Irradiation of 8 and 9 at *λ* = 400 nm released defined polyP_9_10 or polyP_10_11, respectively, without requiring acid or base treatment. The reaction proceeded cleanly; however, precipitation followed by extensive washing failed to completely remove the DEACM-OH 2 cleavage product, resulting in the isolation of a yellow-brown precipitate. Therefore, purification *via* SAX was required, which led to significant losses and ultimately reduced the yields to 38% for 10 and 26% for 11, respectively. Importantly, this new synthesis approach conceptually allows for further extension of the polyP chain by using polyP_9_ or polyP_10_ as starting materials.

### An unexpected quasi-intramolecular reaction during the uncaging process

**Fig. 1:**
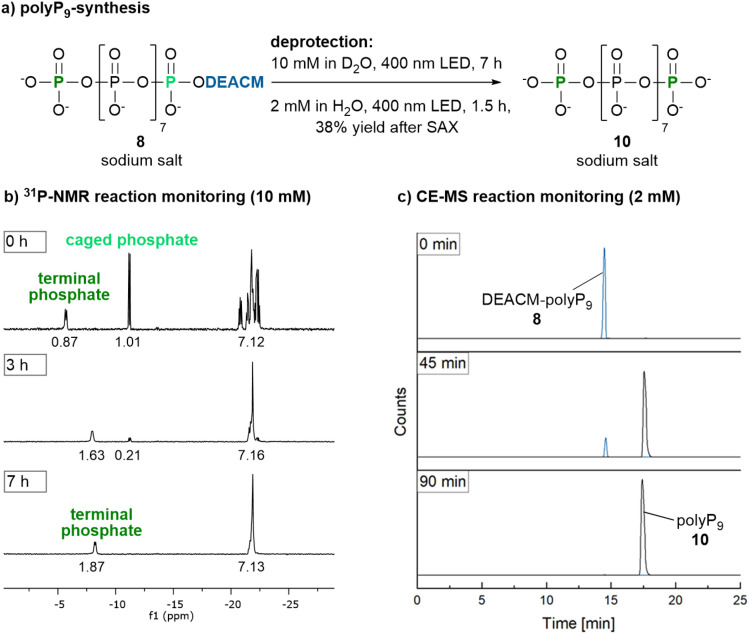
(a) Photorelease of polyP_9_10 from 8 at 400 nm. (b) At 10 mM in H_2_O, ^31^P-NMR monitoring indicated complete photorelease after approx. 7 h. (c) At a lower concentration of 2 mM in H_2_O, CE-MS monitoring (with sample dilution to 500 μM prior to analysis), showed completion of cleavage after approx. 90 min. Detailed time courses are available in Fig. ESI-2.†

It is possible to track the uncaging process (Fig. 1a) in water by ^31^P-NMR. This requires high concentrations (approx. 10 mM) to detect the phosphate resonances of the termini. As expected, during polyP_9_ release from mono-caged polyP_9_8, the ratio of the free phosphate signal to the caged phosphate signal gradually shifted from 1 : 1 to 2 : 0 over time (Fig. 1b and ESI-2a†). However, this process was slow, taking approximately 7 h to completion, as the cleaved DEACM-OH 2 chromophore competes for light absorption and is poorly water soluble, leading to precipitation and an opaque reaction mixture. A more practical approach was to conduct the reaction at lower concentration, such as 2 mM or less, and track its progress by capillary electrophoresis coupled to mass spectrometry^31^ (CE-MS, Fig. 1c and ESI-2b†). Dilution significantly shortens the reaction time to 90 min.

**Scheme 3:**
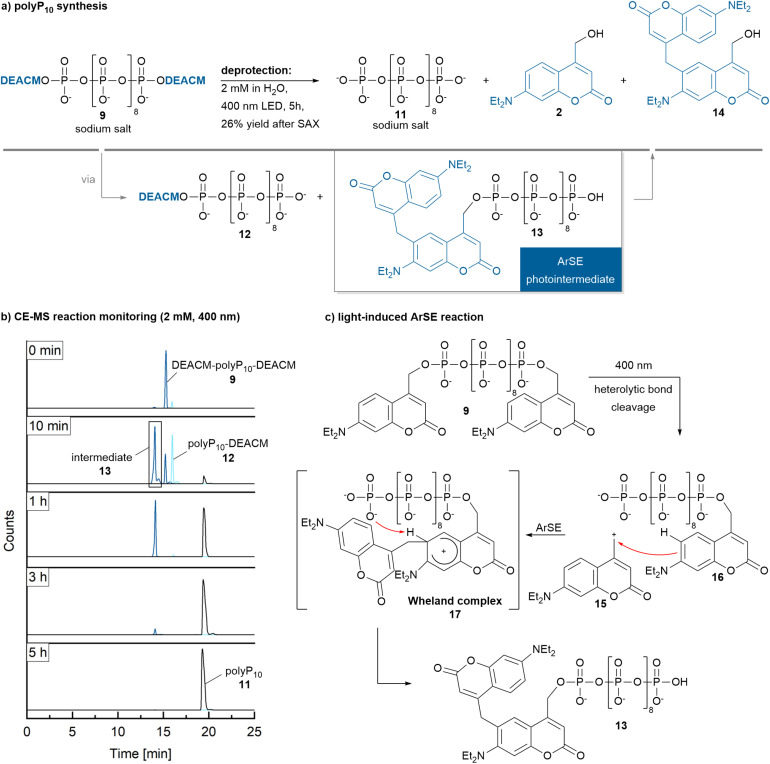
(a) Photorelease of polyP_10_11 from 9 (2 mM in H_2_O) at 400 nm. (b) CE-MS reaction monitoring (sample dilution to 500 μM prior to analysis) revealed complete photorelease within 5 h, proceeding through two intermediates, 12 and 13. (c) The formation of 13 is proposed to be a quasi-intramolecular ArSE reaction, occurring after the cleavage of one of the two photoremovable protecting groups from a loop-like pre-oriented structure (see Fig. 2). Evidence for the release of the byproducts 2 and 14 is provided in Fig. ESI-4.†

**Fig. 2:**
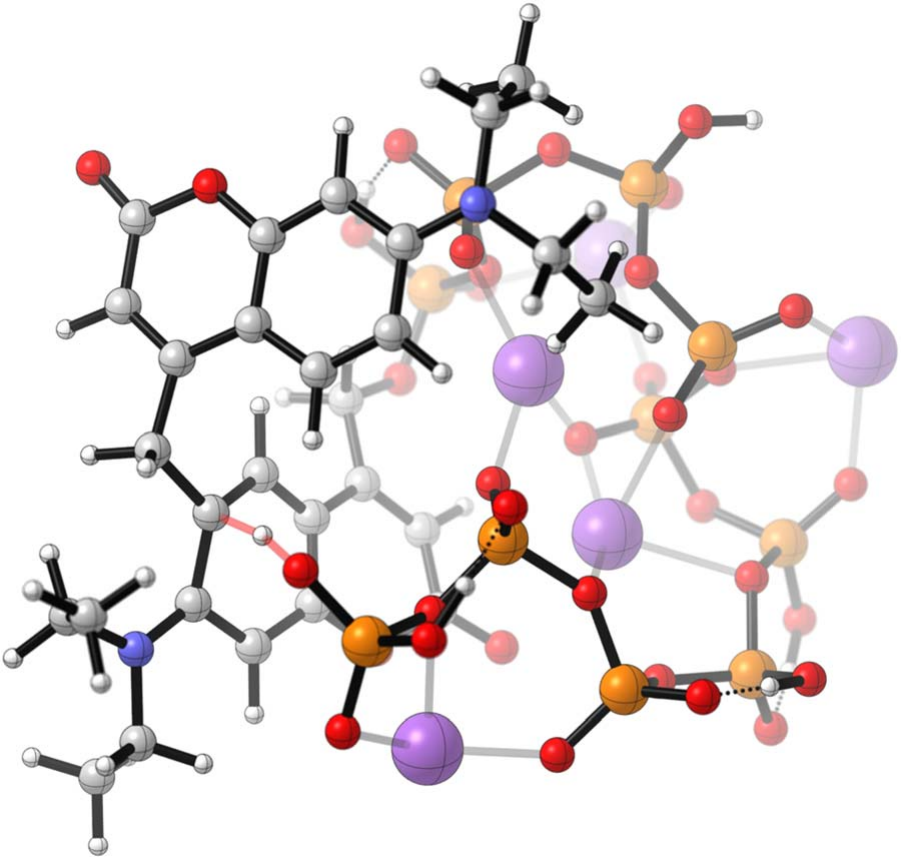
Calculated transition state of the proton abstraction (red bonds) in Wheland complex 17 supporting a loop-like structure of the polyP_10_ chain (P: orange, O: red). The counterions are Na^+^ (purple). [BP86/def2SVP-D3BJ-SMD(water)].

A similar behaviour as discussed above was expected for the bis-photocaged polyP_10_9 (Scheme 3a). The reaction should proceed through the mono-photocaged polyP_10_ intermediate 12. However, interestingly, a new main distinct peak appeared after a very short irradiation time in the CE-MS profile in addition to the peaks for the starting material 9, mono-caged polyP_10_12 and the free polyP_10_11 both at 2 mM of 9 (Scheme 3b and Fig. ESI-3a†), as well as at 100 μM of 9 (Fig. ESI-3b†). This new peak represents a constitutional isomer with the exact same mass as the starting material 9. The new isomer could still be further cleaved under irradiation to yield polyP_10_11 albeit at a reduced rate. The reaction (2 mM) was complete after approximately 5 h of irradiation. Literature reports that coumarins can undergo reversible [2 + 2] cycloadditions^30*c*,32^ or decarboxylation^30*c*,33^ under UV light, the latter ruled out by the requirement of identical mass. Thus, the [2 + 2] cycloaddition would be a viable explanation. However, the expected cycloadduct would no longer function as a photocage, which contradicts our observations. Yet, regeneration of the cage and cleavage might be a result of a [2 + 2] cycloreversion. To identify the intermediate, it was generated by stopping the reaction after 1 h irradiation time, when it had accumulated next to polyP_10_11, followed by isolation *via* SAX. Full NMR characterization suggested the formation of the substitution product 13 (Scheme 3). The proposed mechanism, illustrated in Scheme 3c, is also supported by DFT calculations (Fig. 2, [BP86/def2SVP-D3BJ-SMD(water)]). It starts with the photolysis of bis-DEACM-caged polyP_10_9. Coumarin photocages are believed to operate through the heterolysis of the DEACM–OP bond in the excited state. This generates a contact ion pair^34^ consisting of the primary DEACM cation 15 and its conjugated base, the anion of the leaving group 16.^35^ Unlike typical pathways where the cation would either quickly recombine with the anion or be intercepted by the solvent water after escape from the contact ion pair,^35^15 and 16 instead undergo an electrophilic aromatic substitution (ArSE). The reaction proceeds through Wheland complex 17, in which a phosphate group oxygen abstracts the aromatic proton (Fig. 2). For this quasi-intramolecular reaction to occur, the two DEACM residues must be in close proximity, as otherwise the primary cation generated during photoheterolysis rapidly reacts with water.^34^ This suggests that in the starting material 9, the polyP_10_ chain adopts a loop-like conformation, stabilized by π- stacking interactions between the DEACM-modifications in the polar solvent water. Since the newly formed intermediate 13 retains the coumarin photocage structure, prolonged irradiation leads to the release of unmodified polyP_10_11, albeit more slowly, potentially as a result of more efficient relaxation pathways. Indeed, HRMS analysis of the fully deprotected polyP_10_ reaction mixture – lyophilized after light irradiation and redissolved in DCM – revealed the presence of the final cleavage product 14 alongside DEACM–OH 2 (Fig. ESI-4†). Examples in which the contact ion pair of a coumarin-caged compound undergoes reaction pathways beyond simple recombination to the starting material or solvent trapping have been reported. These include an intramolecular cyclization rearrangement in styryl-substituted coumarins^36^ and deprotonation of a tertiary coumarin cation yielding an alkene rather than the expected alcohol.^34^ Photocleavage of a trimethylsilyl-substituted coumarin-based photocage also affords an alkene by either intramolecular silylcarbonylation, hydrolysis or Peterson-type desilylation after photoexcitation.^37^ Photo-Claisen rearrange-ments that impair release efficiency have also been observed in coumarin-caged tyrosine^38^ and 4-hydroxytamoxifen analogues.^39^ ArSE reactions have not been previously described.

**Scheme 4:**
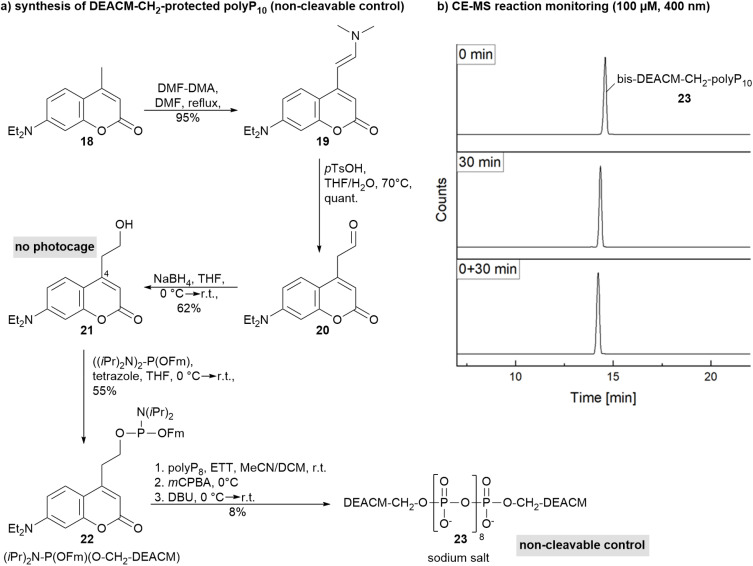
(a) Synthesis of bis-DEACM–CH_2_–protected polyP_10_23 as non-cleavable control. (b) CE-MS reaction monitoring shows no formation of an isobaric product. Abbreviations: DMF-DMA: *N*,*N*-dimethylformamide dimethyl acetal, DMF: dimethylformamide, *p*TsOH: *p*-toluenesulfonic acid, THF: tetrahydrofuran, Fm: fluorenylmethyl, ETT: 5-(ethylthio)-1*H*-tetrazole, DCM: dichloromethane, *m*CPBA: *meta*-chloroperbenzoic acid, DBU: 1,8-diazabicyclo[5.4.0]undec-7-ene.

To further demonstrate that the light-induced heterolysis of the DEACM–OP bond is essential for side-product formation in our system, bis-DEACM–CH_2_–protected polyP_10_23 was synthesized (Scheme 4a) and irradiated at *λ* = 400 nm (Scheme 4b and Fig. ESI-5†). In this derivative, the DEACM carbon chain at position 4 is extended by one CH_2_ group, eliminating its uncaging pathways, while still potentially enabling the known [2 + 2] cycloaddition of coumarins. Electropherograms showed no additional peak with the same mass (Scheme 4b and Fig. ESI-5†), confirming that ArSE product formation did not occur under these conditions and requires primary cation generation in a contact ion pair. Extended irradiation (beyond 2 h) led to decomposition, resulting in a mixture of undefined products. The synthesis of DEACM–CH_2_–OH 21 was accomplished in three steps, starting from commercially available coumarin 18*via* enamine formation, hydrolysis and reduction (Scheme 4a). The protected polyP_10_23 exhibits an absorption maximum at 381 nm and two fluorescence maxima of 437 nm and 474 nm. The presence of multiple emission maxima, not only in 23, but also in the mono-DEACM-caged polyP_9_8 and bis-DEACM-caged polyP_10_9 (Table 1), may be explained by the existence of chromophore π-stacking interactions. This supports not only a loop-like arrangement of the polyP chain in bis-caged molecules, but highlights the potential for intermolecular interactions leading to supramolecular aggregation.^40^ Bis-DEACM–CH_2_ protected polyP_10_23 is the first example of a fluorophore end-labeled monodisperse double-digit polyP and may have versatile applications, such as fluorescence-based tracking of cellular uptake and direct fluorescent detection of polyphosphorylated proteins on gels without the need for staining methods.

**Table 1:** Photophysical properties in H_2_O

polyP	Absorption *λ*_max_^a^/nm	*ε*_max_ b/(M^−1^ cm^−1^)	Emission *λ*_max_^c^/nm
DEACM-polyP_9_8	386	16 340	445, 491, 527
^18^O-DEACM-polyP_9_34	386	16 220	445, 491, 527
Bis-DEACM-polyP_10_9	382	20 280	440, 490, 527
Bis-^18^ O-DEACM-polyP_10_35	382	26 440	440, 490, 527
Bis-DEACM–CH_2_–polyP_10_23	381	21 060	437, 474
Bis-DEAC_450_-polyP_10_24	438	28 474	523, 543
Bis-TPP-DEAC_450_-polyP_10_27	441	16 975	520, 542

a Wavelength of the absorption maximum, 50 μM.

b Molar extinction coefficient at the absorption maximum *λ*_max_.

c Wavelength of the emission maxima upon excitation at the absorption maximum, 100 nM.

### Synthesis of clickable, red-shifted photocaged polyP_10_

**Fig. 3:**
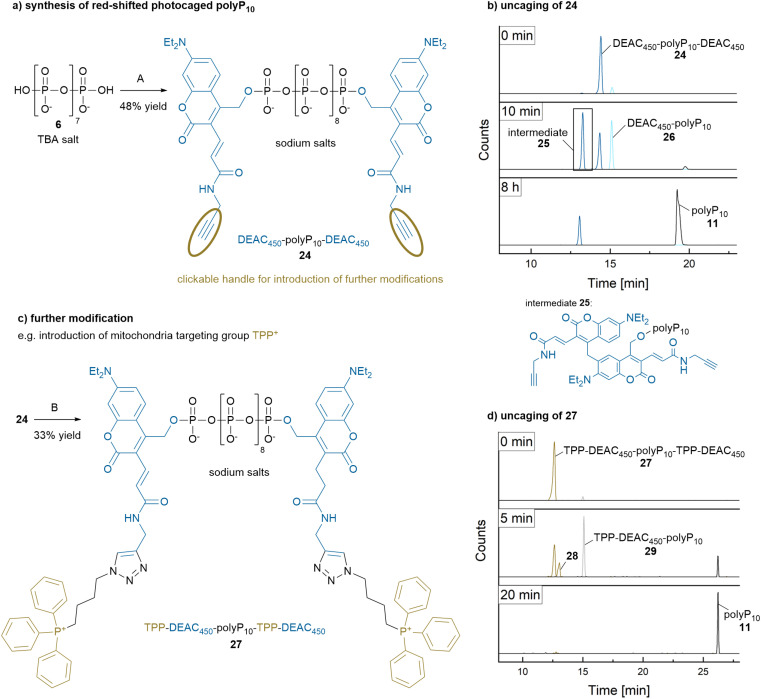
Synthesis and uncaging of red-shifted and modified caged polyP_10_'s. (a) Synthesis of bis-DEAC_450_ caged polyP_10_24. A: 1. (*i*Pr)_2_N–P(OFm)(ODEAC_450_) (3.0 eq.), ETT (20 eq.), MeCN/DCM, r.t., 30 min. 2. *m*CPBA (3.0 eq.), 0 °C, 20 min. 3. DBU (5 vol%), 0 °C → r.t., 1 h. (b) Photorelease of polyP_10_11 from 24 (100 μM in H_2_O) at 490 nm. CE-MS reaction monitoring demonstrated that the photorelease proceeds through two intermediates, 25 and 26. The formation of 25 follows the mechanism suggested in Scheme 3. The photorelease process was slowed down through 25 and incomplete even after 8 h of irradiation. (c) Synthesis of bis-TPP-DEAC_450_ caged polyP_10_27. B: (4-azidobutyl) triphenyl phosphonium bromide (2.0 eq.), CuSO_4_·5H_2_O (1.0 eq.), THPTA (5.0 eq.), sodium ascorbate (10 eq.), 100 mM TEAA/DMSO, r.t., 3 h. (d) Photorelease of polyP_10_11 from 27 (100 μM in H_2_O) at 490 nm. CE-MS reaction monitoring revealed complete photorelease within 20 min *via* mono-caged polyP_10_29. The isobaric intermediate 28, formed from 27, was not isolated and identified. Abbreviations: Fm: fluorenylmethyl, ETT: 5-(ethylthio)-1*H*-tetrazole, DCM: dichloromethane, DMSO: dimethyl sulfoxide, *m*CPBA: *meta*-chloroperbenzoic acid, DBU: 1,8-diazabicyclo[5.4.0]undec-7-ene, TPP: triphenyl-phosphonium, THPTA: tris[(1-hydroxy-propyl-1*H*-1,2,3-triazol-4-yl)methyl]amine, TEAA: triethylammonium acetate.

Bis-DEACM-photocaged polyP_10_9 has an absorption maximum at 382 nm (Table 1) and can be cleaved with a 400 nm LED. However, for specific applications, such as cellular studies, red-shifted activation is preferred to high-energy UV light. Visible light has better tissue penetration and has reduced photo-toxicity to cells.^41^ By using different photocages, our synthetic approach allows for easy tuning of these parameters. Moreover, targeting moieties and modifications that enhance cellular uptake could be installed.^42^ Intracellular delivery of polyP into cells has previously been achieved using polycationic molecular transporters by noncovalent polyplex formation.^43^ Choosing clickable^42^ DEAC_450_–OH^44^4 instead of DEACM-OH 2 as photocage on the P-amidite, a photocaged polyP_10_24 with an absorption maximum around 438 nm was obtained (Fig. 3a and Table 1). Additionally, it features a clickable handle for further probe development, such as organelle-specific targeting.^45^ In-terestingly, during the uncaging process with 490 nm light, the direct photorelease again competed with an ArSE reaction as identified by CE-MS, LC-MS and NMR (Fig. 3b and ESI-7†).

It likely follows the mechanism and required loop pre-arrangement proposed in Scheme 3c. While the bis-DEACM-polyP_10_ ArSE intermediate 13 fully released free polyP_10_11 (see Fig. ESI-3b†) within 60 min at a concentration of 100 μM, the formation of the ArSE intermediate of bis-DEAC_450_-polyP_10_25 significantly slowed down the polyP_10_11 release, preventing complete polyP_10_ liberation even after 8 h irradiation at 100 μM (Fig. 3b and ESI-6†). This may in part be attributed to the lower output power of the 490 nm LED (140 (mW)^3^) compared to the 400 nm LED (265 (mW)^3^), yet we surmise that additional rotational degrees of freedom for excited state inactivation and potentially energy transfer between the two chromophores followed by dissipation are also playing a role here. Notwithstanding, the reaction proceeded cleanly (see Fig. 3b), but at a much slower rate.

As an example for an organelle-targeting modification, we clicked^46^ the mitochondria-targeting group triphenyl-phosphonium (TPP^+^) to the bis-DEAC_450_-polyP_10_24 to obtain 27 (Fig. 3c). PolyP has been proposed to be produced by the mitochondrial F_0_F_1_-ATP synthase in mammalian cells,^47^ and consequently its subcellular targeting for biological studies would be beneficial. However, the slow-release kinetics described above would be a major obstacle for further tool development. Even so, steric hindrance in the TPP^+^ modified caged polyP in combination with coulombic repulsion of positive charges might reduce or obliterate the ArSE side reaction. Indeed, a 100 μM solution of 27 exposed to 490 nm light fully released unmodified polyP_10_11 within only 20 min (Fig. 3d and ESI-8†). The isobaric intermediate 28, formed from 27, did not significantly affect the photorelease kinetics and was not further characterized. These results indicate that 27 is, in principle, suitable for uncaging in living cells.

### Synthesis of ^18^O-labeled polyP_9_ and polyP_10_

**Scheme 5:**
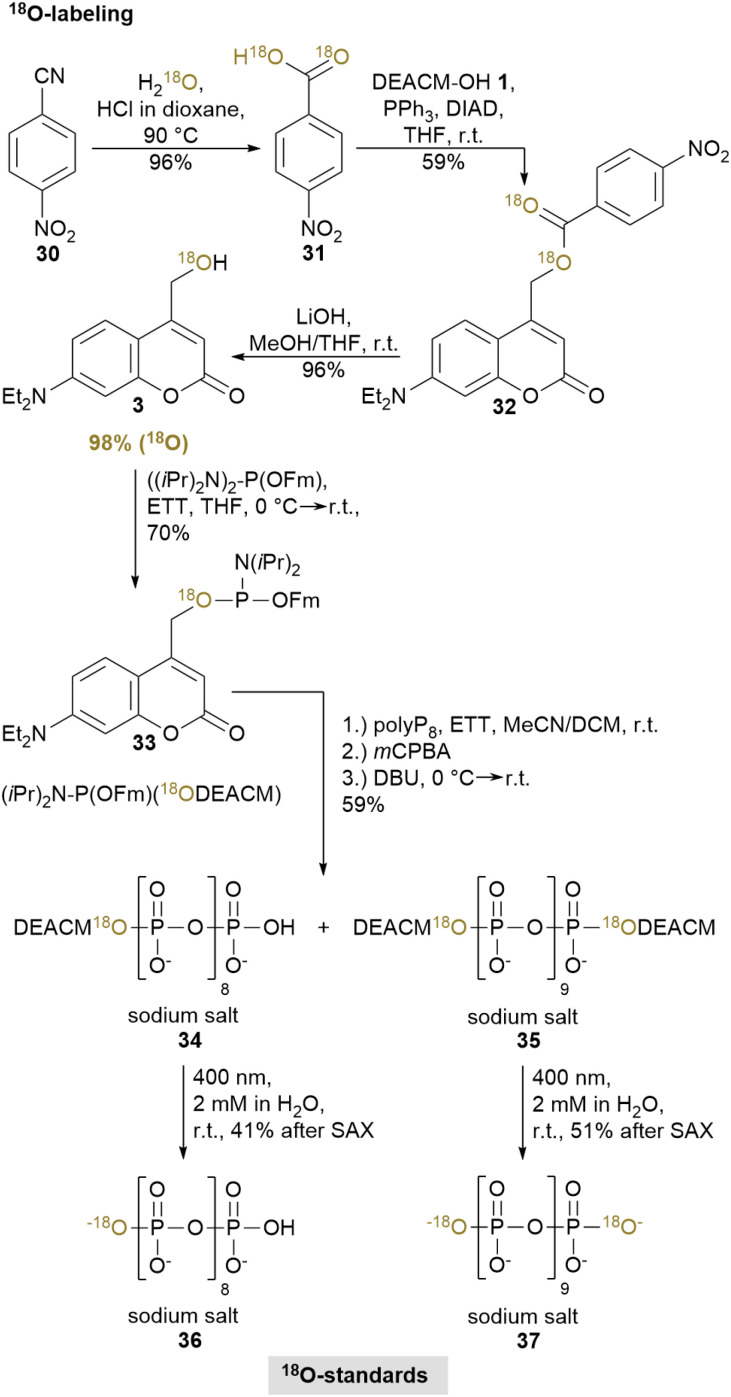
Synthesis of ^18^O-labeled polyP_9_36 and ^18^O-labeled polyP_10_37. Abbreviations: DIAD: diisopropyl azodicarboxylate, THF: tetrahydrofuran, Fm: fluorenylmethyl, ETT: 5-(ethylthio)-1*H*-tetrazole, DCM: dichloromethane, *m*CPBA: *meta*-chloroperbenzoic acid, DBU: 1,8-diazabicyclo[5.4.0]undec-7-ene.

We have recently demonstrated the use of ^18^O-labeled phosphorylated metabolites as internal standards for quantitative CE-MS analysis.^48^ Among available isotope labeling strategies, ^18^O-labeling represents the only suitable approach for polyP, as oxygen is the only element with stable isotopes present in polyP. While Haas *et al.* achieved ^18^O-labeling of polyP_4_ using a base-labile P-amidite,^48^ our synthesis method now enables the straightforward incorporation of ^18^O-labels into the terminal phosphates of polyP_9/10_ (and by extension beyond) by using ^18^O-labeled DEACM-OH 3 as protecting group (Scheme 5). This heavy photocage was synthesized *via* Mitsunobo esterification of DEACM-OH 2 with ^18^O-labeled 4-nitrobenzoic acid 31, followed by hydrolysis based on a ^18^O- labeling strategy for alcohols from Beddoe *et al.*^49^ The corresponding photocaged P-amidite 33 was synthesized according to standard procedures.^50^ Coupling 2.5 eq. of P-amidite 33 to polyP_8_6 yielded a mixture of mono-^18^ O-DEACM-polyP_9_34 (20% yield) and bis-^18^ O-DEACM-polyP_10_35 (39% yield) which were well separable by SAX. Deprotection of 34 and 35 with 400 nm at 2 mM for 2 or 8 h, respectively, yielded ^18^O-labeled polyP_9_36 with 97% ^18^O-isotope enrichment (5.8% natural abundance) or ^18^O-labeled polyP_10_37 with 95 : 5 (^18^O_2_ : ^18^O) isotope ratio (6.4% natural abundance). Importantly, these are the first examples of an ^18^O-labeled photocage designed specifically to release ^18^O-labeled metabolites for downstream use, unlike previous strategies, where the label is incorporated during uncaging *via*^18^O-enriched water,^35,51^ pre-installed in the biomolecule^52^ or where the focus lies solely on the uncaging mechanism itself.^53^ This ^18^O-labeling approach can be adapted for the synthesis of diverse ^18^O-labeled phosphorylated metabolites for use in biology, medicine and environmental science.

## Conclusions

This study discloses the synthesis of two monodisperse polyphosphates, polyP_9_ and polyP_10_. It follows a new strategy to access for the first time polyP in the two-digit range. By generating photocaged versions, an effective separation of the different chain lengths in solution becomes possible. The uncaging process selectively releases the pure polyPs with defined chain length. It can be tracked conveniently with CE-MS in an aqueous environment.

During our study, we identified a novel photolysis side reaction involving quasi-intramolecular electrophilic aromatic substitution in bis-DEACM and bis-DEAC_450_ caged polyP_10_, slowing down photorelease. This discovery suggests a loop-like structure of the caged polyP_10_ starting materials, possibly stabilized through π-stacking in water. The unique deactivation mechanism *via* ArSE for coumarin type photocages from the contact ion pair state^34^ has not been described previously and is supported by DFT calculations. Importantly, the side-reaction can be reduced by introducing larger substituents on the photocage, such as TPP^+^.

Additionally, varying the photocage enables tailoring the uncaging wavelength and incorporating handles for further functional modifications. Both are paving the way for organelle-specific delivery of polyP_10_ that can be released by light irradiation within cells, a focus for our future studies. Furthermore, utilizing an ^18^O-labeled photocage allows for the introduction of ^18^O into polyP_9_ and polyP_10_, which can be applied as internal references in mass spectrometry. This strategy is readily extendable to the synthesis of other ^18^O-labeled phosphorylated metabolites to allow their identification and quantification in complex biological samples *via* quantitative CE-MS. Their light-controlled release in living cells offers a tool to study intracellular dynamic phosphate turnovers and perturb cellular polyP metabolism.^54^

## Author contributions

S. Moser and H. Jessen designed the molecules. S. Moser and G. Hans synthesized most of the compounds. In addition, J. Ma, T. Haas, and N. Jork provided precursors. S. Moser characterized the compounds. F. Bauer performed DFT calculations. S. Moser drafted the initial manuscript and prepared the schemes and figures. B. Breit and H. Jessen reviewed the manuscript draft. H. Jessen conceived the project and provided feedback.

## Conflicts of interest

There are no conflicts to declare.

## Supplementary Material

SC-016-D5SC04037J-s001

## Data Availability

The data supporting this article have been included as part of the ESI.†
