# Supplementary material for: Synthesis of monodisperse inorganic polyphosphate polyP10via a photocaging strategy
Source: Chem Sci. 2025 Jul 10;16(32):14635–45. doi: 10.1039/d5sc04037j (PMC12257170; doi:10.1039/d5sc04037j)
Supplement: SC-016-D5SC04037J-s001 [file SC-016-D5SC04037J-s001.pdf]

Supplementary Information (SI) for Chemical Science.
This journal is © The Royal Society of Chemistry 2025

Supporting Information – Experimental details

Synthesis of monodisperse inorganic polyphosphate polyP$_{10}$ via a photocaging strategy

*Sandra Moser, Gloria Hans, Jiahui Ma, Thomas Haas, Nikolaus Jork, Felix Bauer, Bernhard Breit and Henning J. Jessen**

Table of Content:

1. Synthesis 3
 1.1 General Methods and Materials 3
 1.2 Synthesis of polyP$_8$ via water opening 5
 1.3 Synthesis of polyP$_9$ and polyP$_{10}$ via photocaged polyP$_{9/10}$ 6
 1.4 Synthesis of ^{18}O-labeled polyP$_9$ and polyP$_{10}$ 11
 1.5 Synthesis of DEACM-CH$_2$ protected polyP$_{10}$ 16
 1.6 Synthesis of DEAC$_{450}$ caged polyP$_{10}$'s 19
2. Absorption and Fluorescence Spectra 24
3. Photolysis 25
4. DFT-Studies 29
5. Supporting Literature 35
5. NMR Spectra 36
6. HRMS (ESI) Data 96

Abbreviations:

**aq.**	aqueous
**CE**	capillary electrophoresis
**DBU**	1,8-diazabicyclo[5.4.0]undec-7-ene
**DCM**	dichloromethane
**DEACM**	[7-(diethylamino)coumarin-4-yl]methyl
**DIAD**	diisopropyl azodicarboxylate
**DMSO**	dimethyl sulfoxide
**eq.**	equivalent
**ESI**	electrospray ionization
**EtOAc**	ethyl acetate
**ETT**	5-(ethylthio)-1*H*-tetrazole
**Fm**	fluorenylmethyl
**HRMS**	high resolution mass spectrometry
***m*CPBA**	*meta*-chloroperbenzoic acid
**MS**	mass spectrometry
**NMR**	nuclear magnetic resonance
**NP-MPLC**	normal-phase medium pressure liquid chromatography
**polyP**	Inorganic polyphosphate
**PP_i**	pyrophosphate
**qTOF**	quadrupole time-of-flight
**RP-MPLC**	reverse-phase medium pressure liquid chromatography
**r.t.**	room temperature
**TBA**	tetrabutylammonium
**TEA**	triethylammonium
**TEAA**	triethylammonium acetate
**THF**	tetrahydrofuran
**THPTA**	tris[(1-hydroxy-propyl-1*H*-1,2,3-triazol-4-yl)methyl]amine
**TLC**	thin layer chromatography
**UV**	ultraviolet

1. Synthesis

1.1 General Methods and Materials

Reactions were carried out in flame-dried glassware under argon atmosphere when dry solvents were used. Reagents were purchased from commercial suppliers and were used without further purification. Solvents were obtained in analytical grade and were used as received. Dry solvents were purified using Braun Solvent Purification System 800 (Et_2O, THF) or purchased from Thermo Scientific Chemicals (DCM (99,8%, Extra Dry over Molecular Sieve, Stabilized, AcroSeal®), MeCN (99,9%, Extra Dry over Molecular Sieve, AcroSeal®)). Water was purified with a Milli-Q® lab water system. Reaction control was done by thin layer chromatography (TLC) on Merck silica gel 60 F_{254} aluminium sheets with visualization achieved by UV-light (λ = 245 nm) or $^{31}P\{^1H\}$-NMR.

Flash chromatography was carried out using silica gel 60 (0.04 – 0.063 nm) from *Macherery-Nagel*.

Normal-Phase-Medium Pressure Liquid Chromatography (NP-MPLC) was performed with the Flash Chromatography System PuriFlash® 430 from Interchim®. PuriFlash® Silica HP Flash Columns were purchased from Interchim®.

Preparative Reverse-Phase-MPLC (RP-MPLC) was performed with the Flash Chromatography System PuriFlash® 5.125 from Interchim® using an Interchim® PuriFlash® C18 AQ column.

Strong anion exchange chromatography was performed using an automated ÄKTA pure™ system and QSepharose® Fast Flow (Sigma-Aldrich). Crude products were loaded as aq. solutions and eluted using increasing concentrations of aq. 1 M $NaClO_4$ solution.

Cation exchange for the preparation of TBA salts was performed with Dowex® 50WX8 H^+, followed by neutralization with TBA hydroxide and subsequent lyophilization or with a Chelex® 100 column preconditioned with TBA(Br) (500 mM).

Lyophilization was performed using a Christ Alpha 1-4 LDplus.

Centrifugation was performed with an Eppendorf 5804R.

NMR-spectroscopy: The ^{1}H-, ^{13}C-, ^{31}P-NMR spectra were measured on a Bruker Avance Neo 400 MHz (101 MHz for ^{13}C, 162 MHz for ^{31}P) NMR spectrometer with broadband CryoProbe Prodigy and Bruker Avance Neo 700 MHz (176 MHz for ^{13}C, 283 MHz for ^{31}P) NMR spectrometer with broadband CryoProbe Prodigy. All signals were referred to an internal solvent signal (^{1}H-NMR: $CDCl_3$: δ = 7.26 ppm, D_2O: δ = 4.79 ppm; ^{13}C-NMR: $CDCl_3$: δ = 77.16 ppm). The signals of ^{31}P-NMR spectra were referenced to an external standard. The chemical shifts are quoted in ppm. The splitting patterns are labeled as: singlet (s), broad singlet (br s), doublet (d), triplet (t), quartett (q), septet (sept), multiplet (m). The coupling constants J are given in Hertz (Hz). The evaluation of NMR-spectra was performed using the software MestreNova from Mestrelab Research.

High resolution mass spectrometry (HRMS) was conducted by C. Warth from the analytical department of the Institute of Organic Chemistry at the University of Freiburg on a Thermo LCQ Advantage (spray voltage: 2.5 – 4.5 kV, spray current: 5 µA, ion transfer tube: 250 (150) °C, evaporation temp.: 50-400 °C).

CE-ESI-MS experiments were performed on a bare-fused silica capillary, activated for 10 min with NaOH (1 M) before first measurement, with a length of 100 cm (50 µm internal diameter and 365 µm outer diameter) on an Agilent 7100 capillary electrophoresis system coupled to a qTOF (6520, Agilent) equipped with a commercial CE-MS adapter and sprayer kit from Agilent. 35 mM ammonium acetate titrated by ammonia solution to pH 9.75 was background electrolyte. Samples were injected by applying 100 mbar pressure for 10 s, followed by an injection of a background electrolyte plug by applying 50 mbar for 5 s. For each analysis, a constant CE current of 23 µA was established by applying 30 kV over the capillary. The sheath liquid was composed of a water-isopropanol (1:1) mixture spiked with mass references. It was introduced at a constant flowrate of 1.5 µL/min. ESI-qTOF-MS was conducted in the negative ionization mode with published settings.[1] Automatic recalibration of each acquired spectrum was performed using reference masses of reference standards (TFA anion, $[M-H]^-$, 112.9855), and (HP-0921, $[M-H+CH_3COOH]^-$, 980.0163. Data were processed using the Agilent CE ChemStation Software and smoothed (1×).

1.2 Synthesis of polyP$_8$ via water opening

1.2.1 c-PyPA 1

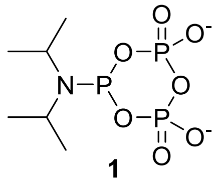

1

The compound was synthesized in two steps from pyrophosphate sodium salt as reported previously. Analytical data were identical to literature.[2] The compound was stored as stock solution (0.11 M in MeCN) at -20 °C.

1.2.2 polyP$_8$ 6

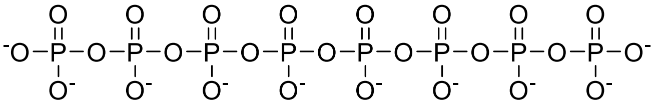

6

sodium salt

ETT (686 mg, 5.27 mmol, 7.0 eq.) was coevaporated with dry MeCN (3 ×). PP$_i$ × 2 TBA in MeCN (0.15 M, 5.0 mL, 0.75 mmol, 1.0 eq.) was added and reaction mixture was stirred for 5 min at r.t. The reaction mixture was cooled to -20 °C, c-PyPa **1** (0.11 M, 20 mL, 2.2 mmol, 2.9 eq.) was added, and stirred for 55 min (cooling bath removed after 10 min). Then *m*CPBA (70 %, 737 mg, 2.99 mmol, 4.0 eq.) was added at -10 °C and the reaction mixture was stirred for 40 min (cooling bath removed after 20 min). The reaction mixture was dropped into water (60 mL) at r.t. and the solution was stirred for 80 min. The crude product was precipitated by $NaClO_4$ (0.5 M in acetone, 100 mL), centrifuged, washed with cold acetone (3×) and purified by strong anion exchange chromatography (QSepharose® Fast Flow, increasing concentration of aq. $NaClO_4$-solution (1 M) in H_2O). Product containing fractions were combined, lyophilized, precipitated with cold $NaClO_4$ acetone solution (0.5 M, 35 mL), washed with cold acetone (3×) and dried to afford **6** (235 mg, 268 µmol, 36%) as a white solid.

^{31}P{^{1}H}-NMR (162 MHz, D_2O) δ = -8.70 – -10.58 (m, 2P), -21.80 – -22.59 (m, 6P) ppm.
HRMS CE-ESI calc. for $H_8O_{25}P_8$ [M-2H]$^{2-}$: 327,8639, found: 327,8638.

1.2.3 Di-Fm-P-amidite 7a

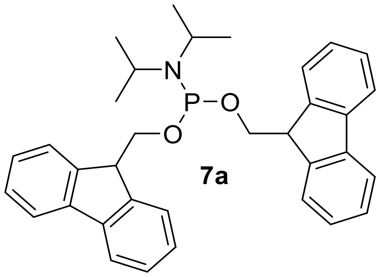

7a

(iPr)$_2$N-P(OFm)$_2$ **7a** was synthesized in two steps starting from PCl_3 as reported in literature. Analytical data were consistent with the values reported in literature.[3]

1.3 Synthesis of polyP$_9$ and polyP$_{10}$ via photocaged polyP$_{9/10}$

1.3.1 Fm-P-diamidite 38

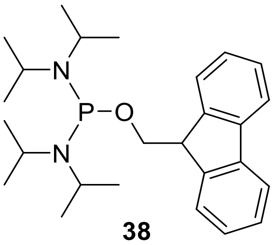

38

((iPr)$_2$N)$_2$-P(OFm) (**38**) was synthesized according to a reported procedure.[4] Analytical data were consistent with the values reported in literature.[4]

1.3.2 DEACM-OH 2

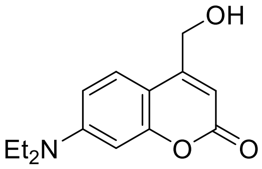

2

The compound **2** was synthesized according to literature procedures[5,6] in three steps from commercial available 7-diethylamino-4-methylcoumarin. Analytical data were identical to those reported.[5,6]

1.3.3 DEACM-Fm-P-amidite 7b

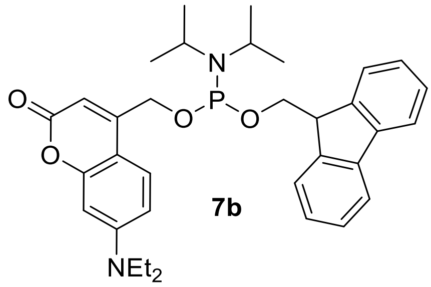

7b

The synthesis of **7b** is based on a previously reported procedure.[4]

Fm-P-diamidite **38** (88%, 433 mg, 893 µmol, 1.1 eq.), DEACM-OH **2** (200 mg, 809 µmol, 1.0 eq.) and ETT (116 mg, 891 µmol, 1.1 eq.) were separately coevaporated in dry MeCN (3 × 4 mL). Fm-P-diamidite **38** and DEACM-OH **2** were dissolved in dry THF (each 10 mL) and combined. The solution was cooled to 0 °C and ETT (dissolved in 4 mL dry THF) was added. The reaction mixture was stirred at 0 °C for 30 min. Subsequently the cooling bath was removed and the reaction mixture was stirred for further 60 min at r.t. Afterwards, dry Et_2O (20 mL) was added and the formed precipitate was filtered off over neutral Al_2O_3. The solvent was removed under reduced pressure and the crude product was purified by NP-MPLC (0-30% EtOAc with 3% NEt_3 in cyclohexane with 3% NEt_3) to obtain product **7b** (^{31}P-NMR purity: 89%; 184 mg, 286 µmol, 35%) as a yellow oil.

^{1}H-NMR (400 MHz, $CDCl_3$) δ = 7.73 (ddd, *J* = 7.7, 1.2, 1.2 Hz, 1H), 7.72 (ddd, *J* = 7.7, 1.2, 1.2 Hz, 1H), 7.65 (dddd, *J* = 7.4, 0.9, 0.9, 0.9 Hz, 1H), 7.60 (dddd, *J* = 7.5, 0.9, 0.9, 0.9 Hz, 1H), 7.40 – 7.34 (m, 2H), 7.32 – 7.27 (m, 2H), 7.25 (d, *J* = 8.9 Hz, 1H), 6.57 – 6.49 (m, 2H), 6.24 (t, *J* = 1.4 Hz, 1H), 4.73 – 4.59 (m, 2H), 4.21 – 4.07 (m, 2H), 3.89 (dt, *J* = 9.8, 6.8 Hz, 1H), 3.67 (hept, *J* = 6.7 Hz, 1H), 3.65 (hept, *J* = 6.7 Hz, 1H), 3.41 (q, *J* = 7.1 Hz, 4H), 1.21 (t, *J* = 7.0 Hz, 6H), 1.20 (d, *J* = 6.8 Hz, 6H), 1.16 (d, *J* = 6.8 Hz, 6H) ppm. **^{31}P{^{1}H}-NMR** (162 MHz, $CDCl_3$) δ = 148.31 (s) ppm. **^{31}P-NMR** (162 MHz, $CDCl_3$) δ = 148.30 (pseudo-hept, *J* = 7.5 Hz) ppm. ^{13}C-NMR (101 MHz, $CDCl_3$) δ = 162.4, 156.3, 153.1 (d, *J* = 7.2 Hz), 150.5, 144.9, 144.6, 141.5, 141.5, 127.6, 127.6, 127.1, 127.0, 125.4, 125.2, 124.6, 120.0, 119.9, 108.5, 106.6, 106.4, 97.9, 65.9 (d, *J* = 17.4 Hz), 61.5 (d, *J* = 18.2 Hz), 49.3 (d, *J* = 7.7 Hz), 44.9, 43.4, 43.3, 24.9, 24.8, 24.7, 24.7, 12.6 ppm. **HRMS ESI** calc. for $C_{34}H_{42}N_2O_4P$ $[M+H]^+$: 573.2877, found: 573.2887.

1.3.4 DEACM photocaged polyP$_9$ 8 and polyP$_{10}$ 9

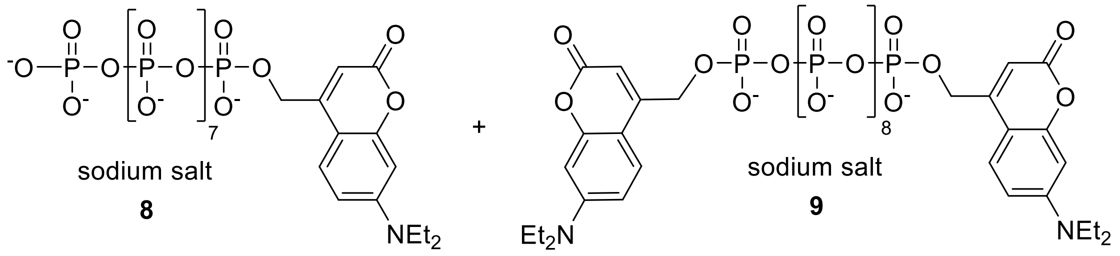

PolyP$_8$ × 4 TBA (68 mg, 42 µmol, 1.0 eq.), DEACM-Fm-P-amidite **7b** (^{31}P-NMR purity: 89%, 71 mg, 0.11 mmol, 2.6 eq.) and ETT (82 mg, 0.63 mmol, 15 eq.) were separately coevaporated with dry MeCN (3 × 2 mL). PolyP$_8$ × 4 TBA was dissolved in dry MeCN (3 mL) and was added to ETT. Subsequently, DEACM-Fm-P-amidite **7b** was dissolved in dry DCM (3 mL) and was to the reaction mixture. After stirring 30 min at r.t., the mixture was cooled to 0 °C and *m*CPBA (70%, 26 mg, 0.11 mmol, 2.6 eq.) was added. After 20 min DBU (0.3 mL, 5 Vol-%) was added dropwise at 0 °C and the reaction was stirred for 1 h at room temperature. Afterwards, the solution was added dropwise to cold ether (35 mL). The suspension was centrifuged, washed with cold ether (2 × 25 mL) and the dried crude product was purified by anion exchange chromatography (Q Sepharose® Fast Flow, increasing concentration of aq. $NaClO_4$-solution (1 M) in H_2O). Fractions eluted with 15-18% and fractions eluted with 18-20% aq. $NaClO_4$ were combined separately, lyophilized, precipitated with cold $NaClO_4$ acetone solution (0.5 M, 35 mL) and washed with acetone (3 × 15 mL) to afford mono-DEACM caged polyP$_9$ **8** (13 mg, 11 µmol, 26%) and bis-DEACM caged polyP$_{10}$ **9** (12 mg, 8.0 µmol, 19%) as a yellow sodium salts.

Mono-DEACM caged polyP$_9$ **8**:

^{1}H-NMR (400 MHz, D_2O) δ = 7.61 (d, *J* = 9.1 Hz, 1H), 6.90 (dd, *J* = 9.1, 2.6 Hz, 1H), 6.71 (d, *J* = 2.5 Hz, 1H), 6.35 (t, *J* = 1.1 Hz, 1H), 5.29 (dd, *J* = 7.9, 1.4 Hz, 2H), 3.49 (q, *J* = 7.1 Hz, 4H), 1.21 (t, *J* = 7.1 Hz, 6H) ppm. **^{31}P{^{1}H}-NMR** (162 MHz, D_2O) δ = -5.57 (d, *J* = 19.2 Hz, 1P), -11.12 (d, *J* = 16.7 Hz, 1P), -20.69 (dd, *J* = 18.9, 15.0 Hz, 1P), -21.10 – -22.58 (m, 6P) ppm. **^{31}P-NMR** (162 MHz, D_2O) δ = -5.57 (d, *J* = 18.7 Hz, 1P), -11.12 (dt, *J* = 16.1, 7.8 Hz, 1P), -20.70 (dd, *J* = 19.0, 14.9 Hz, 1P), -21.20 – -22.88 (m, 6P) ppm. **^{13}C-NMR** (101 MHz, $CDCl_3$) δ = 166.0, 155.7, 154.6 (d, *J* = 7.7 Hz), 151.4, 125.4, 110.3, 106.1, 104.0, 97.4, 63.8 (d, *J* = 4.6 Hz), 44.6, 11.6 ppm. **HRMS CE-ESI** calc. for $C_{14}H_{24}NO_{30}P_9$ $[M-2H]^{2-}$: 482.4016, found: 482.4016.

Bis-DEACM caged polyP$_{10}$ **9**:

^{1}H-NMR (400 MHz, D_2O) δ = 7.39 (d, *J* = 9.1 Hz, 2H), 6.73 (d, *J* = 9.5 Hz, 2H), 6.43 (s, 2H), 6.17 (s, 2H), 5.18 (d, *J* = 6.5 Hz, 4H), 3.42 (q, *J* = 7.0 Hz, 8H), 1.20 (t, *J* = 7.0 Hz, 12H) ppm. **^{31}P{^{1}H}-NMR** (162 MHz, D_2O) δ = -10.65 – -11.30 (m, 2P), -21.01 – -22.03 (m, 6P) ppm. **^{13}C-NMR** (101 MHz, $CDCl_3$) δ = 165.7, 155.3, 154.5 (d, *J* = 8.4 Hz), 151.1, 125.0, 110.0, 105.5, 103.1, 96.8, 63.7 (d, *J* = 4.0 Hz), 44.5, 11.8 ppm. **HRMS CE-ESI** calc. for $C_{28}H_{40}N_2O_{35}P_{10}$ $[M-2H]^{2-}$: 636.9399, found: 636.9410.

1.3.5 Bis-DEACM-polyP$_{10}$ photolysis intermediate 13

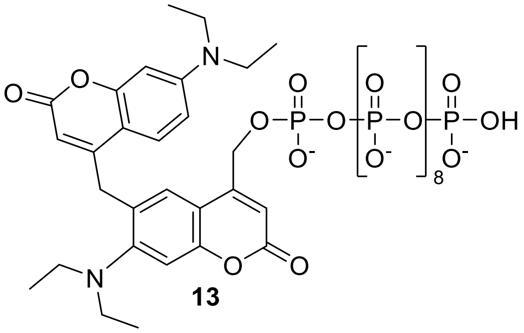

13

Bis-DEACM caged polyP$_{10}$ **9** (5.0 mg, 3.3 µmol, 2 mM in H_2O) was irradiated, under stirring, with a 400 nM LED for 90 min. The solution was diluted with H_2O (10 mL) and directly injected to strong anion exchange chromatography system for purification (Q Sepharose® Fast Flow, increasing concentrations of 1 M $NaClO_4$ in H_2O). Fractions eluted with 24-30% aqueous $NaClO_4$ were combined and concentrated by lyophilization. Precipitation in cold $NaClO_4$ acetone solution (0.5 M, 35 mL), washing with cold acetone (10 mL) and drying afforded **13** (1.2 mg, 0.80 µmol, 24%) as an orange solid.

^{1}H-NMR (700 MHz, D_2O) δ = 7.66 (d, *J* = 9.1 Hz, 1H), 7.65 (s, 1H), 7.33 (s, 1H), 6.81 (dd, *J* = 9.2, 2.6 Hz, 1H), 6.71 (d, *J* = 2.6 Hz, 1H), 6.67 (t, *J* = 1.6 Hz, 1H), 5.76 (t, *J* = 0.9 Hz, 1H), 5.31 (d, J = 1.6 Hz, 2H), 4.31 (s, 2H), 3.48 (q, *J* = 7.1 Hz, 4H), 3.10 (q, *J* = 7.1 Hz, 4H), 1.20 (t, *J* = 7.1 Hz, 6H), 0.96 (t, *J* = 7.1 Hz, 6H) ppm. **^{31}P{^{1}H}-NMR** (283 MHz, D_2O) δ = -5.36 (br s, 1P), -11.13 (d, *J* = 17.4 Hz, 1P), -20.47 – -22.75 (m, 8P) ppm. **^{13}C-NMR** (176 MHz, $CDCl_3$) δ = 165.9, 164.9, 159.4, 155.7, 154.0, 153.8 (d, J = 7.8 Hz), 153.0, 151.3, 130.7, 126.6, 126.3, 112.9, 110.8, 110.0, 109.2, 108.5,

106.3, 97.5, 63.6 (d, J = 4.5 Hz), 47.2, 44.6, 33.7, 11.6, 11.1 ppm. **HRMS CE-ESI** calc. for $C_{28}H_{40}N_2O_{35}P_{10}$ $[M\text{-}2H]^{2-}$: 636.9399, found: 636.9405.

1.3.6 polyP$_9$ 10 and polyP$_{10}$ 11

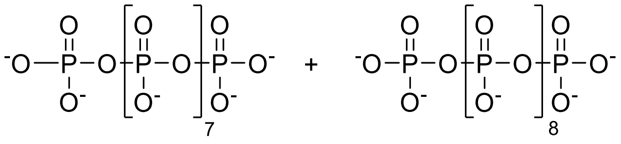

sodium salt **10**   sodium salt **11**

Mono-DEACM caged polyP$_9$ **8** (5.0 mg, 4.2 µmol, 2 mM in H_2O) or bis-DEACM caged polyP$_{10}$ **9** (3.0 mg, 2.0 µmol, 2 mM in H_2O) were irradiated, under stirring, with a 400 nM LED for 90 min or 8 h, respectively. The solution was diluted with H_2O (10 mL) and directly injected to strong anion exchange chromatography system for purification (Q Sepharose® Fast Flow, increasing concentrations of 1 M $NaClO_4$ in H_2O). Fractions eluted with 10-11% aqueous $NaClO_4$ or 11-13%, respectively, were combined and concentrated by lyophilization. Precipitation in cold $NaClO_4$ acetone solution (0.5 M, 35 mL), washing with cold acetone (3 × 10 mL) and drying afforded polyP$_9$ **10** (1.6 mg, 1.6 µmol, 38%) or polyP$_{10}$ **11** (0.55 mg, 0.51 µmol, 26%) as a beige solid.

polyP$_9$ **10**:

^{31}P-NMR (162 MHz, D_2O) δ = -5.39 (br s, 2P), -20.01 – -22.92 (m, 7P) ppm. **HRMS ESI** calc. for $P_9H_9O_{28}$ $[M\text{-}2H]^{2-}$: 367.8465, found: 367.8466.

polyP$_{10}$ **11**:

^{31}P-NMR (162 MHz, D_2O) δ = -4.78 – -5.64 (m, 2P), -19.89 – -23.41 (m, 8P) ppm. **HRMS ESI** calc. for $P_{10}H_{10}O_{31}$ $[M\text{-}2H]^{2-}$: 407.8297, found: 407.8299.

1.4 Synthesis of ^{18}O-labeled polyP$_9$ and polyP$_{10}$

1.4.1 $^{18}O_2$-4-nitrobenzoic acid (31)

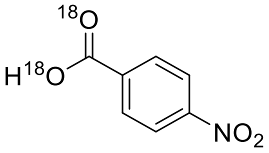

31

The compound was synthesized from 4-nitrobenzonitrile and $H_2{}^{18}O$ (^{18}O = 99%) as reported in literature.[7] Analytical data were consistent with those reported.[7]

1.4.2 $^{18}O_2$-4-nitrobenzoic-DEACM-ester 32

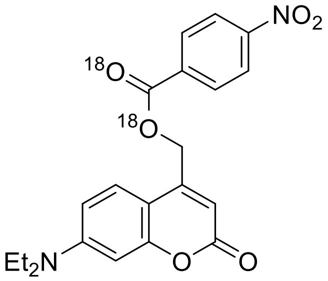

32

DEACM-OH **2** (1.00 g, 4.04 mmol, 1.0 eq.) and $^{18}O_2$-4-nitrobenzoic acid (**31**) (0.69 g, 4.0 mmol, 1.0 eq.) were dissolved in THF (40 mL). PPh_3 (1.27 g, 4.84 mmol, 1.2 eq.) was added and the solution was cooled to 0 °C. DIAD (0.95 mL, 0.98 g, 4.9 mmol, 1.2 eq.) was added dropwise and the solution was stirred for 3 h at r.t. The solvent was removed under reduced pressure and the crude product was purified by flash chromatography (DCM:MeOH, 98:2). Compound **32** was obtained as a yellow solid (0.97 g, 2.4 mmol, 59%).

^{1}H-NMR (400 MHz, $CDCl_3$) δ = 8.36 – 8.26 (m, 4H), 7.36 (d, *J* = 9.0 Hz, 1H), 6.61 (dd, *J* = 9.0, 2.6 Hz, 1H), 6.54 (d, *J* = 2.6 Hz, 1H), 6.21 (t, *J* = 1.2 Hz, 1H), 5.51 (d, *J* = 1.2 Hz, 2H), 3.43 (q, *J* = 7.1 Hz, 5H), 1.22 (t, *J* = 7.1 Hz, 6H) ppm. **^{13}C-NMR** (101 MHz, $CDCl_3$) δ = 164.1, 161.8, 156.6, 151.1, 151.0, 148.8, 134.7, 131.1, 124.5, 123.9, 108.9, 106.9, 106.0, 98.1, 62.8, 44.9, 12.6 ppm. **HRMS ESI** calc. for $C_{21}H_{21}N_2O_4{}^{18}O_2$ $[M+H]^+$: 401.1479, found: 401.1482.

1.4.3 DEACM-^{18}OH 3

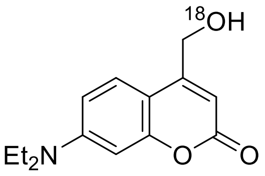

3

Ester **32** (860 mg, 2.15 mmol, 1.0 eq.) was dissolved in a mixture of MeOH/THF (1:1, 14 mL) and the solution was cooled to 0 °C. LiOH • H_2O (99 mg, 2.4 mmol, 1.1 eq.) and H_2O (2.2 mL) were added and the reaction mixture was stirred at r.t. for 2 h. H_2O (40 mL) was added and the solution was extracted with DCM (3 × 40 mL). The organic layer was washed with sat. NaCl (80 mL), dried over Na_2SO_4 and concentrated under reduced pressure. The crude product was purified by flash chromatography (DCM:MeOH, 98:2). Compound **3** was obtained as a yellow solid (514 mg, 2.06 mmol, 96%).

^{1}H-NMR (400 MHz, $CDCl_3$) δ = 7.32 (d, *J* = 9.0 Hz, 1H), 6.57 (dd, *J* = 9.0, 2.6 Hz, 1H), 6.52 (d, *J* = 2.6 Hz, 1H), 6.25 (t, *J* = 1.4 Hz, 1H), 4.83 (dd, *J* = 6.2, 1.3 Hz, 2H), 3.41 (q, *J* = 7.1 Hz, 4H), 1.83 (t, *J* = 6.1 Hz, 1H), 1.21 (t, *J* = 7.1 Hz, 6H) ppm. **^{13}C-NMR** (101 MHz, $CDCl_3$) δ = 162.5, 156.4, 154.3, 150.7, 124.5, 108.7, 106.4, 105.8, 98.0, 61.3, 44.9, 12.6 ppm. **HRMS ESI** calc. for $C_{14}H_{18}NO_2{}^{18}O$ $[M+H]^+$: 250.1324, found: 250.1324. **Isotope enrichment** (CE-ESI-QTOF, $[M-H]^-$, 248.1178 ($^{18}O_1$) / 246.1136 ($^{18}O_0$)): 98:2.

1.4.4 ^{18}O-DEACM-Fm-P-amidite 33

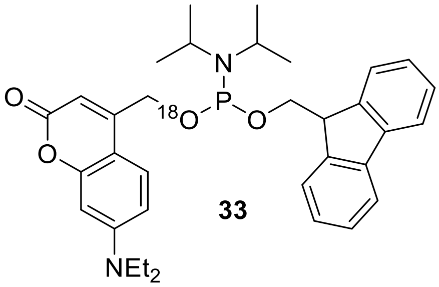

33

Fm-P-diamidite **38** (88%, 214 mg, 441 µmol, 1.2 eq.), DEACM-^{18}OH **3** (90 mg, 361 µmol, 1.0 eq.) and ETT (116 mg, 891 µmol, 1.1 eq.) were separately coevaporated in dry MeCN (3 × 2 mL). Fm-P-diamidit **38** and DEACM-^{18}OH **3** were

dissolved in dry THF (each 10 mL) and combined. The solution was cooled to 0 °C and ETT (dissolved in 2 mL dry THF) was added. The reaction mixture was stirred at 0 °C for 30 min. Subsequently the cooling bath was removed and the reaction mixture was stirred for further 60 min at r.t. Afterwards, dry Et_2O (10 mL) was added and the formed precipitate was filtered off over neutral Al_2O_3. The solvent was removed under reduced pressure to obtain product **33** (^{31}P-NMR purity: 72%; 202 mg, 253 µmol, 70%) as a yellow oil. A small portion of **33** crystallized in a mixture of EtOAc and n-pentane overnight at -20 °C affording **33** with a ^{31}P-NMR purity of 91%.

^{1}H-NMR (400 MHz, $CDCl_3$) δ = 7.73 (ddd, *J* = 7.6, 1.1, 1.1 Hz, 1H), 7.73 (ddd, *J* = 7.6, 1.1, 1.1 Hz, 1H), 7.65 (dddd, *J* = 7.4, 0.9, 0.9, 0.9 Hz, 1H), 7.60 (dddd, *J* = 7.5, 0.9, 0.9, 0.9 Hz, 1H), 7.40 – 7.34 (m, 2H), 7.32 – 7.27 (m, 2H), 7.25 (d, *J* = 9.0 Hz, 1H), 6.57 – 6.49 (m, 2H), 6.24 (t, *J* = 1.4 Hz, 1H), 4.73 – 4.59 (m, 2H), 4.21 – 4.16 (m, 1H), 4.11 – 4.07 (m, 1H), 3.89 (dt, *J* = 9.8, 6.9 Hz, 1H), 3.67 (hept, *J* = 6.9 Hz, 1H), 3.65 (hept, *J* = 6.9 Hz, 1H), 3.41 (q, *J* = 7.1 Hz, 4H), 1.21 (t, *J* = 7.1 Hz, 6H), 1.20 (d, *J* = 6.9 Hz, 6H), 1.16 (d, *J* = 6.8 Hz, 6H) ppm. **^{31}P{^{1}H}-NMR** (162 MHz, $CDCl_3$) δ = 148.26 (s) ppm. **^{31}P-NMR** (162 MHz, $CDCl_3$) δ = 148.26 (pseudo-hept, *J* = 7.5 Hz) ppm. ^{13}C-NMR (101 MHz, $CDCl_3$) δ = 162.4, 156.3, 153.1 (d, *J* = 7.2 Hz), 150.5, 144.9, 144.6, 141.5, 141.5, 127.6, 127.6, 127.1, 127.0, 125.5, 125.2, 124.6, 120.0, 119.9, 108.5, 106.6, 106.4, 97.9, 65.9 (d, *J* = 17.4 Hz), 61.5 (d, *J* = 18.2 Hz), 49.3 (d, *J* = 7.7 Hz), 44.9, 43.4, 43.3, 24.9, 24.8, 24.7, 24.7, 12.6 ppm. **HRMS ESI** calc. for $C_{34}H_{43}N_2O_3{}^{18}OP$ $[M+H]^+$: 575.2919, found: 575.2926.

1.4.5 ^{18}O-labeled DEACM photocaged polyP$_9$ 34 and polyP$_{10}$ 35

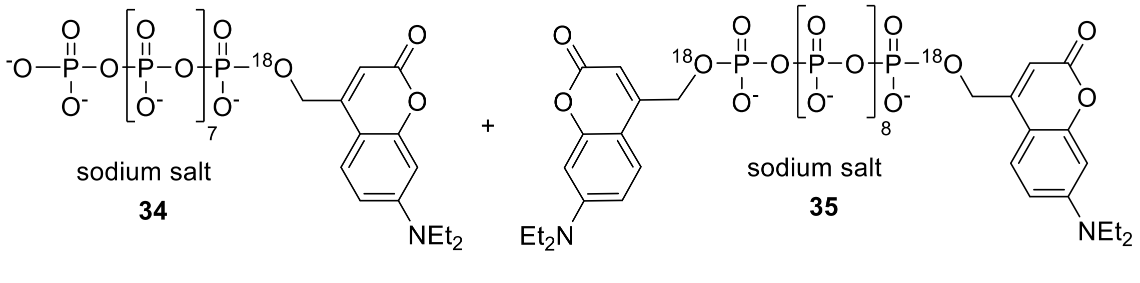

PolyP$_8$ × 4 TBA (synthesis of **34**: 68 mg, 42 µmol, 1.0 eq.; synthesis of **35**: 82 mg, 51 µmol, 1.0 eq.), ^{18}O-DEACM-Fm-P-amidite **33** (synthesis of **34**: ^{31}P-NMR purity: 94%, 38 mg, 62 µmol, 1.5 eq.; synthesis of **35**: ^{31}P-NMR purity: 72%, 100 mg, 125 µmol, 2.5 eq.) and ETT (synthesis of **34**: 82 mg, 0.63 mmol, 15 eq.; synthesis of

35: 98 mg, 0.75 mmol, 15 eq.) were separately coevaporated with dry MeCN (3 × 2 mL). PolyP$_8$ × 4 TBA was dissolved in dry MeCN (3 mL) and was added to ETT. Subsequently, ^{18}O-DEACM-Fm-P-amidite **33** was dissolved in dry DCM (3 mL) and was to the reaction mixture. After stirring 30 min at r.t., the mixture was cooled to 0 °C and *m*CPBA (synthesis of **34**: 16 mg, 65 µmol, 1.5 eq.; synthesis of **35**: 70%, 32 mg, 130 µmol, 2.5 eq.) was added. After 20 min DBU (0.3 mL, 5 Vol-%) was added dropwise at 0 °C and the reaction was stirred for 1 h at r.t. Afterwards, the solution was added dropwise to cold ether (35 mL). The suspension was centrifuged, washed with cold ether (2 × 25 mL) and the dried crude product was purified by anion exchange chromatography (Q Sepharose® Fast Flow, increasing concentration of aq. $NaClO_4$-solution (1 M) in H_2O). Fractions eluted with 17-18% (synthesis of **34**) or fractions eluted with 18-20% (synthesis of **35**) aq. $NaClO_4$ were combined separately, lyophilized, precipitated with cold $NaClO_4$ acetone solution (0.5 M, 35 mL) and washed with acetone (3 × 15 mL) to afford mono-^{18}O-DEACM caged polyP$_9$ **34** (10 mg, 8.4 µmol, 20%) or bis-^{18}O$_2$-DEACM caged polyP$_{10}$ **35** (30 mg, 20 µmol, 39%) as a yellow sodium salts.

Mono-^{18}O-DEACM caged polyP$_9$ **34**:

^{1}H-NMR (400 MHz, D_2O) δ = 7.63 (d, *J* = 9.1 Hz, 1H), 6.91 (dd, *J* = 9.1, 2.6 Hz, 1H), 6.73 (d, *J* = 2.6 Hz, 1H), 6.36 (t, *J* = 0.9 Hz, 1H), 5.30 (dd, *J* = 7.8, 1.4 Hz, 2H), 3.50 (q, *J* = 7.1 Hz, 4H), 1.22 (t, *J* = 7.1 Hz, 6H) ppm. **^{31}P{^{1}H}-NMR** (162 MHz, D_2O) δ = -5.45 (d, *J* = 18.9 Hz, 1P), -11.14 (d, *J* = 17.0 Hz, 1P), -20.66 (dd, *J* = 18.1 , 14.6 Hz, 1P), -21.18 – -22.85 (m, 6P) ppm. **^{31}P-NMR** (162 MHz, D_2O) δ = -5.45 (d, *J* = 19.2 Hz, 1P), -11.14 (dt, *J* = 16.3, 7.7 Hz, 1P), -20.66 (dd, *J* = 17.8, 15.0 Hz, 1P), -21.13 – -22.61 (m, 6P) ppm. **^{13}C-NMR** (101 MHz, $CDCl_3$) δ = 166.0, 155.7, 154.6, 151.4, 125.4, 110.3, 106.1, 104.0, 97.5, 63.8 (d, *J* = 5.2 Hz), 44.6, 11.6 ppm. **HRMS CE-ESI** calc. for $C_{14}H_{24}NO_{29}{}^{18}OP_9$ $[M-2H]^{2-}$: 483,4037, found: 483,4033.

Bis-^{18}O$_2$-DEACM caged polyP$_{10}$ **35**:

^{1}H-NMR (400 MHz, D_2O) δ = 7.40 (d, *J* = 9.1 Hz, 2H), 6.74 (dd, *J* = 9.5, 2.5 Hz, 2H), 6.45 (d, *J* = 2.5 Hz, 2H), 6.19 (s, 2H), 5.18 (d, *J* = 7.0 Hz, 4H), 3.43 (q, *J* = 7.0 Hz, 8H), 1.20 (t, *J* = 7.1 Hz, 12H) ppm. **^{31}P{^{1}H}-NMR** (162 MHz, D_2O) δ = -10.84 – -11.28 (m, 2P), -21.15 – -21.98 (m, 6P) ppm. **^{13}C-NMR** (101 MHz, $CDCl_3$) δ = 165.7, 155.3, 154.5 (d, *J* = 8.4 Hz), 151.1, 125.0, 110.0, 105.6, 103.1, 96.8, 63.7 (d, *J* = 4.6 Hz), 44.5,

11.8 ppm. **HRMS CE-ESI** calc. for $C_{28}H_{40}N_2O_{33}{}^{18}O_2P_{10}$ $[M-2H]^{2-}$: 638.9442, found: 638.9442.

1.4.6 ^{18}O-labeld polyP$_9$ 36 and polyP$_{10}$ 37

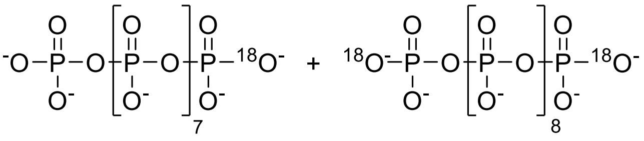

sodium salt **36** sodium salt **37**

Mono-^{18}O-DEACM caged polyP$_9$ **34** (5.0 mg, 4.2 µmol, 2 mM in H_2O) or bis-$^{18}O_2$-DEACM caged polyP$_{10}$ **35** (12 mg, 8.0 µmol, 2 mM in H_2O) were irradiated, under stirring, with a 400 nM LED for 2 h or 8 h, respectively. The solution was diluted with H_2O (10 mL) and directly injected to strong anion exchange chromatography system for purification (Q Sepharose® Fast Flow, increasing concentrations of 1 M $NaClO_4$ in H_2O). Fractions eluted with 9-11% aqueous $NaClO_4$ or 10-13%, respectively, were combined and concentrated by lyophilization. Precipitation in cold $NaClO_4$ acetone solution (0.5 M, 35 mL), washing with cold acetone (3 × 10 mL) and drying afforded ^{18}O-polyP$_9$ **36** (80% purity, 2.1 mg, 1.7 µmol, 41%) or ^{18}O-polyP$_{10}$ **37** (83% purity, 5.3 mg, 4.1 µmol, 51%) as a beige solid.

^{18}O-polyP$_9$ **36**:

^{31}P-NMR (162 MHz, D_2O) δ = -4.72 – -5.87 (m, 2P), -20.05 – -23.28 (m, 8P) ppm. **HRMS ESI** calc. for $P_9H_9O_{27}{}^{18}O$ $[M-2H]^{2-}$: 368.8486, found: 368.8488. **Isotope enrichment** (CE-ESI-QTOF, $[M-2H]^{2-}$, 368.8486 ($^{18}O_1$) / 367.8465 ($^{18}O_0$)): 97:3.

$^{18}O_2$-polyP$_{10}$ **37**:

^{31}P-NMR (162 MHz, D_2O) δ = -6.32 (br s, 2P), -20.48 – -22.34 (m, 8P) ppm. **HRMS ESI** calc. for $P_{10}H_{10}O_{29}{}^{18}O_2$ $[M-2H]^{2-}$: 409.8339, found: 409.8339. (CE-ESI-QTOF, $[M-2H]^{2-}$, 409.8339 ($^{18}O_2$) / 408.8318 ($^{18}O_1$) / 407.8297 ($^{18}O_0$)): 95:5:0.

1.5 Synthesis of DEACM-CH_2 protected polyP$_{10}$

1.5.1 (*E*)-7-(Diethylamino)-4-[2-(dimethylamino)vinyl]-2*H*-chromen-2-one (19)

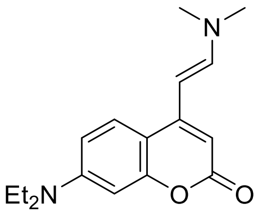

19

Enamine **19** was synthesized according a literature procedure[5] in one steps from commercial available 7-diethylamino-4-methylcoumarin. Analytical data were identical to literature.[5]

1.5.2 2-(7-(diethylamino)-2-oxo-2H-chromen-4-yl)acetaldehyde (20)

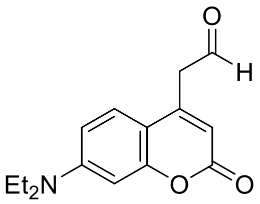

20

Enamine **19** (2.0 g, 7.0 mmol, 1.0 eq.) was dissolved in THF/water (3:1, 60 mL). *p*-Toluenesulfonic acid × H_2O (1.33 g, 6.99 mmol, 1.0 eq.) was added and the reaction mixture was stirred at 70 °C for 3 h before it was partitioned between EtOAc and water. The aqueous phase was extracted with EtOAc (2×). The combined organic layers were washed with brine (2×) and were dried over Na_2SO_4 before the solvent was removed under reduced pressure. The crude product of aldehyde **20** (2.0 g, 7.0 mmol, quant.) was obtained as a brown solid and was used without further purification in the next step.

^{1}H-NMR (400 MHz, $CDCl_3$) δ = 9.71 (t, *J* = 2.0 Hz, 1H), 7.23 (d, *J* = 9.0 Hz, 1H), 6.56 (dd, *J* = 9.0, 2.6 Hz, 1H), 6.49 (d, *J* = 2.6 Hz, 1H), 5.99 (t, *J* = 0.8 Hz, 1H), 3.72 (dd, *J* = 2.1, 0.8 Hz, 2H), 3.40 (q, *J* = 7.1 Hz, 4H), 1.19 (t, *J* = 7.1 Hz, 6H) ppm. **^{13}C-NMR** (101 MHz, $CDCl_3$) δ = 196.6, 161.7, 156.6, 151.1, 146.9, 125.7, 110.5, 109.0, 108.1, 98.0, 47.0, 45.0, 12.5 ppm. **HRMS ESI** calc. for $C_{15}H_{18}NO_3$ $[M+H]^+$: 260.1281, found: 260.1285.

1.5.3 DEACM-CH_2-OH 21

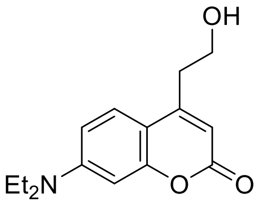

21

To a solution of the crude product of aldehyde **20** (3.6 g, 14 mmol, 1.0 eq.) in dry THF (320 mL) $NaBH_4$ (1.2 g, 32 mmol, 2.3 eq.) was added at 0 °C. Afterwards the cooling bath was removed and the reaction mixture was stirred overnight at room temperature. Subsequently, sat. aq. $NaHCO_3$-solution (320 mL) was added. The aqueous layer was washed with EtOAc (2 ×) and the combined organic layers were washed with brine and dried over $MgSO_4$ before the solvent was removed under reduced pressure. The crude product was purified by flash chromatography (cyclohexane/EtOAc 1:3 containing NEt_3) to obtain DEACM-CH_2-OH **21** (2.28 g, 8.73 mmol, 62%) as a yellow solid.

^{1}H-NMR (400 MHz, $CDCl_3$) δ = 7.43 (d, *J* = 9.0 Hz, 1H), 6.58 (dd, *J* = 9.0, 2.6 Hz, 1H), 6.49 (d, *J* = 2.6 Hz, 1H), 5.99 (s, 1H), 3.96 (t, *J* = 6.5 Hz, 2H), 3.40 (q, *J* = 7.1 Hz, 4H), 2.95 (td, *J* = 6.5, 0.8 Hz, 2H), 1.20 (t, *J* = 7.1 Hz, 6H). **^{13}C-NMR** (101 MHz, $CDCl_3$) δ = 162.3, 156.5, 153.6, 150.8, 125.5, 108.7, 108.7, 108.4, 98.0, 61.5, 44.9, 34.9, 12.6 ppm. **HRMS ESI** calc. for $C_{15}H_{20}NO_3$ $[M+H]^+$: 262.1438, found: 262.1438.

1.5.4 DEACM-CH_2-Fm-P-amidite 22

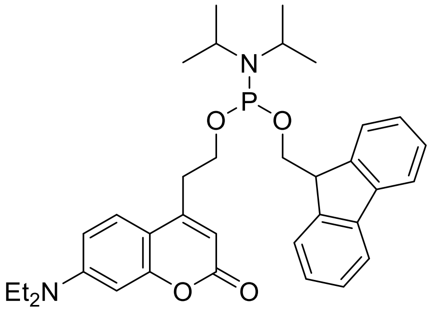

22

Fm-P-diamidite **38** (97%, 870 mg, 1.98 mmol, 1.1 eq.) and DEACM-CH_2-OH **21** (472 mg, 1.81 mmol, 1.0 eq.) were separately coevaporated in dry MeCN (3 × 4 mL). Both were dissolved in dry THF (each 10 mL) and combined. The solution was cooled

to 0 °C and tetrazole (0.45 M in MeCN, 4.8 mL, 2.2 mmol, 1.2 eq.) was added. The reaction mixture was stirred at 0 °C for 30 min. Subsequently the cooling bath was removed and the reaction mixture was stirred for further 20 min at r.t. Afterwards, dry Et_2O (10 mL) was added and the formed precipitate was filtered off over neutral Al_2O_3 under argon. The solvent was removed under reduced pressure and the crude product was purified by NP-MPLC (0-30% EtOAc with 3% NEt_3 in cyclohexane with 3% NEt_3) to obtain product **22** (^{31}P-NMR purity: 100%; 581 mg, 990 µmol, 55%) as a yellow oil.

^{1}H-NMR (400 MHz, $CDCl_3$) δ = 7.73 (ddd, *J* = 7.5, 1.0, 1.0 Hz, 1H), 7.73 (ddd, *J* = 7.5, 1.0, 1.0 Hz, 1H), 7.64 (dddd, *J* = 7.4, 0.9, 0.9, 0.9 Hz, 1H), 7.60 (dddd, *J* = 7.4, 1.0, 1.0, 1.0 Hz, 1H), 7.40 – 7.34 (m, 3H), 7.33 – 7.26 (m, 2H), 6.52 – 6.48 (m, 2H), 5.97 (t, *J* = 0.9 Hz, 1H), 4.15 (t, *J* = 6.7 Hz, 1H), 4.03 (ddd, *J* = 9.9, 6.6, 6.6 Hz, 1H), 3.91 – 3.78 (m, 3H), 3.59 (hept, *J* = 6.8 Hz, 1H), 3.56 (hept, *J* = 6.8 Hz, 1H), 3.38 (q, *J* = 7.1 Hz, 4H), 2.92 (tdd, *J* = 6.9, 0.8, 0.8 Hz, 2H), 1.18 (t, *J* = 7.0 Hz, 6H), 1.13 (d, *J* = 6.8 Hz, 6H), 1.12 (d, *J* = 6.8 Hz, 6H) ppm. **^{31}P{^{1}H}-NMR** (162 MHz, $CDCl_3$) δ = 146.77 (s) ppm. **^{31}P-NMR** (162 MHz, $CDCl_3$) δ = 146.77 (pseudo-hept, *J* = 7.1 Hz) ppm. **^{13}C-NMR** (101 MHz, $CDCl_3$) δ = 162.3, 156.5, 153.6, 150.6, 145.0, 144.7, 141.5, 141.4, 127.6, 127.5, 127.0, 127.0, 125.7, 125.5, 125.2, 120.0, 119.9, 108.9, 108.6 108.6, 98.0, 66.0 (d, *J* = 17.7 Hz), 62.1 (d, *J* = 17.5 Hz), 49.3 (d, *J* = 7.7 Hz), 44.8, 43.2, 43.1, 33.7 (d, *J* = 6.5 Hz), 24.8, 24.8, 24.7, 24.7, 12.6 ppm. **HRMS ESI** calc. for $C_{35}H_{44}N_2O_4P$ $[M+H]^+$: 587.3033, found: 587.3037.

1.5.5 Bis-DEACM-CH_2 protected polyP$_{10}$ 23

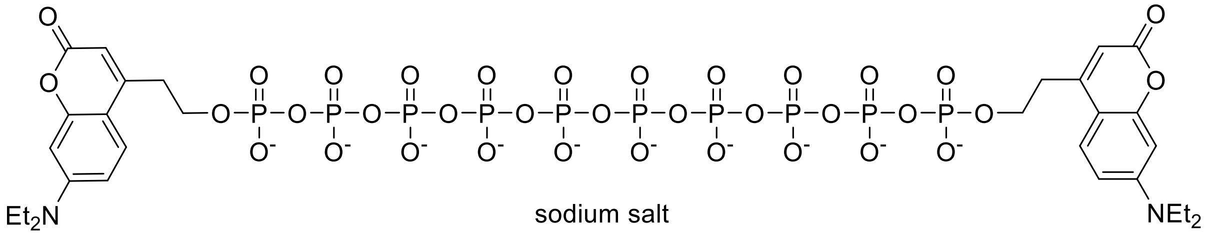

23

PolyP$_8$ × 4 TBA (101 mg, 63.0 µmol, 1.0 eq.), DEACM-CH_2-Fm-P-amidite **22** (^{31}P-NMR purity: 100%, 109 mg, 186 µmol, 3.0 eq.) and ETT (162 mg, 1.25 mmol, 20 eq.) were separately coevaporated with dry MeCN (3 × 3 mL). PolyP$_8$ × 4 TBA was dissolved in dry MeCN (4 mL) and was added to ETT. Subsequently, DEACM-CH_2-Fm-P-amidite **22** was dissolved in dry DCM (4 mL) and was to the reaction mixture.

After stirring 40 min at r.t, the mixture was cooled to 0 °C and *m*CPBA (70%, 46.5 mg, 189 µmol, 3.0 eq.) was added. After 20 min DBU (0.4 mL, 5 Vol-%) was added dropwise at 0 °C and the reaction was stirred for 1 h at r.t. Afterwards, the solution was added dropwise to cold ether (60 mL). The suspension was centrifuged, washed with cold ether (2 × 35 mL) and the dried crude product was purified by anion exchange chromatography (Q Sepharose® Fast Flow, increasing concentration of aq. $NaClO_4$-solution (1 M) in H_2O). Fractions eluted with 22-28% aq. $NaClO_4$ were combined, lyophilized, precipitated with cold $NaClO_4$ acetone solution (0.5 M, 35 mL) and washed with acetone (2 × 30 mL) to afford bis-DEACM-CH_2 protected $polyP_{10}$ **23** (8.0 mg, 5.3 µmol, 8%) as a yellow sodium salt.

^{1}H-NMR (400 MHz, D_2O) δ = 7.54 (d, *J* = 9.1 Hz, 2H), 6.75 (dd, *J* = 9.1, 2.6 Hz, 2H), 6.45 (d, *J* = 2.5 Hz, 2H), 6.06 (s, 2H), 4.31 (dt, *J* = 6.6, 6.6 Hz, 4H), 3.43 (q, *J* = 7.1 Hz, 8H), 3.11 (t, *J* = 6.2 Hz, 4H), 1.20 (t, *J* = 7.1 Hz, 12H) ppm. **^{31}P{^{1}H}-NMR** (162 MHz, D_2O) δ = -10.87 (d, *J* = 17.1 Hz, 2P), -21.20 – -22.28 (m, 8P) ppm. **^{31}P-NMR** (162 MHz, D_2O) δ = -10.87 (dt, *J* = 16.3, 7.4 Hz, 2P), -21.32 – -22.17 (m, 8P) ppm. **^{13}C-NMR** (101 MHz, D_2O) δ = 165.6, 156.4, 155.5, 151.2, 126.1, 110.0, 108.1, 106.3, 97.0, 64.5 (d, *J* = 5.9 Hz), 44.5, 32.2 (d, *J* = 7.5 Hz), 11.8 ppm. HRMS CE-ESI calc. for $C_{30}H_{44}N_2O_{35}P_{10}$ $[M-2H]^{2-}$: 650.9556, found: 650.9557.

1.6 Synthesis of $DEAC_{450}$ caged $polyP_{10}$'s

1.6.1 $DEAC_{450}$ 4

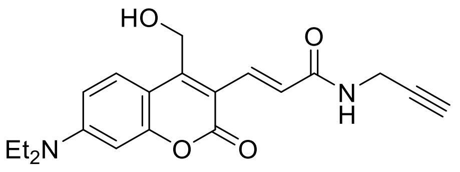

4

$DEAC_{450}$ **4** was synthesized according to literature.[8] Analytical data were identical to those reported.[8]

1.6.2 DEAC450-Fm-P-amidite 39

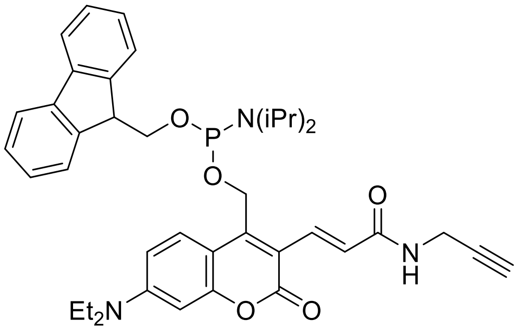

39

((iPr)$_2$N)P(OFm)(ODEAC$_{450}$) **39** was synthesized according to a reported procedure.[8] Analytical data were identical to literature.[8]

1.6.3 Bis-DEAC450 photocaged polyP10 24

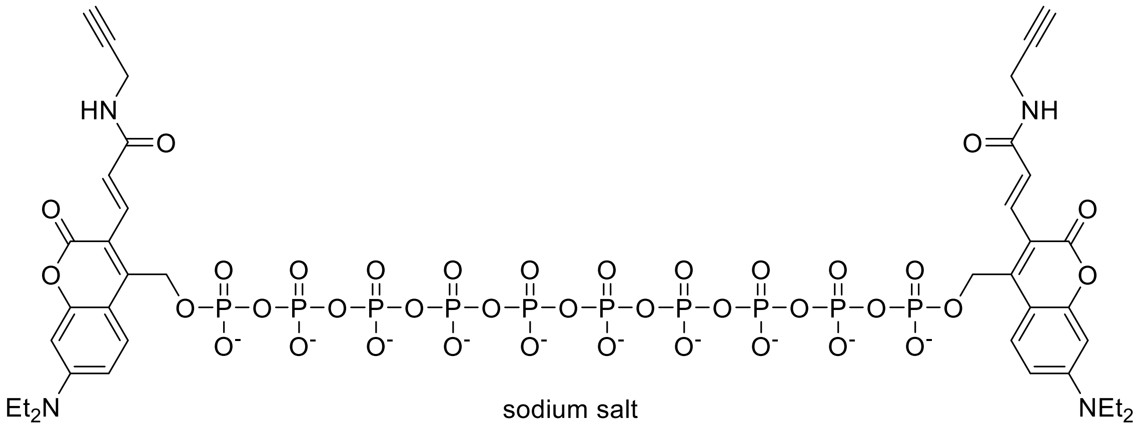

24

PolyP$_8$ x 5 TBA (89 mg, 48 µmol, 1.0 eq.), DEAC$_{450}$-Fm-P-amidite **39** (^{31}P-NMR purity: 80%, 122 mg, 144 µmol, 3.0 eq.) and ETT (125 mg, 960 µmol, 20 eq.) were separately coevaporated with dry MeCN (3 × 2 mL). PolyP$_8$ × 5 TBA was dissolved in dry MeCN (4 mL) and was added to ETT. Subsequently, DEAC$_{450}$-Fm-P-amidite **39** was dissolved in dry DCM (4 mL) and was added to the reaction mixture. After stirring 30 min at r.t. protected from light, the mixture was cooled to 0 °C and *m*CPBA (70%, 35 mg, 142 µmol, 3.0 eq.) was added. After 20 min DBU (0.4 mL, 5 Vol-%) was added dropwise at 0 °C and the reaction was stirred for 1 h at r.t. Afterwards, the solution was added dropwise to cold ether (60 mL). The suspension was centrifuged, washed with cold ether (2 × 25 mL) and the dried crude product was purified by anion exchange

chromatography (Q Sepharose® Fast Flow, increasing concentration of aq. $NaClO_4$-solution (1 M) in H_2O). Fractions eluted with 20-25% aq. $NaClO_4$ were combined, lyophilized, precipitated with cold $NaClO_4$ acetone solution (0.5 M, 35 mL) and washed with acetone (2 × 15 mL) to afford bis-DEAC450 caged polyP10 **24** (39 mg, 23 µmol, 48%) as an orange sodium salt.

^{1}H-NMR (400 MHz, D_2O) δ = 7.68 (d, *J* = 9.4 Hz, 2H), 7.37 (d, *J* = 15.7 Hz, 2H), 6.80 (d, *J* = 15.6 Hz, 2H), 6.75 (dd, *J* = 9.4, 2.5 Hz, 2H), 6.31 (d, *J* = 2.5 Hz, 2H), 5.01 (d, *J* = 5.5 Hz, 4H), 4.09 (d, *J* = 2.4 Hz, 4H), 3.37 (q, *J* = 7.0 Hz, 8H), 2.70 (t, *J* = 2.5 Hz, 2H), 1.18 (t, *J* = 7.0 Hz, 12H) ppm. **^{31}P{^{1}H}-NMR** (162 MHz, D_2O) δ = -11.15 (d, *J* = 15.1 Hz, 2P), -20.91 – -21.66 (m, 8P) ppm. **^{31}P-NMR** (162 MHz, D_2O) δ = -10.78 – -11.45 (m, 2P), -20.80 – -21.85 (m, 8P) ppm. **^{13}C-NMR** (101 MHz, $CDCl_3$) δ = 168.5, 162.8, 154.9, 151.8, 148.8 (d, *J* = 9.3 Hz), 132.7, 127.8, 122.9, 112.0, 110.9, 107.6, 96.5, 80.0, 71.9, 59.7 (d, *J* = 3.9 Hz), 44.9, 29.0, 12.1 ppm. **HRMS CE-ESI** calc. for $C_{40}H_{50}N_4O_{37}P_{10}$ $[M-2H]^{2-}$: 743.9771, found: 743.9773.

1.6.4 Bis-DEAC450-polyP10 photolysis intermediate 25

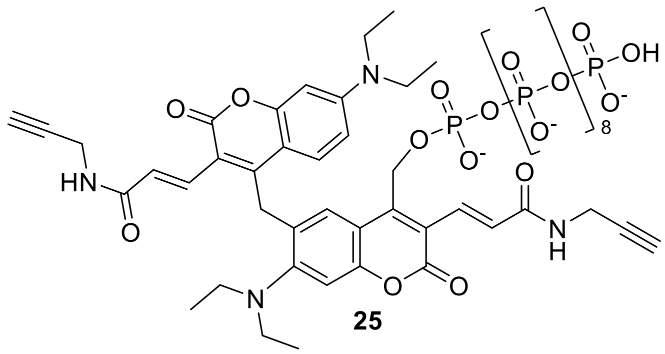

25

Bis-DEAC450 caged polyP10 **24** (3 × 1.93 mg, 3 × 1.1 µmol, 3 × 100 µM in H_2O) was irradiated, under stirring, with a 490 nM LED for 90 min. The solution was directly injected to strong anion exchange chromatography system for purification (Q Sepharose® Fast Flow, increasing concentrations of 1 M $NaClO_4$ in H_2O). Fractions eluted with 32-42% aqueous $NaClO_4$ were combined and concentrated by lyophilization. Precipitation in cold $NaClO_4$ acetone solution (0.5 M, 35 mL), washing with cold acetone (2 × 10 mL) and drying afforded **25** (2.0 mg, 1.2 µmol, 36%) as an orange solid.

^{1}H-NMR (700 MHz, D_2O) δ = 7.96 (s, 1H), 7.83 (d, *J* = 9.4 Hz, 1H), 7.66 (d, *J* = 15.9 Hz, 1H), 7.35 (d, *J* = 15.6 Hz, 1H), 7.15 (s, 1H), 6.94 (d, *J* = 15.9 Hz, 1H), 6.85 (d, *J* = 9.3 Hz, 1H), 6.70 (s, 1H), 6.53 (d, *J* = 15.8 Hz, 1H), 5.28 (d, *J* = 5.5 Hz, 2H), 4.61 (s, 2H), 4.15 (s, 2H), 3.93 (s, 2H), 3.52 (q, *J* = 7.1 Hz, 4H), 3.02 (q, *J* = 7.1 Hz, 4H), 2.67 (s, 1H)*, 2.60 (s, 1H)*, 1.23 (t, *J* = 7.1 Hz, 6H), 0.86 (t, *J* = 6.9 Hz, 6H) ppm. **^{31}P{^{1}H}-NMR** (283 MHz, D_2O) δ = -4.51 – -5.45 (m, 1P), -11.52 (d, *J* = 16.7 Hz, 1P), -19.94 – -20.45 (m, 1P), -20.87 – -22.04 (m, 7P) ppm. **HRMS CE-ESI** calc. for $C_{40}H_{50}N_4O_{37}P_{10}$ $[M-2H]^{2-}$: 743.9771, found: 743.9765.

*Not visible after long time in D_2O due to H/D exchange of acidic alkyne-protons.

1.6.5 (4-Azidobutyl) triphenyl phosphonium bromide (40)

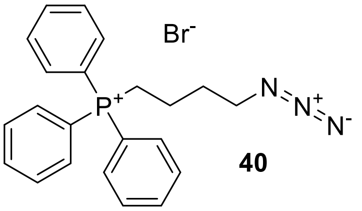

40

Compound **40** was synthesized as previously described[9] and recrystallized at r.t. with a mixture of EtOAc and DCM (2:1). Analytical data were consistent with the values reported in literature.[9]

1.6.6 Bis-TPP-DEAC$_{450}$ photocaged polyP$_{10}$ 27

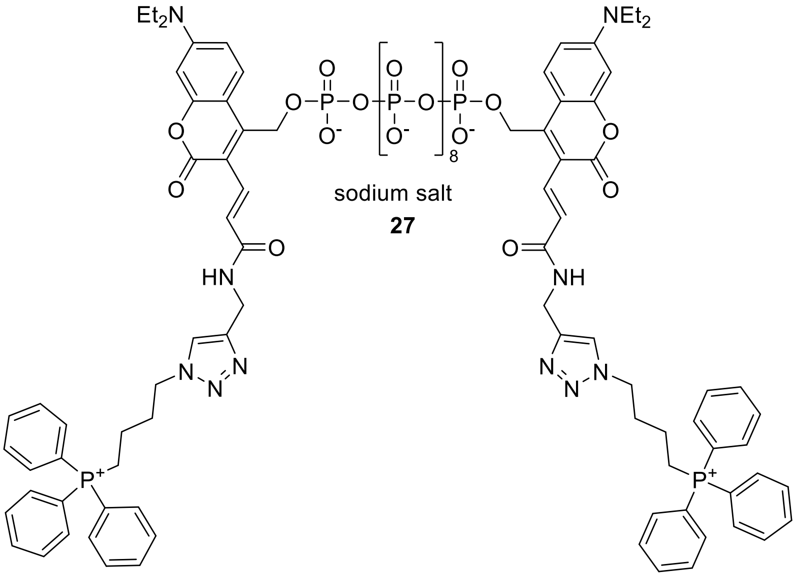

Bis-DEAC$_{450}$ caged polyP$_{10}$ **24** (10.0 mg, 5.85 µmol, 1.0 eq.) was dissolved in 100 mM triethylammonium acetate buffer (pH 7, 2.25 mL). The solution was degassed with an argon stream for 10 min. (4-Azidobutyl) triphenyl phosphonium bromide (**40**) (5.18 mg, 11.8 µmol, 2.0 eq.) was dissolved in DMSO (0.25 mL) and was added to the mixture. Subsequently, 20 mM $CuSO_4$ • 5 H_2O solution (292 µL, 5.84 µmol, 1.0 eq.) and 50 mM THPTA solution (585 µL, 29.3 µmol, 5.0 eq.) were premixed and added. Then sodium ascorbate (11.6 mg, 58.6 µmol, 10 eq.) was added and the reaction mixture was stirred for 3 h at r.t. The mixture was directly injected to RP-MPLC for purification (increasing concentration of MeCN in H_2O with 20% 100 mM TEAA buffer). The product containing fractions were combined and lyophilized. The TEA salt of **27** was dissolved in H_2O (3 mL) and chelex (each 20-40 mg) was added. After stirring gently for 15-20 min, the mixture was filtered through a syringe filter. Addition of chelex, stirring and filtration were repeated two times before the sample was lyophilized. The sodium salt of product **27** (4.4 mg, 1.9 µmol, 33%) was isolated as an orange solid.

^{1}H-NMR (400 MHz, D_2O) δ = 7.88 (s, 2H), 7.76 – 7.59 (m, 8H), 7.53 – 7.35 (m, 26H), 6.92 (d, *J* = 15.6 Hz, 2H), 6.77 – 6.66 (m, 2H), 5.96 (s, 2H), 5.04 (s, 4H), 4.52 (s, 4H), 4.45 – 4.32 (m, 4H), 3.31 – 3.15 (m, 8H), 3.15 – 3.00 (m, 4H), 2.06 – 1.95 (m, 4H), 1.43 – 1.31 (m, 4H), 1.12 – 0.93 (m, 12H) ppm. **^{31}P{^{1}H}-NMR** (162 MHz, D_2O) δ = 23.05 (s, 2P), -11.88 – -11.73 (m, 2P), -20.79 - -21.66 (m, 8P) ppm. **HRMS CE-ESI** calc. for $C_{84}H_{96}N_{10}O_{37}P_{12}$ [M-2H]$^{2-}$: 1103.1322, found: 1103.1319.

2. Absorption and Fluorescence Spectra

Absorption spectra were recorded on a Shimadzu UV-1900i UV-Vis spectrometer. Fluorescence spectra were recorded on a JASCO Spectrofluorometer FP-8300. Quarz cuvettes (Ø = 10 mm) were used.

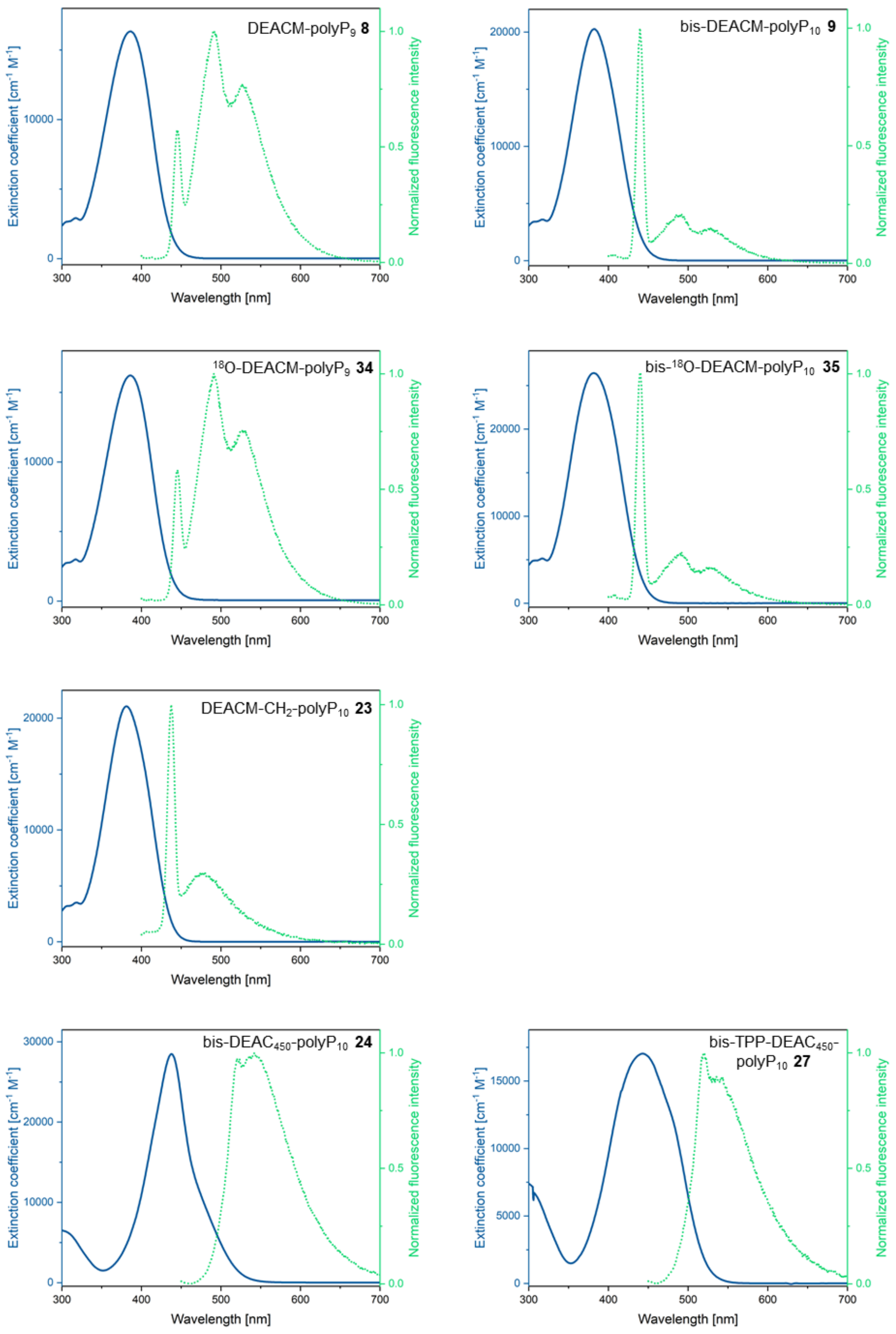

Figure ESI-1: UV/vis* (solid line) and fluorescence emission** (dashed line) spectra of the photocaged polyP$_{9/10}$'s **8**, **9**, **34**, **35**, **24**, **27** and fluorescently modified polyP$_{10}$ **23**. *50 µM in H_2O, **100 nM in H_2O.

3. Photolysis

Samples were irradiated with Mightex High-Power LED Collimator Sources (400 nm, 45 mm aperture, 3W, typical output power 265 (mW)[3]; 490 nm, 22 mm aperture, 1W, typical output power 140 (mW)[3]).

<u>DEACM-polyP9 **8**:</u>

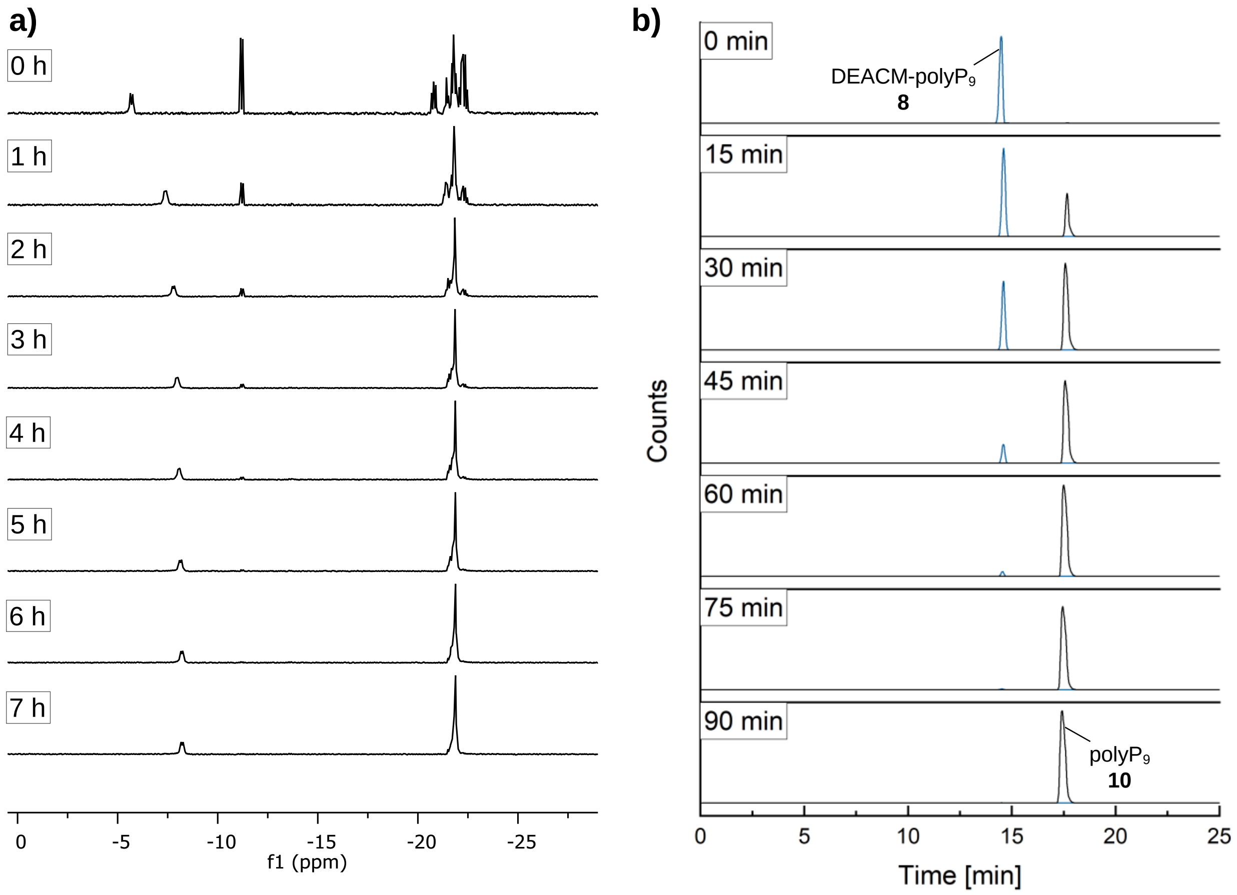

Figure ESI-2: a) ^{31}P-NMR reaction monitoring of uncaging of mono-DEACM caged polyP$_9$ **8** (10 mM in H_2O) at 400 nm. b) CE-MS analysis of uncaging of mono-DEACM caged polyP$_9$ **8** (2 mM in H_2O) at 400 nm (diluted to 500 µM prior to CE-MS measurement).

Bis-DEACM-polyP10 **9**:

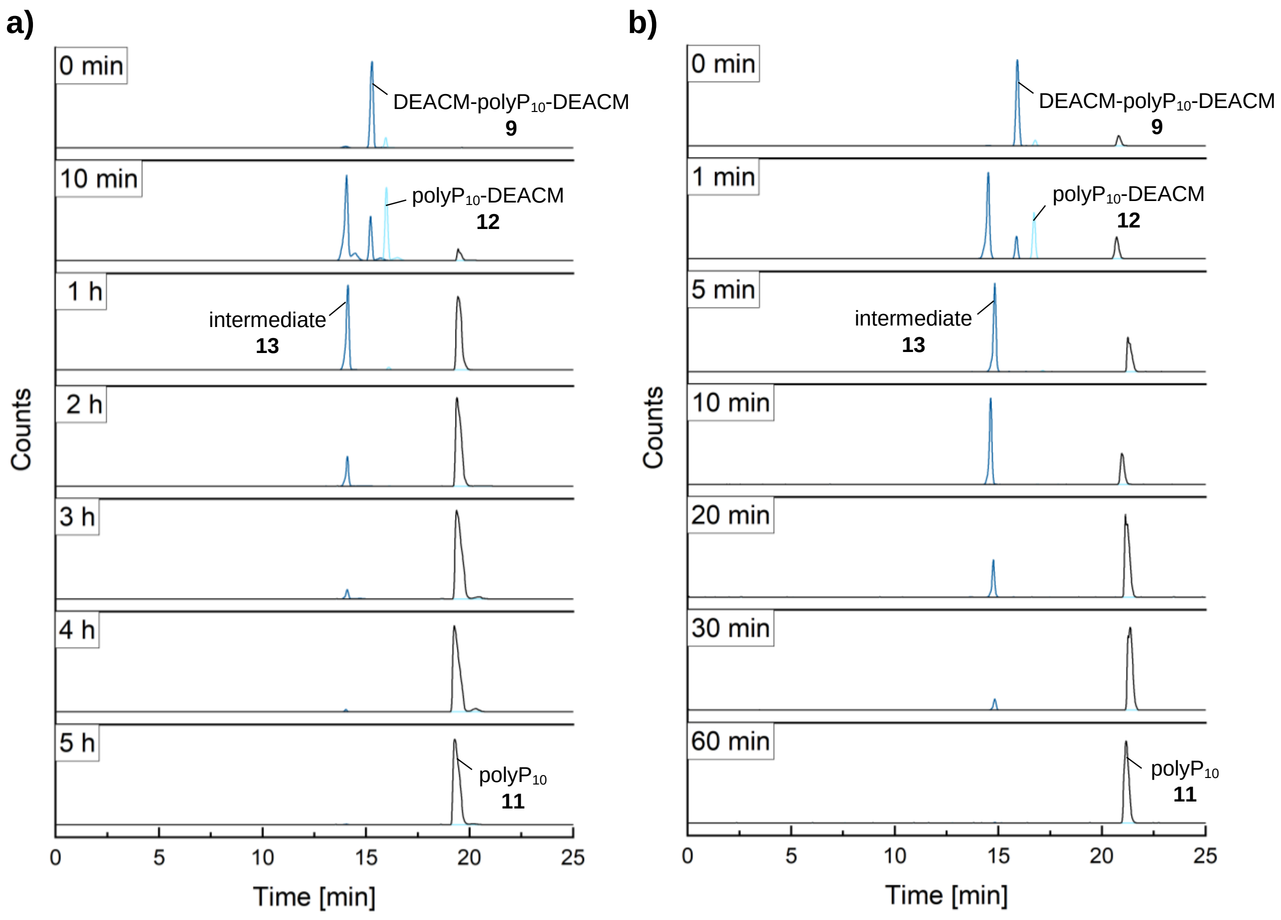

Figure ESI-3: CE-MS analysis of uncaging of bis-DEACM caged polyP10 **9** at 400 nm. a) 2 mM in H_2O (diluted to 500 µM prior to CE-MS measurement), b) 100 µM in H_2O.

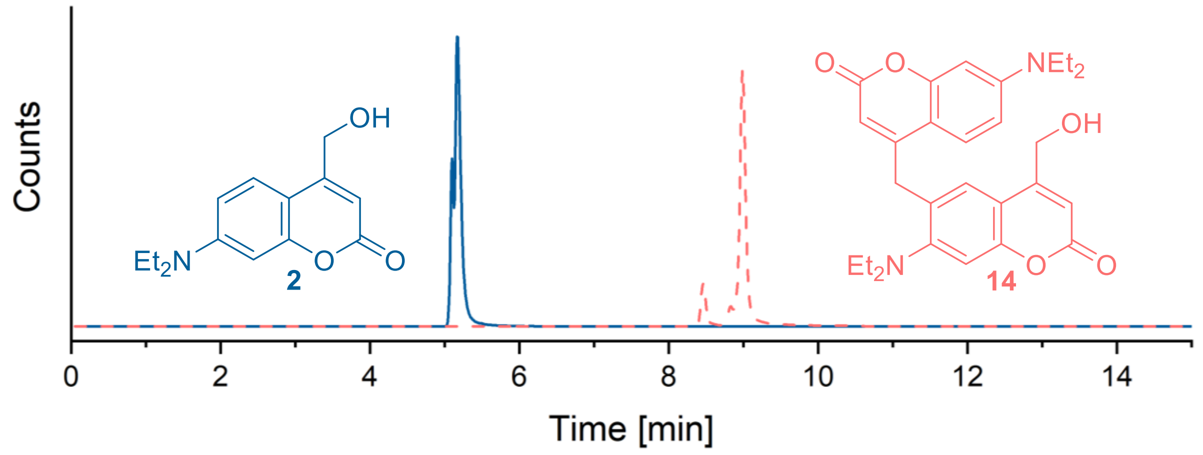

Figure ESI-4: LC-HRMS analysis of the fully deprotected reaction mixture of **9** (2 mM in H_2O, irradiated at 400 nm for 8 h), lyophilized post-irradiation and re-dissolved in DCM, shows that the masses corresponding to **2** and **14** can be found.

Bis-DEACM-CH_2-polyP$_{10}$ **23**:

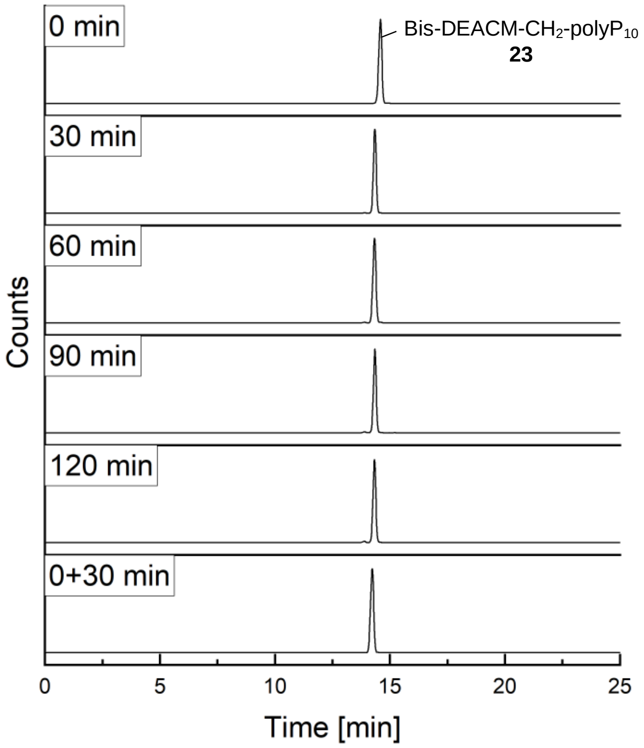

Figure ESI-5: CE-MS analysis of irradiating bis-DEACM-polyP$_{10}$ **23** (100 µM in H_2O) with 400 nm.

Bis-DEAC450-polyP10 **24**:

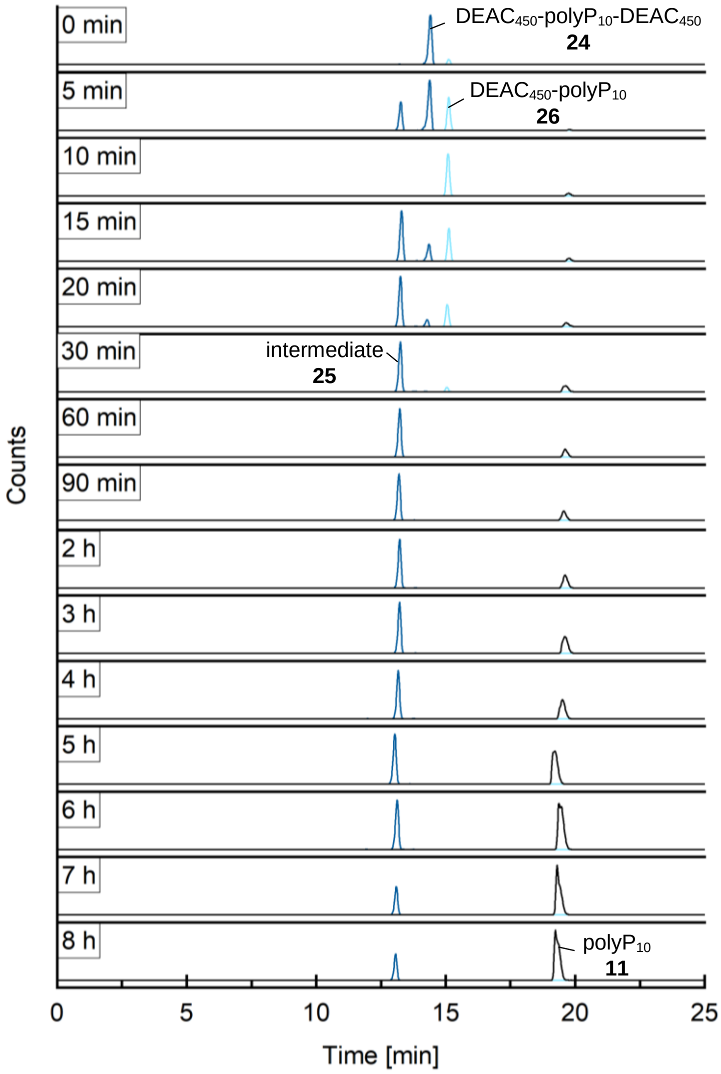

Figure ESI-6: CE-MS analysis of uncaging of bis-DEAC450-polyP10 **24** (100 µM in H_2O) at 490 nm.

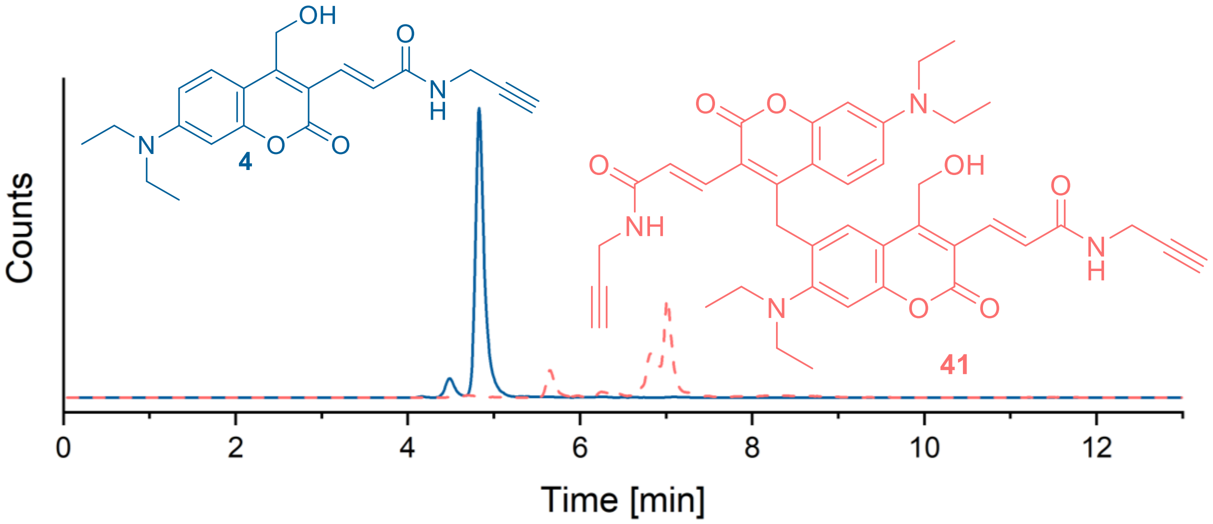

Figure ESI-7: LC-HRMS analysis of the fully deprotected reaction mixture of **24** (100 µM in H_2O, irradiated at 490 nm for 8 h), lyophilized post-irradiation and re-dissolved in DCM, shows that the masses corresponding to **4** and **41** can be found.

Bis-TPP-DEAC450-polyP10 **27**:

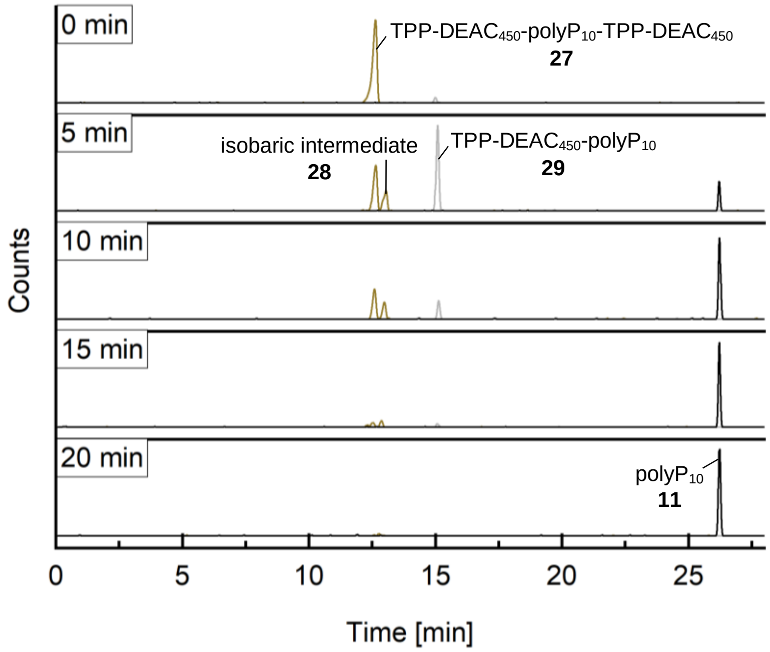

Figure ESI-8: CE-MS analysis of uncaging of TPP-DEAC450-polyP10 **27** (100 µM in H_2O) at 490 nm.

4. DFT-Studies

General remarks

All DFT calculations have been carried out using Gaussian16.[10] All geometries were optimized in the gas phase using the BP86[11] functional in combination with the def2SVP basis set[12] for all atoms. In all cases the ultrafine integral grid was employed (int=grid=ultrafine), moreover, we used the scf=tight keyword for all structures. During the geometry optimization we considered the solvent water by the use of the SMD model[13] and the dispersion energies were considered by the use of the Grimme D3 correction together with Becke-Johnson damping (keyword EmpiricalDispersion=GD3BJ).[14] Frequency calculations were performed in order to obtain thermal corrections at 298 K. All optimized species showed no imaginary frequencies during vibrational analysis. The polyP-chains in all calculated structures were stabilized by being partly protonated and coordinating sodium ions.

Atomic coordinates

DEACM cation 15

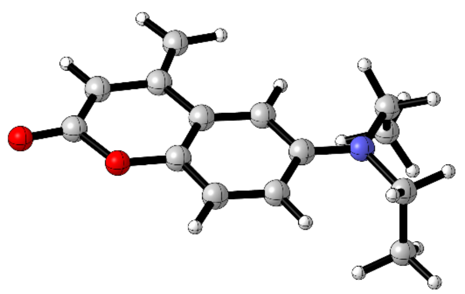

E/hartree	-747.968529352
E+zvp/hartree	-747.692069
G/hartree	-747.735581

C	-2.7288685	2.5552418	0.1053537
C	-2.9432935	1.1888808	0.0799837
C	-4.2786555	0.7333818	-0.0538283
C	-4.6348505	-0.6343932	-0.0923073
O	-3.5308375	-1.5455902	0.0060797
C	-2.2268195	-1.1730742	0.1363697
C	-1.8543745	0.2025038	0.1861447
C	-0.5104885	0.5106968	0.3238567
C	0.4817835	-0.5090302	0.4118137
N	1.7985035	-0.1989332	0.5669597
C	2.8540765	-1.2412042	0.5059127
C	3.1463305	-1.7140092	-0.9235243
C	2.2579975	1.2083108	0.6431857
C	2.4207535	1.8636688	-0.7342683
C	0.0564645	-1.8839282	0.3423177
C	-1.2724085	-2.2026652	0.2119307
O	-5.7666005	-1.1529202	-0.2002603

H	-3.5683355	3.2368288	0.0248067
H	-1.7428145	2.9924728	0.2058167
H	-5.0828735	1.4532208	-0.1328653
H	-0.2133575	1.5469378	0.3518107
H	3.7497665	-0.8032422	0.9465267
H	2.5622065	-2.0808572	1.1412917
H	3.5082465	-0.8888212	-1.5428223
H	2.2552255	-2.1383572	-1.3964973
H	3.9199835	-2.4882992	-0.8913813
H	3.2139555	1.1970958	1.1680817
H	1.5591975	1.7730408	1.2638497
H	2.7219895	2.9076378	-0.5984983
H	1.4832395	1.8450048	-1.2986353
H	3.1906205	1.3561108	-1.3217193
H	0.7782765	-2.6843702	0.3988137
H	-1.6040395	-3.2313392	0.1657007

DEACM-polyP$_{10}$ anion 16

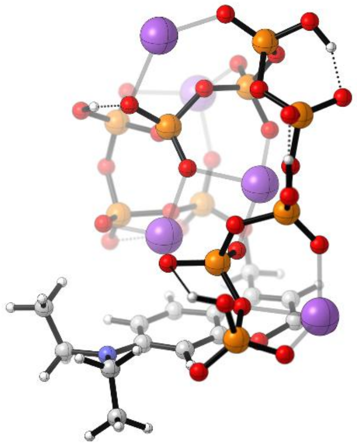

E/hartree	-7307.214165
E+zvp/hartree	-7306.749700
G/hartree	-7306.855424

O	3.3421144	-2.7066170	2.4416075
P	1.8523054	-2.8848650	2.3268765
O	1.2657914	-2.4989760	0.8290295
P	1.0487274	-3.6118270	-0.4249975
O	2.6338144	-4.0433910	-0.7807045
P	3.9088864	-3.1076820	-1.1560145
O	3.4958294	-1.6656910	-0.4974195
P	3.3382464	-0.1700120	-1.2100115
O	4.6219894	0.5848100	-0.5122035
P	5.0101684	0.6986530	1.1345135
O	5.8240574	2.1531940	1.0017335
P	5.7720374	3.2020110	-0.2485345

O	4.1635714	3.4541340	-0.5302735
P	3.3061524	4.7294920	0.1338475
O	1.9045954	3.9173260	0.5663215
P	0.3447824	4.4032250	0.1719815
O	-0.1226696	3.1984430	-0.8129595
P	-1.0737006	1.8584800	-0.3744865
O	-2.4291096	2.5699090	0.1564055
P	-3.6686266	3.4780870	-0.6199835
O	-4.6729286	2.5110490	-1.2131335
O	-4.0159216	4.4701400	0.4870465
O	-0.3974376	1.1927710	0.8299765
O	-1.2434316	1.0438550	-1.6463505

O	0.4880504	5.6690790	-0.7485975
O	-0.4671976	4.5324710	1.4322805
O	2.9392044	5.6985290	-0.9926015
O	4.0589704	5.1869150	1.3749705
O	6.3478524	4.5423010	0.3756165
O	6.4584544	2.7111730	-1.4929265
O	6.0102324	-0.3902950	1.4621285
O	3.7512384	0.9017500	1.9541205
O	2.0152684	0.4206400	-0.7715385
O	3.7482054	-0.2968050	-2.6861725
O	5.1480044	-3.6511800	-0.4973655
O	3.9273444	-2.8926180	-2.7295605
O	0.4567604	-2.7919080	-1.5498805
O	0.4356784	-4.8640780	0.1876945
O	1.2771294	-4.3525250	2.5971055
O	1.1120924	-1.8538060	3.3145795
C	-0.2133546	-2.0841150	3.8955015
C	-1.2056296	-1.1042230	3.3194305
C	-1.3219926	0.1448070	3.9106665
C	-2.1981456	1.1466580	3.3763585
O	-3.0013446	0.7803880	2.3062885
C	-2.8151006	-0.3916720	1.6116415
C	-1.9554646	-1.4054090	2.1355765
C	-1.8719236	-2.6075930	1.3749765
C	-2.5181516	-2.7517930	0.1566145
N	-3.8803286	-1.7871700	-1.6347405
C	-3.7934586	-3.0125730	-2.4381585
C	-2.5371206	-3.0867190	-3.3079785
C	-4.5074176	-0.6274600	-2.2812025
C	-5.9984336	-0.4809950	-1.9739485
C	-3.3146036	-1.6850550	-0.3963565
C	-3.4809616	-0.5080690	0.3963065
O	-2.3283716	2.3186780	3.7702635
H	-0.0890926	-1.9107950	4.9800925
H	-0.5132536	-3.1360200	3.7325045
H	-0.7230696	0.4218250	4.7894055
H	-1.2491166	-3.4378870	1.7343425
H	-3.8607756	-3.8928720	-1.7684765
H	-4.7024936	-3.0423060	-3.0732955
H	-1.6163136	-3.1280080	-2.6922795
H	-2.4667276	-2.2056010	-3.9793355
H	-2.5688376	-3.9966100	-3.9422065
H	-4.3536606	-0.7430090	-3.3735705
H	-3.9609346	0.2907210	-1.9886105
H	-6.4058306	0.4070490	-2.4983805
H	-6.1759866	-0.3454060	-0.8873575
H	-6.5674446	-1.3728750	-2.3090365
H	-2.3756306	-3.6852770	-0.4013815
H	-4.0893166	0.3395170	0.0584105
H	1.5307964	5.8085900	-0.9898905
H	0.8675274	-4.7344500	1.7245045
H	5.5935794	4.9564290	0.9520705
H	3.8628014	-1.8799990	-2.9330815
H	-2.7269286	3.7406160	-2.5774925
O	-2.8222996	4.2892480	-1.7677805
Na	1.7646404	1.5299520	1.2678505
Na	5.3637244	-2.5340000	1.5241315
Na	0.0143734	-0.7499470	-0.6072045
Na	5.8125794	0.8768630	-2.6226585
Na	-2.5170356	3.9304020	2.1453625

<u>Wheland complex **17**</u>

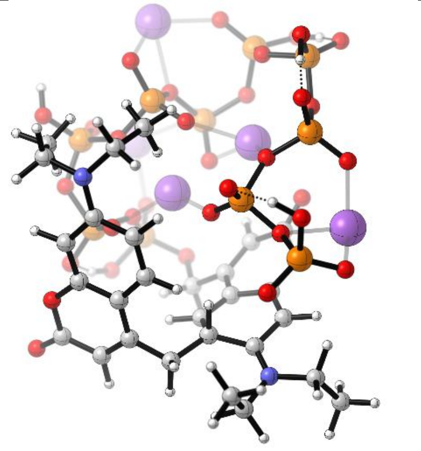

E/hartree	-8054.93112975
E+zvp/hartree	-8054.196798
G/hartree	-8054.318565

O	3.6785484	3.1743702	-3.9296686
P	2.2690544	3.2168912	-3.4092166
O	2.1480814	2.8115552	-1.8154416
P	1.4636734	3.7041642	-0.5499236
O	2.8815414	4.1857372	0.2171084
P	4.2256524	3.3133912	0.4500314
O	3.5807524	1.8375882	0.7592484
P	4.3405354	0.3568622	1.0214354
O	5.3093044	0.2758762	-0.3509596
P	4.7947434	-0.1773388	-1.8712186
O	5.1407554	-1.8406838	-1.8247726
P	6.1801174	-2.7222618	-0.9177296
O	5.1124424	-3.3484778	0.1950444
P	4.4341854	-4.8669778	0.1970664
O	2.9163784	-4.5258738	-0.3253406
P	1.4644124	-4.6579428	0.5784224
O	1.1573804	-3.0419898	0.5035054
P	-0.2235346	-2.1180788	0.8640524
O	-1.3317146	-2.9073108	-0.0479316
P	-2.7725026	-3.6264278	0.5738614
C	-3.2974916	2.6405922	0.1493854
C	-3.2477316	3.9156962	-0.3859386
C	-2.4301146	4.9634202	0.1716764
O	-1.6991626	4.6381712	1.3212864
C	-1.6816516	3.3677382	1.8527014
C	-2.4829566	2.3280192	1.2954484
C	-2.3511376	1.0487422	1.9098734
C	-1.4396646	0.8122802	2.9282474
N	0.3282234	1.6254282	4.4279444
C	0.7160994	0.2505962	4.7729424
C	1.6950354	-0.3851138	3.7810694
C	1.2672314	2.6884832	4.8110374
C	2.3485434	3.0053652	3.7712114
C	-0.6119906	1.8641842	3.4513364
C	-0.8115136	3.1692952	2.9213574
O	-2.3126226	6.1173212	-0.2419006

O	-3.7494926	-2.5158878	0.9143634
O	-3.0611336	-4.6669768	-0.5135916
O	0.0804344	-0.7785388	0.2183614
O	-0.5629816	-2.3074908	2.3377744
O	1.8852864	-5.0802508	1.9936044
O	0.4745944	-5.4568018	-0.2330336
O	4.3154994	-5.2966718	1.6929494
O	5.1945624	-5.7399428	-0.7787656
O	6.6219754	-3.9238158	-1.8396526
O	7.2598904	-1.9239678	-0.2478566
O	5.6869484	0.5141772	-2.8726576
O	3.2718734	-0.1381448	-1.9287856
O	3.2140314	-0.6550528	0.9167464
O	5.3457304	0.4507212	2.1496964
O	5.2330794	3.3186782	-0.6653766
O	4.7214844	3.9237852	1.8420774
O	0.7408904	2.6837282	0.2923494
O	0.8221554	4.9581032	-1.1246046
O	1.4935894	4.5984132	-3.5530926
O	1.4012154	2.0737262	-4.1606636
C	0.0326044	1.8485552	-3.7817376
C	-0.1781156	0.4383522	-3.3118086
C	0.7466744	-0.5516288	-3.5137526
C	0.4856194	-1.9097708	-3.0759236
O	-0.8560266	-2.2699108	-2.8761716
C	-1.7914656	-1.3134918	-2.5841186
C	-1.4191906	0.0901562	-2.6316886
C	-2.2353426	0.9549602	-1.9561626
C	-3.3816926	0.4513472	-1.1355656
N	-5.2686166	-1.2302368	-1.5236336
C	-6.3776626	-0.4275658	-0.9684236
C	-6.5929346	-0.6811398	0.5229754
C	-5.6703996	-2.6248758	-1.8507926
C	-5.9878756	-2.8119718	-3.3319096
C	-4.0116266	-0.8269858	-1.6848646
C	-3.0520906	-1.7379308	-2.2500966
O	1.3080724	-2.7970078	-2.8661396
H	-3.8374486	4.1808112	-1.2750286
H	-2.9636186	0.1988502	1.5708564
H	-0.1976166	-0.3694328	4.8576564
H	1.1586364	0.2828732	5.7889644
H	1.3216484	-0.2851028	2.7442304
H	2.6964754	0.0856842	3.8297664
H	1.8148104	-1.4671508	3.9922624
H	1.7366304	2.3730422	5.7640114
H	0.6865344	3.6049962	5.0453954
H	1.9086534	3.2447212	2.7830164
H	2.9367724	3.8855482	4.1008994
H	3.0511084	2.1614592	3.6308004
H	-1.3368006	-0.2143848	3.2970264
H	-0.2241816	4.0321972	3.2550754
H	-0.5993346	2.0077222	-4.6828396
H	-0.3120976	2.5853812	-3.0279996
H	1.7427744	-0.3422368	-3.9227096
H	-1.9841796	2.0229542	-1.8766136
H	-6.2062366	0.6365012	-1.1909386
H	-7.2783866	-0.7231018	-1.5422596
H	-5.7046596	-0.3935468	1.1197914
H	-6.8046956	-1.7495958	0.7270614
H	-7.4596846	-0.0836248	0.8694794
H	-6.5666426	-2.8354618	-1.2382356
H	-4.8863106	-3.3203908	-1.4962506
H	-6.3225836	-3.8550818	-3.5006346
H	-5.1003176	-2.6262428	-3.9698486
H	-6.8014686	-2.1319178	-3.6563186
H	-2.8223326	-0.0363498	-0.2848646
H	-3.2926756	-2.7985898	-2.3777126
H	3.2476564	-5.2345248	1.9884784
H	1.1412464	4.9141112	-2.6220056
H	6.1127444	-4.7935948	-1.5553866
H	5.6925354	3.8171702	1.9671884
H	-1.5947836	-3.7027848	2.3628554
O	-2.1837876	-4.3807738	1.8993214
C	-4.1922526	1.6028272	-0.4951516
H	-4.8491446	1.1927552	0.2927074
H	-4.8179606	2.1006852	-1.2590526
Na	2.7251414	-2.1453178	-0.8942636
Na	5.5530874	2.7650282	-2.8081886
Na	1.6121854	0.6844632	-0.3646816
Na	7.3084614	-0.0315058	0.9532994
Na	-1.0063706	-4.5910008	-1.6744436

TS-intramolecular deprotonation

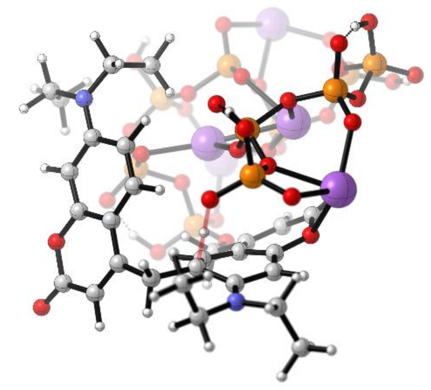

E/hartree	-8054.92669546
E+zvp/hartree	-8054.196887
G/hartree	-8054.317389
Imaginary frequency	-1138.06 cm^{-1}

O	3.5446736	2.7283825	-4.1221109
P	2.2611486	2.7545215	-3.3383509
O	2.5617416	2.6639175	-1.7170969
P	2.0107006	3.7041765	-0.4950469
O	3.5009226	4.1646775	0.1183201
P	4.8179506	3.2561525	0.3621131
O	4.1508666	1.8118175	0.7526381
P	4.8979776	0.3291635	1.0681971
O	5.6655286	0.0645705	-0.4033969
P	4.9633396	-0.3754355	-1.8513349
O	5.4758556	-1.9795705	-1.9632039
P	6.2498626	-2.9711245	-0.9375779
O	4.9908216	-3.4428485	0.0584711
P	4.4140956	-4.9924745	0.2962191
O	2.8187556	-4.7108975	0.0894871
P	1.6018106	-4.7451135	1.2990731
O	1.4156026	-3.1131625	1.3627021
P	0.2048046	-1.9408395	1.3033721
O	-1.0281874	-2.7634245	0.5852871
P	-2.5587644	-2.9941645	1.2995251
C	-3.8325934	2.2160025	-0.2698579
C	-4.2108884	3.3261355	-1.0063739
C	-3.6603884	4.6396685	-0.7900079
O	-2.7104794	4.7594915	0.2326401

C	-2.2883884	3.6778475	0.9783721
C	-2.8254944	2.3735725	0.7517021
C	-2.2710524	1.3331875	1.5501561
C	-1.2694674	1.5677335	2.4803751
N	0.2097336	3.1189265	3.6597431
C	0.9229136	2.0001245	4.2939611
C	1.9383226	1.2851565	3.3975911
C	0.7225756	4.4720625	3.9083381
C	1.7839266	4.9776365	2.9257351
C	-0.7609674	2.8897515	2.7174281
C	-1.3153434	3.9461285	1.9394721
O	-3.9343534	5.6727135	-1.4029389
O	-3.3113344	-1.6464825	1.1900761
O	-3.0696444	-4.2401055	0.5933531
O	0.7602476	-0.9562995	0.2866611
O	-0.1898194	-1.5666295	2.7242181
O	2.3001456	-5.2145775	2.5812651
O	0.4051496	-5.4608715	0.7283921
O	4.6285316	-5.3475145	1.8040921
O	5.0102166	-5.8975745	-0.7603409
O	6.6126726	-4.2337145	-1.8121539
O	7.3600576	-2.3307995	-0.1541389
O	5.6366806	0.3962375	-2.9594309
O	3.4504366	-0.4221215	-1.6678919
O	3.7230816	-0.6134805	1.2472741
O	6.0583976	0.4904175	2.0261521
O	5.7774426	3.1816085	-0.7943909
O	5.3527616	3.9081895	1.7196841
O	1.3006086	2.7977485	0.4797311
O	1.3924776	4.9340185	-1.1405399
O	1.2939016	4.0044815	-3.5199099
O	1.4022096	1.4216145	-3.6486809
C	0.0876666	1.2359575	-3.0888659
C	-0.1473304	-0.2140695	-2.7772189
C	0.7918516	-1.1855245	-3.0067409
C	0.5383746	-2.5565275	-2.6183639
O	-0.8116114	-2.9439935	-2.4794879
C	-1.7881274	-1.9976865	-2.2789989
C	-1.4298234	-0.6091245	-2.2255439
C	-2.3623604	0.2274865	-1.6474179
C	-3.6162844	-0.2448135	-1.0797379
N	-5.3493234	-2.0679175	-1.4387579
C	-6.5332054	-1.3355105	-0.9457469
C	-6.7607604	-1.5178625	0.5543321
C	-5.6252614	-3.5041925	-1.6897939
C	-5.9156374	-3.8112615	-3.1568879
C	-4.1133584	-1.5612595	-1.6073359

C	-3.0817874	-2.4369295	-2.0975489
O	1.3686976	-3.4312505	-2.3929389
H	-4.9653914	3.2480025	-1.8023829
H	-2.6317314	0.3023705	1.4421281
H	0.1827466	1.2686835	4.6815941
H	1.4354856	2.4168425	5.1828951
H	1.4407976	0.8030885	2.5343101
H	2.7102746	1.9811935	3.0163561
H	2.4458276	0.4833885	3.9713871
H	1.1423106	4.4656775	4.9343181
H	-0.1358934	5.1755775	3.9331591
H	1.3679946	5.1246575	1.9099431
H	2.1661796	5.9597415	3.2734021
H	2.6435486	4.2828575	2.8560671
H	-0.8690594	0.7066075	3.0282041
H	-0.9601794	4.9800475	2.0242411
H	-0.6666854	1.5754915	-3.8306949
H	-0.0715784	1.8550455	-2.1781749
H	1.8052346	-0.9430075	-3.3494999
H	-2.1173704	1.2920265	-1.5283339
H	-6.4530814	-0.2747475	-1.2212449
H	-7.3979334	-1.7308495	-1.5159399
H	-5.8891704	-1.1670735	1.1411491
H	-6.9328684	-2.5829415	0.8119031
H	-7.6555454	-0.9417125	0.8648671
H	-6.5036884	-3.7650865	-1.0694509
H	-4.7875604	-4.1065385	-1.2911349
H	-6.1536354	-4.8884755	-3.2679329
H	-5.0488574	-3.5809535	-3.8089749
H	-6.7864224	-3.2278135	-3.5197309
H	-3.2844264	-0.8746045	-0.0207439
H	-3.2921484	-3.4919305	-2.3032039
H	3.6469486	-5.3152305	2.3082171
H	1.2380576	4.5430415	-2.6284939
H	6.0146166	-5.0440145	-1.5303259
H	6.3048226	3.7138185	1.8821641
H	-1.4826804	-2.6201705	3.1104001
O	-2.1987114	-3.2900545	2.8476291
C	-4.5305154	0.9008815	-0.5759089
H	-5.0640014	0.5720115	0.3380011
H	-5.2906934	1.1316225	-1.3438279
Na	2.8108936	-2.1556645	-0.3152489
Na	5.4980546	2.6266925	-2.9754399
Na	2.0837626	0.7307125	-0.0818659
Na	7.7895526	-0.2936815	0.6755531
Na	-1.1607844	-4.7996605	-0.6893169

DEACM-polyP$_{10}$ photolysis intermediate **13**

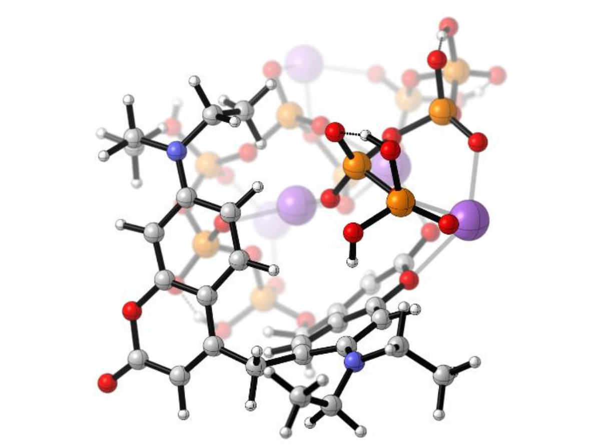

E/hartree	-8054.96570723
E+zvp/hartree	-8054.230923
G/hartree	-8054.350660

O	2.8566448	3.8239459	-3.7565309
P	1.5139578	3.5202039	-3.1517609
O	1.6183388	3.0735899	-1.5662479
P	0.7950728	3.7149809	-0.2317529
O	2.0774798	4.5469139	0.4738041
P	3.6030528	4.0363639	0.6430341

O	3.3803168	2.4279749	0.8687961
P	4.5234098	1.2004409	1.0405111
O	5.4126978	1.4372839	-0.3668189
P	4.9940448	0.9113729	-1.8909309
O	5.7741278	-0.5958771	-1.9146339
P	7.0578038	-1.1571761	-1.0696199
O	6.2750848	-2.0440051	0.0999821
P	6.0028868	-3.6802611	0.1060421
O	4.4069618	-3.7373281	-0.2785809
P	3.1237848	-4.2692471	0.7248231
O	2.4105698	-2.7707871	0.7628481
P	0.8747878	-2.2135781	1.1167601
O	-0.0060102	-3.3972041	0.3214061
P	-1.3003842	-4.1875301	0.9954181
C	-4.2927302	1.7975219	-0.2183619
C	-4.7773042	3.0253889	-0.6309069
C	-4.3413872	4.2709509	-0.0517229
O	-3.4223712	4.1833739	1.0025901
C	-2.9072822	2.9790429	1.4364011
C	-3.2958562	1.7527099	0.8235621
C	-2.6501342	0.5816929	1.3132851
C	-1.7005262	0.6273909	2.3252941
N	-0.4232982	1.9335479	3.9748931
C	0.4254088	0.7834259	4.3127721
C	1.6539598	0.6215149	3.4139641
C	-0.0195722	3.2306849	4.5352971
C	0.9256708	4.0648089	3.6634951
C	-1.3460322	1.8704779	2.9574151
C	-2.0008212	3.0461169	2.4913271
O	-4.6945222	5.4069449	-0.3724389
O	-2.4169572	-3.0286821	1.1469471
O	-1.6117412	-5.3573451	0.1037631
O	0.7309578	-0.9310101	0.3284081
O	0.5729208	-2.3530581	2.6034741
O	3.7481808	-4.6068211	2.0801901
O	2.2878588	-5.2468281	-0.0629319
O	6.1299148	-4.1400301	1.5954451
O	6.8722948	-4.3280071	-0.9496419
O	7.7579888	-2.1987751	-2.0262309
O	7.9175518	-0.0925841	-0.4538159
O	5.6304648	1.8585259	-2.8789669
O	3.5158358	0.5401429	-1.9189139
O	3.7062578	-0.0725151	0.9313231
O	5.5199568	1.5295329	2.1310511
O	4.5389918	4.3663379	-0.4873339
O	3.9608888	4.6671489	2.0691471
O	0.4366858	2.5114549	0.6003861
O	-0.1956452	4.7604799	-0.7206309
O	0.4297978	4.6831509	-3.1884219
O	0.8887618	2.2128539	-3.8719099
C	-0.3989932	1.7098989	-3.4593119
C	-0.3144552	0.2842519	-2.9817529
C	0.8560198	-0.4550481	-3.0829359
C	0.8953238	-1.8179231	-2.6517489
O	-0.3176882	-2.4461341	-2.3943209
C	-1.4789852	-1.7323161	-2.2202349
C	-1.4927732	-0.3322281	-2.4449019
C	-2.6817412	0.3464979	-2.0677279
C	-3.8042622	-0.3029201	-1.5677689
N	-4.9879412	-2.4803691	-1.3303919
C	-6.3463102	-1.9713221	-1.5987389
C	-7.2610972	-1.9061851	-0.3773079
C	-4.9223652	-3.9465641	-1.1854909
C	-4.9270012	-4.7176841	-2.5083339
C	-3.8514682	-1.7589141	-1.5989889
C	-2.6184022	-2.4406911	-1.8440219
O	1.8960558	-2.5457661	-2.4747439
H	-5.5303392	3.0992239	-1.4286699
H	-2.8951972	-0.3918051	0.8671481
H	-0.1848902	-0.1405691	4.2835911
H	0.7364258	0.9113159	5.3695411
H	1.3491388	0.5202289	2.3562861
H	2.3461048	1.4823389	3.4960071
H	2.2112248	-0.2953301	3.6910741
H	0.4652838	3.0148189	5.5081441
H	-0.9336572	3.8153799	4.7715511

H	0.4530418	4.3627329	2.7085021
H	1.2058378	4.9924769	4.2038931
H	1.8549728	3.5137779	3.4259541
H	-1.2129492	-0.3095401	2.6225861
H	-1.7663702	4.0397629	2.8899741
H	-1.0709632	1.7539189	-4.3426399
H	-0.8576052	2.3584199	-2.6829229
H	1.7934018	-0.0043441	-3.4312819
H	-2.6801162	1.4467649	-2.0729599
H	-6.2714162	-0.9914891	-2.1000689
H	-6.7991352	-2.6502131	-2.3544589
H	-6.8854312	-1.2002401	0.3907141
H	-7.3753352	-2.9009841	0.0999391
H	-8.2701772	-1.5688311	-0.6903759
H	-5.8002372	-4.2437741	-0.5776839
H	-4.0357242	-4.2099781	-0.5783559
H	-4.9026952	-5.8067451	-2.3006059
H	-4.0485972	-4.4706881	-3.1381849
H	-5.8423492	-4.5063441	-3.0982239
H	-2.5386052	-2.5687831	0.2716741
H	-2.5517842	-3.5352321	-1.8258689
H	5.1346798	-4.3704111	1.9747801
H	0.0499538	4.8539799	-2.2298519
H	7.4944168	-3.1707321	-1.7460019
H	4.9344928	4.7097539	2.2180801
H	-0.3064452	-3.7192321	2.8115891
O	-0.8796612	-4.5142531	2.4964091
C	-4.8477942	0.5338069	-0.8407289
H	-5.2782392	-0.0826061	-0.0247999
H	-5.6942302	0.8163339	-1.4987929
Na	3.4805148	-1.5746481	-0.9407209
Na	4.8087108	3.9622779	-2.6832759
Na	1.7746478	0.8882129	-0.3251199
Na	7.4719598	1.6774239	0.8514661
Na	0.7024298	-4.6024091	-1.5433429

5. Supporting Literature

1 D. Qiu, M. S. Wilson, V. B. Eisenbeis, R. K. Harmel, E. Riemer, T. M. Haas, C. Wittwer, N. Jork, C. Gu, S. B. Shears, G. Schaaf, B. Kammerer, D. Fiedler, A. Saiardi and H. J. Jessen, *Nat. Commun.*, 2020, **11**, 6035.

2 A. Ripp, M. Krämer, V. Barth, P. Moser, T. M. Haas, J. Singh, T. Huck, L. Gleue, K. Friedland, M. Helm and H. J. Jessen, *Angew. Chemie Int. Ed.*, DOI:10.1002/anie.202414537.

3 L. Bialy and H. Waldmann, *Chem. - A Eur. J.*, 2004, **10**, 2759–2780.

4 A. Hofer, G. S. Cremosnik, A. C. Müller, R. Giambruno, C. Trefzer, G. Superti-Furga, K. L. Bennett and H. J. Jessen, *Chem. - A Eur. J.*, 2015, **21**, 10116–10122.

5 T. Weinrich, M. Gränz, C. Grünewald, T. F. Prisner and M. W. Göbel, *European J. Org. Chem.*, 2017, **2017**, 491–496.

6 J. Ma, A. Ripp, D. Wassy, T. Dürr, D. Qiu, M. Häner, T. Haas, C. Popp, D. Bezold, S. Richert, B. Esser and H. J. Jessen, *Molecules*, 2020, **25**, 5325.

7 R. H. Beddoe, D. C. Edwards, L. Goodman, H. F. Sneddon and R. M. Denton, *Chem. Commun.*, 2020, **56**, 6480–6483.

8 J. Ma, J. Wehrle, D. Frank, L. Lorenzen, C. Popp, W. Driever, R. Grosse and H. J. Jessen, *Chem. Sci.*, 2024, **15**, 6478–6487.

9 N. Wagner, M. Stephan, D. Höglinger and A. Nadler, *Angew. Chemie - Int. Ed.*, 2018, **57**, 13339–13343.

10 Gaussian 16, Revision C.01; M. J. Frisch, G. W. Trucks, H. B. Schlegel, G. E. Scuseria, M. A. Robb, J. R. Cheeseman, G. Scalmani, V. Barone, G. A. Petersson, H. Nakatsuji, X. Li, M. Caricato, A. V. Marenich, J. Bloino, B. G. Janesko, R. Gomperts, B. Mennucci, H. P. Hratchian, J. V. Ortiz, A. F. Izmaylov, J. L. Sonnenberg, D. Williams-Young, F. Ding, F. Lipparini, F. Egidi, J. Goings, B. Peng, A. Petrone, T. Henderson, D. Ranasinghe, V. G. Zakrzewski, J. Gao, N. Rega, G. Zheng, W. Liang, M. Hada, M. Ehara, K. Toyota, R. Fukuda, J. Hasegawa, M. Ishida, T. Nakajima, Y. Honda, O. Kitao, H. Nakai, T. Vreven, K. Throssell, J. A. Montgomery, Jr., J. E. Peralta, F. Ogliaro, M. J. Bearpark, J. J. Heyd, E. N. Brothers, K. N. Kudin, V. N. Staroverov, T. A. Keith, R. Kobayashi, J. Normand, K. Raghavachari, A. P. Rendell, J. C. Burant, S. S. Iyengar, J. Tomasi, M. Cossi, J. M. Millam, M. Klene, C. Adamo, R. Cammi, J. W. Ochterski, R. L. Martin, K. Morokuma, O. Farkas, J. B. Foresman, and D. J. Fox, Gaussian, Inc., Wallingford CT, 2016.

11 (a) A. D. Becke, *Phys. Rev. A*, 1988, **38**, 3098−3100; (b) J. P. Perdew, *Phys. Rev. B*, 1986, **33**, 8822−8824.

12 F. Weigend, R. Ahlrichs, *Phys., Chem. Chem. Phys.*, 2005, **7**, 3297−3305.

13 A. V. Marenich, C. J. Cramer, D. G. Truhlar, *Journal of Physical Chemistry B*, 2009, **113**, 6378-6396.

14 (a) S. Grimme, J. Antony, S. Ehrlich and H. Krieg, *J. Chem. Phys.*, 2010, **132**, 154104; (b) S. Grimme, S. Ehrlich, L. Goerigk, *Journal of computational chemistry,* 2011, **32**, 1456-1465.

5. NMR Spectra

31P{1H}-NMR (162 MHz, D2O)

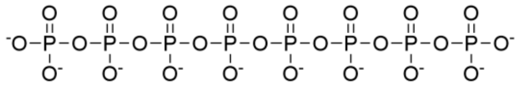

6

sodium salt

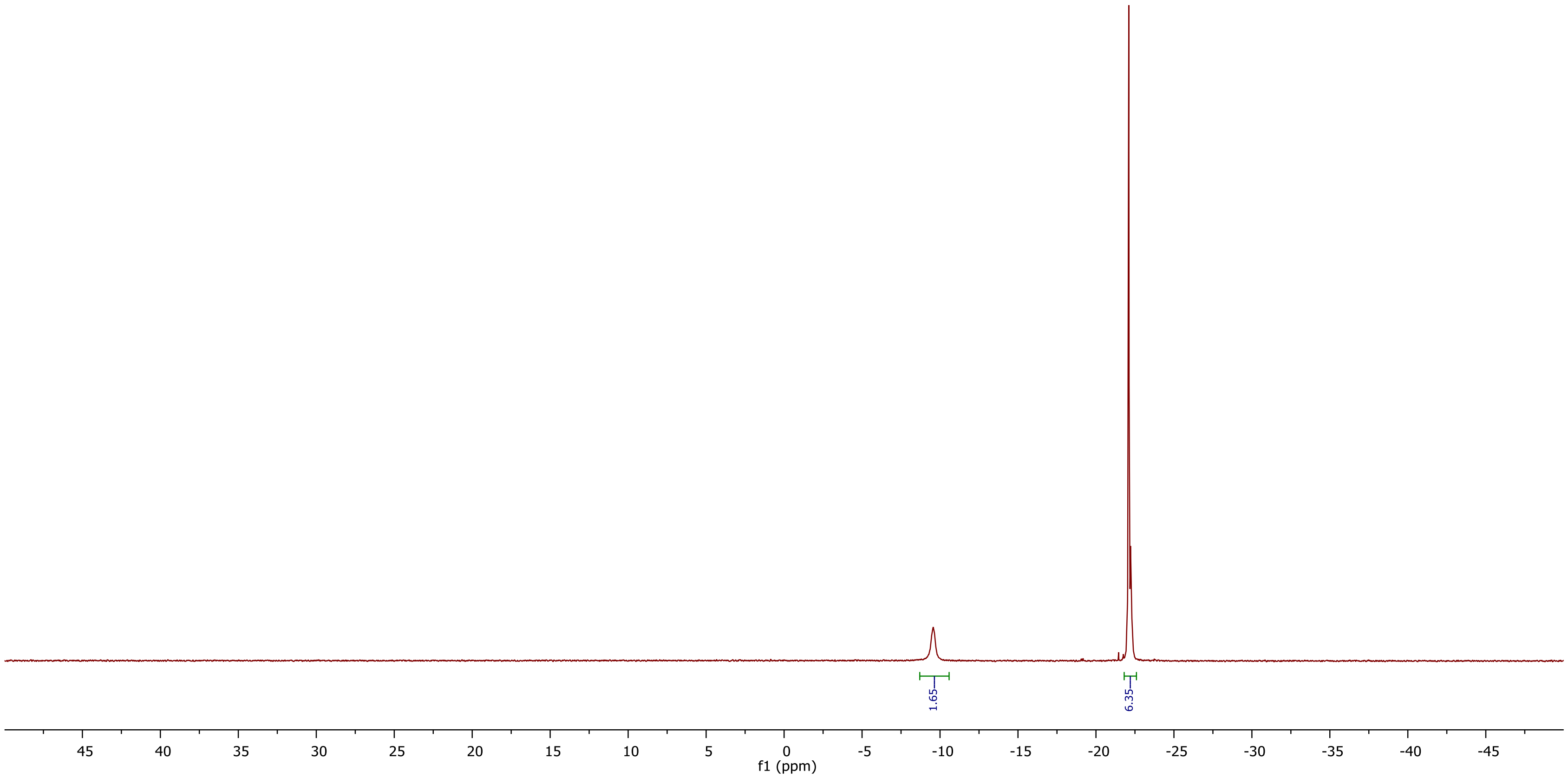

1H-NMR (400 MHz, CDCl3)

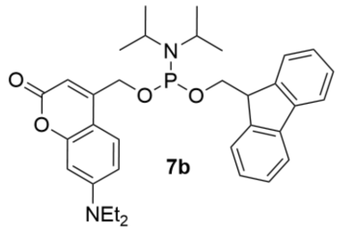

7b

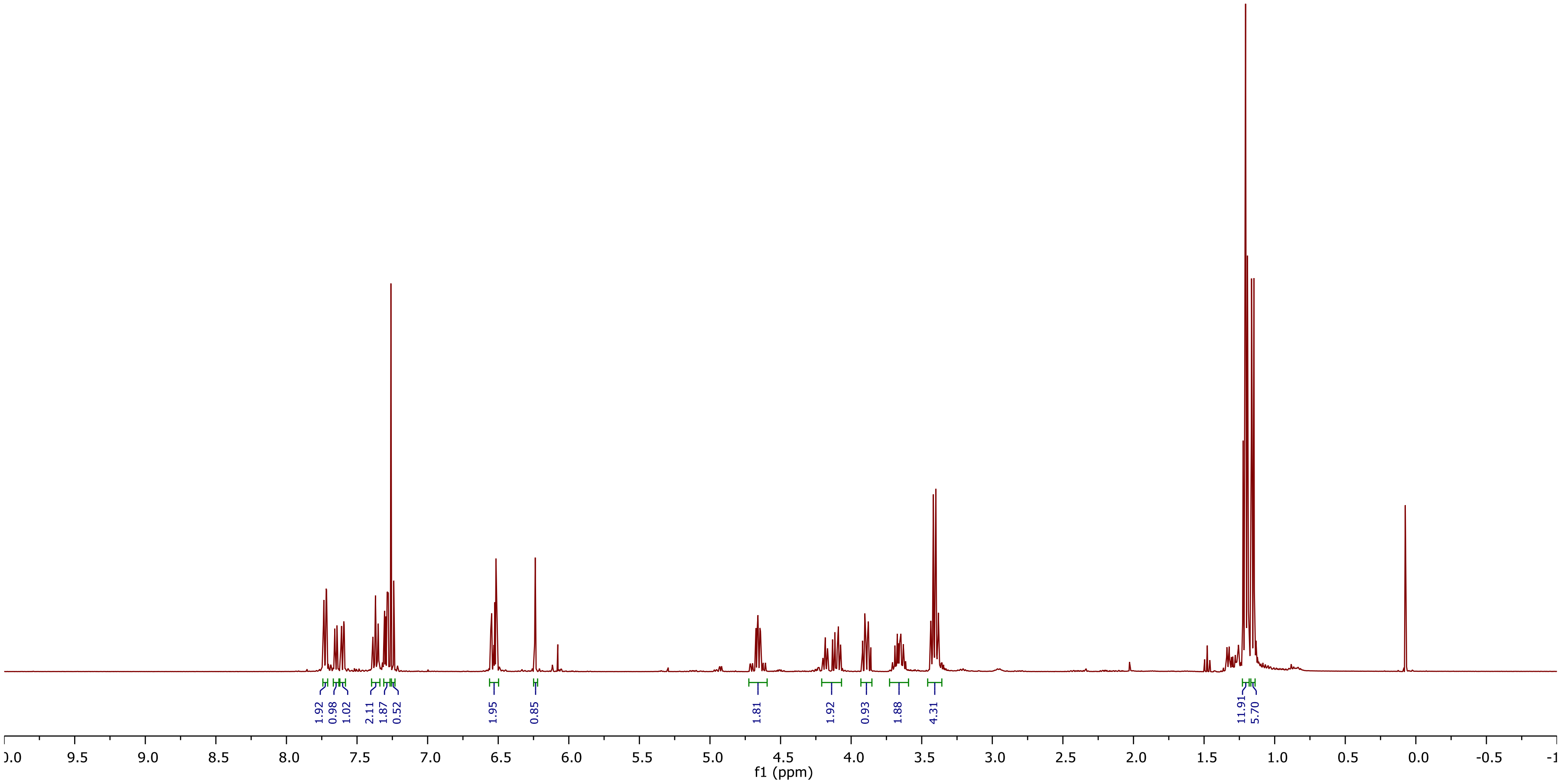

31P{1H}-NMR (162 MHz, CDCl3)

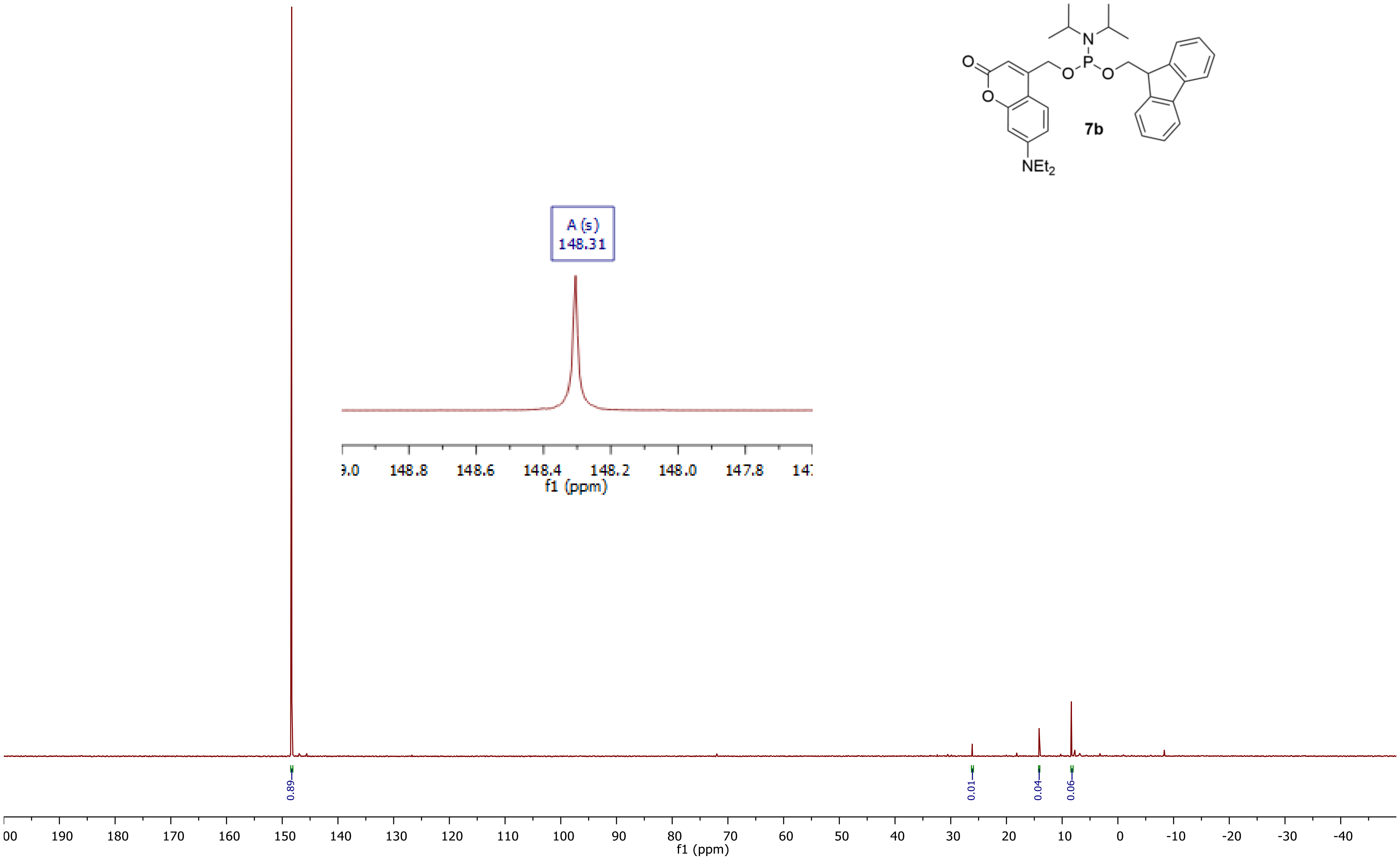

7b

31P-NMR (162 MHz, CDCl3)

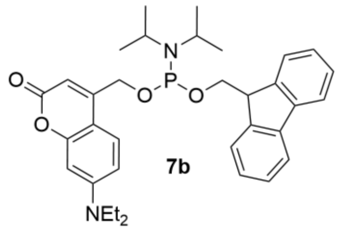

7b

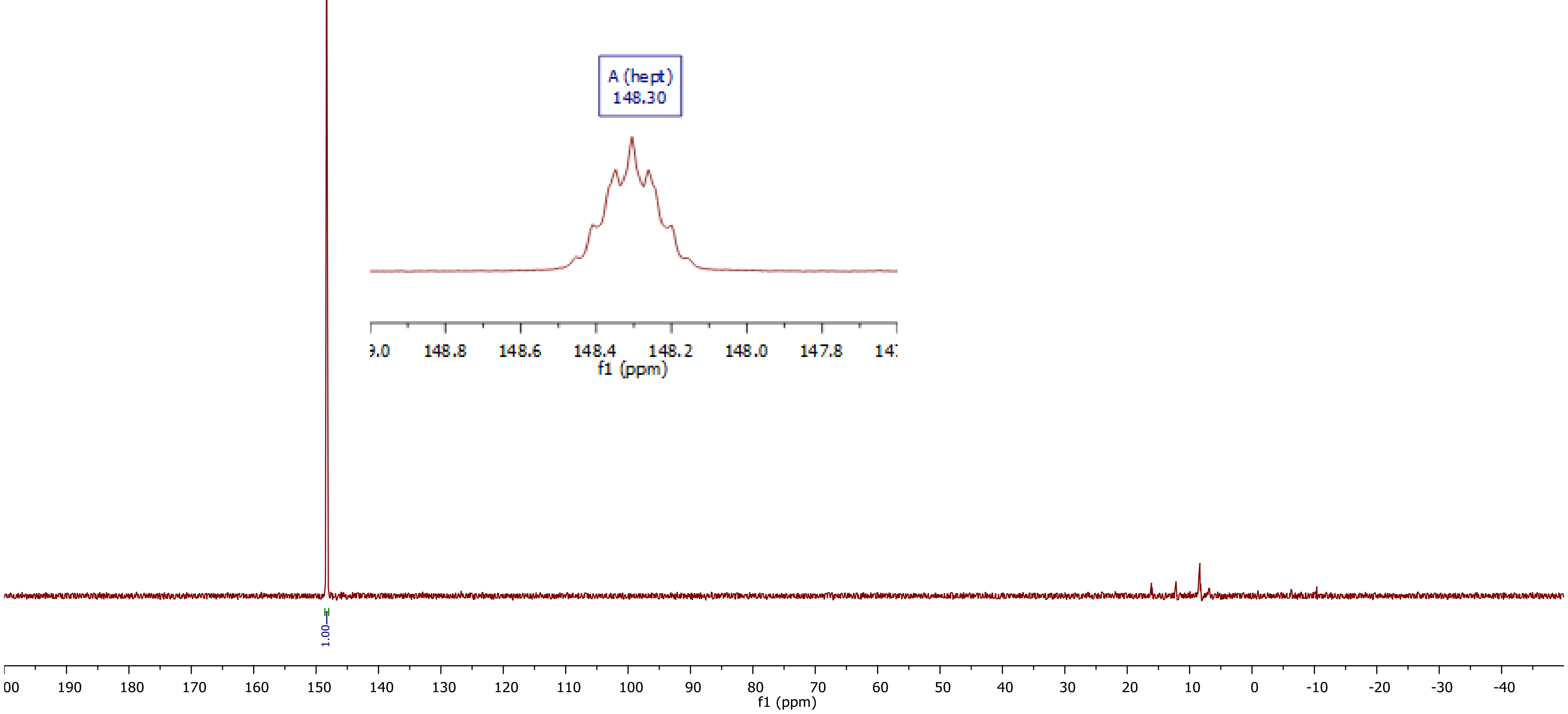

13C-NMR (101 MHz, CDCl3)

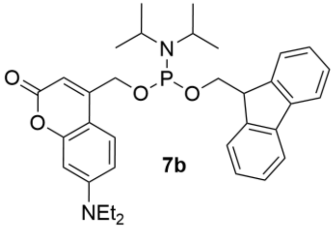

7b

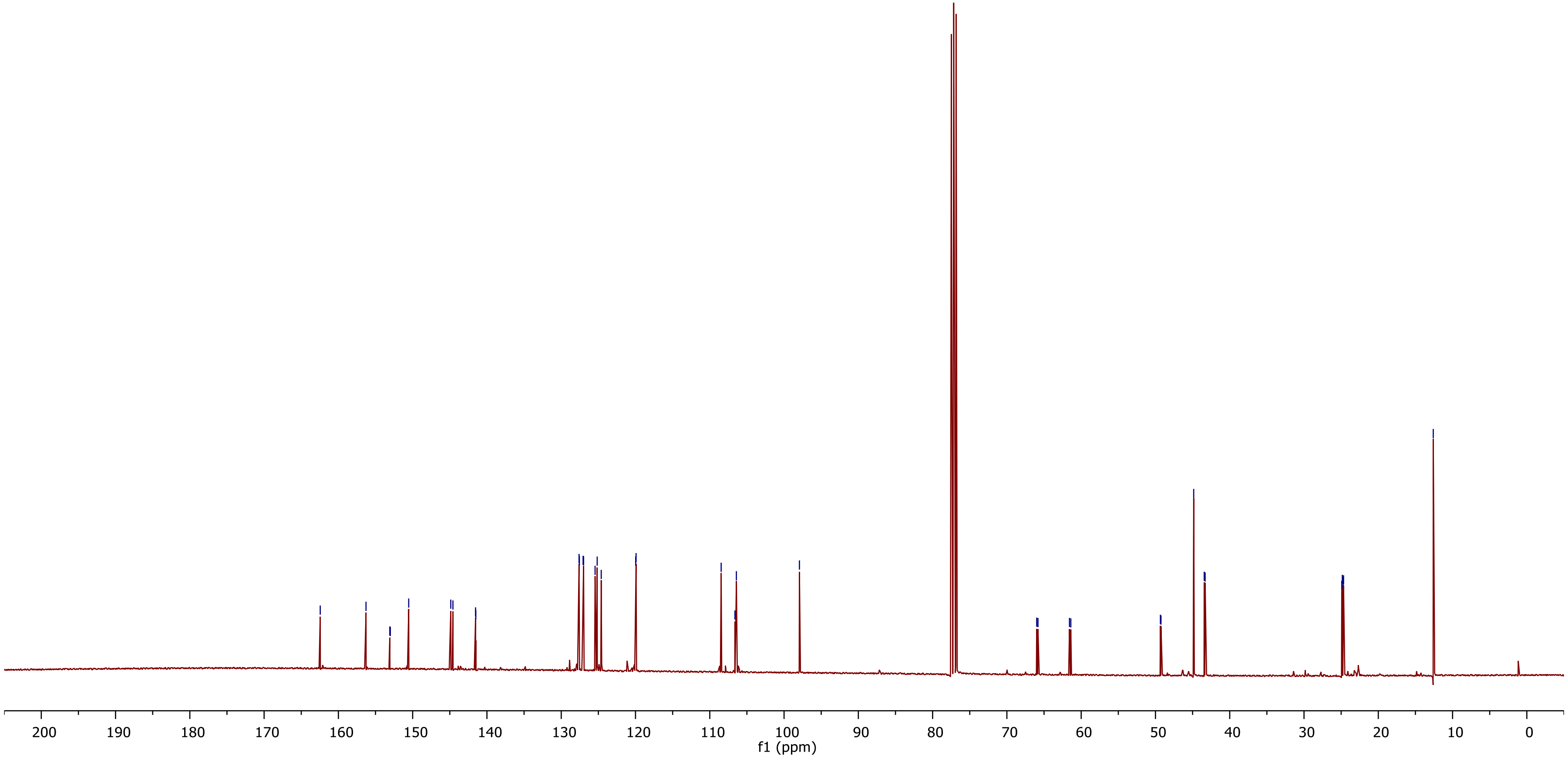

1H-NMR (400 MHz, D2O)

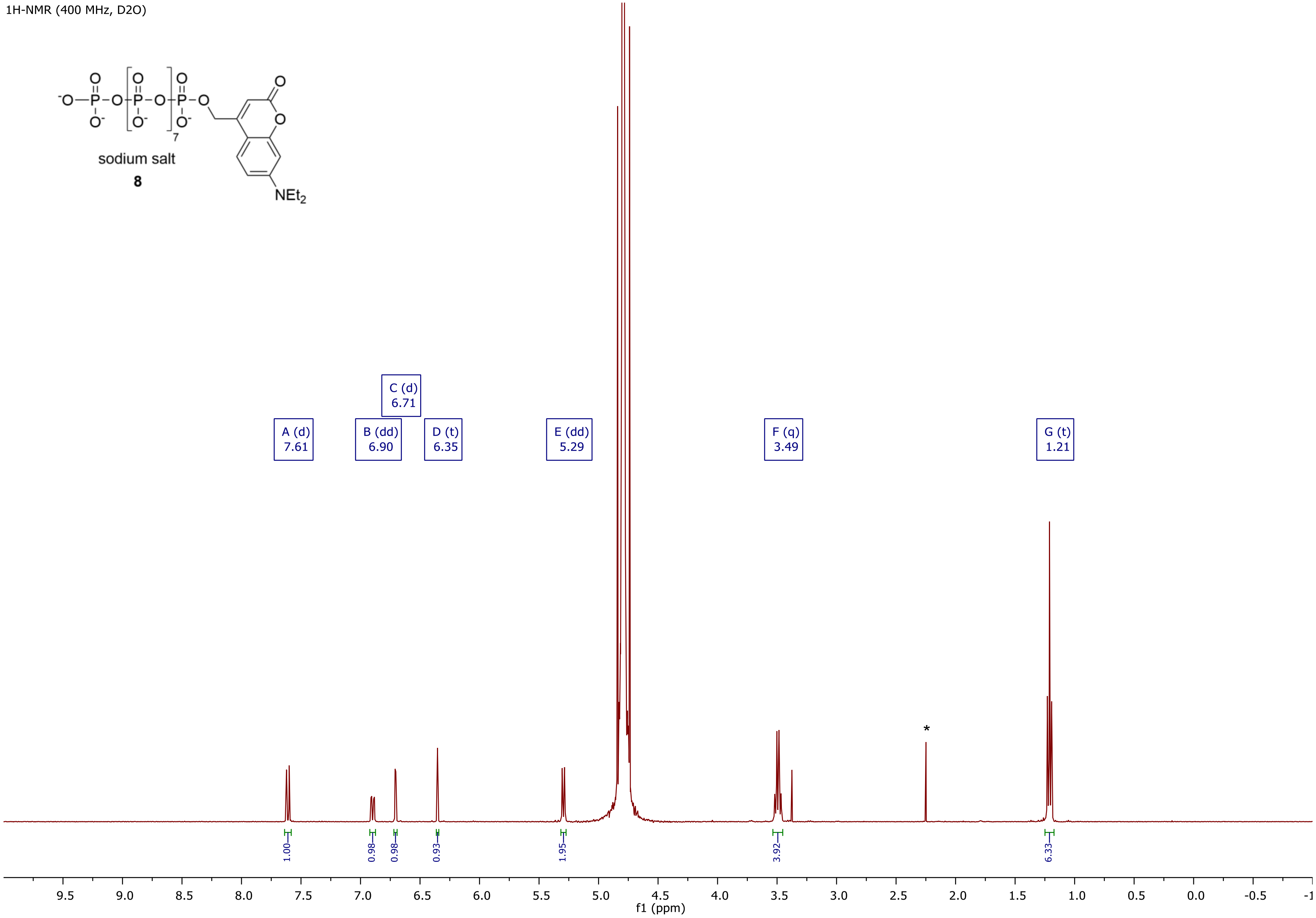

Residual amounts of acetone are marked with asterisks (*).

31P{1H}-NMR (162 MHz, D2O)

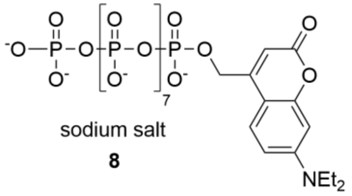

sodium salt
8

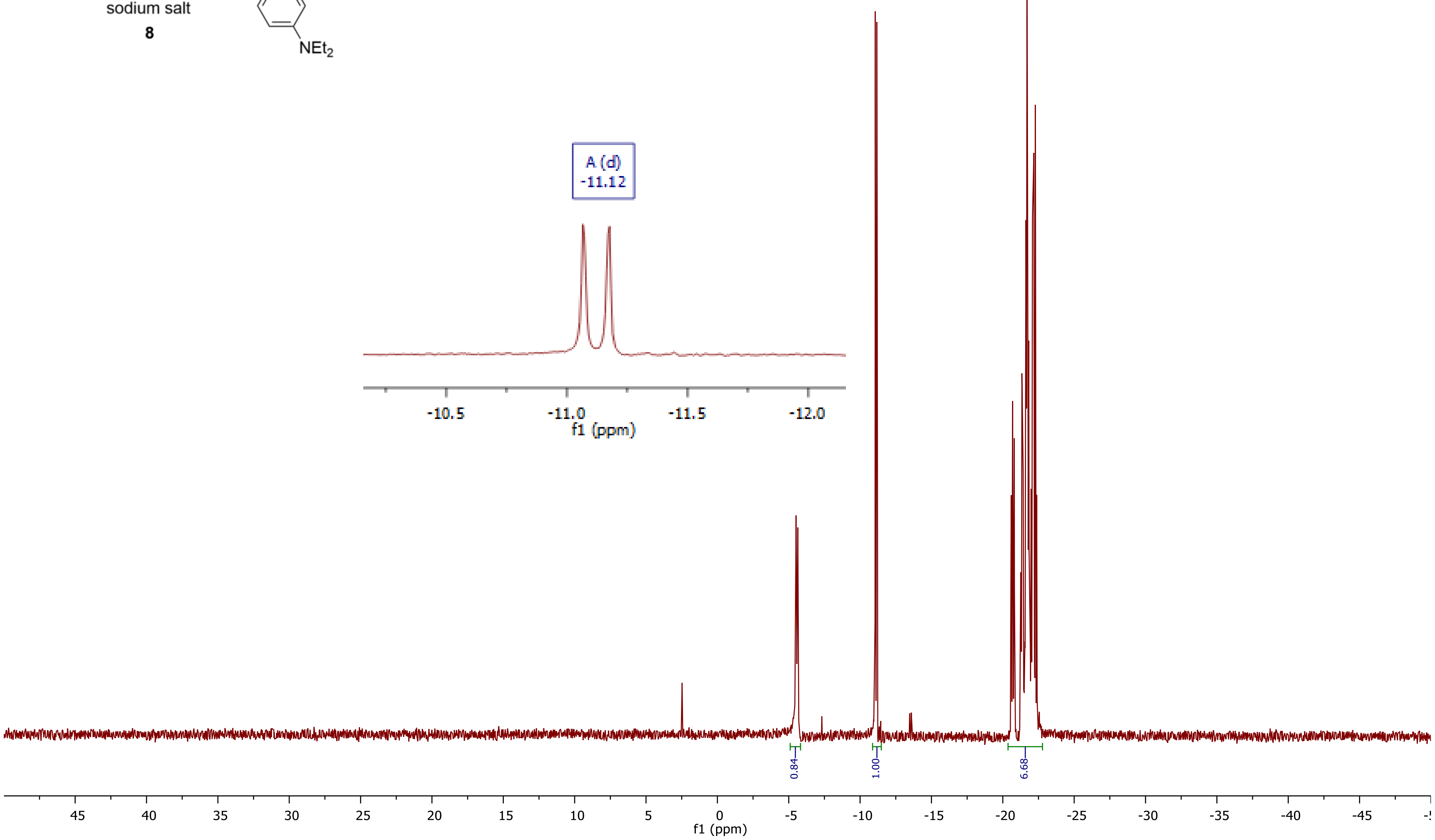

31P-NMR (162 MHz, D2O)

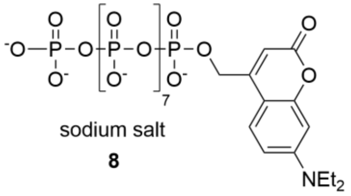

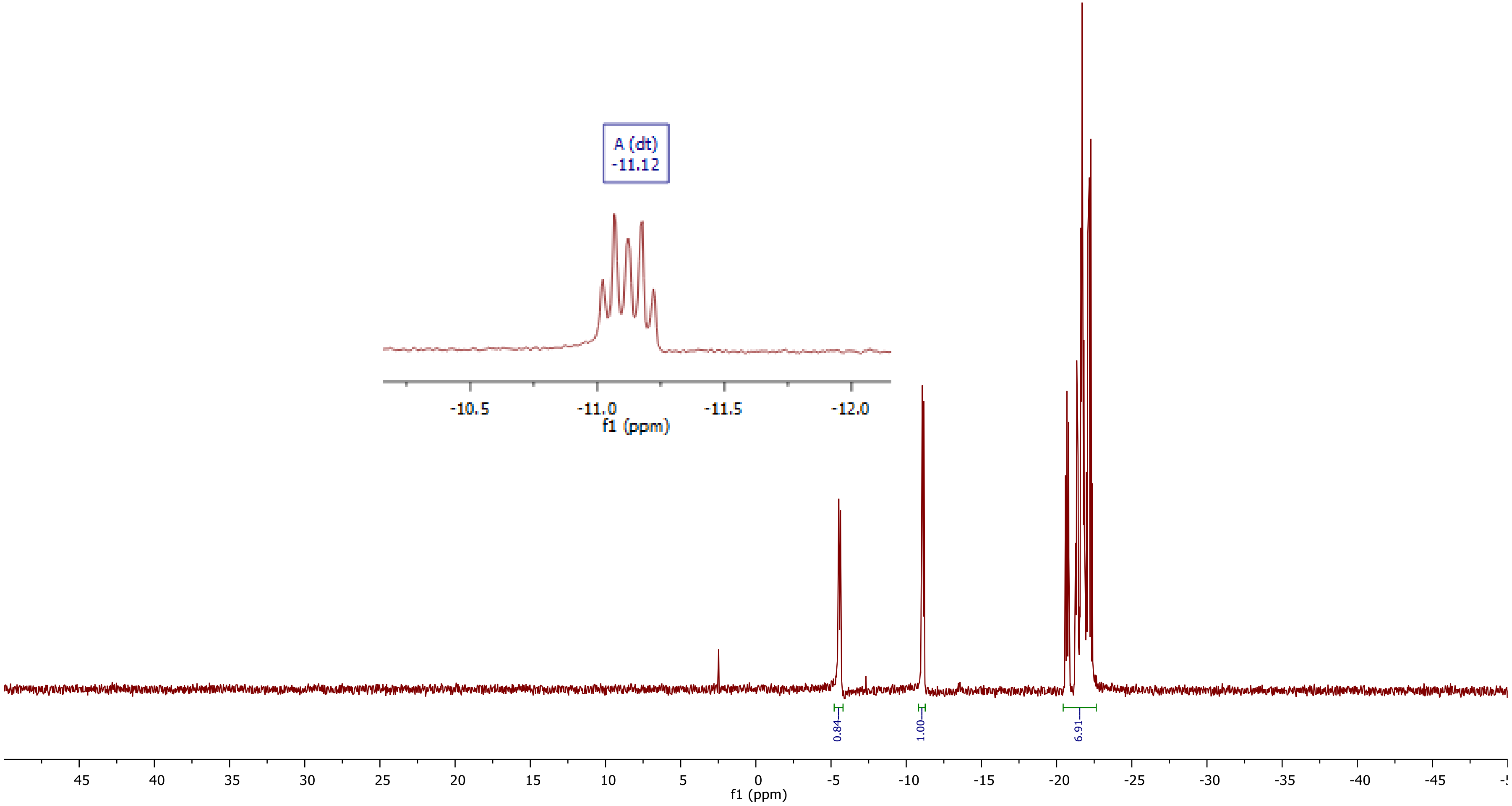

13C-NMR (101 MHz, D2O)

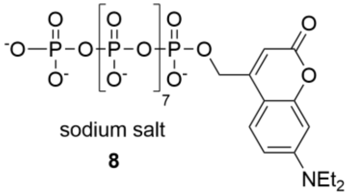

8

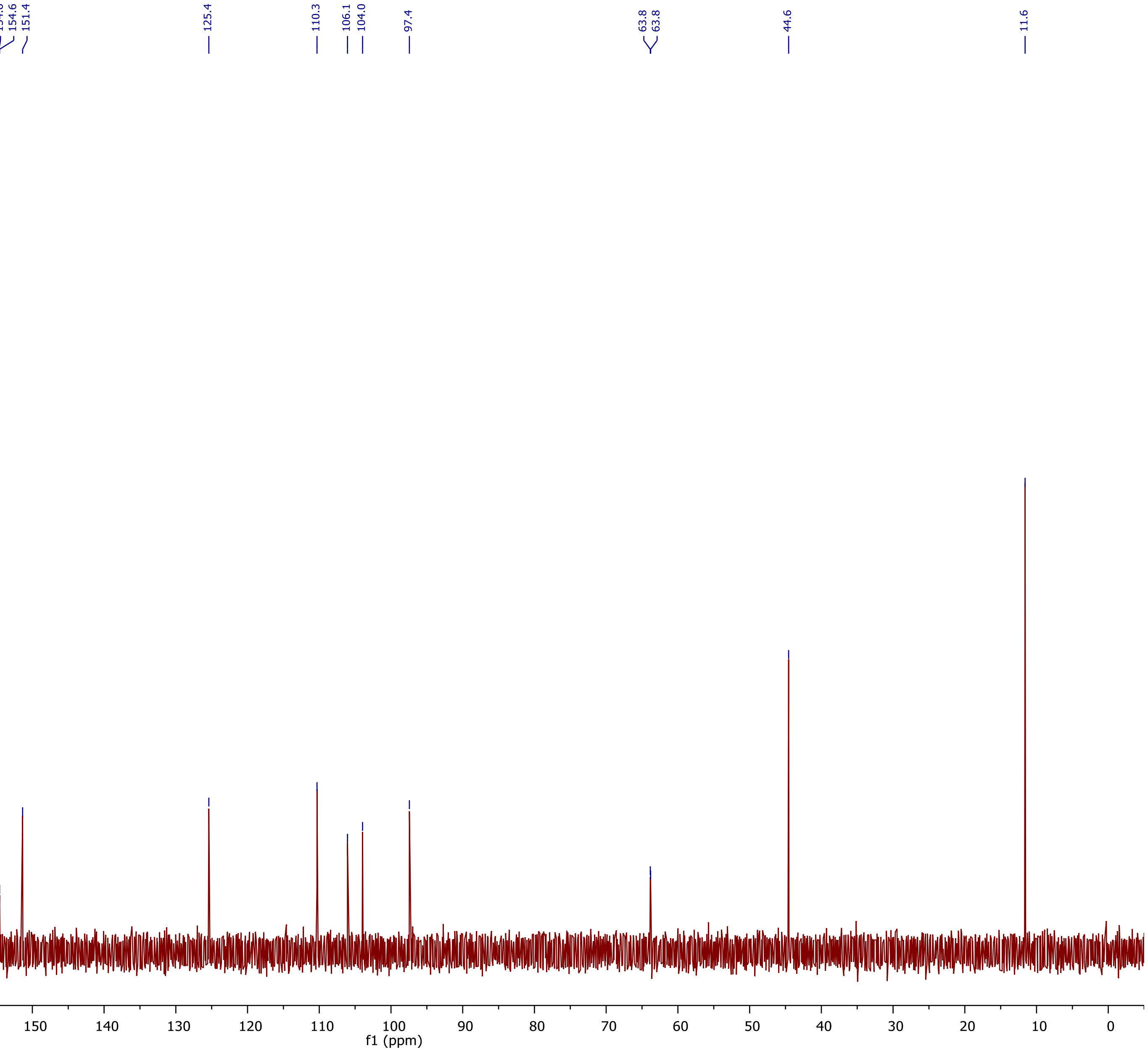

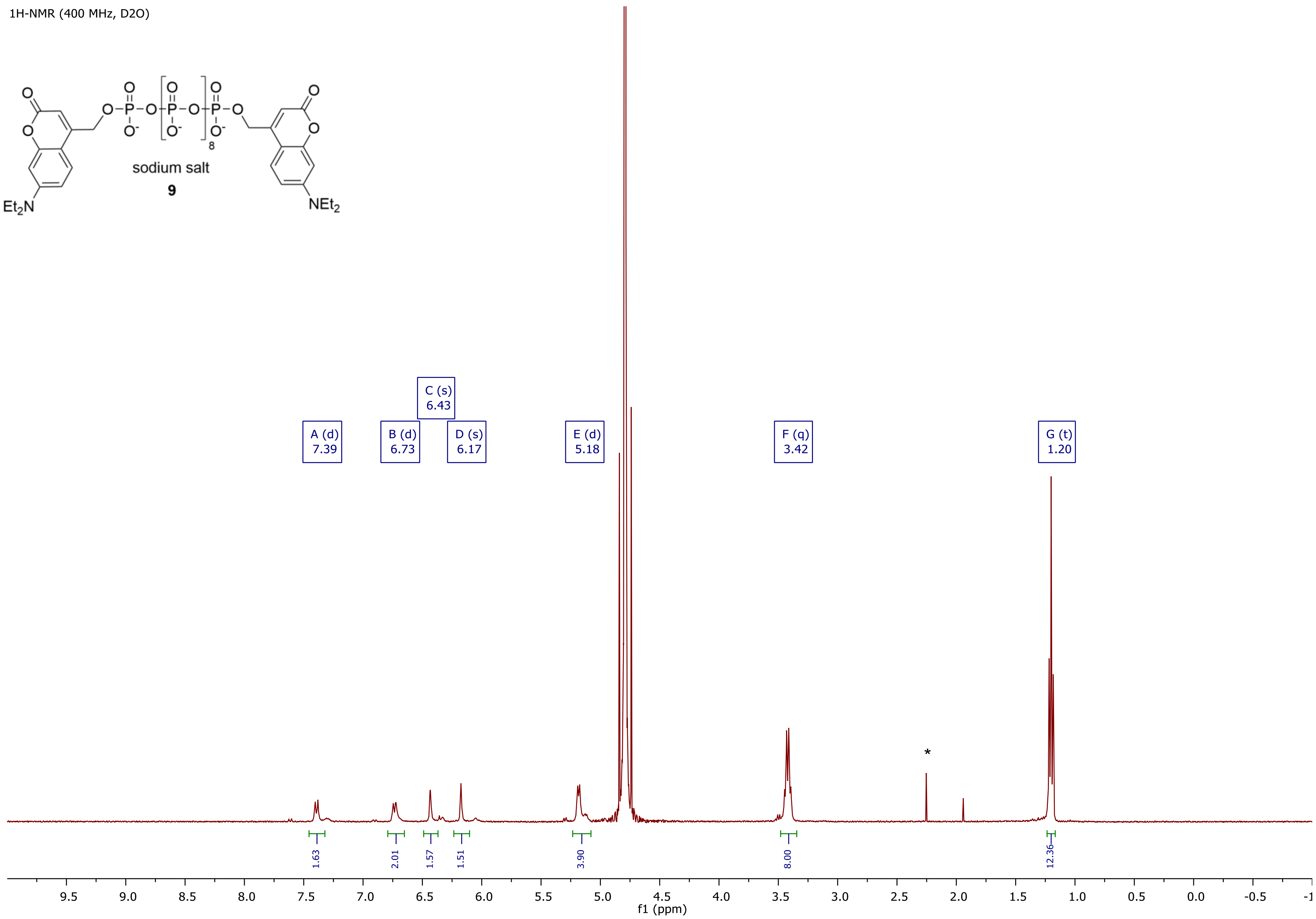

Residual amounts of acetone are marked with asterisks (*).

31P{1H}-NMR (162 MHz, D2O)

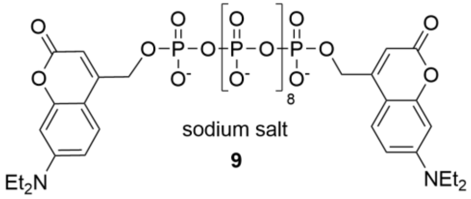

sodium salt

9

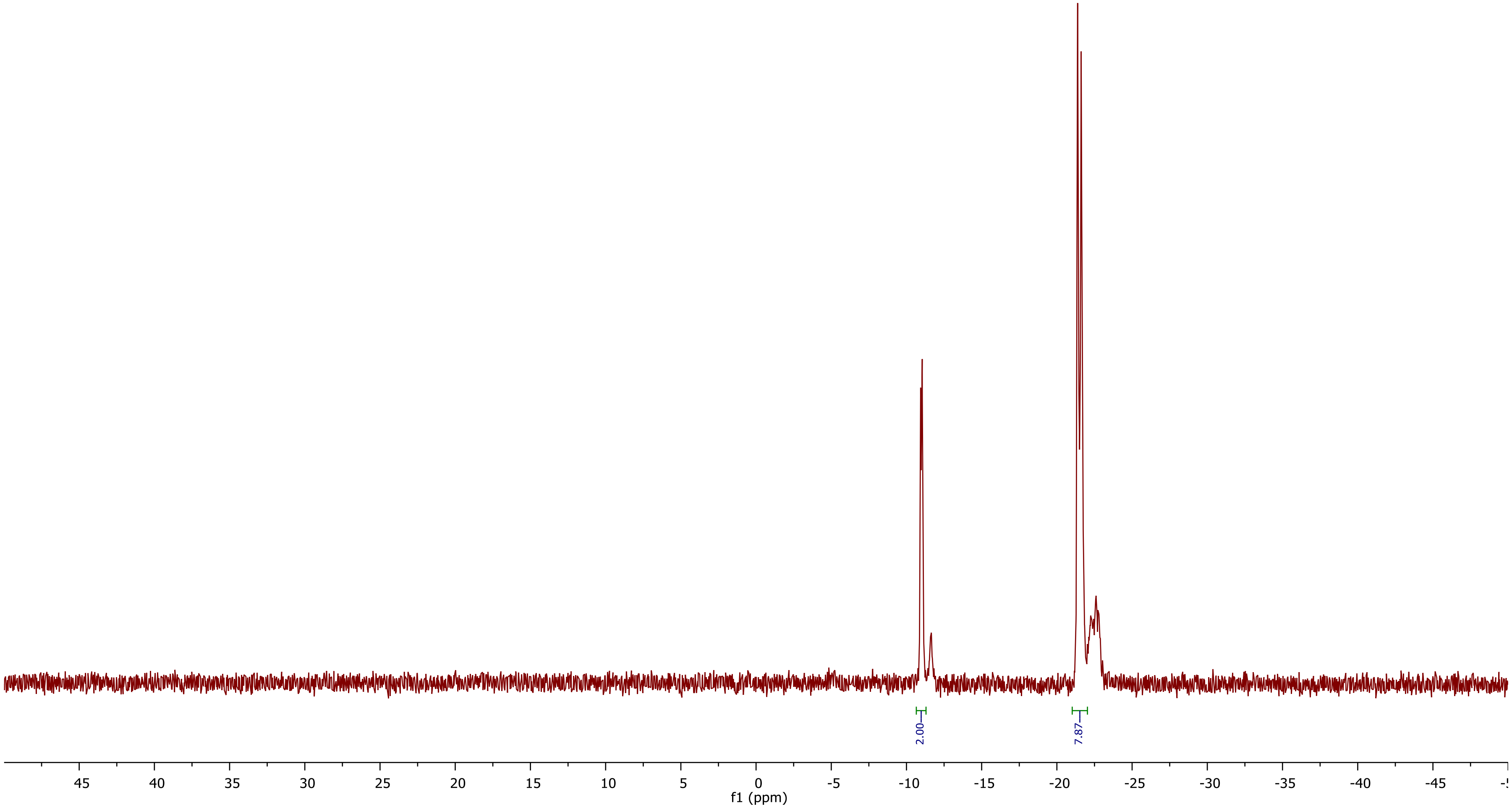

13C-NMR (101 MHz, D2O)

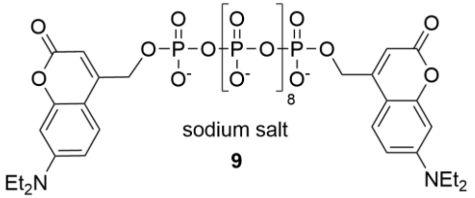

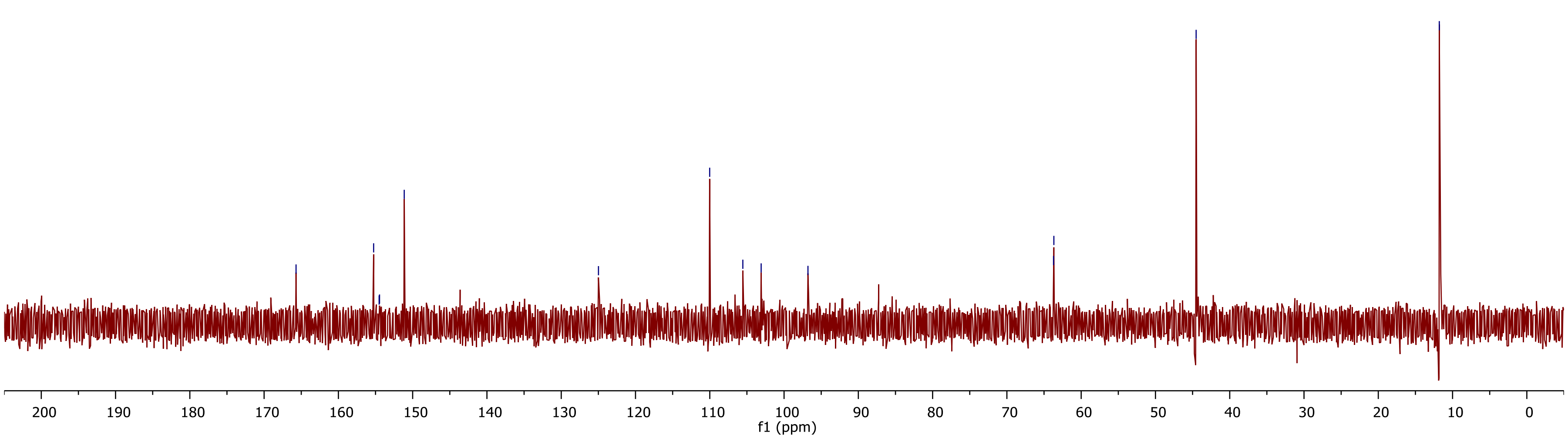

1H-NMR (700 MHz, D2O)

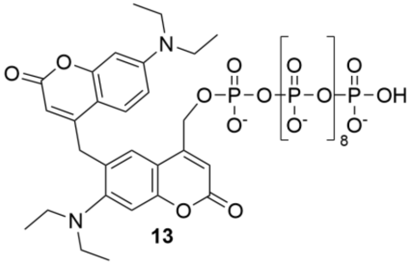

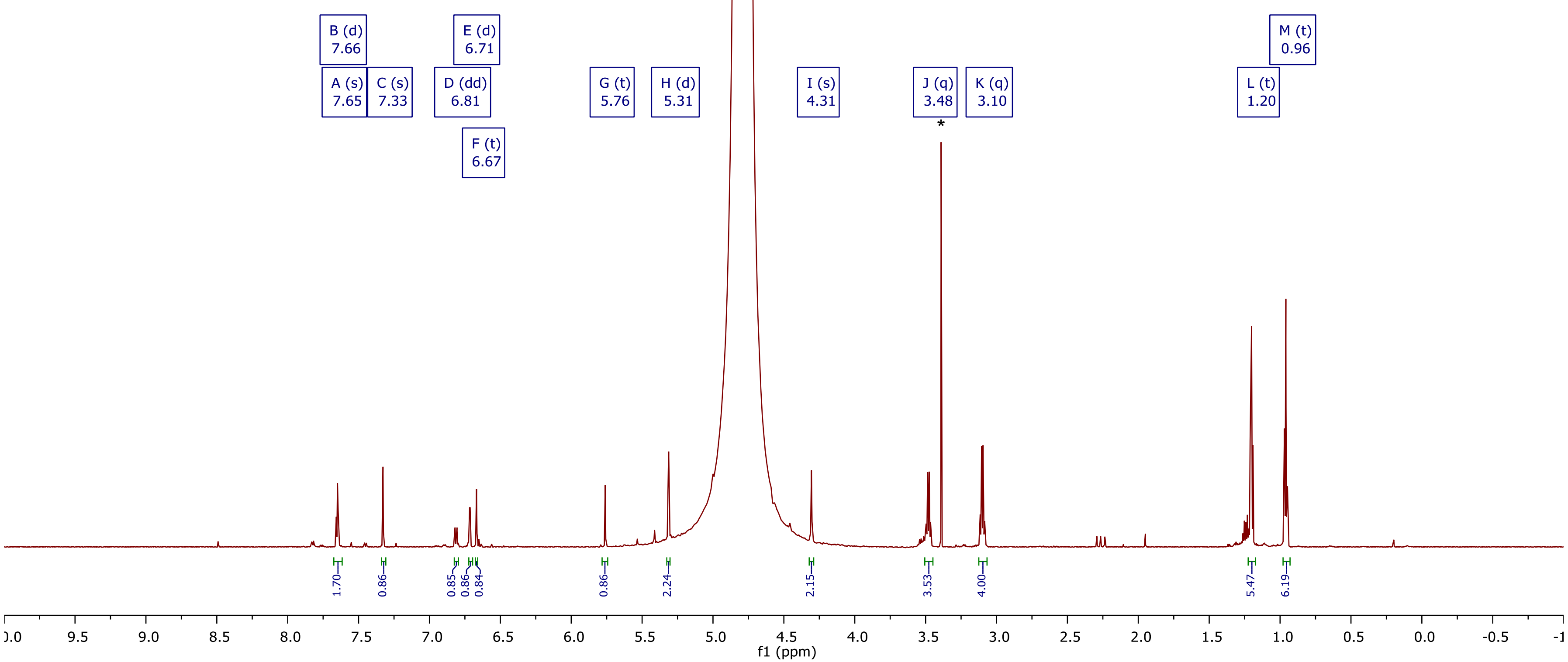

Methanol impurity is marked with asterisks (*).

$^{31}P\{^1H\}$NMR (283 MHz, D_2O)

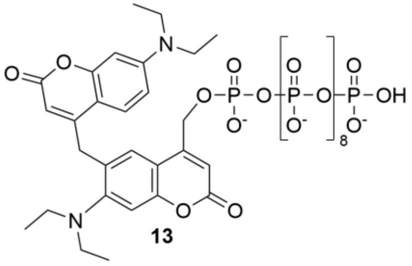

13

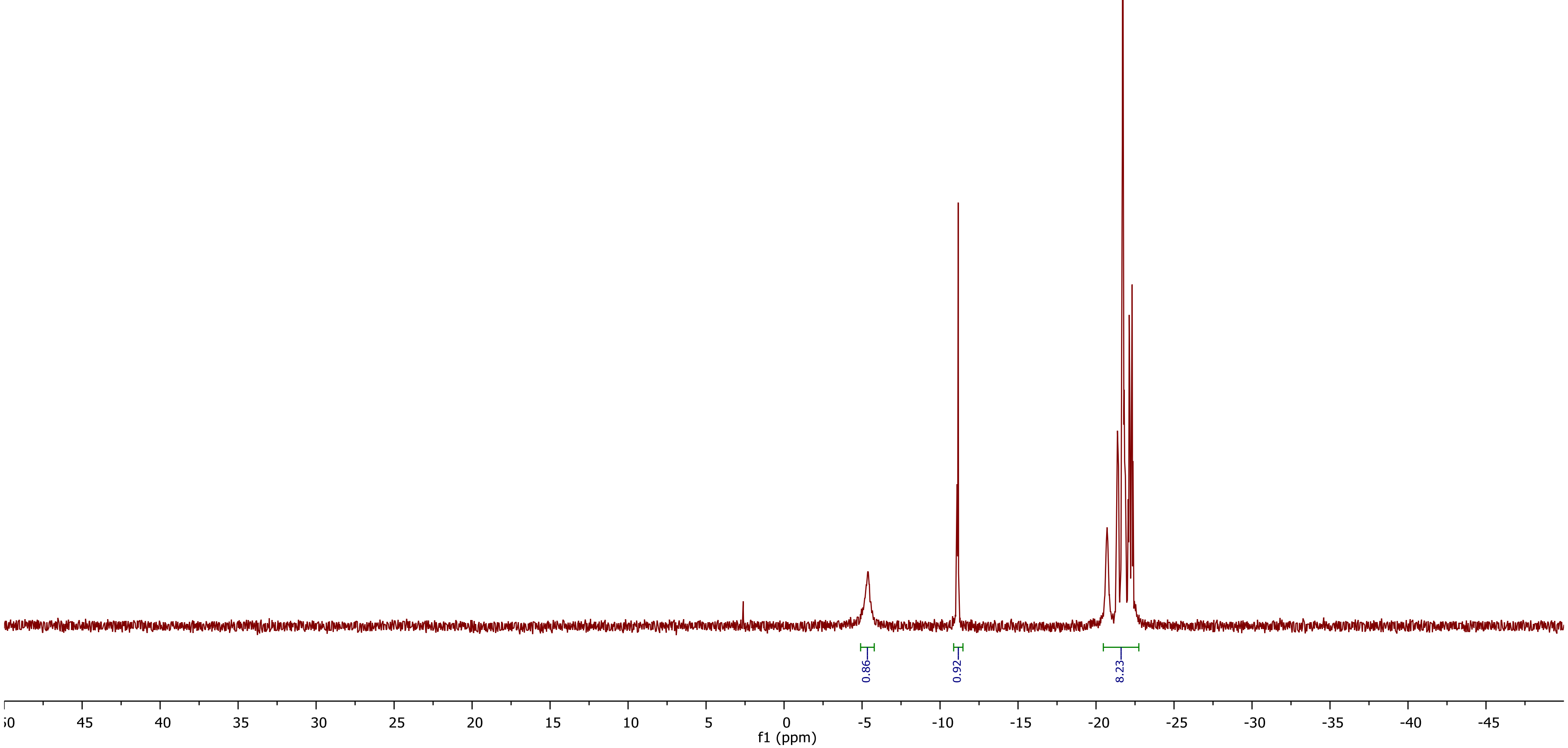

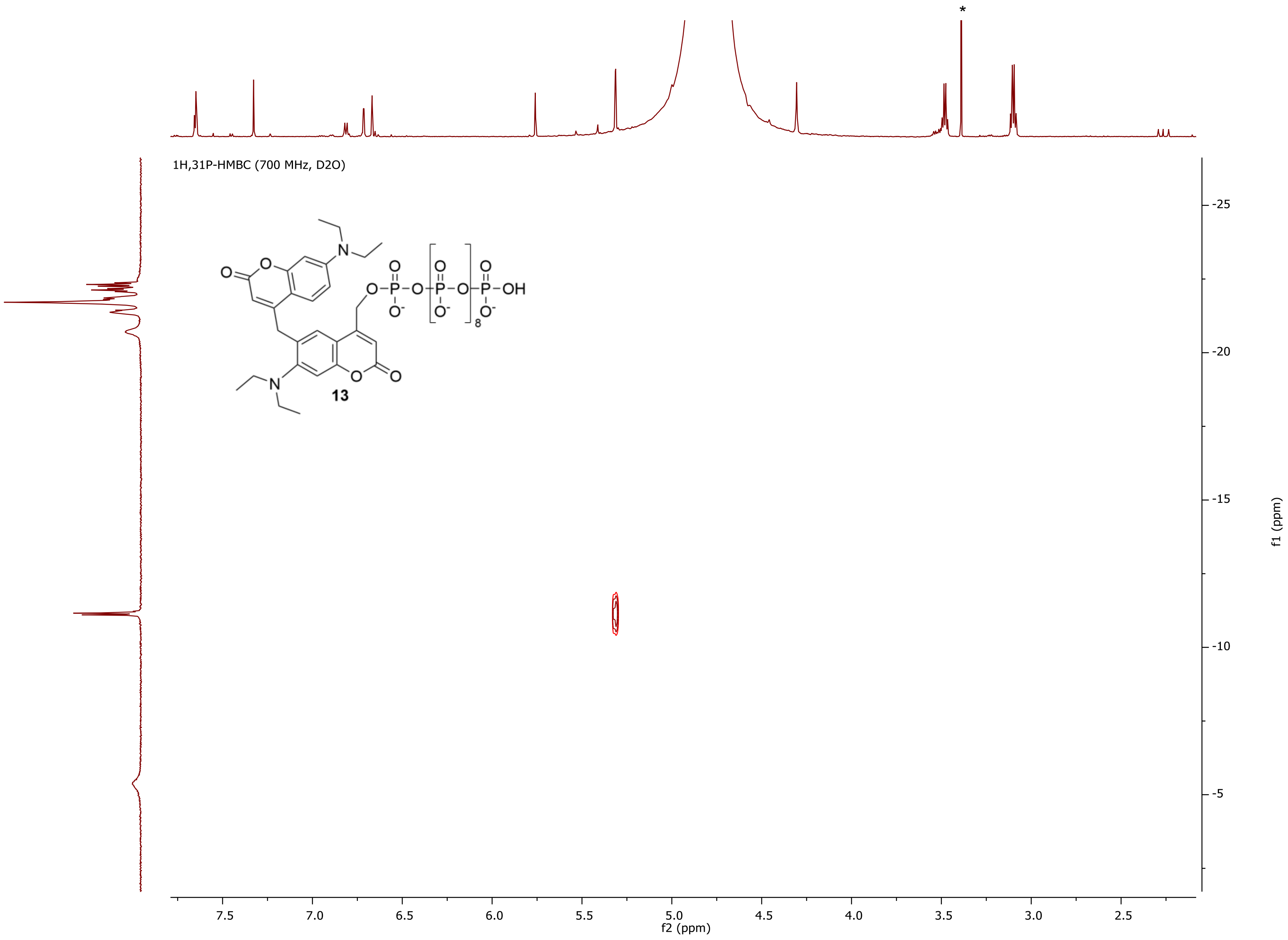

Methanol impurity is marked with asterisks (*).

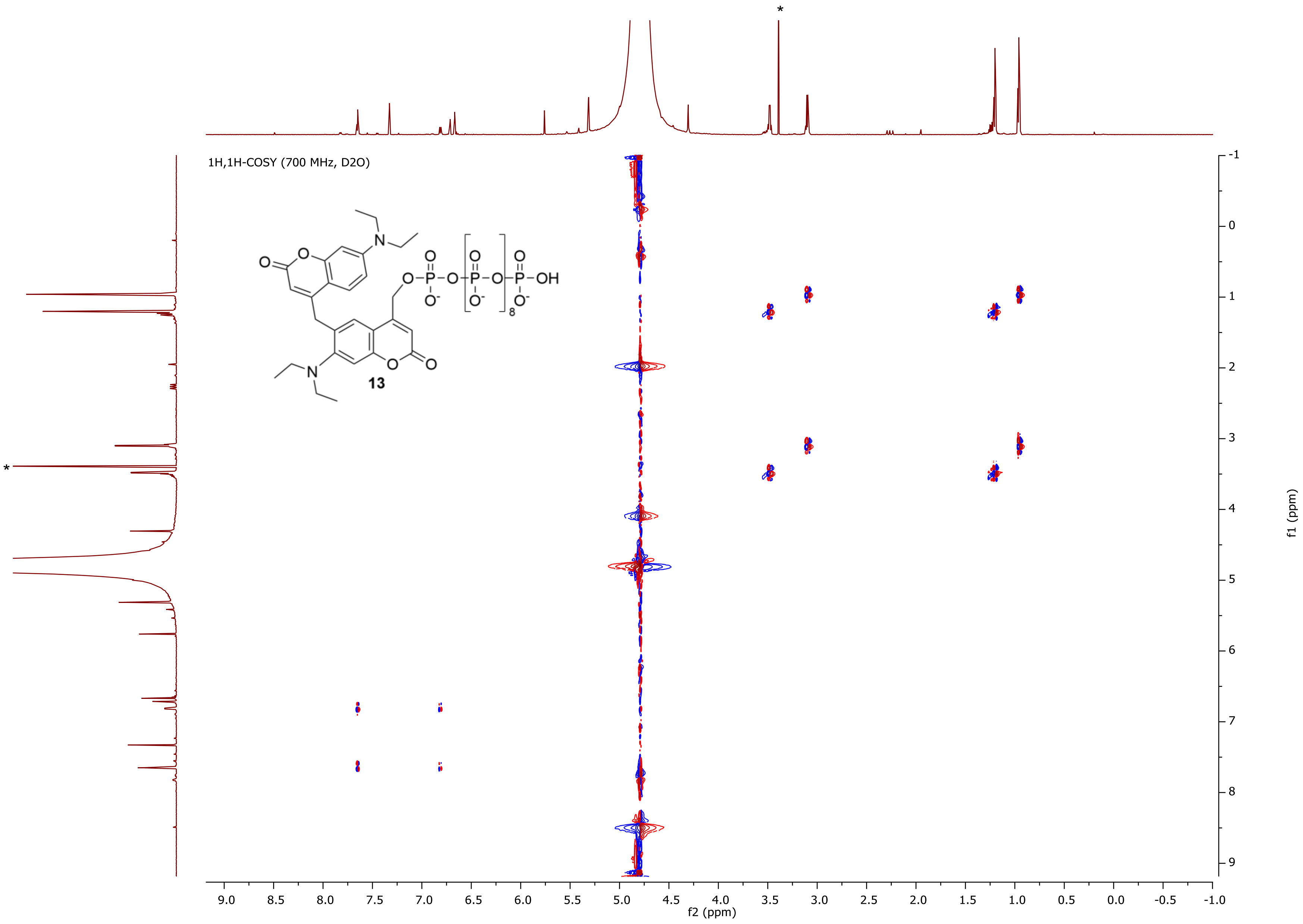

Methanol impurity is marked with asterisks (*).

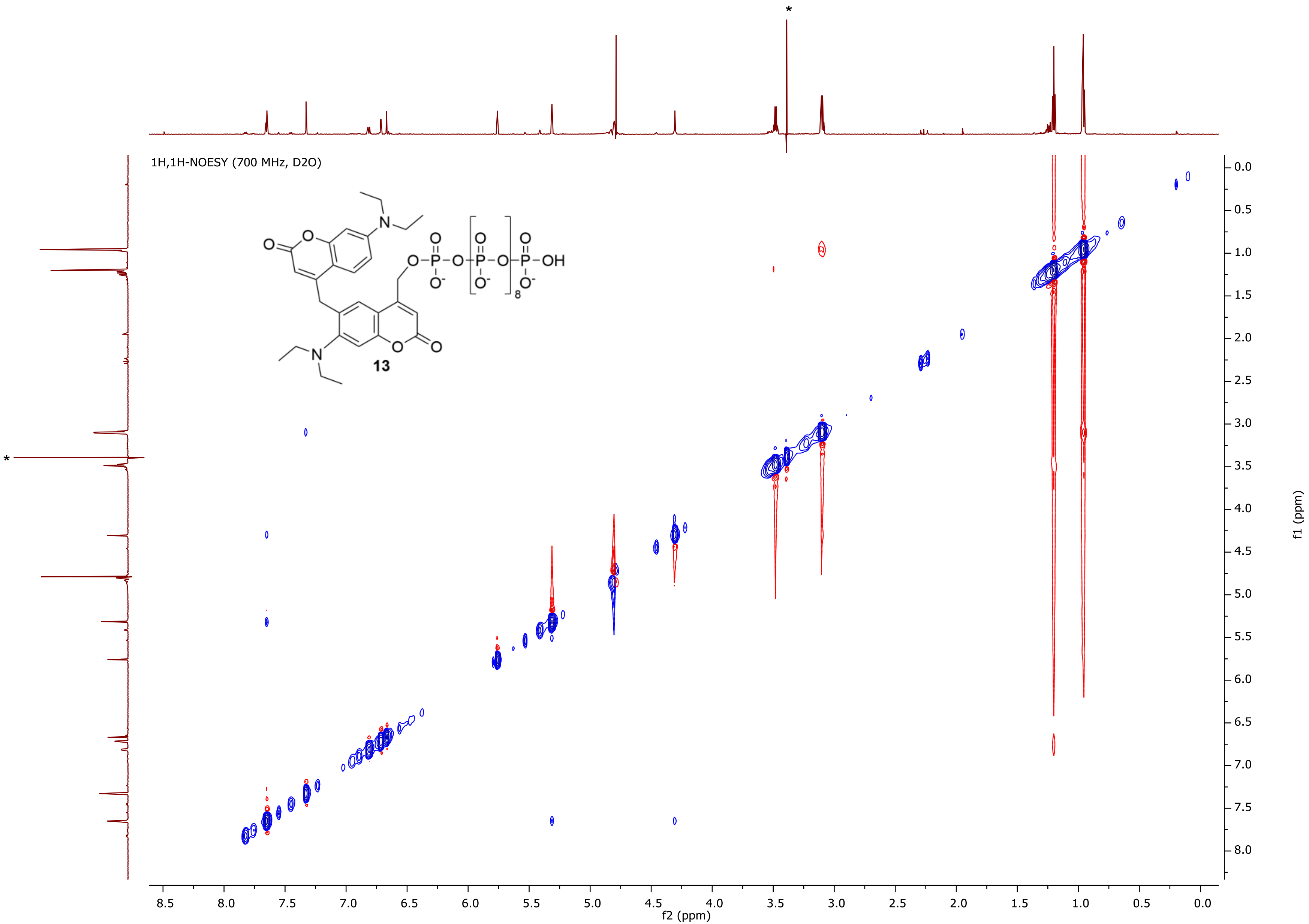

Methanol impurity is marked with asterisks (*).

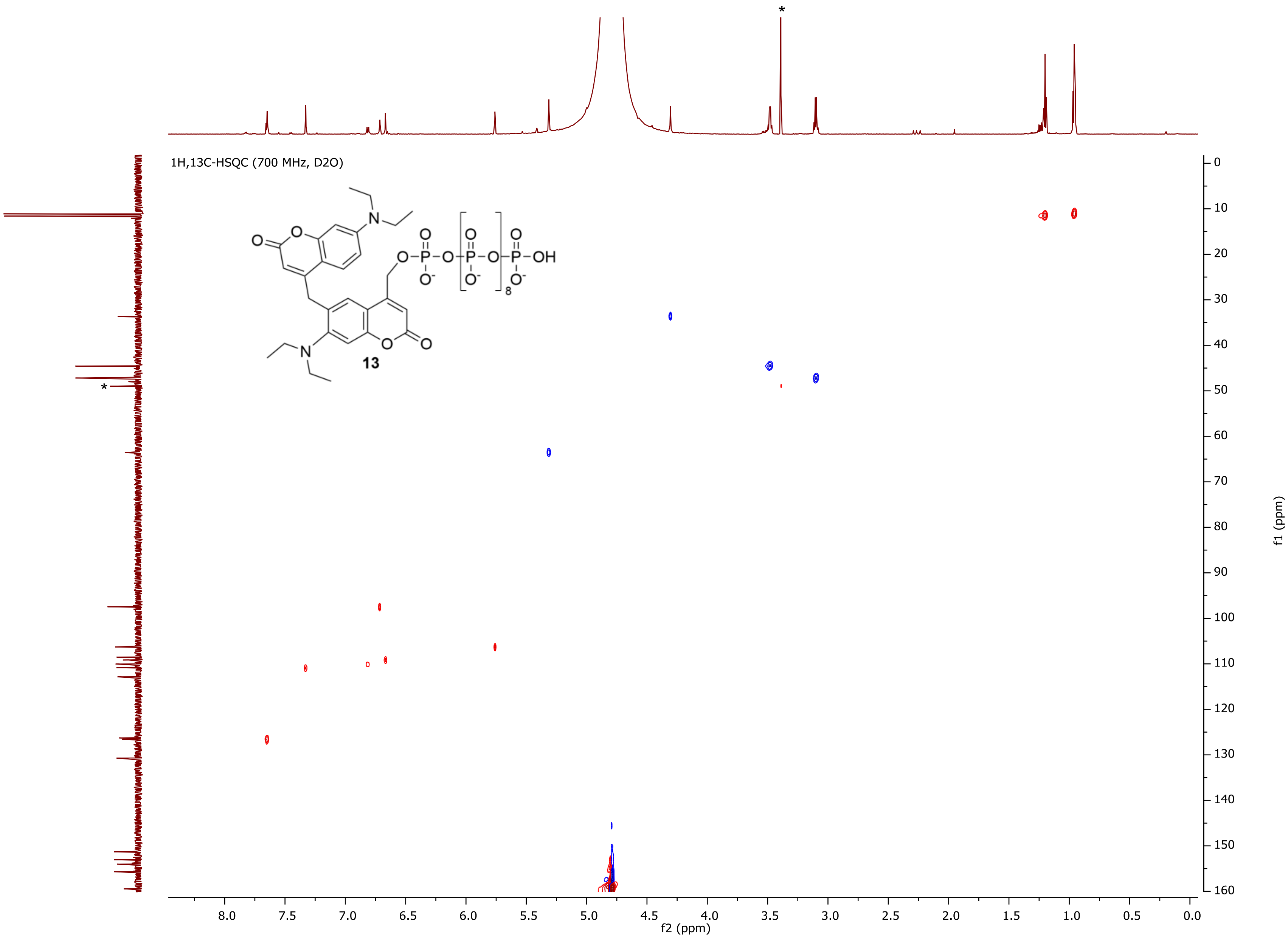

Methanol impurity is marked with asterisks (*).

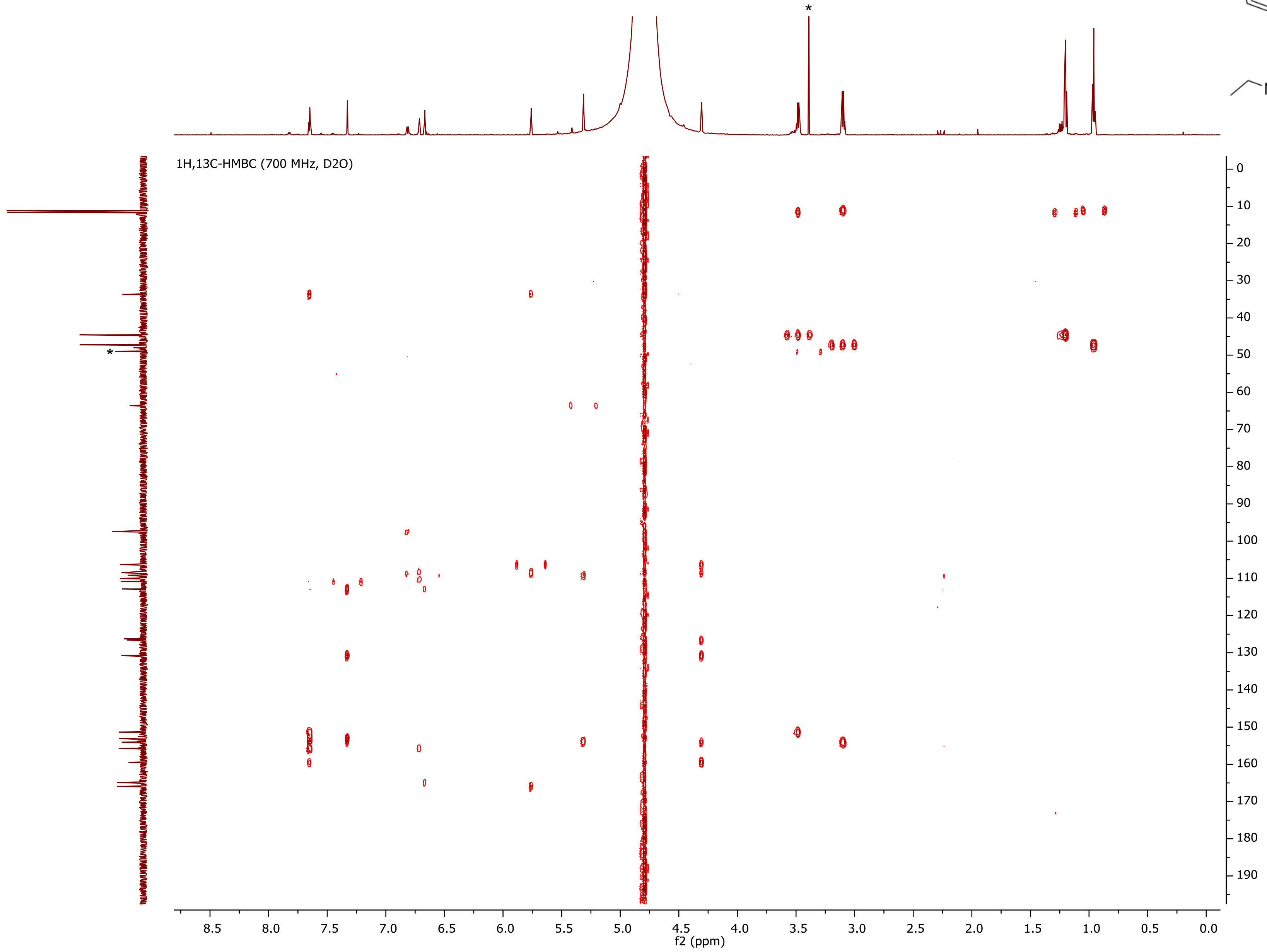

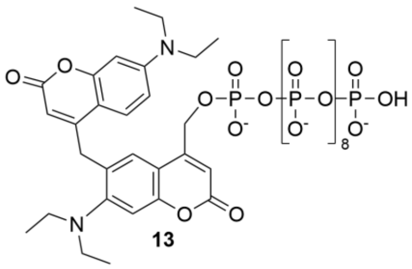

13

Methanol impurity is marked with asterisks (*).

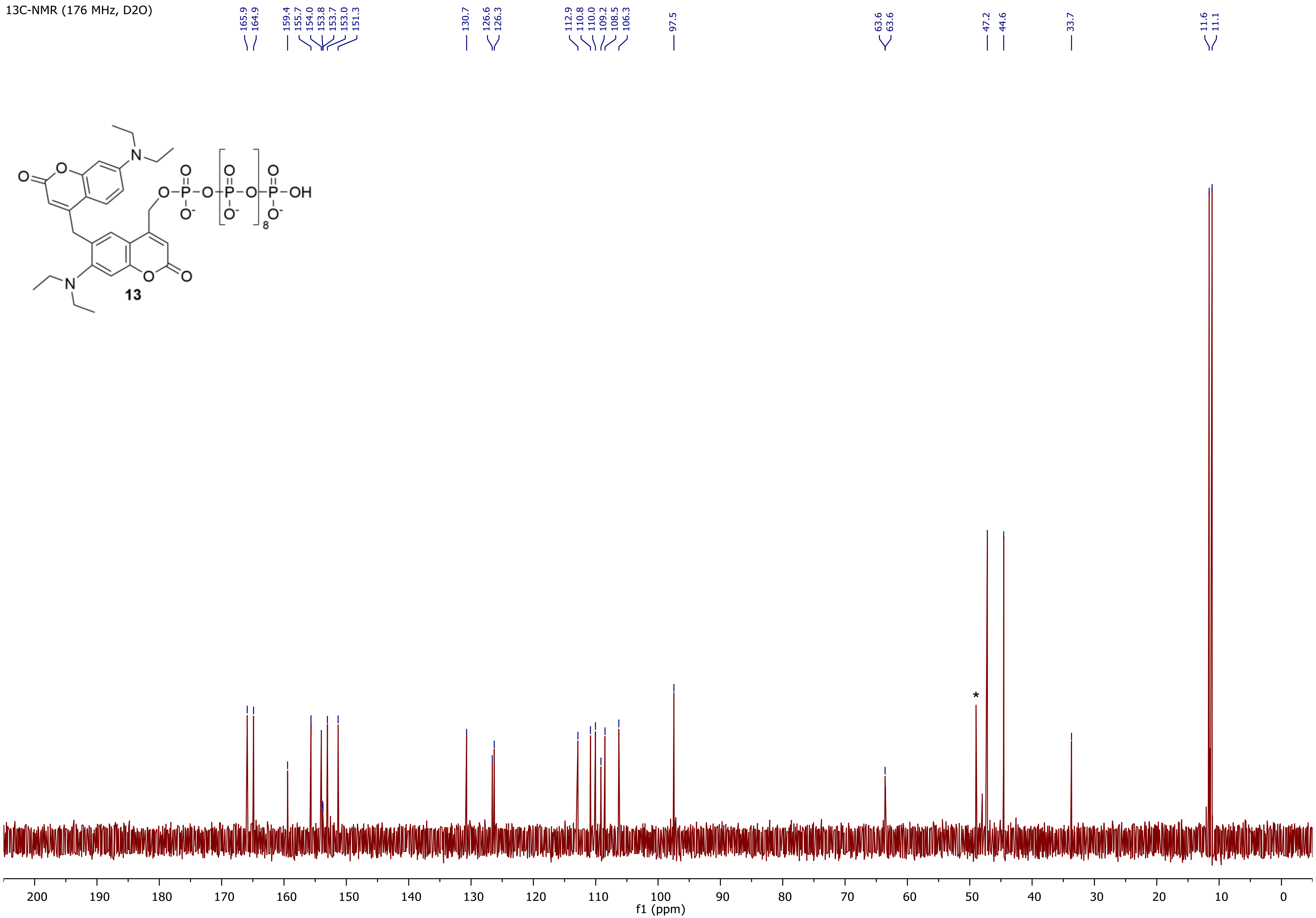

Methanol impurity is marked with asterisks (*).

31P{1H}-NMR (162 MHz, D2O)

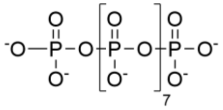

sodium salt

10

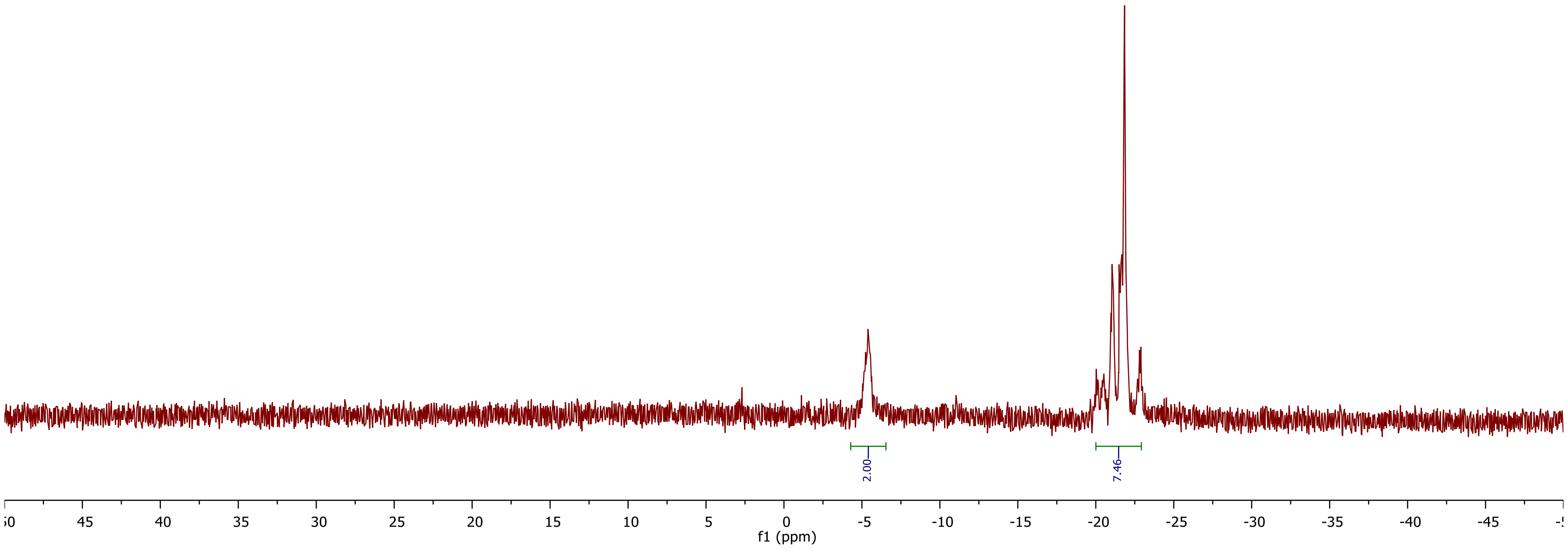

31P{1H}-NMR (162 MHz, D2O)

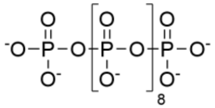

sodium salt

11

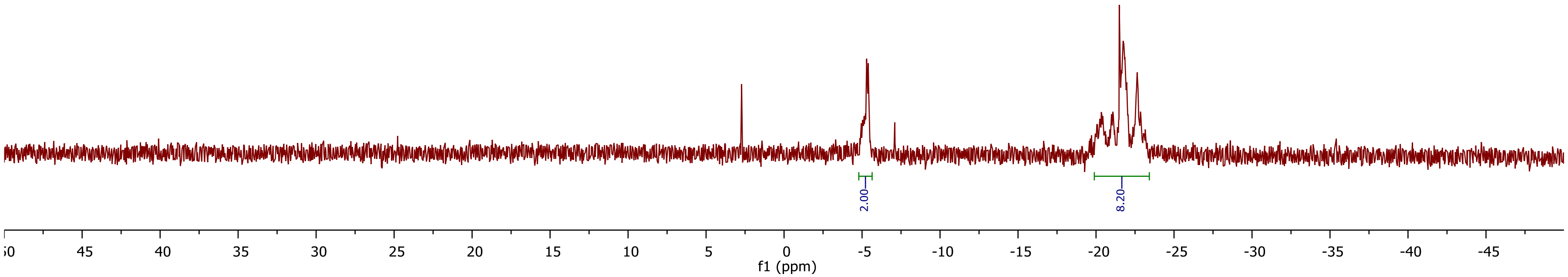

1H-NMR (400 MHz, CDCl3)

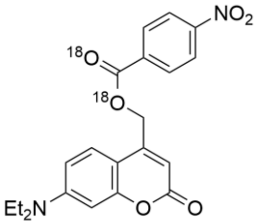

32

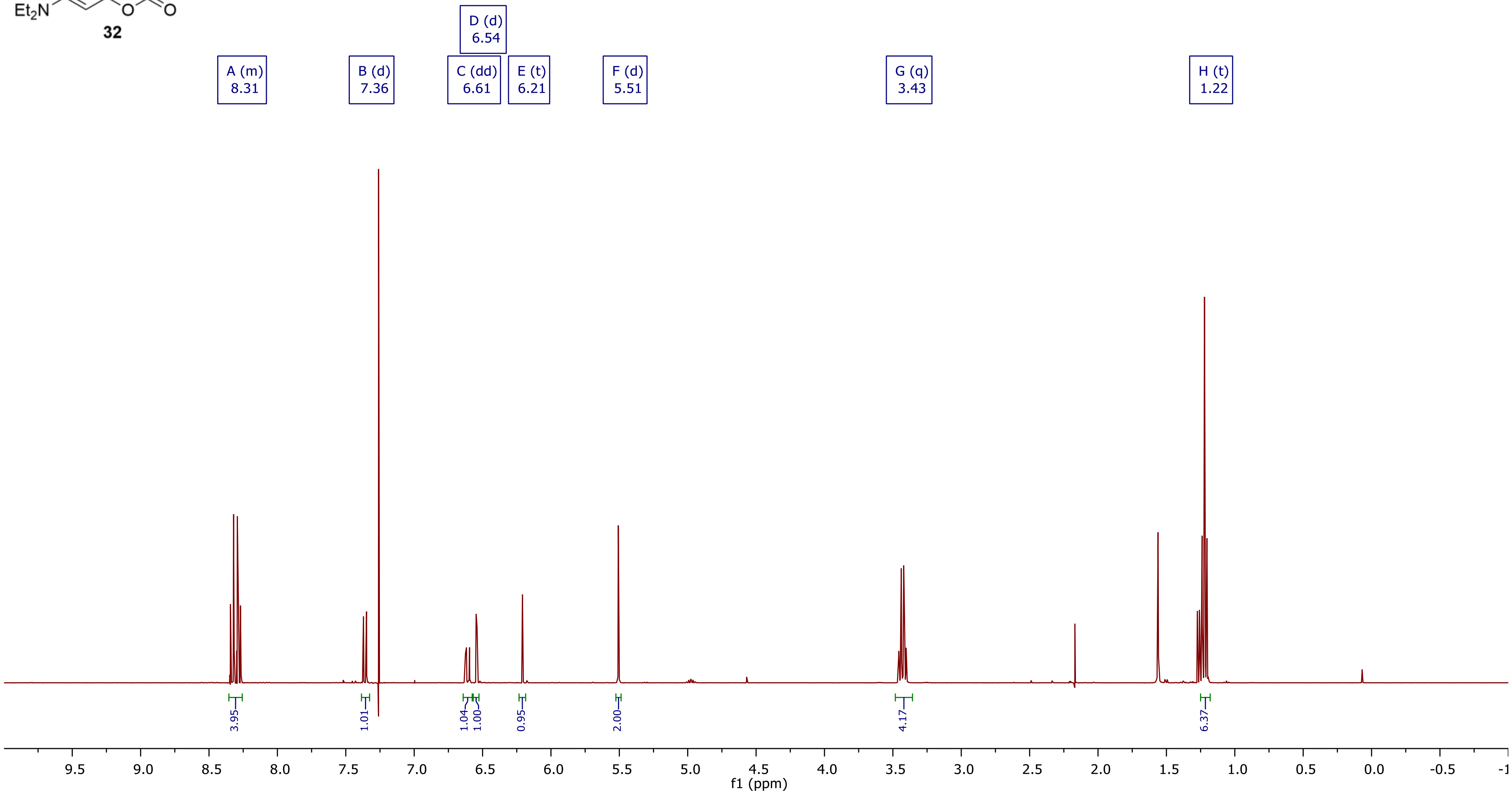

13C-NMR (101 MHz, CDCl3)

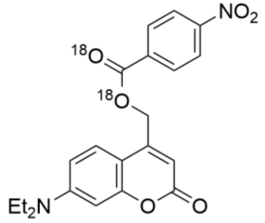

32

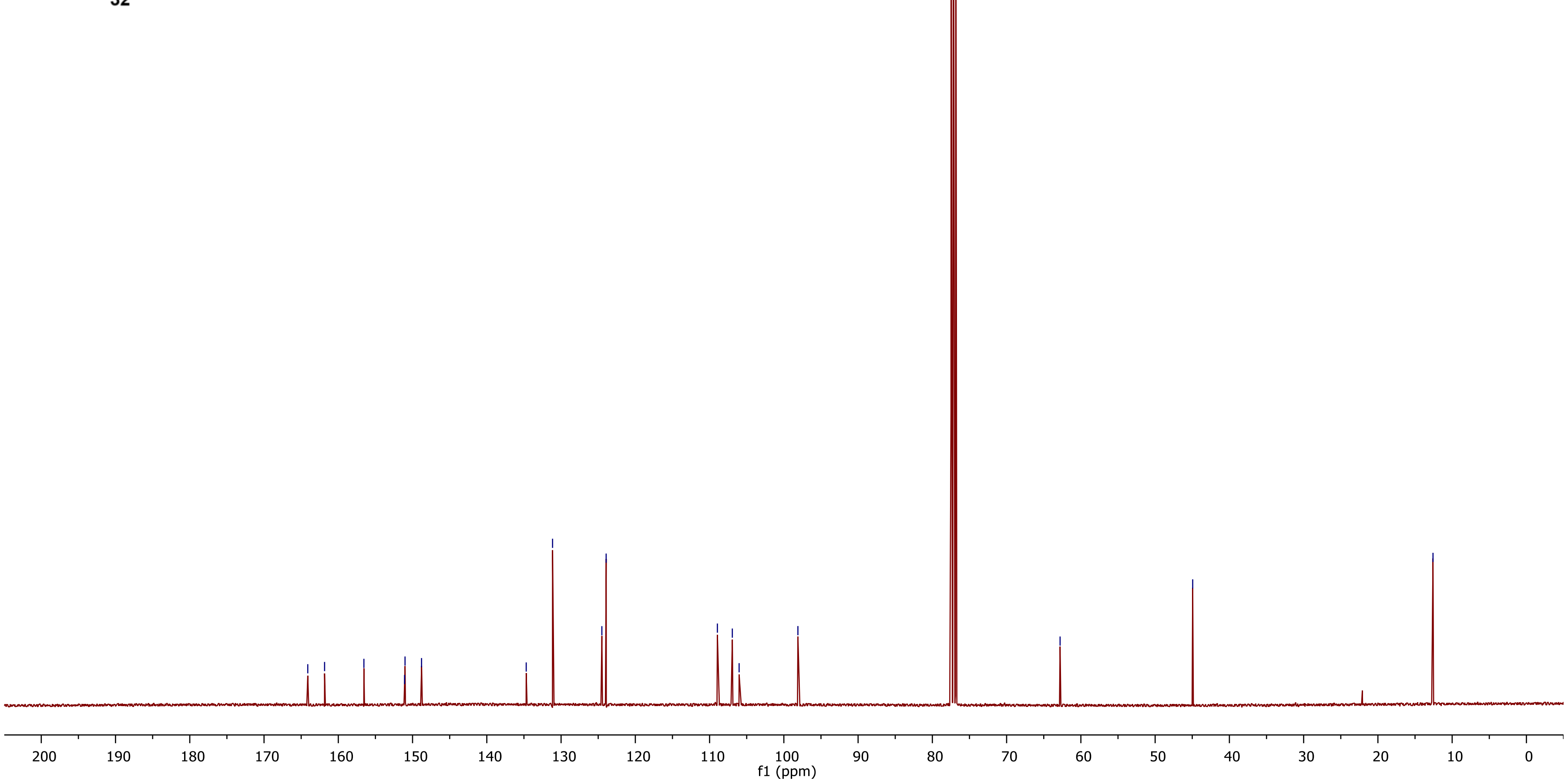

1H-NMR (400 MHz, CDCl3)

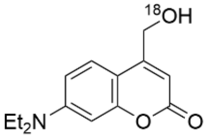

3

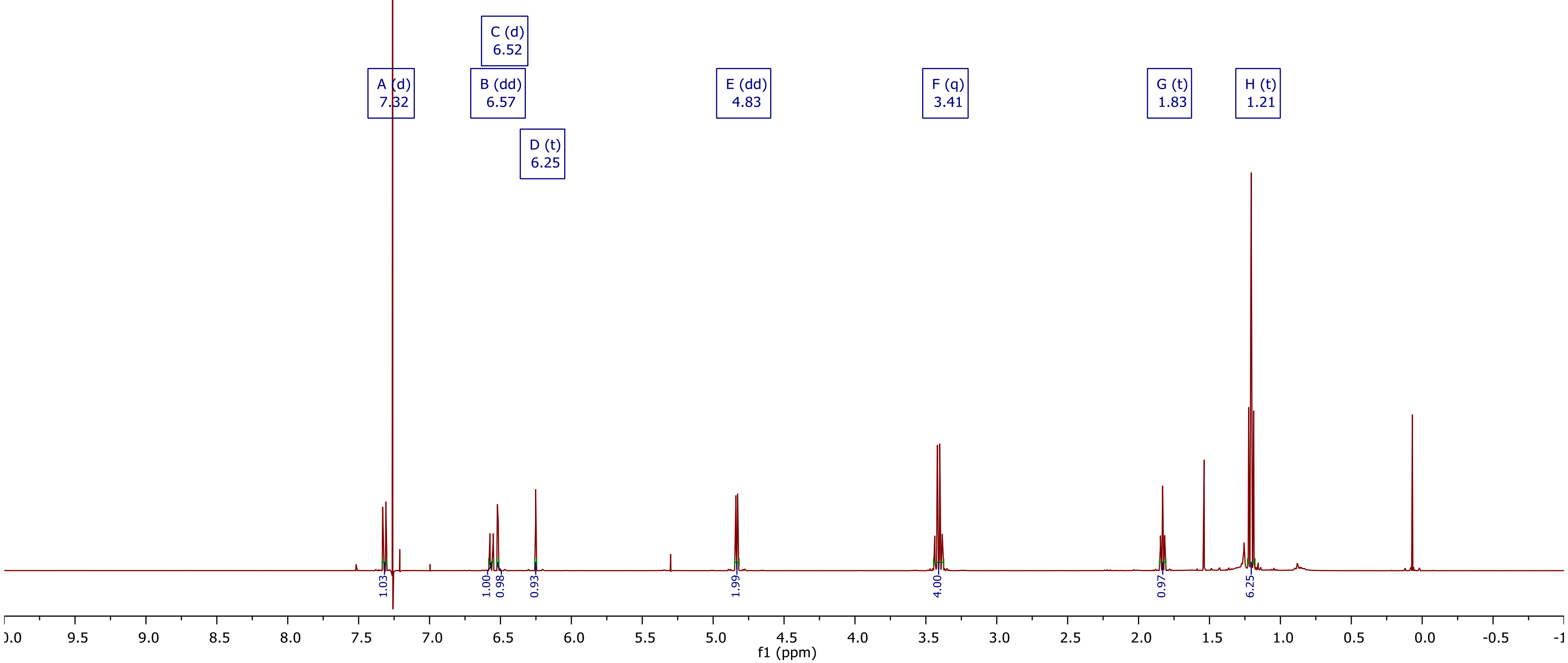

13C-NMR (101 MHz, CDCl3)

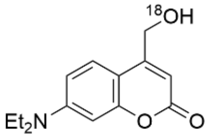

3

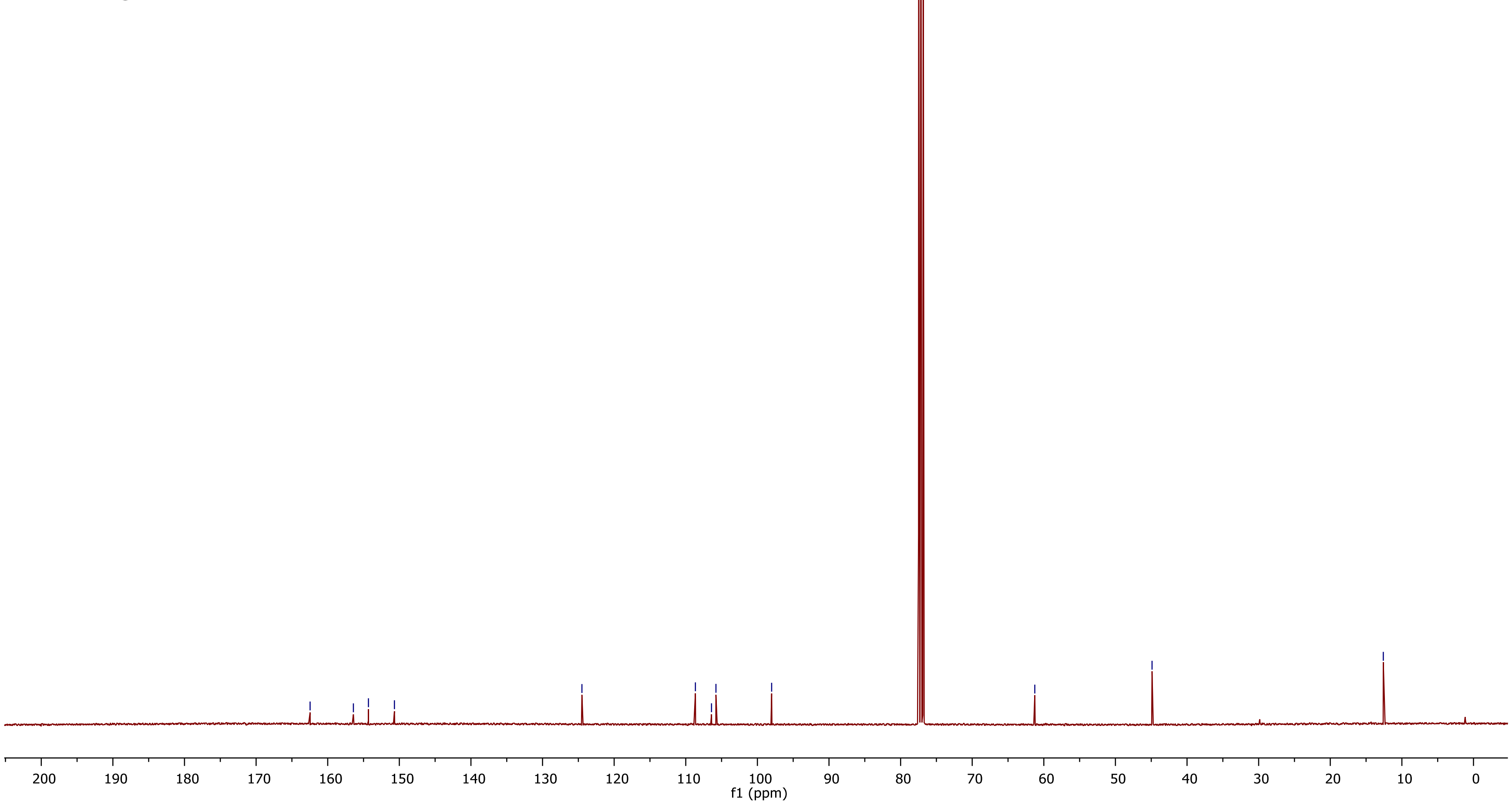

1H-NMR (400 MHz, CDCl3)

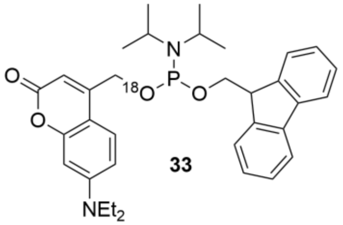

33

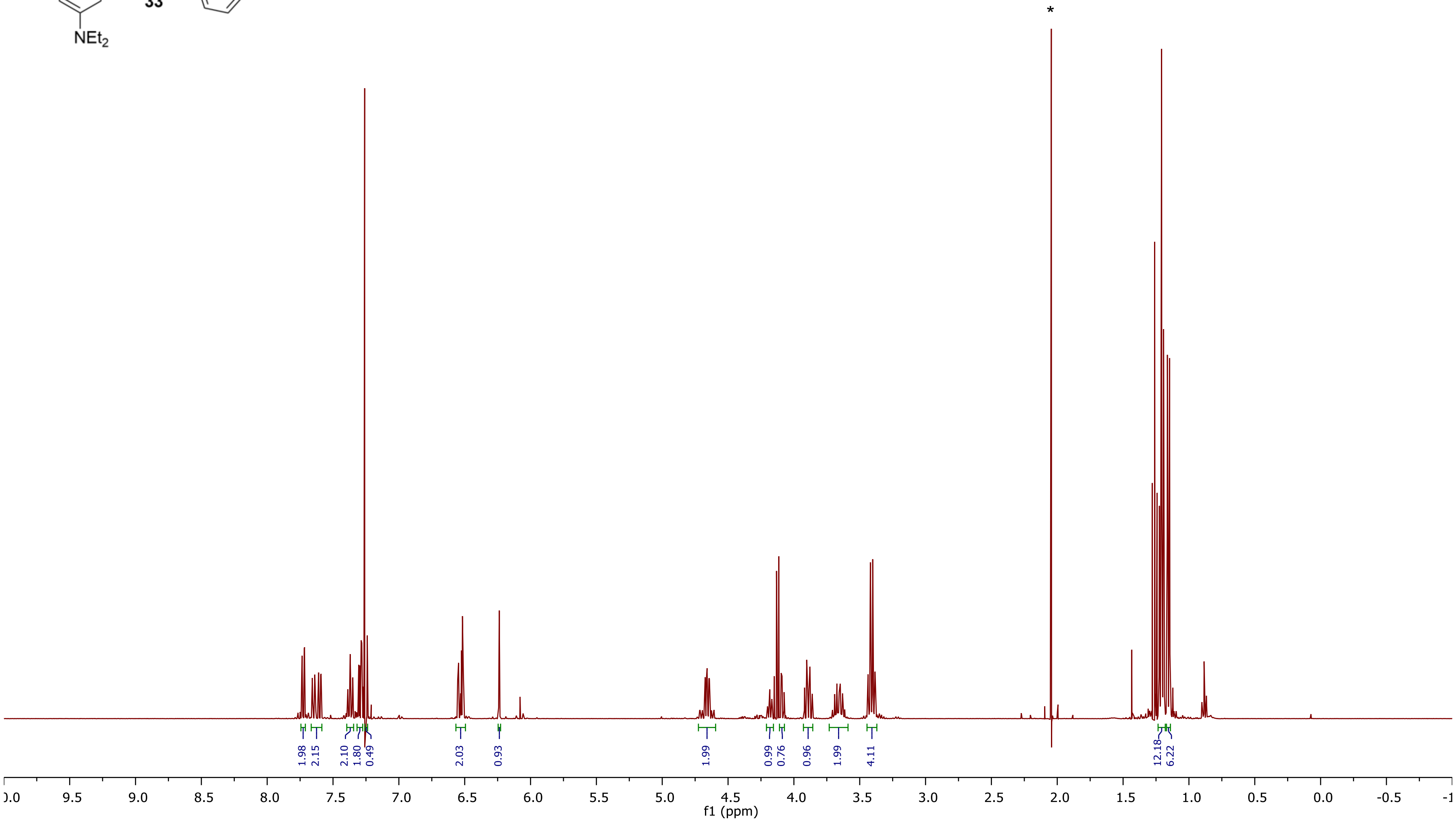

Residual amounts of EtOAc are marked with asterisks (*).

31P{1H}-NMR (162 MHz, CDCl3)

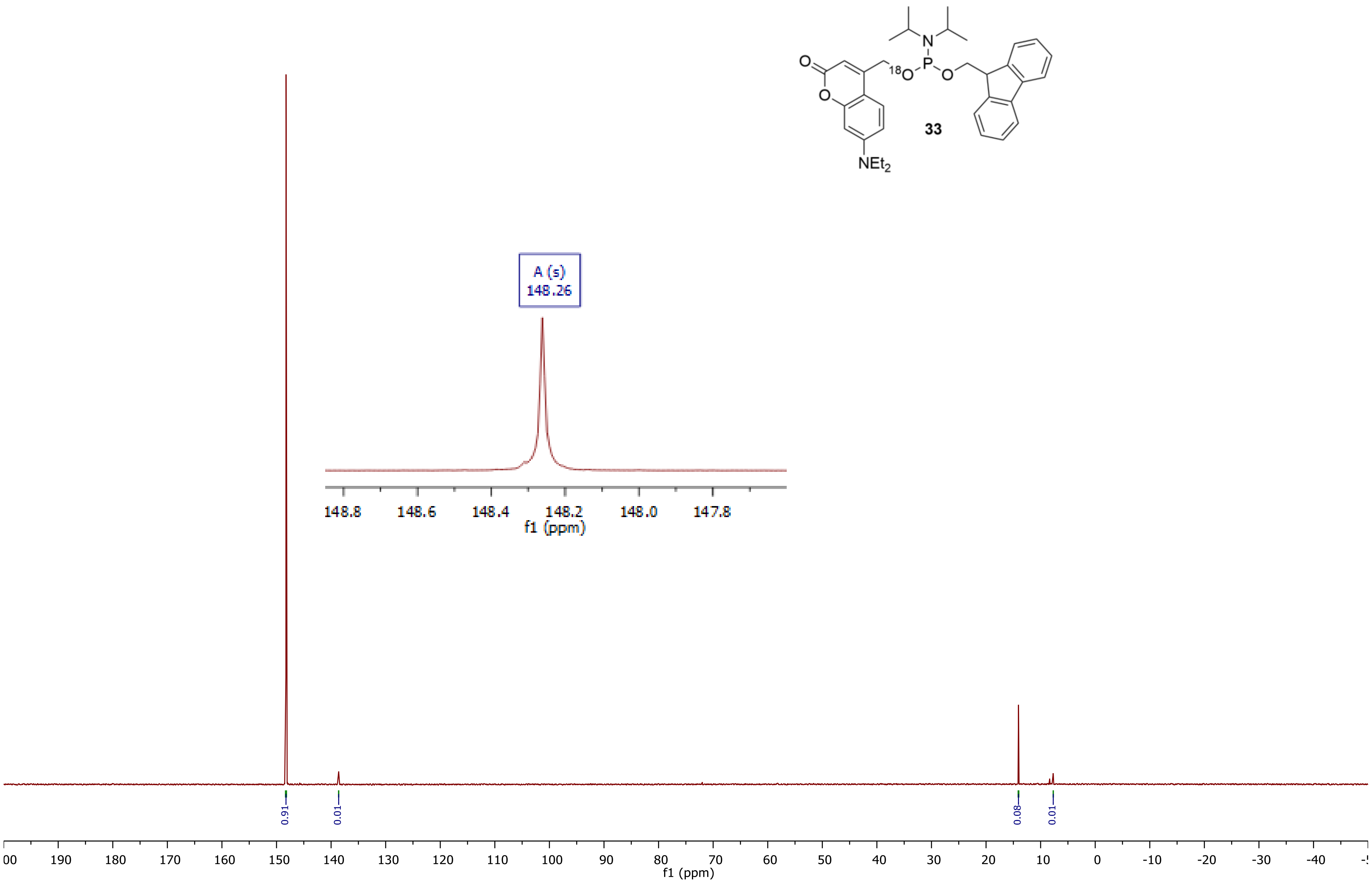

31P-NMR (162 MHz, CDCl3)

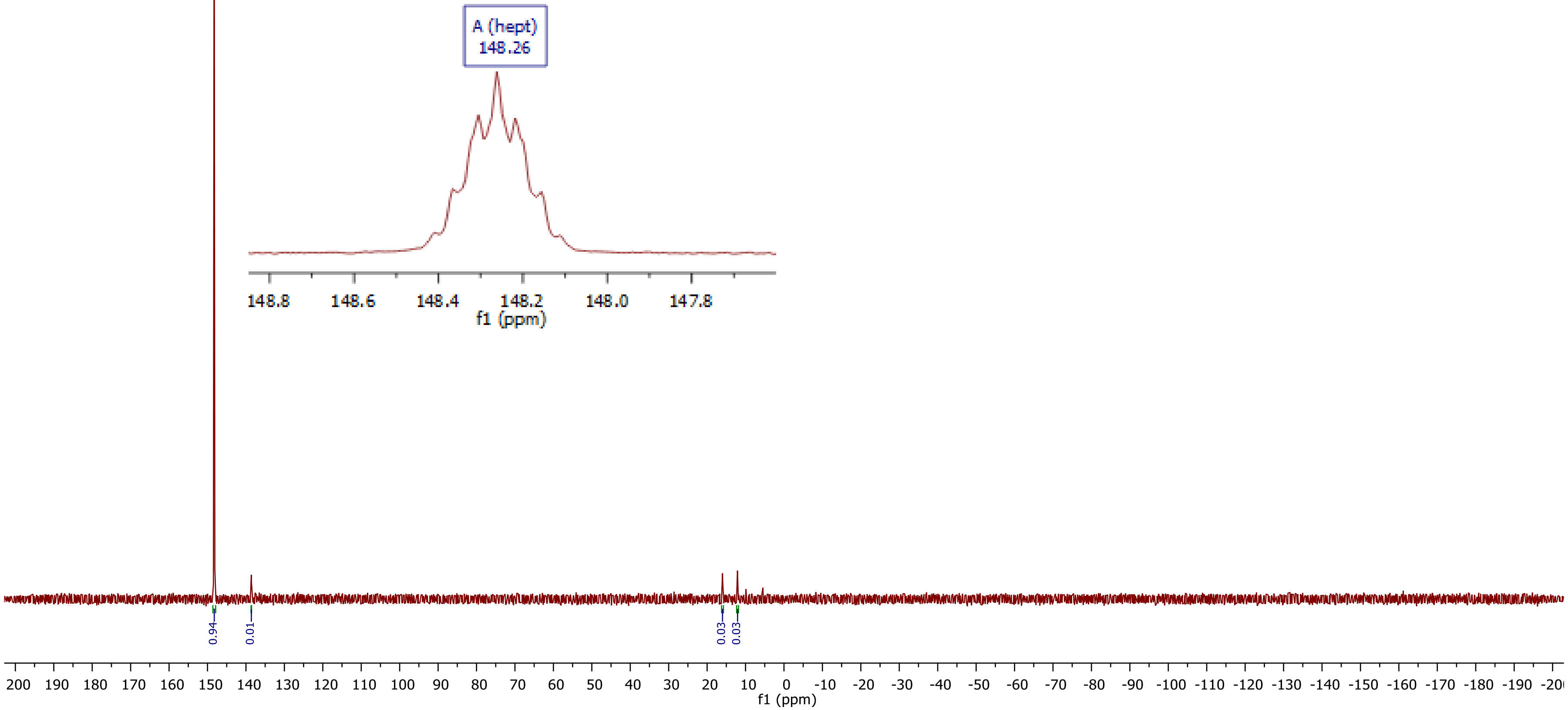

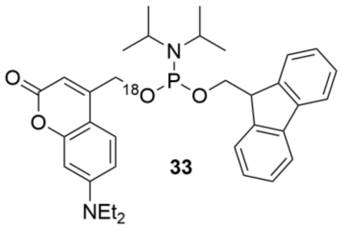

33

13C-NMR (101 MHz, CDCl3)

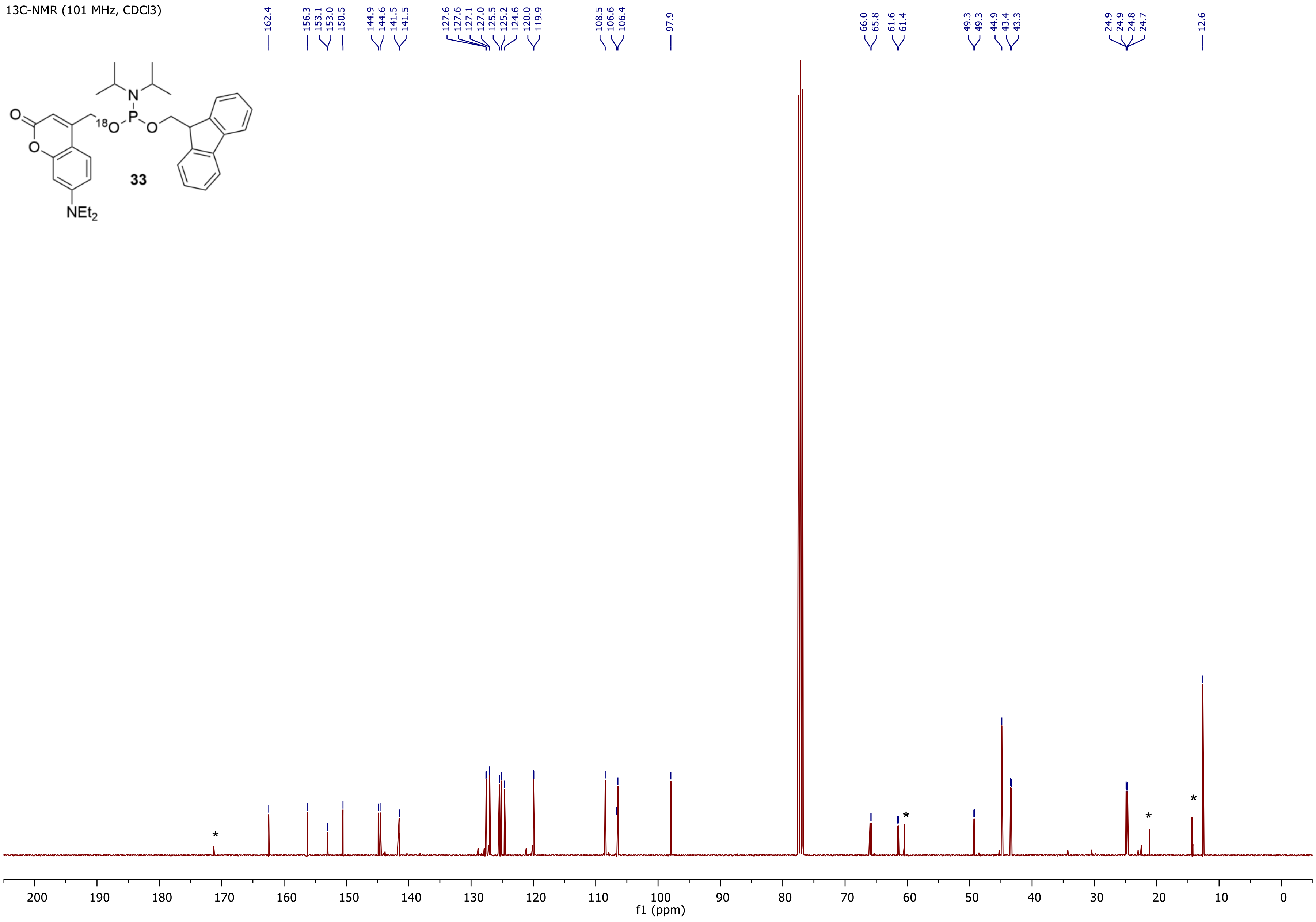

Residual amounts of EtOAc are marked with asterisks (*).

1H-NMR (400 MHz, D2O)

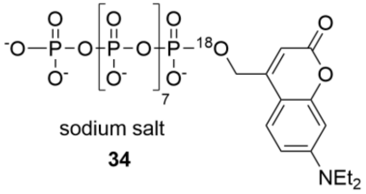

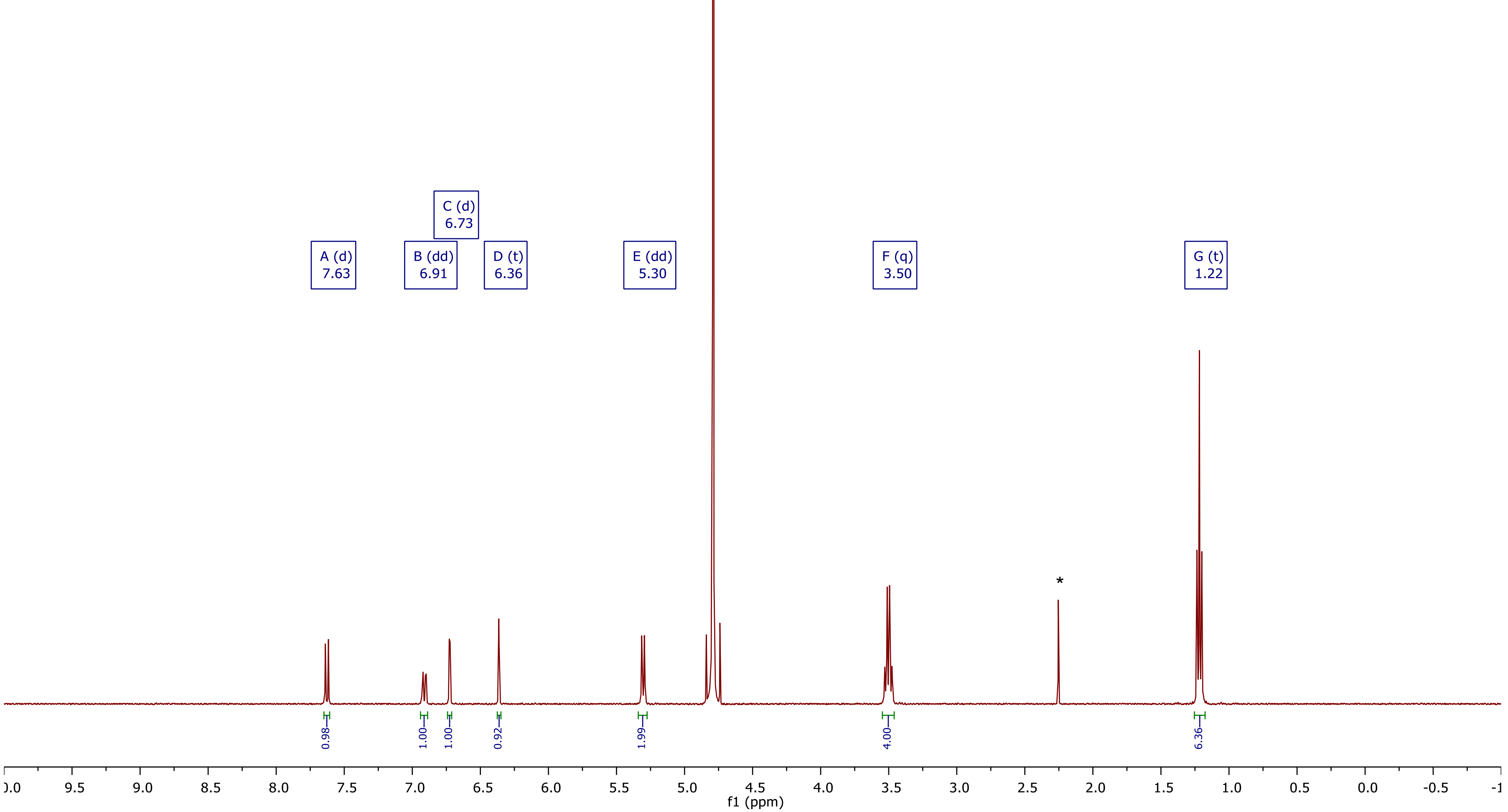

Residual amounts of acetone are marked with asterisks (*).

31P{1H}-NMR (162 MHz, D2O)

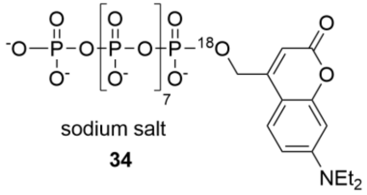

sodium salt
34

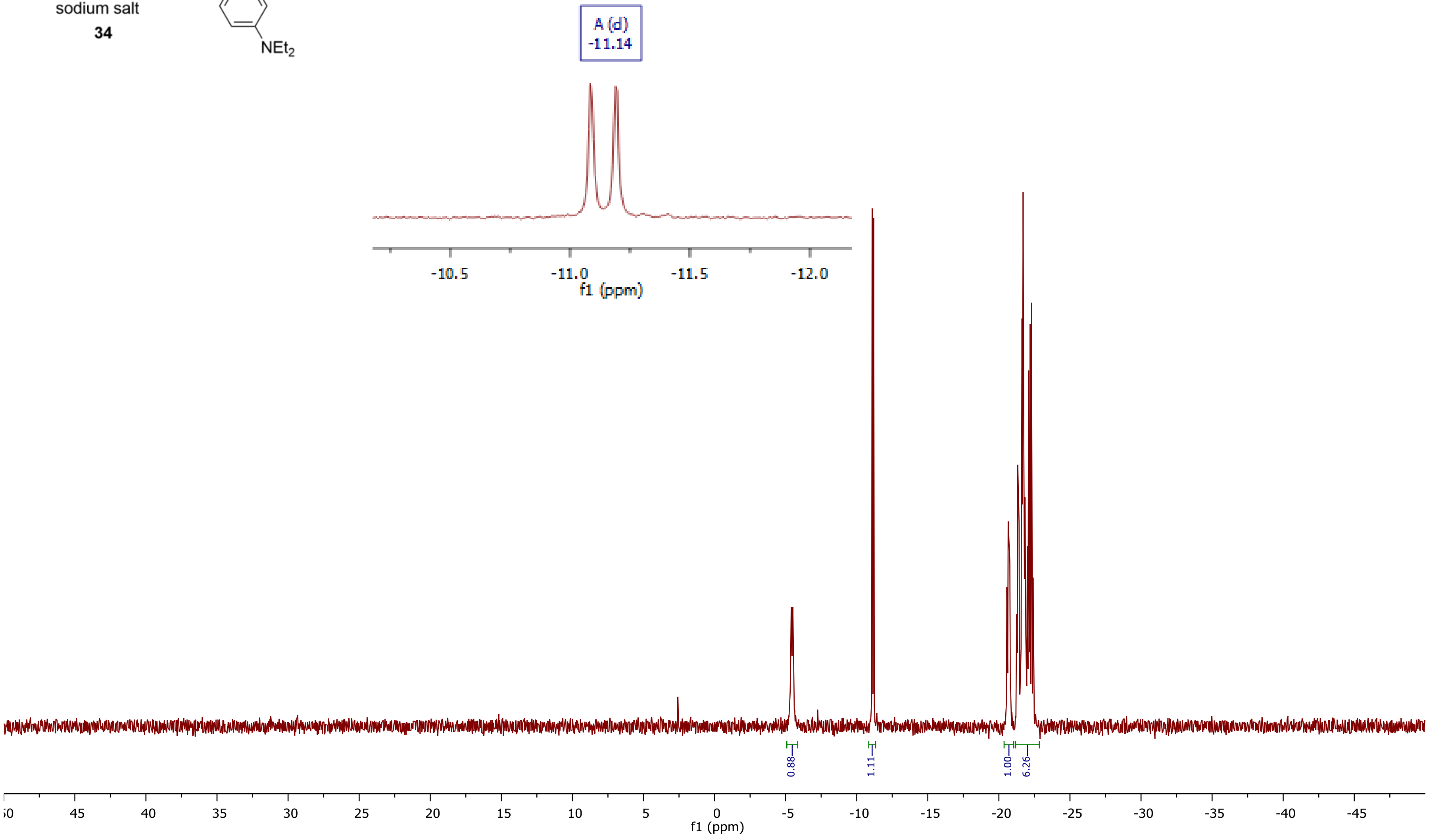

31P-NMR (162 MHz, D2O)

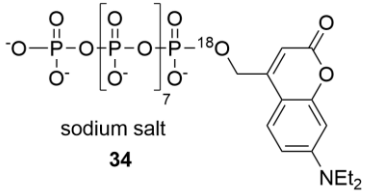

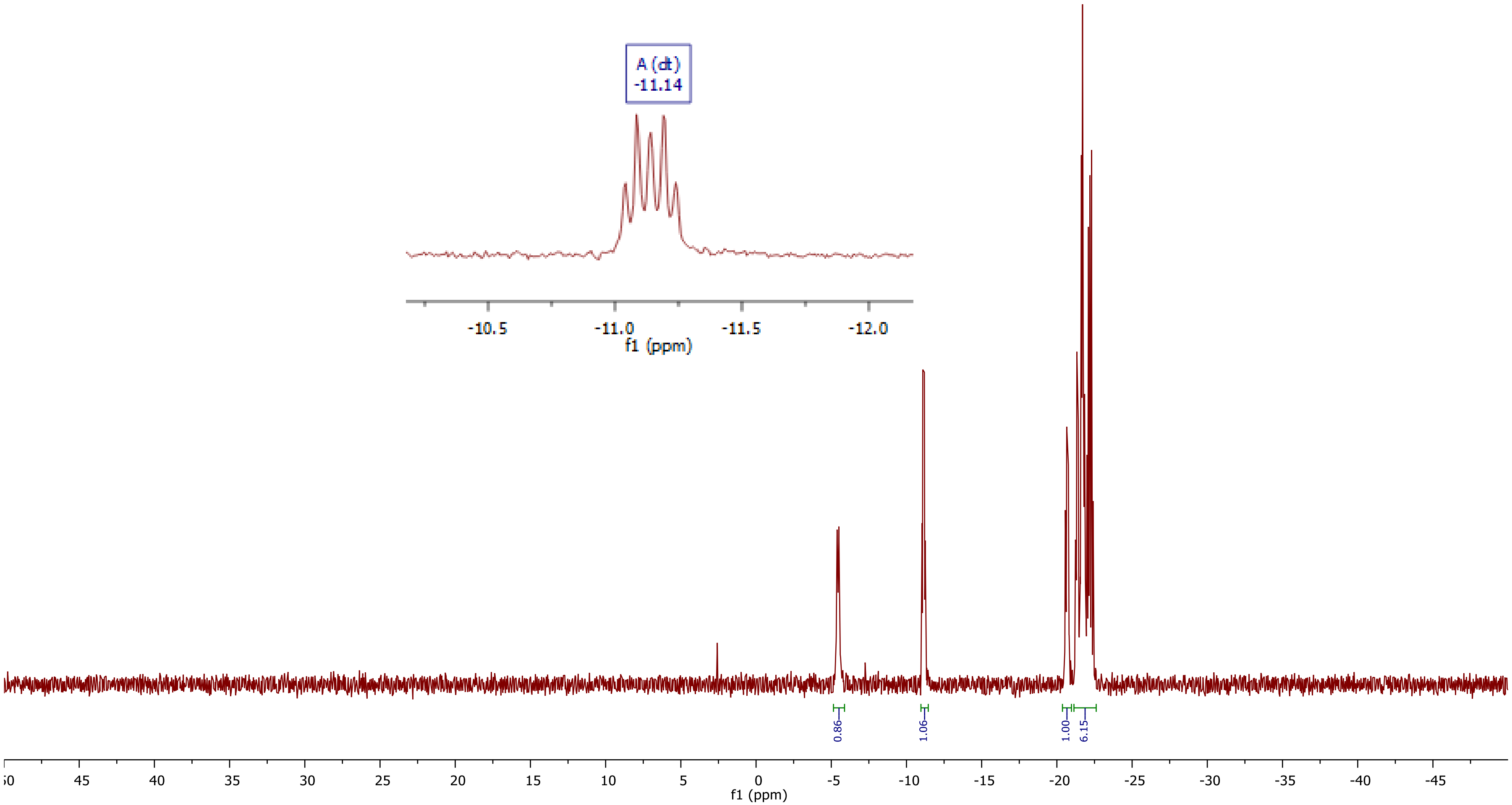

13C-NMR (101 MHz, D2O)

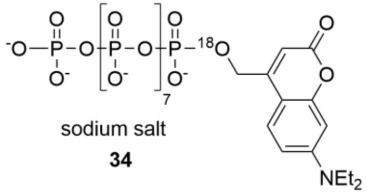

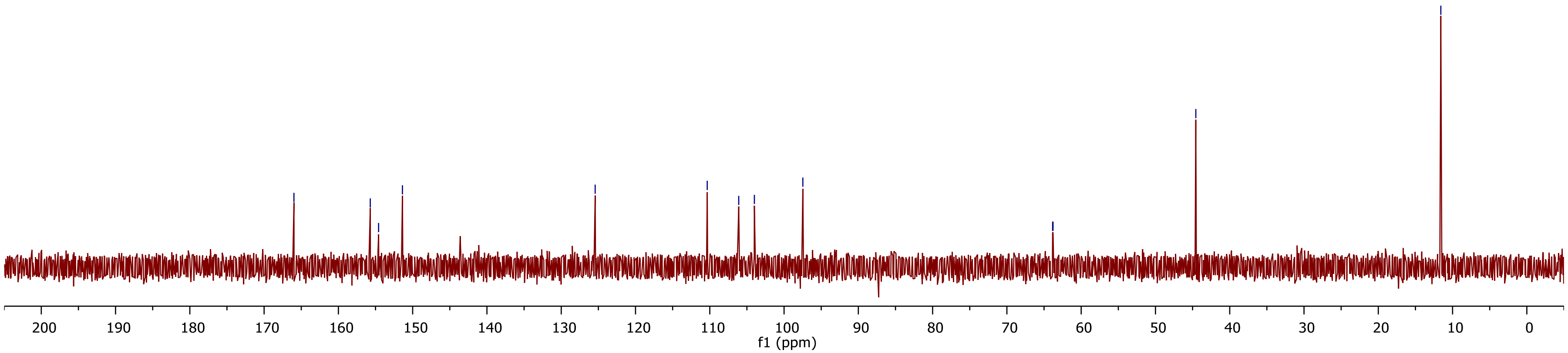

1H-NMR (400 MHz, D2O)

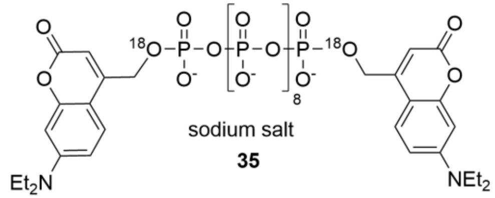

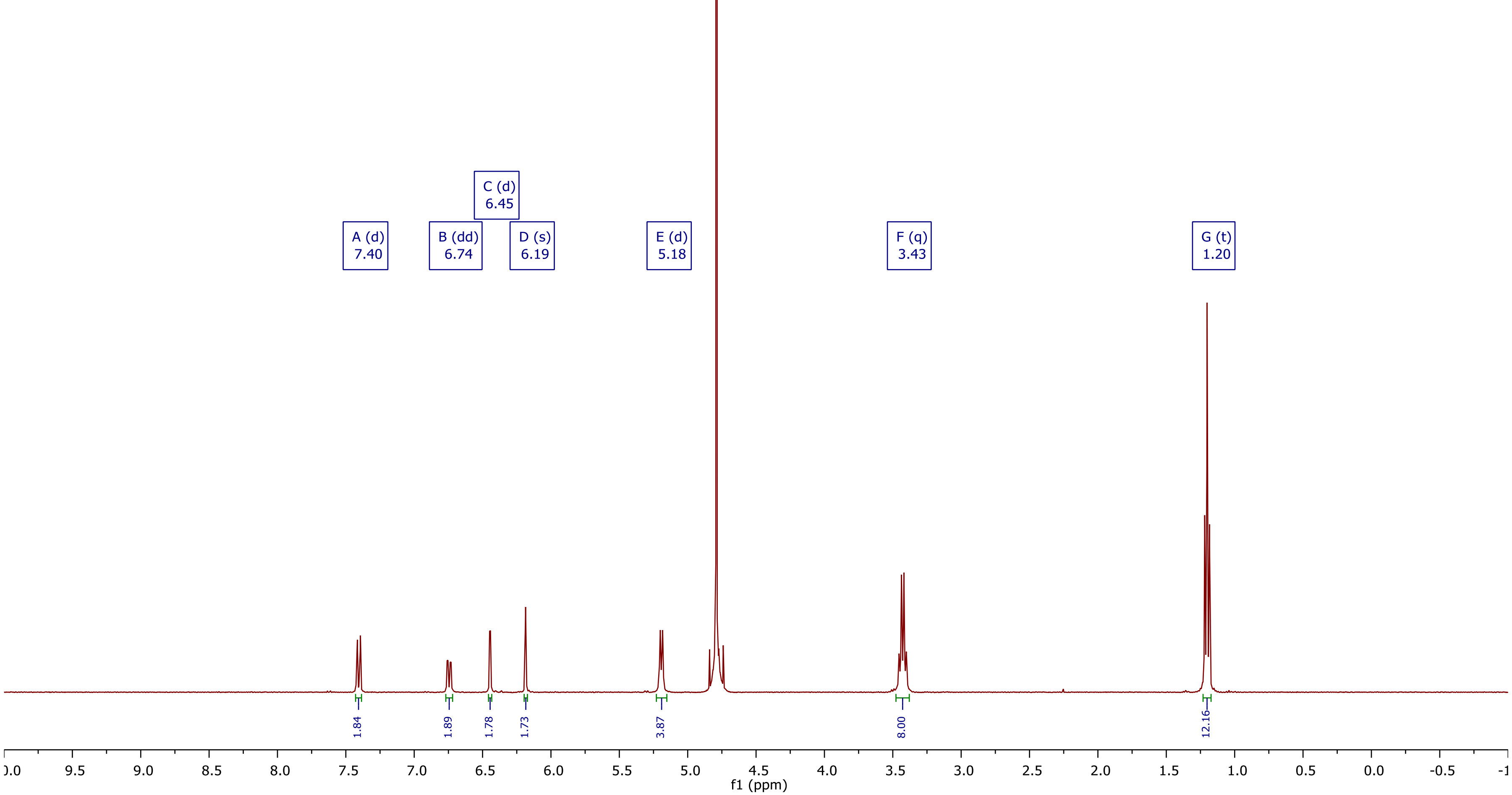

31P{1H}-NMR (162 MHz, D2O)

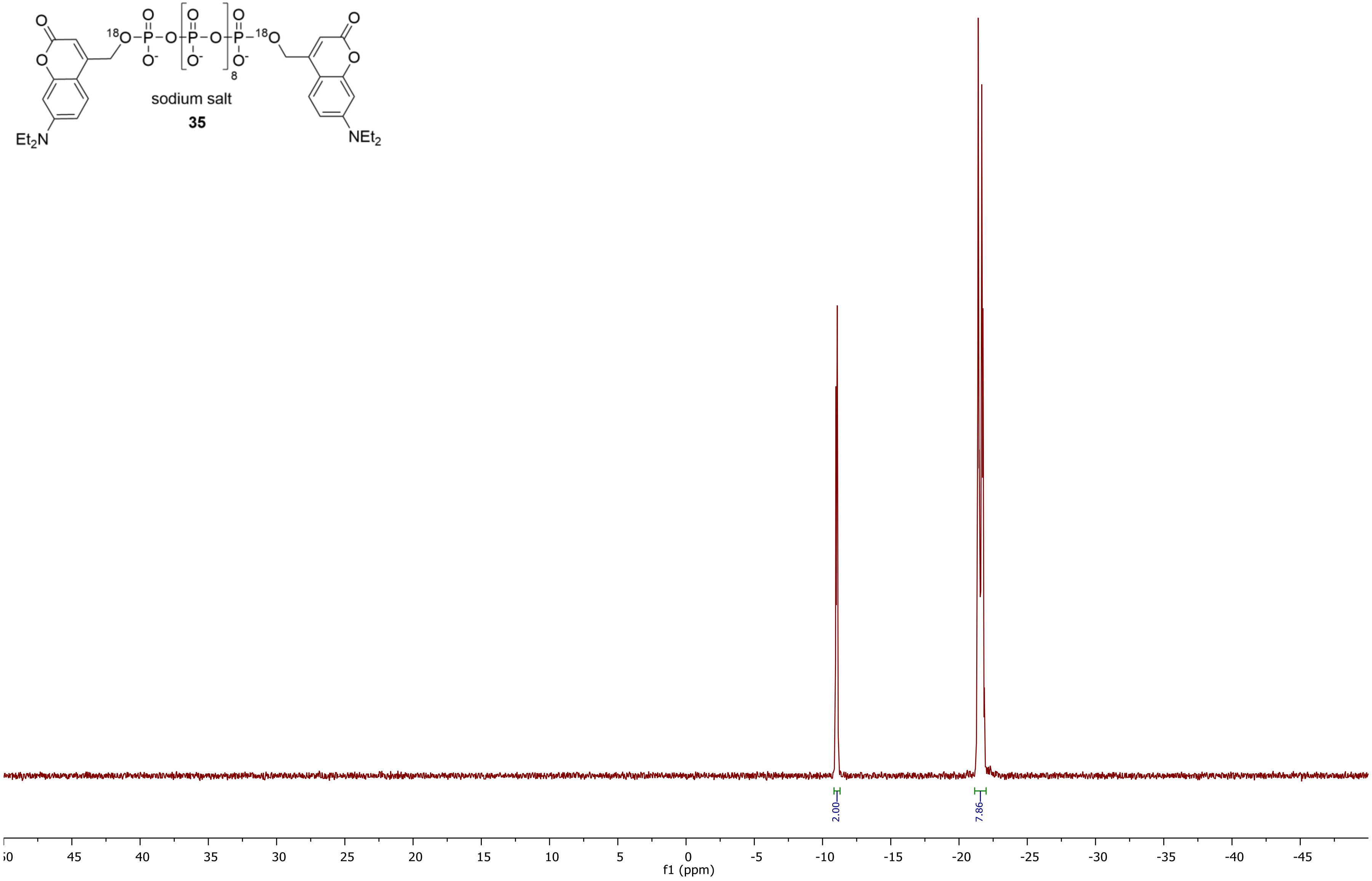

13C-NMR (101 MHz, D2O)

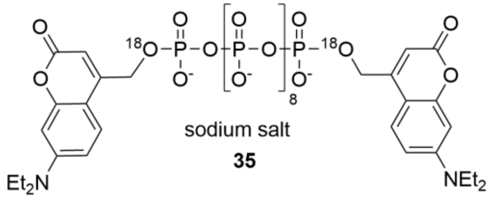

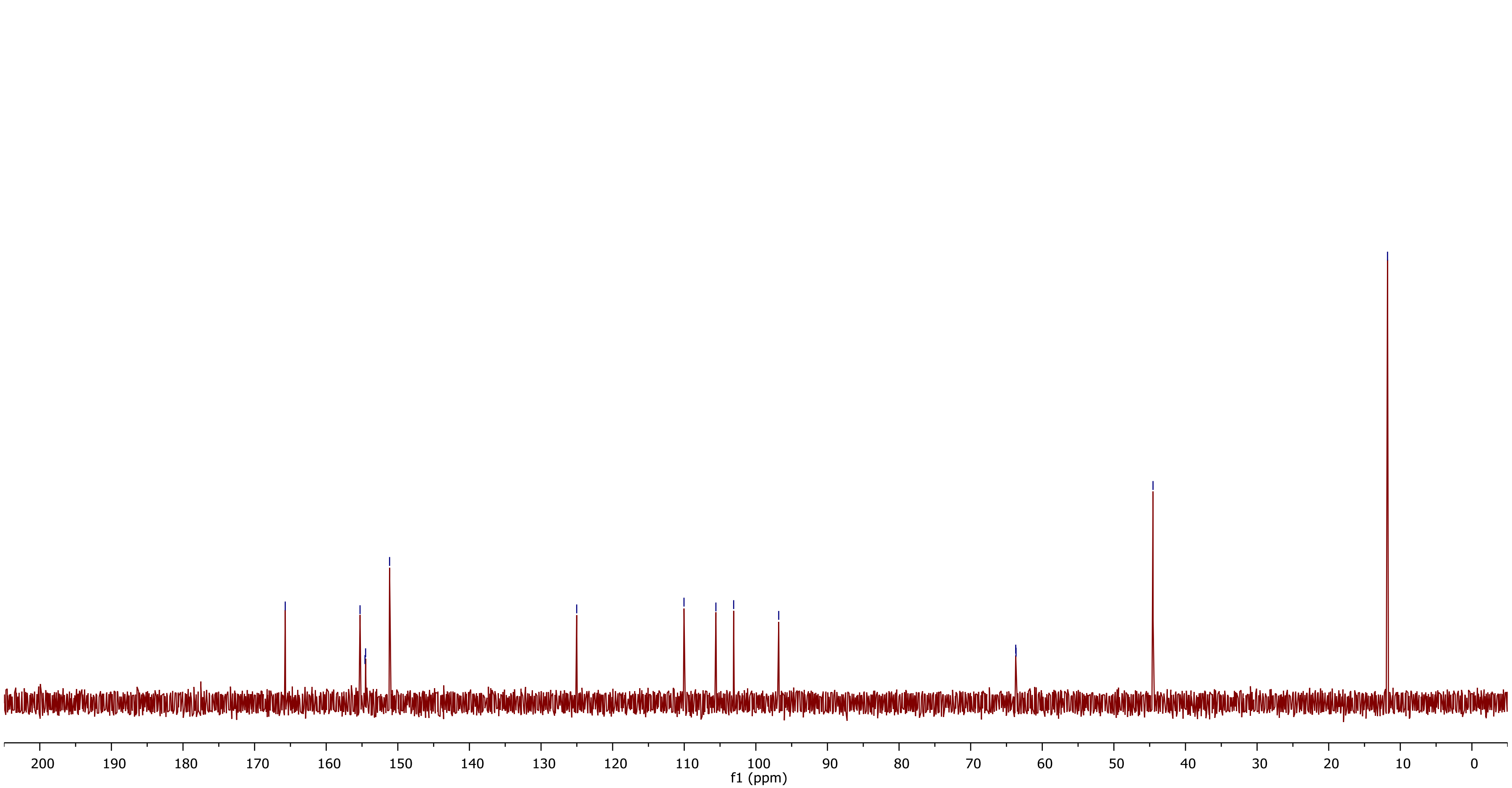

31P{1H}-NMR (162 MHz, D2O)

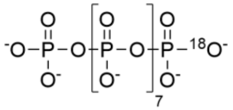

sodium salt
36

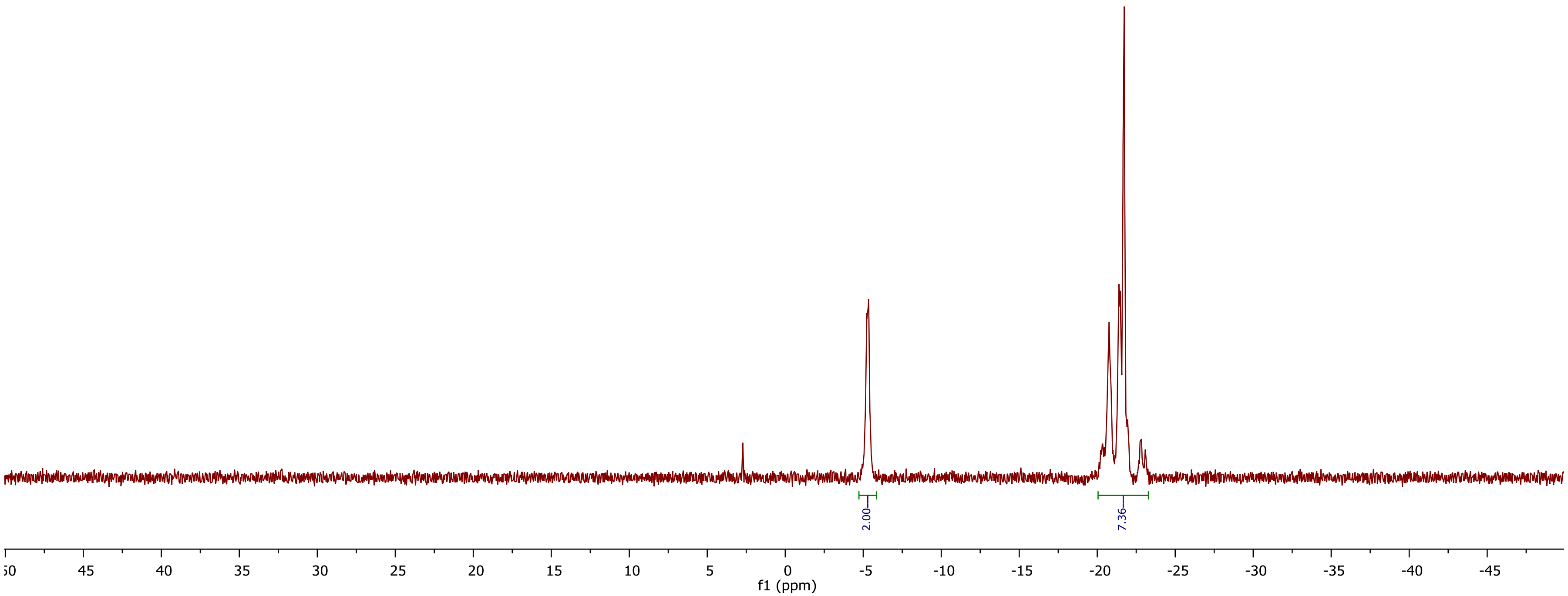

31P{1H}-NMR (162 MHz, D2O)

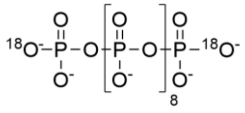

sodium salt

37

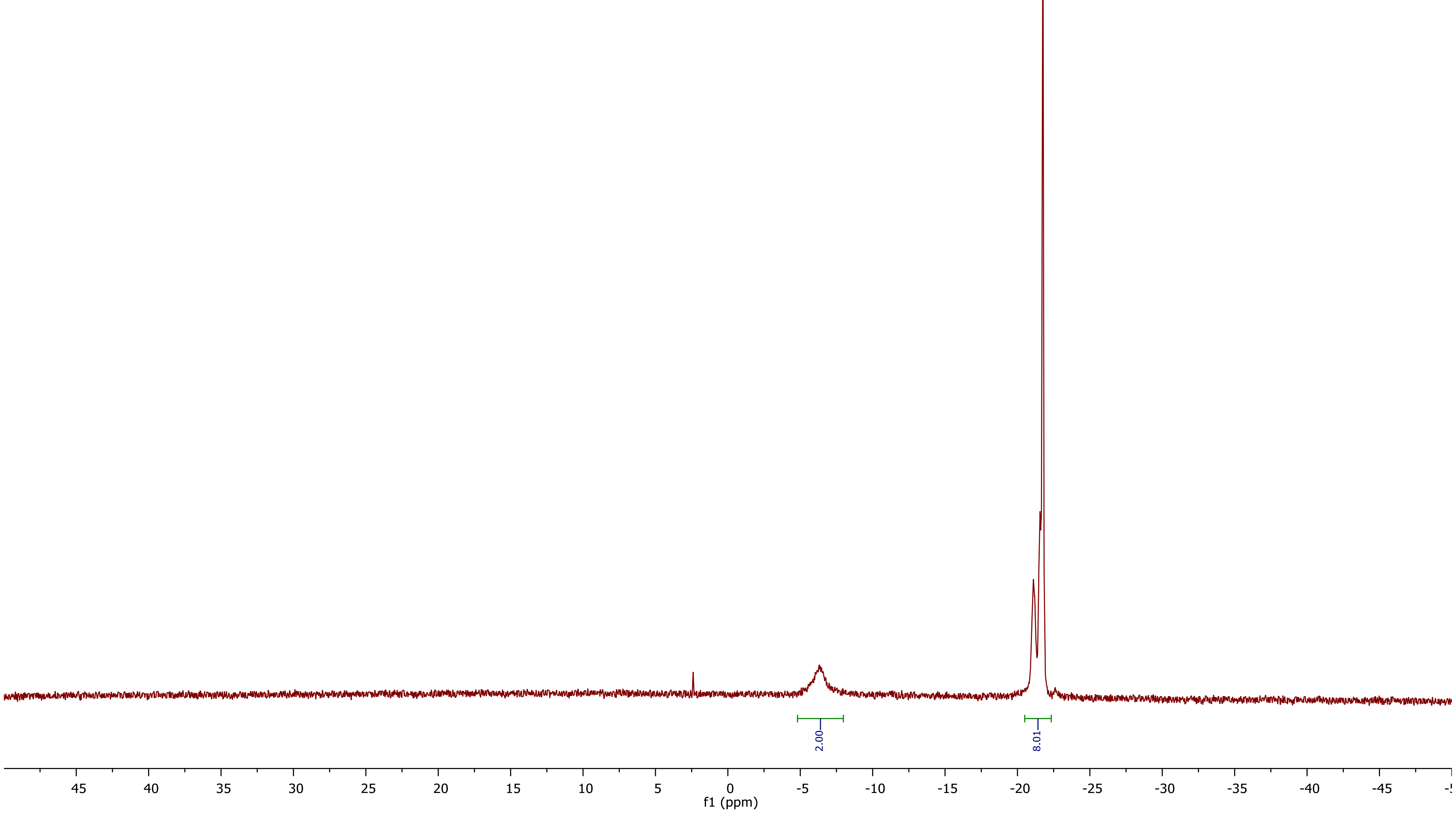

1H-NMR (400 MHz, CDCl3)

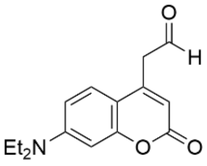

20

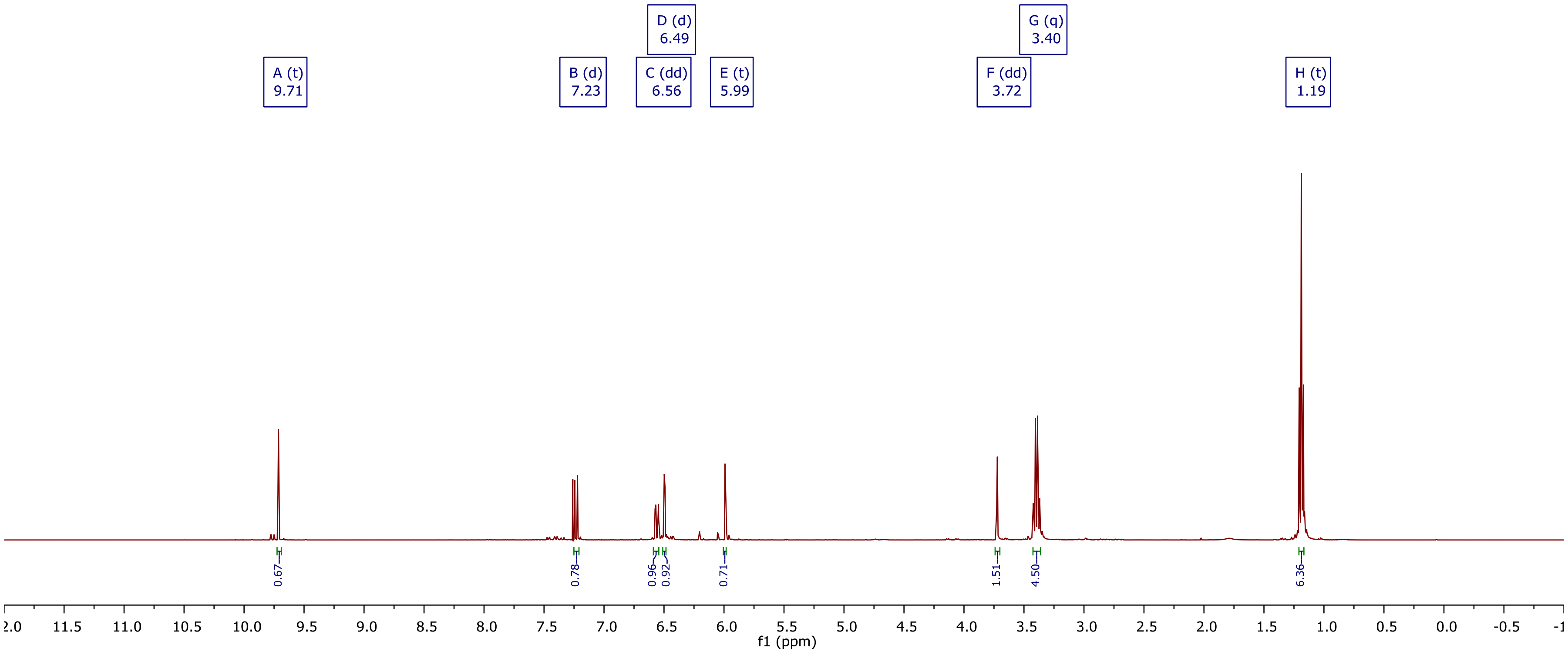

13C-NMR (101 MHz, CDCl3)

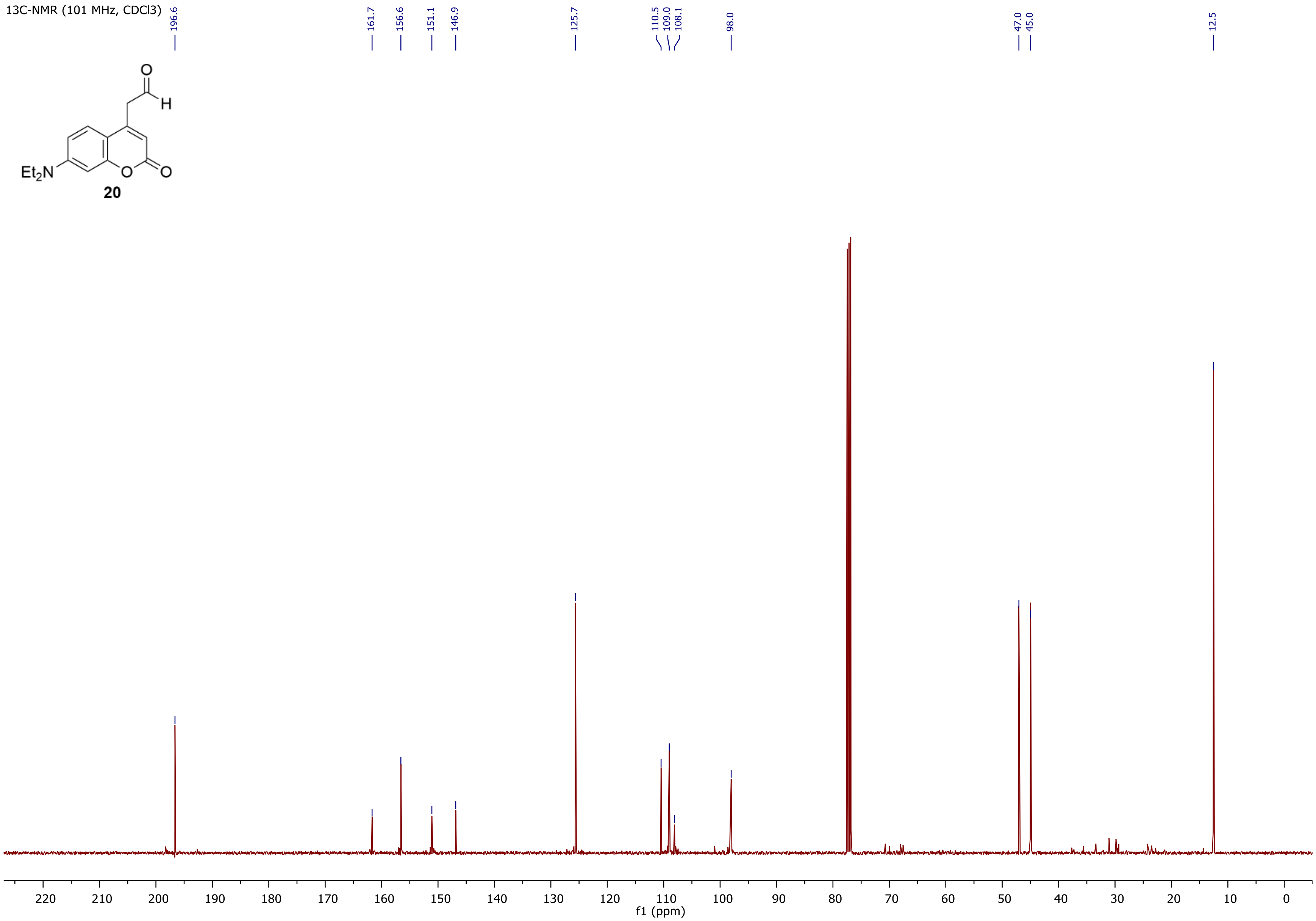

1H-NMR (400 MHz, CDCl3)

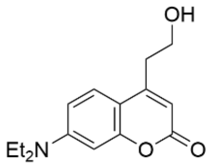

21

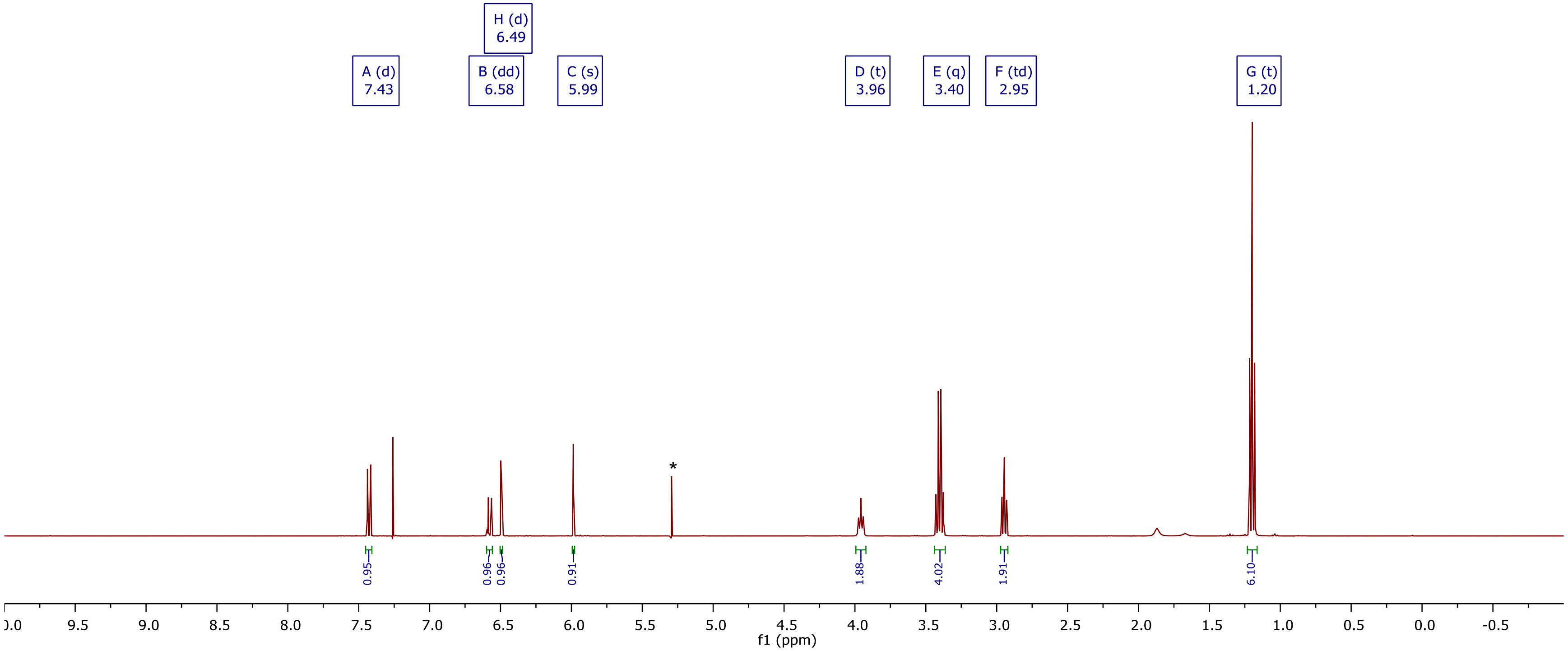

Residual amounts of DCM are marked with asterisks (*).

13C-NMR (101 MHz, CDCl3)

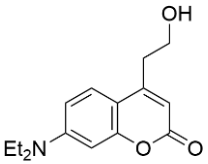

21

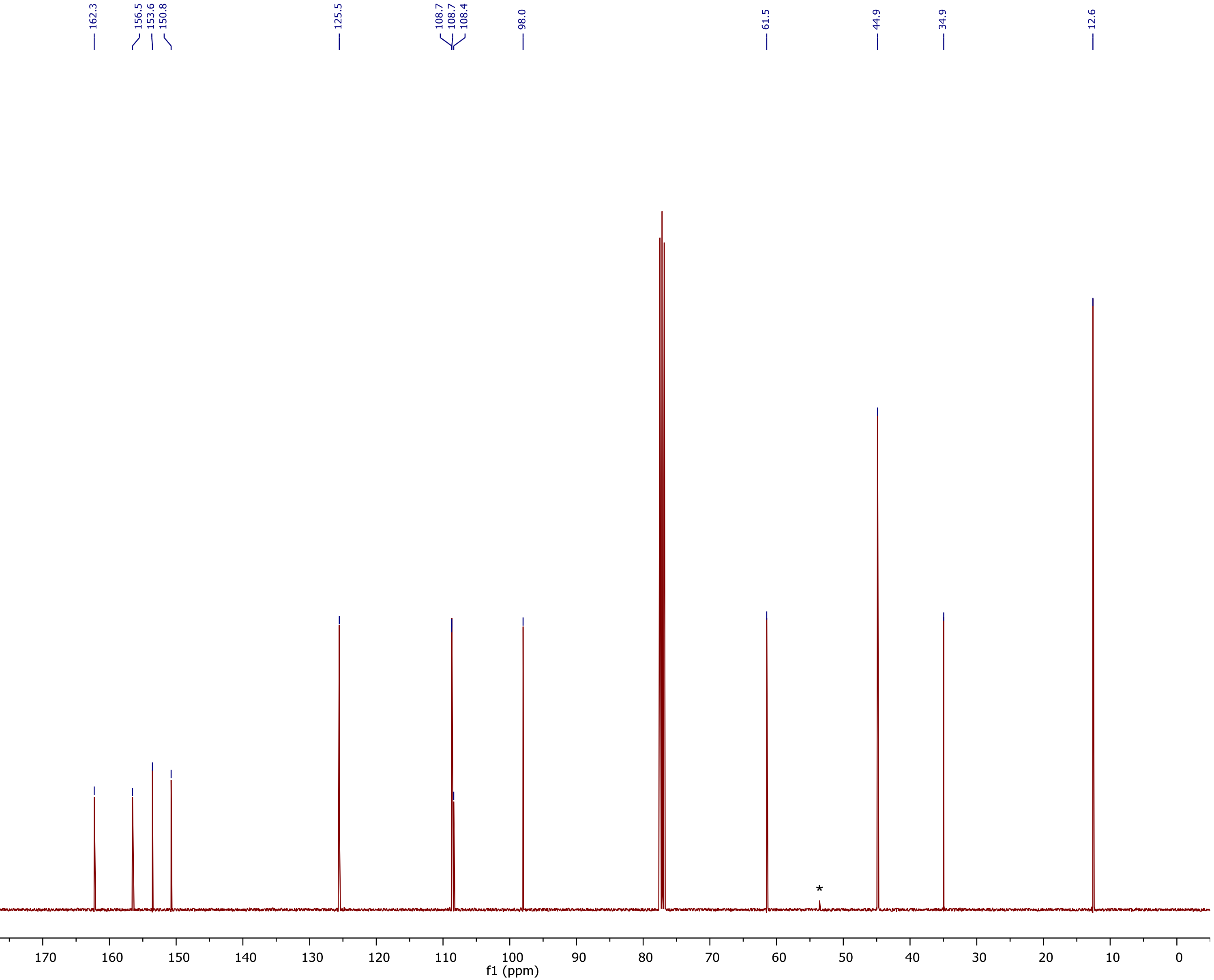

Residual amounts of DCM are marked with asterisks (*).

1H-NMR (400 MHz, CDCl3)

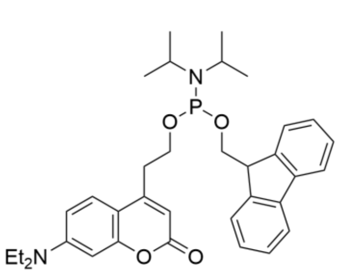

22

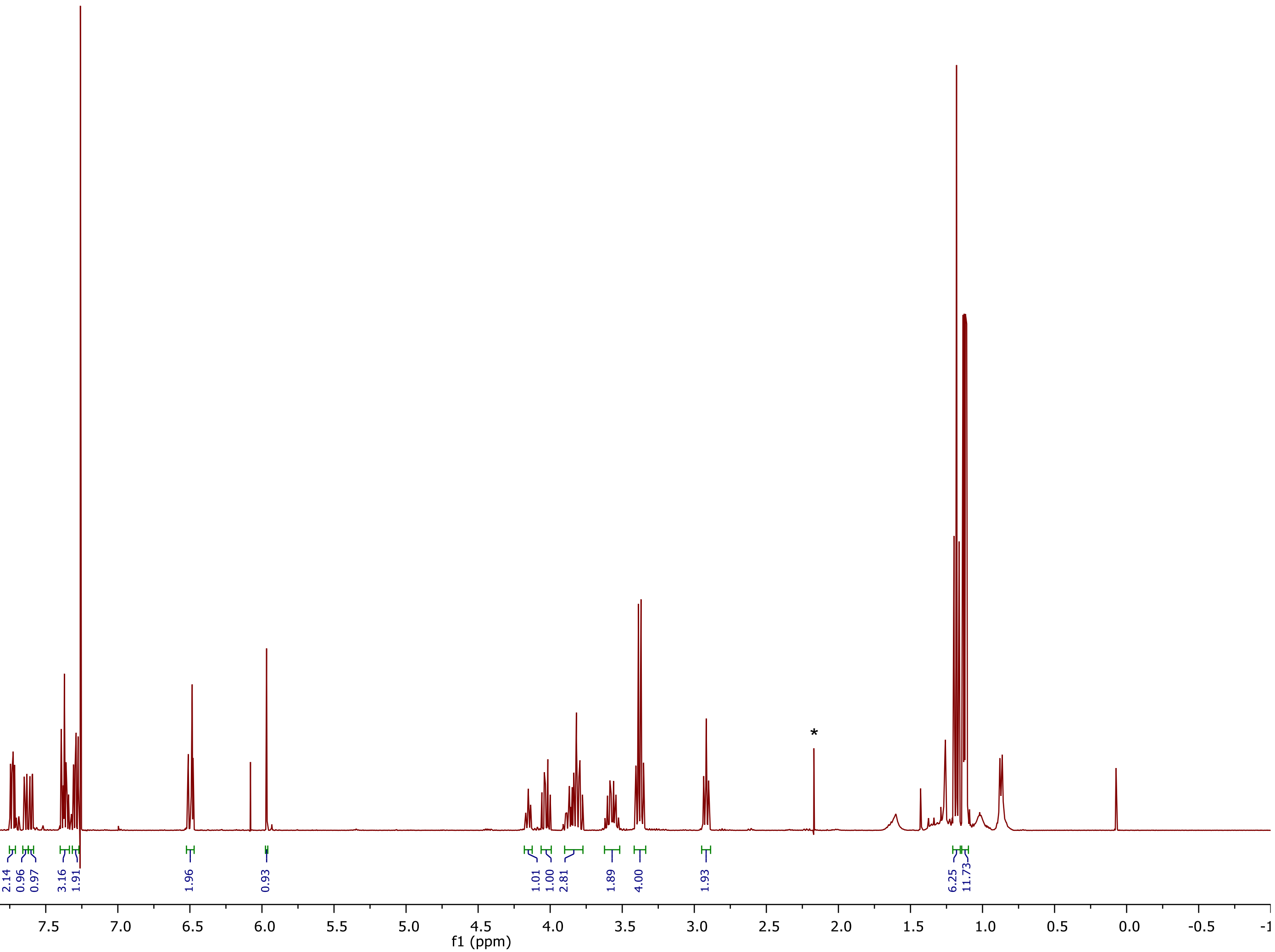

Residual amounts of acetone are marked with asterisks (*).

31P{1H}-NMR (162 MHz, CDCl3)

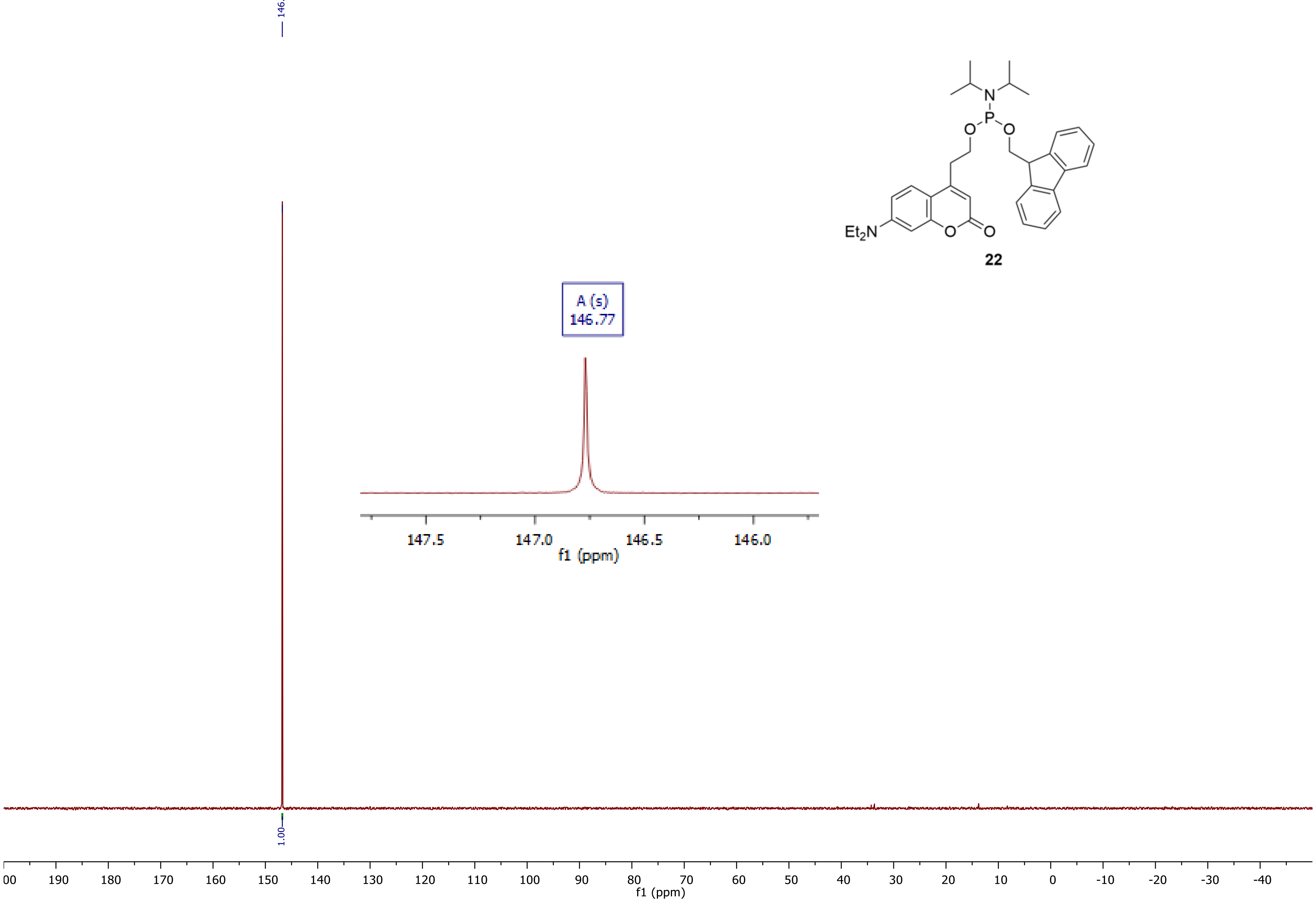

22

31P-NMR (162 MHz, CDCl3)

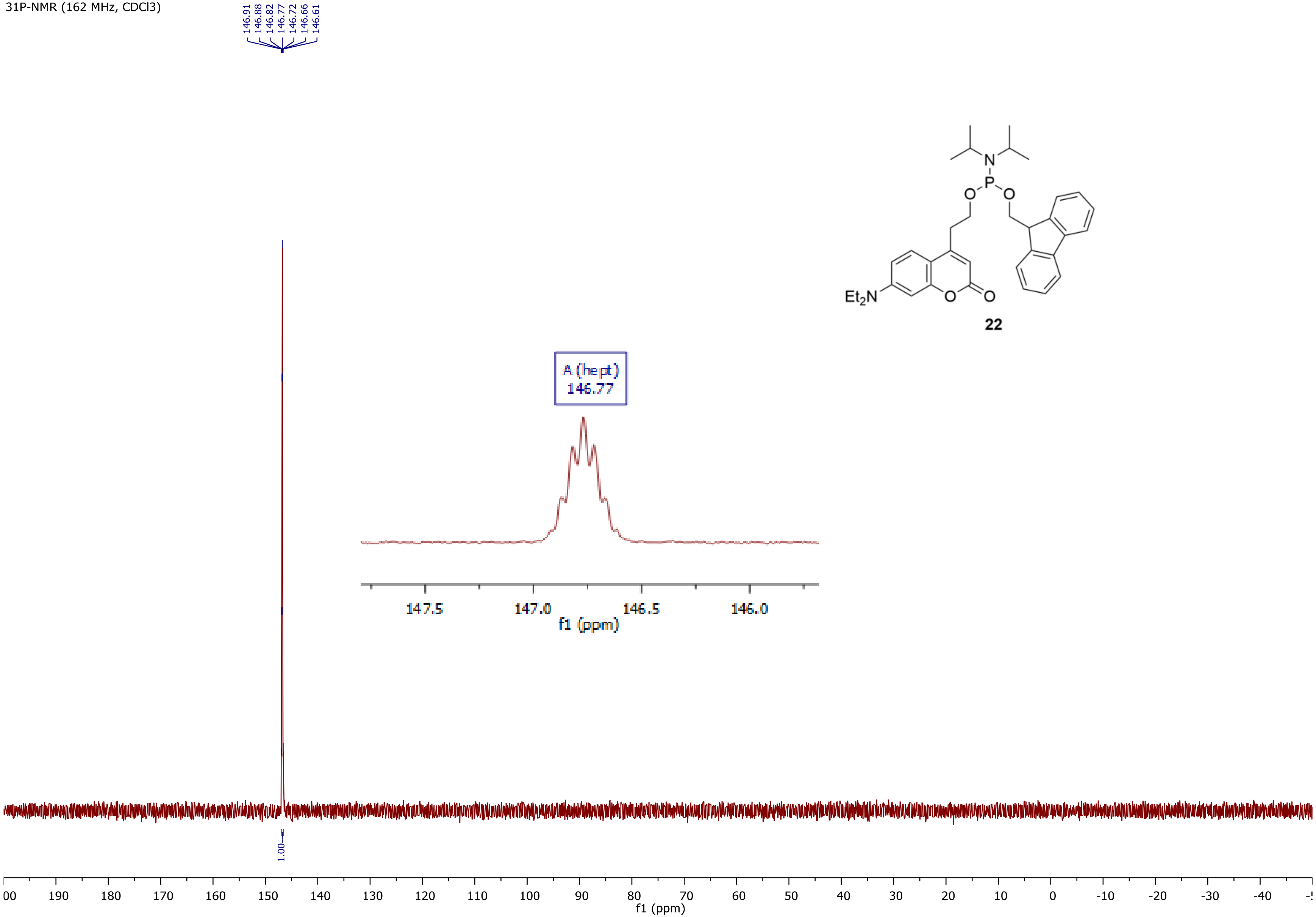

22

13C-NMR (101 MHz, CDCl3)

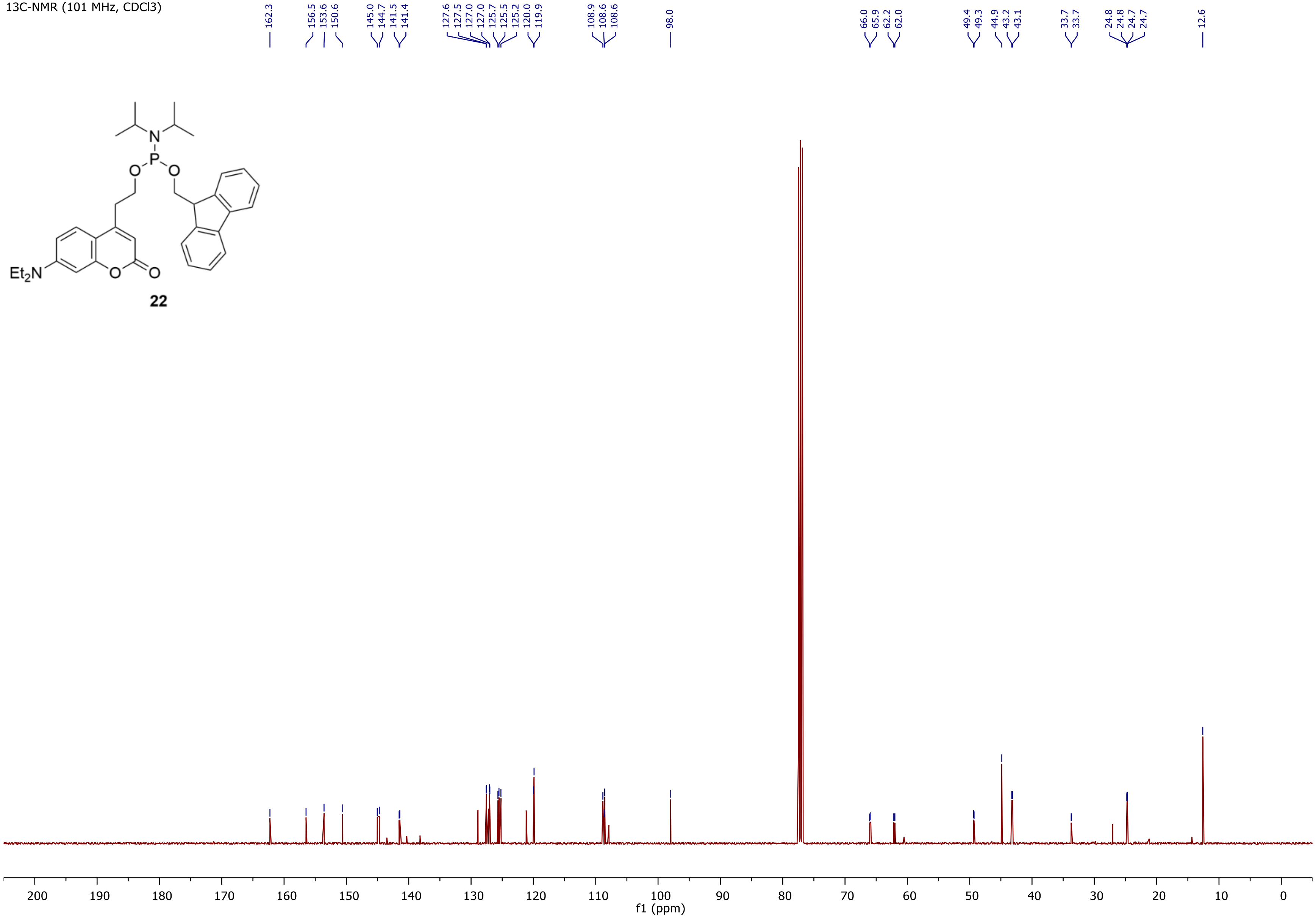

22

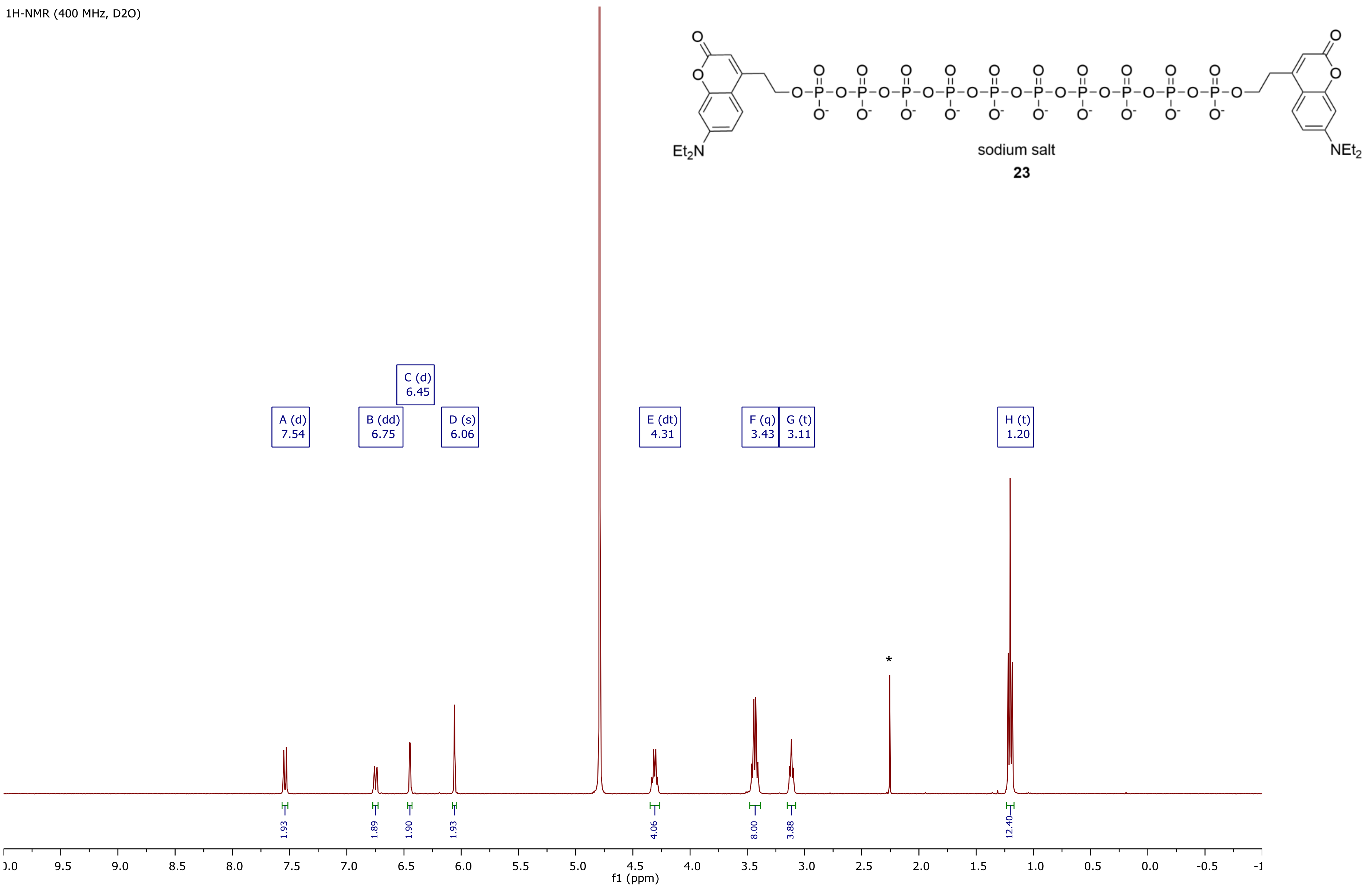

Residual amounts of acetone are marked with asterisks (*).

31P{1H}-NMR (162 MHz, D2O)

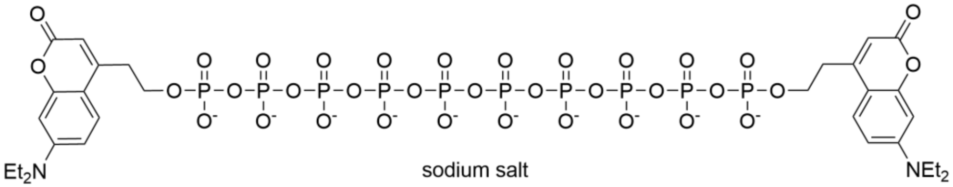

sodium salt

23

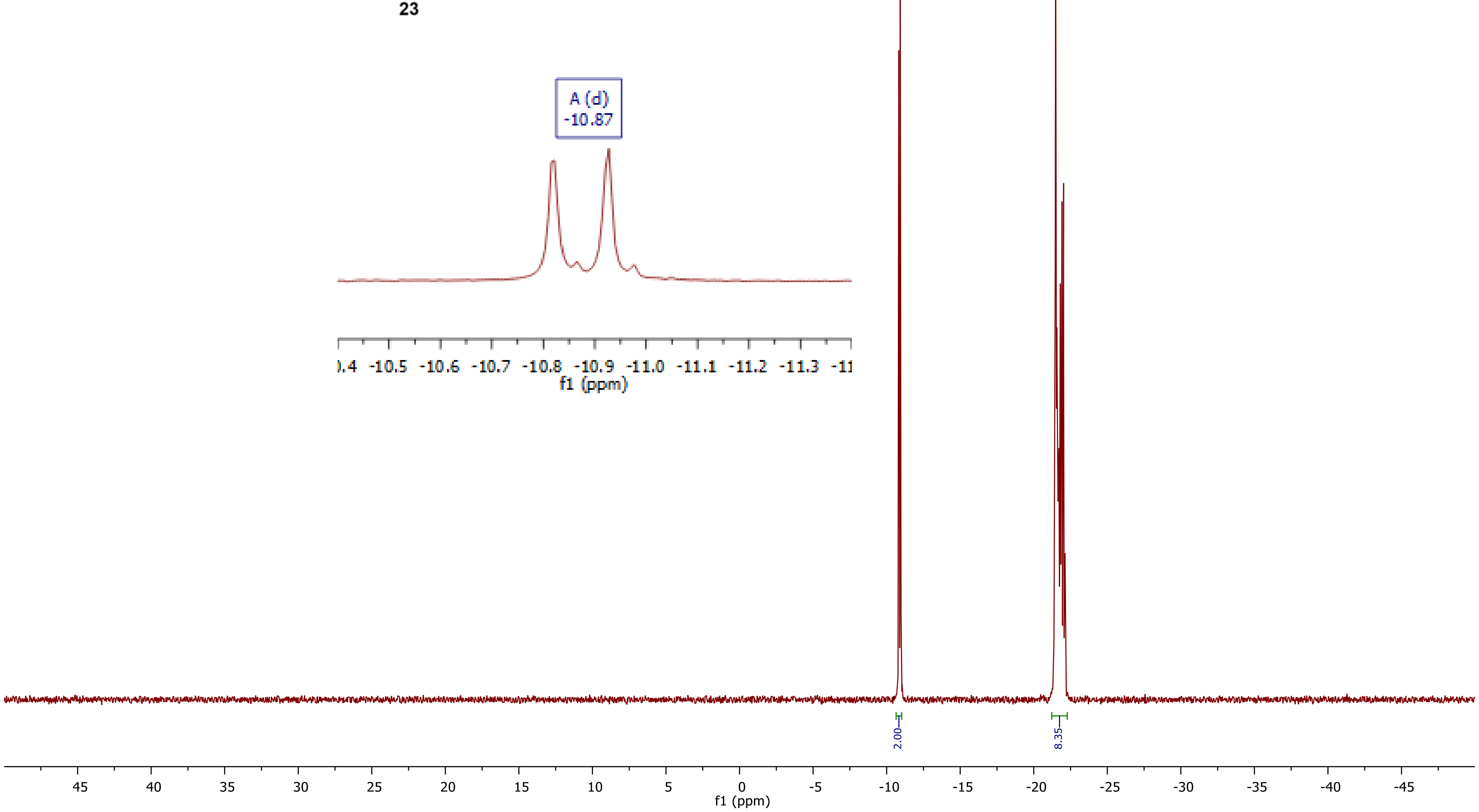

31P-NMR (162 MHz, D2O)

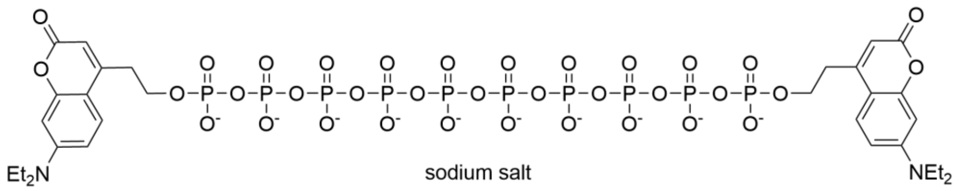

sodium salt

23

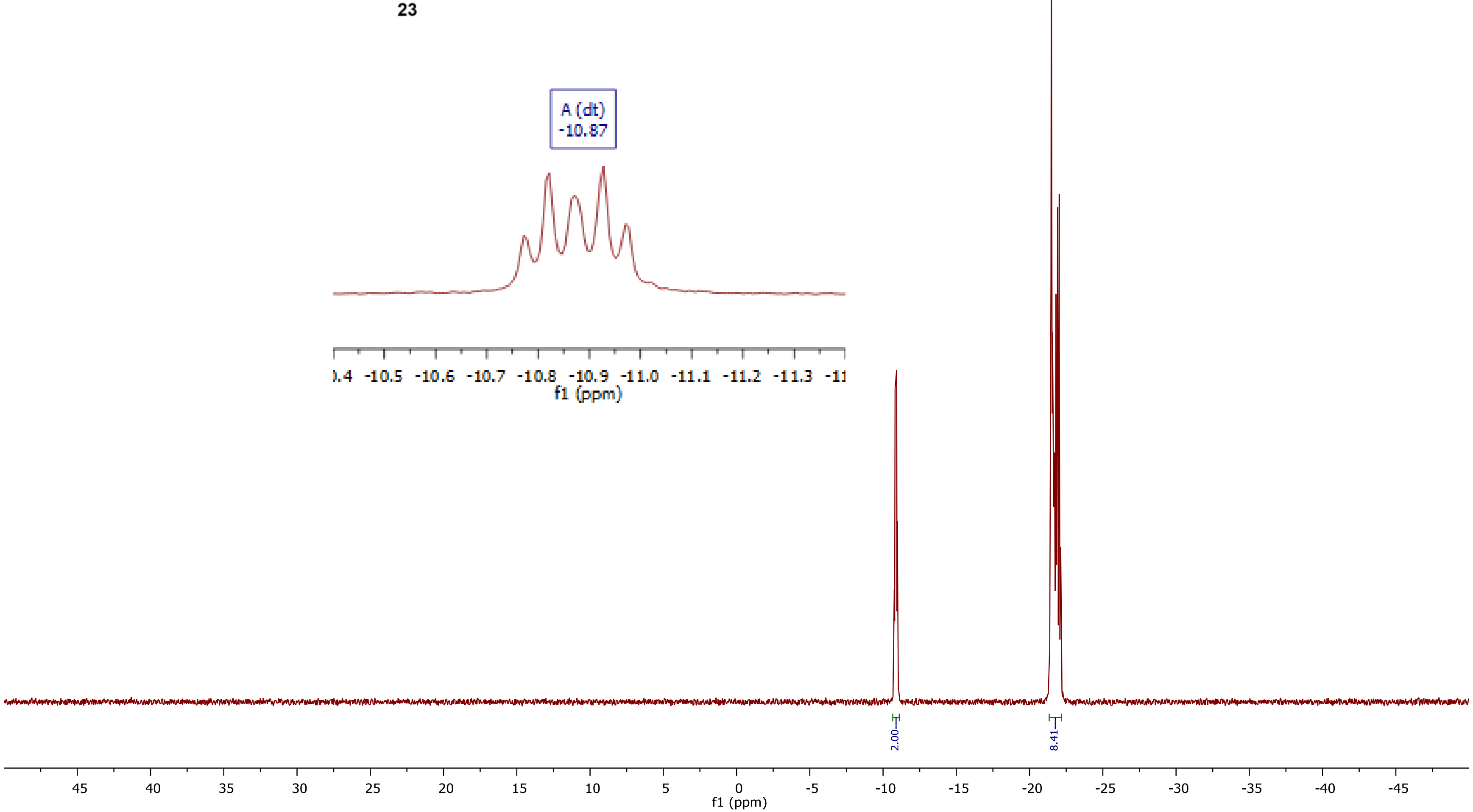

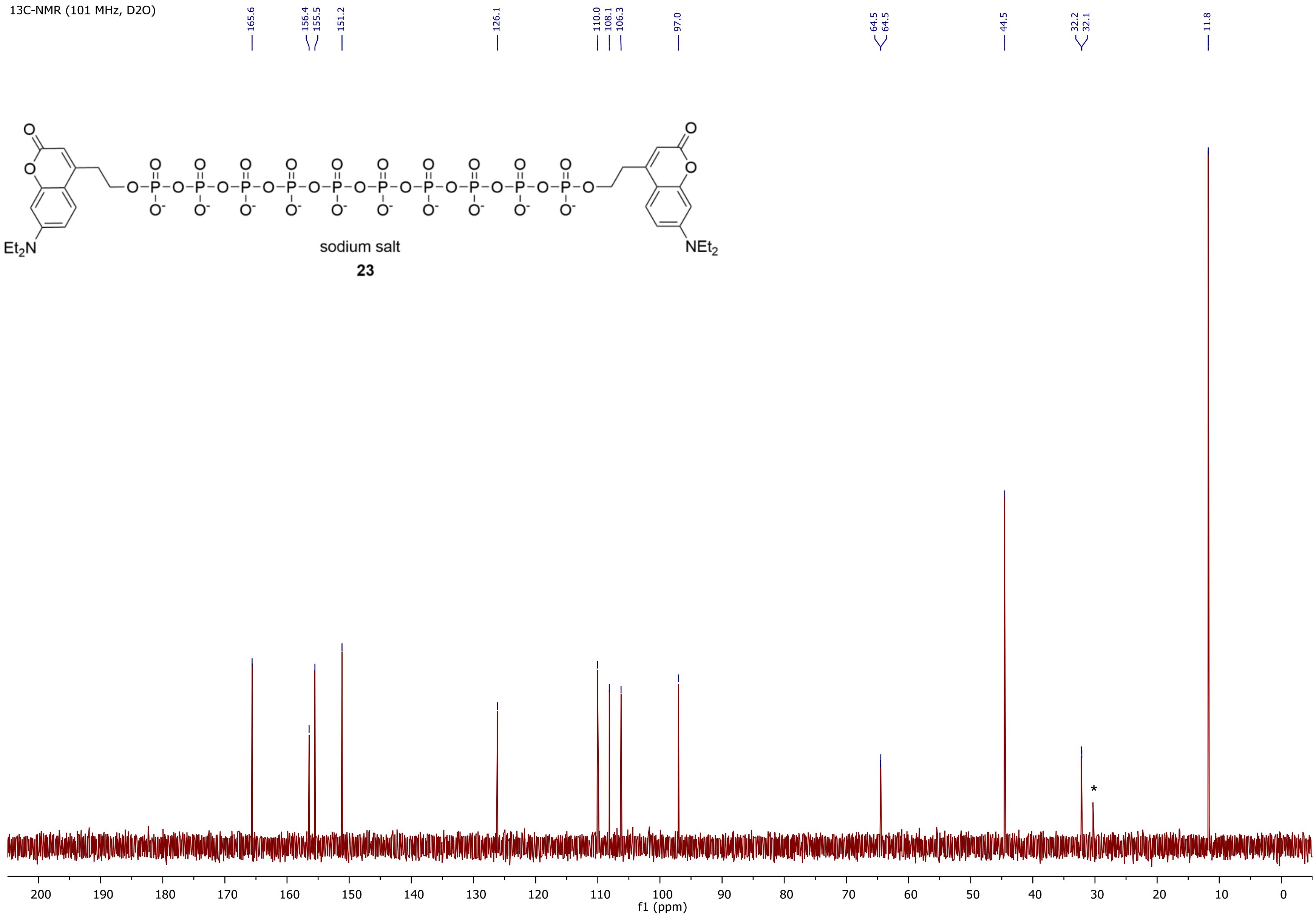

Residual amounts of acetone are marked with asterisks (*).

1H-NMR (400 MHz, D2O)

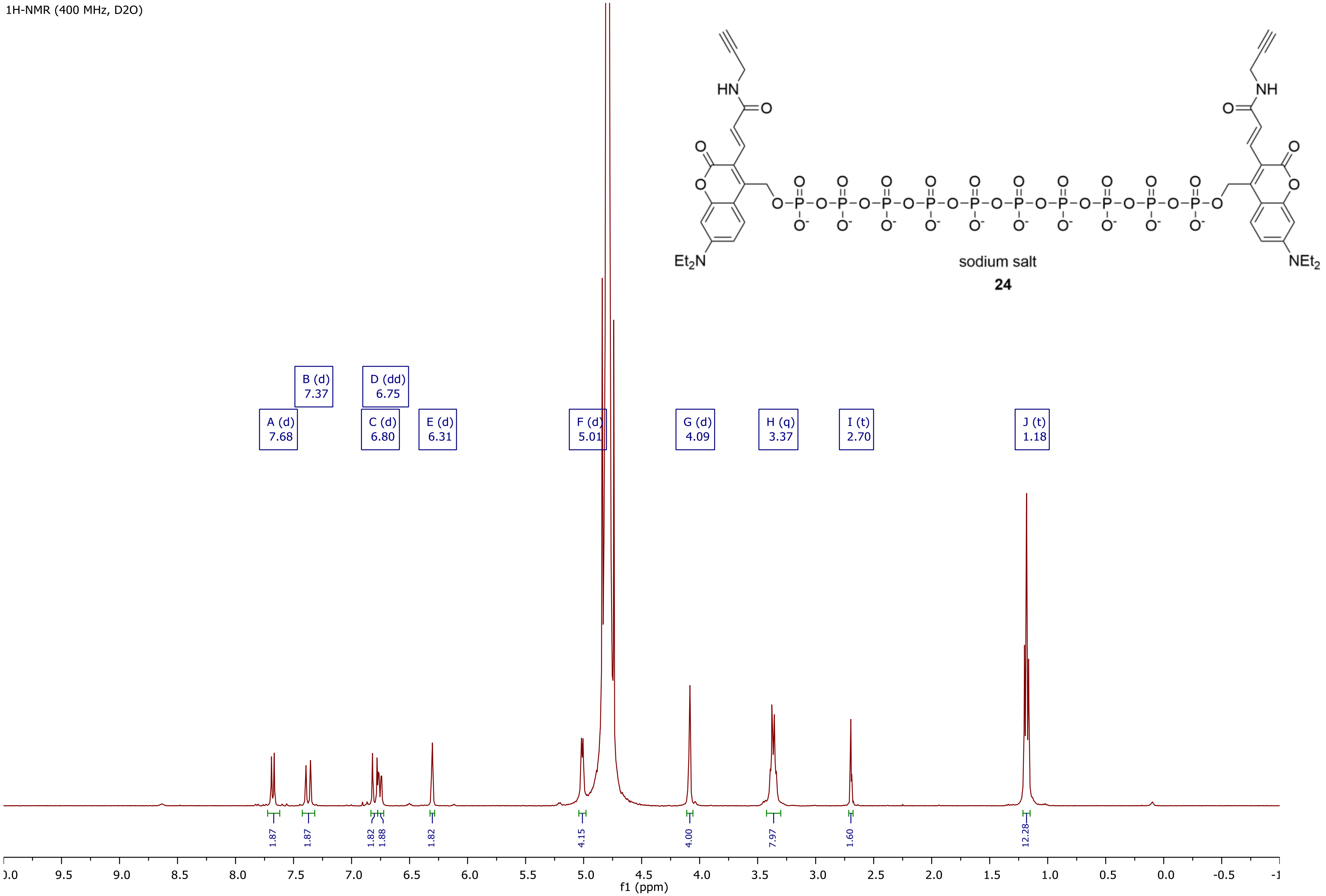

31P{1H}-NMR (162 MHz, D2O)

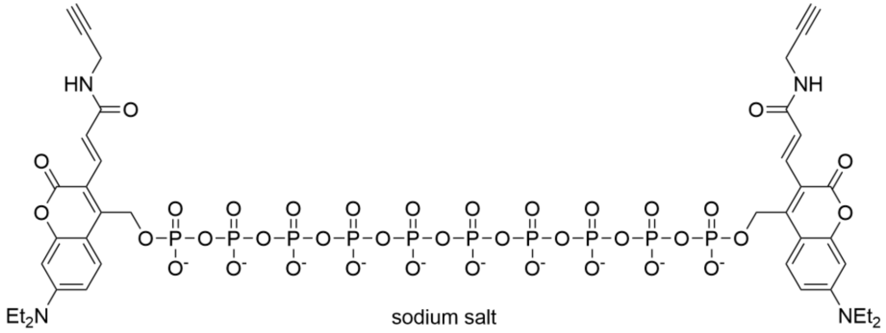

sodium salt

24

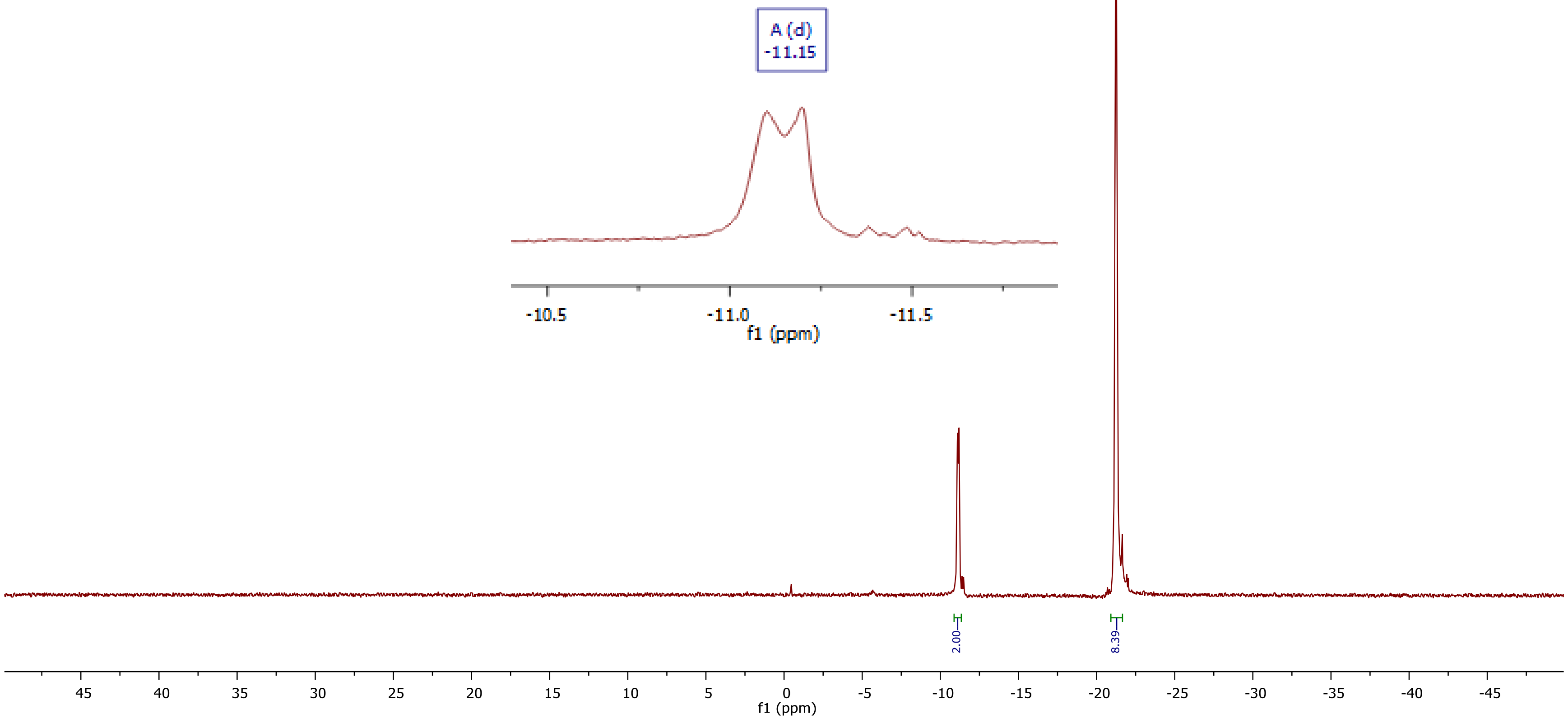

31P-NMR (162 MHz, D2O)

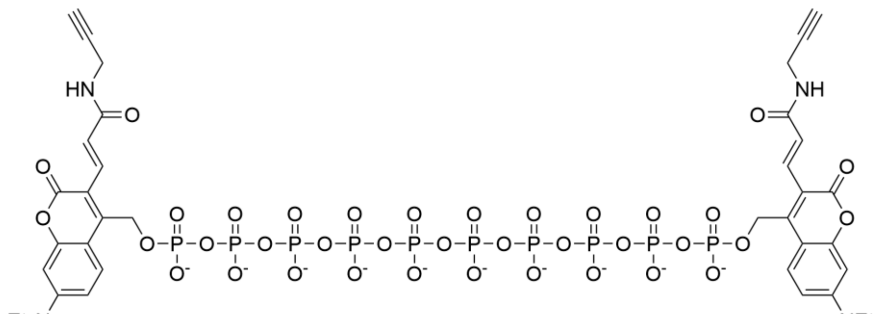

sodium salt

24

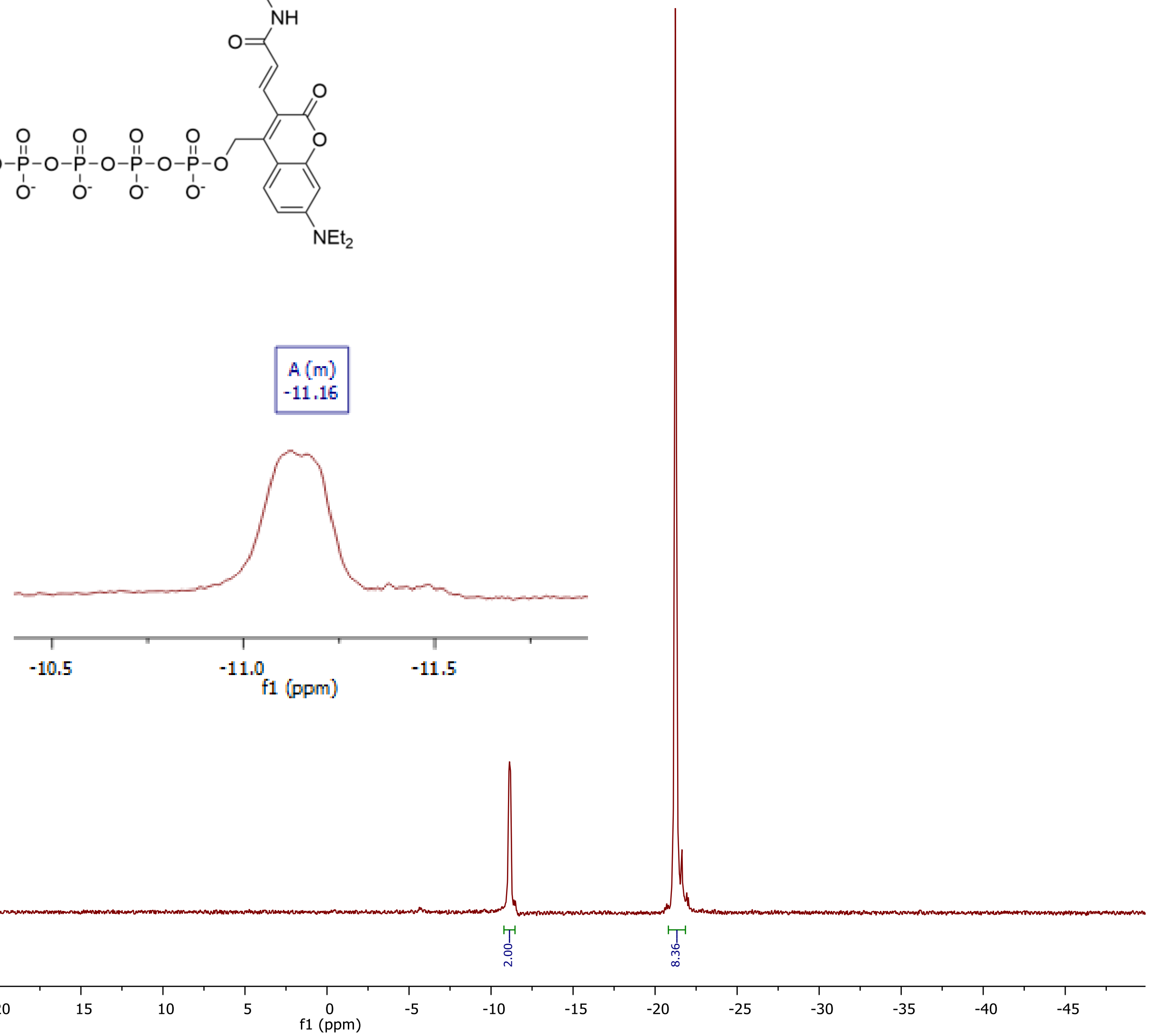

13C-NMR (101 MHz, D2O)

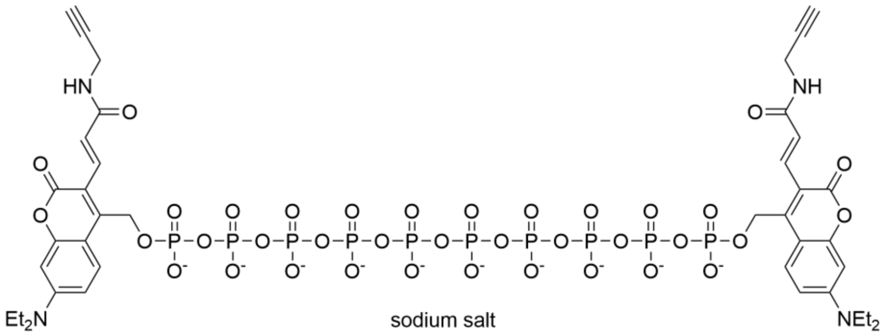

sodium salt

24

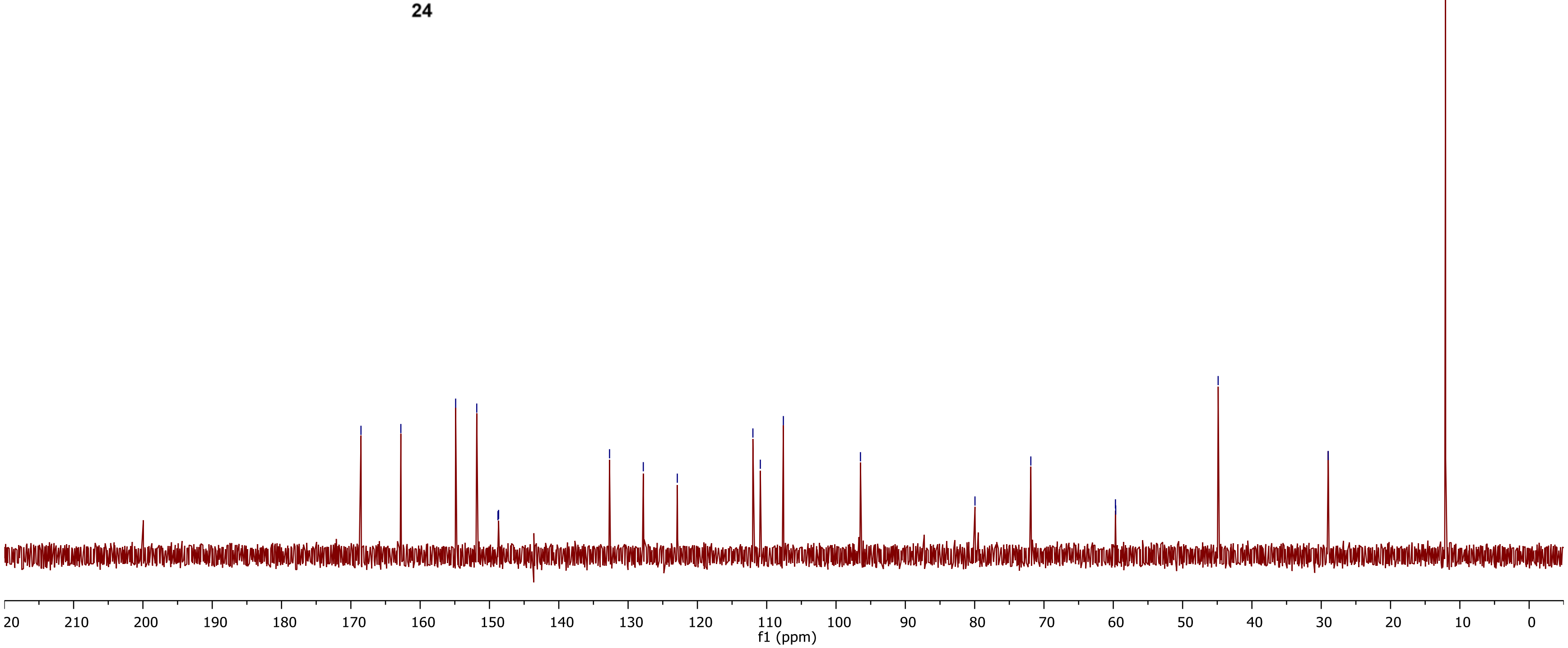

1H-NMR (700 MHz, D2O): before salt removal and shortly in D2O

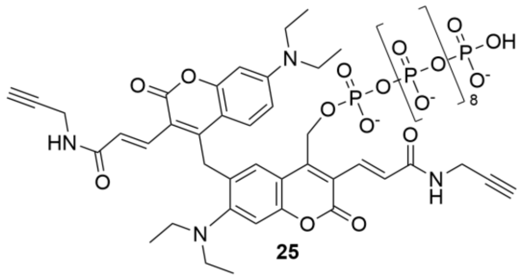

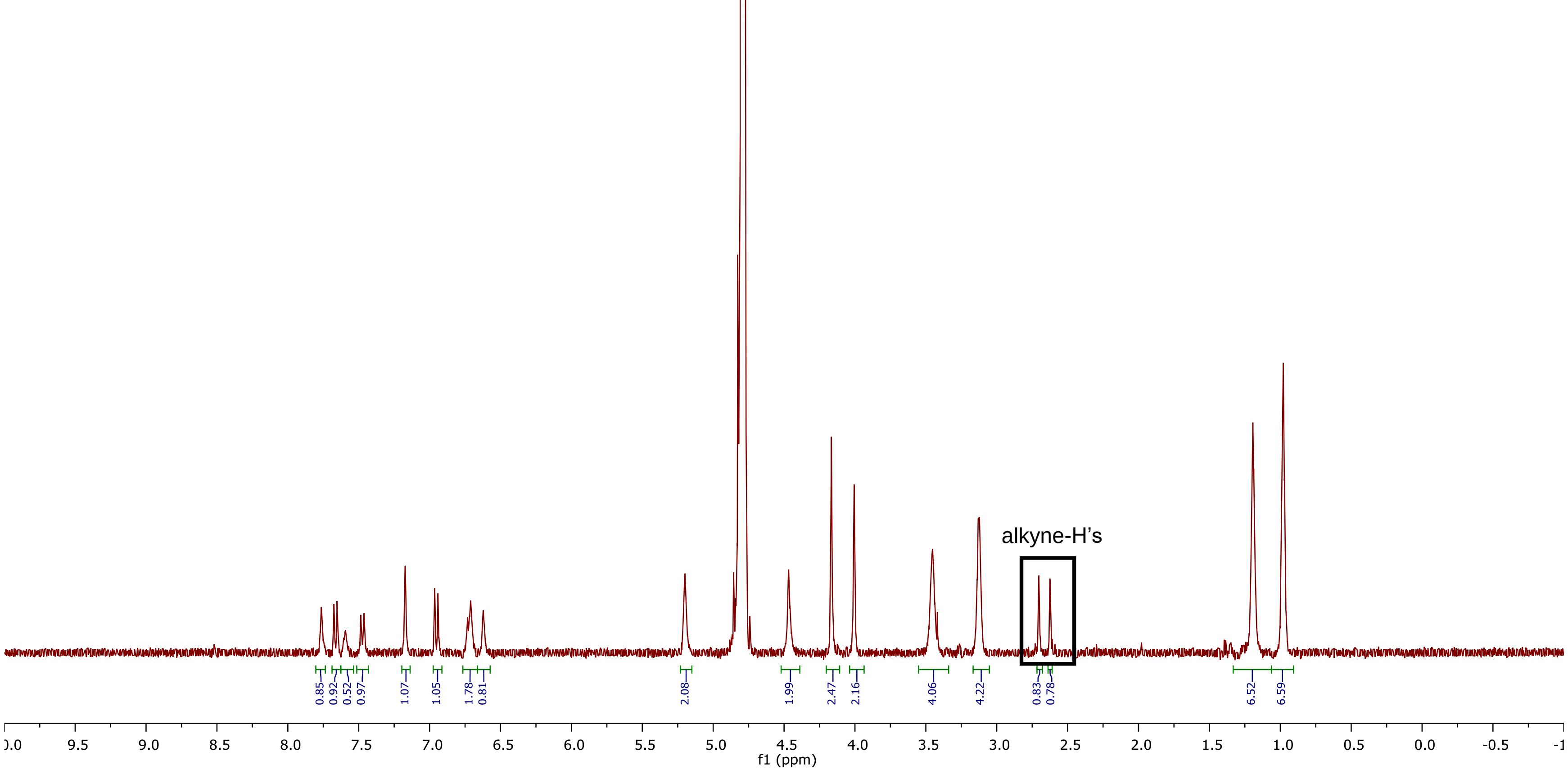

1H-NMR (700 MHz, D2O): after salt removal and long time in D2O

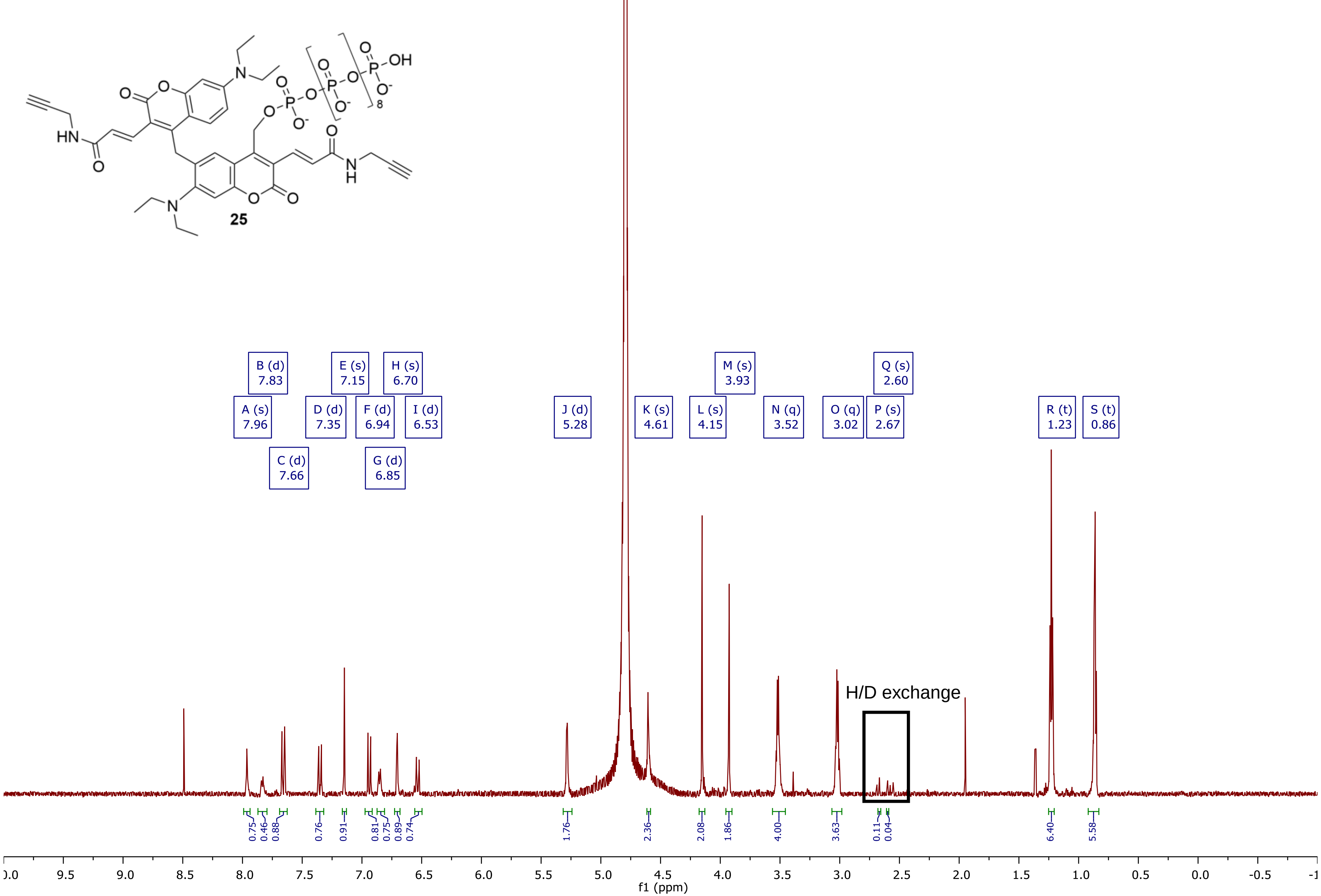

31P{1H}-NMR (283 MHz, D2O)

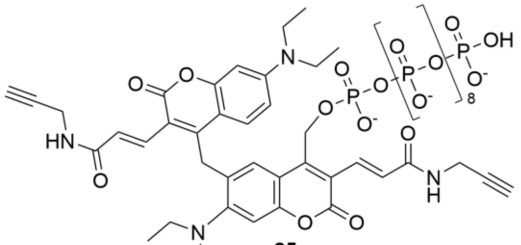

25

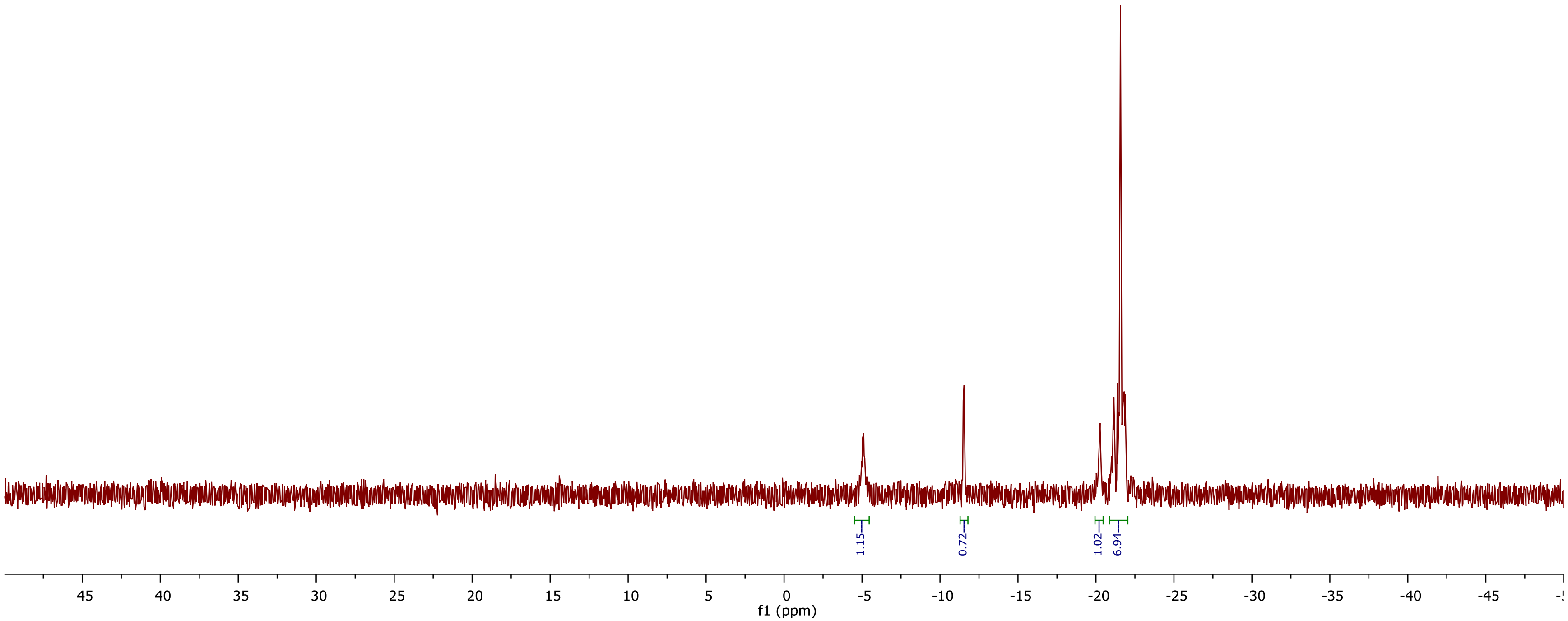

1H-NMR (400 MHz, D2O)

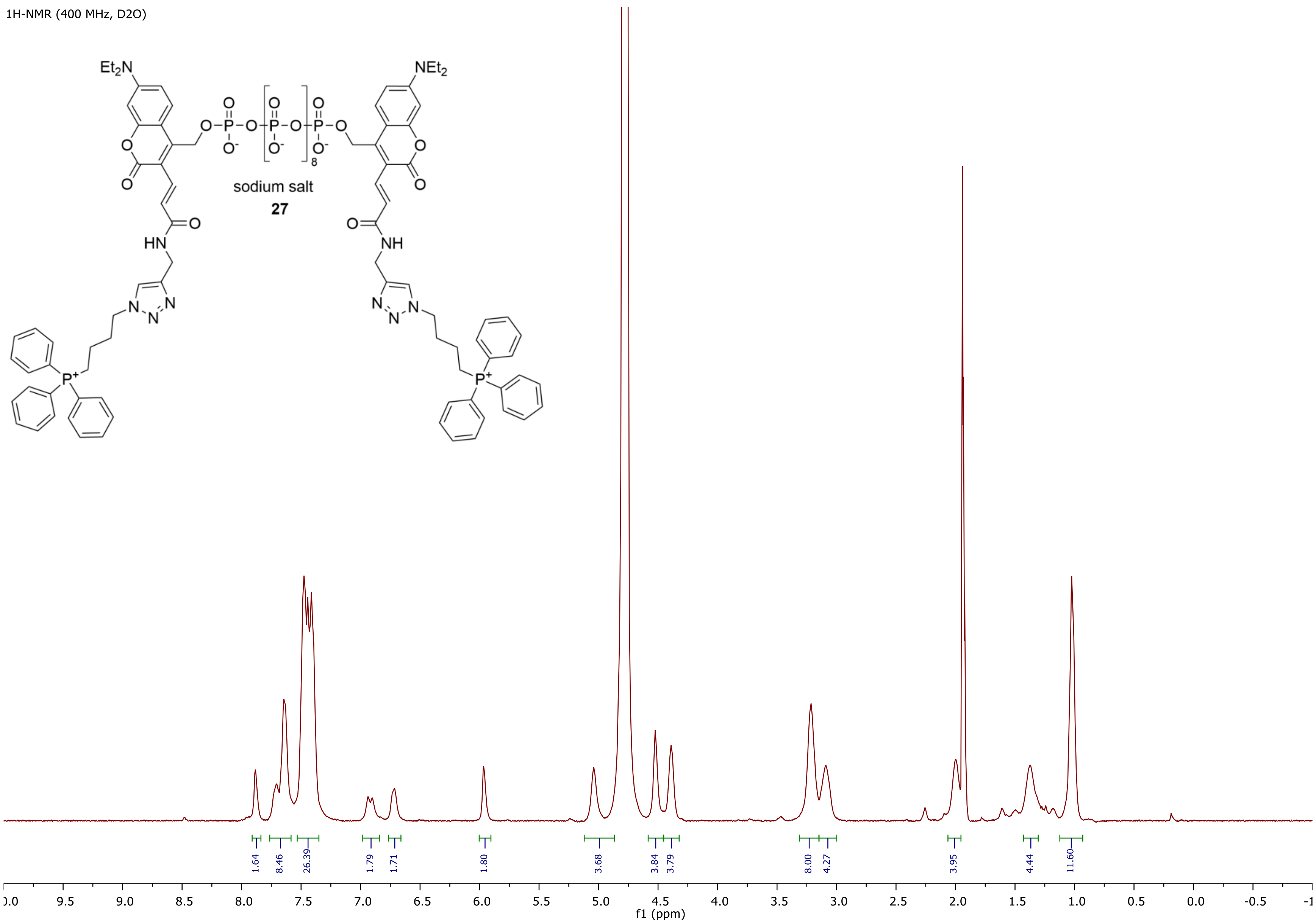

31P{1H}-NMR (162 MHz, D2O)

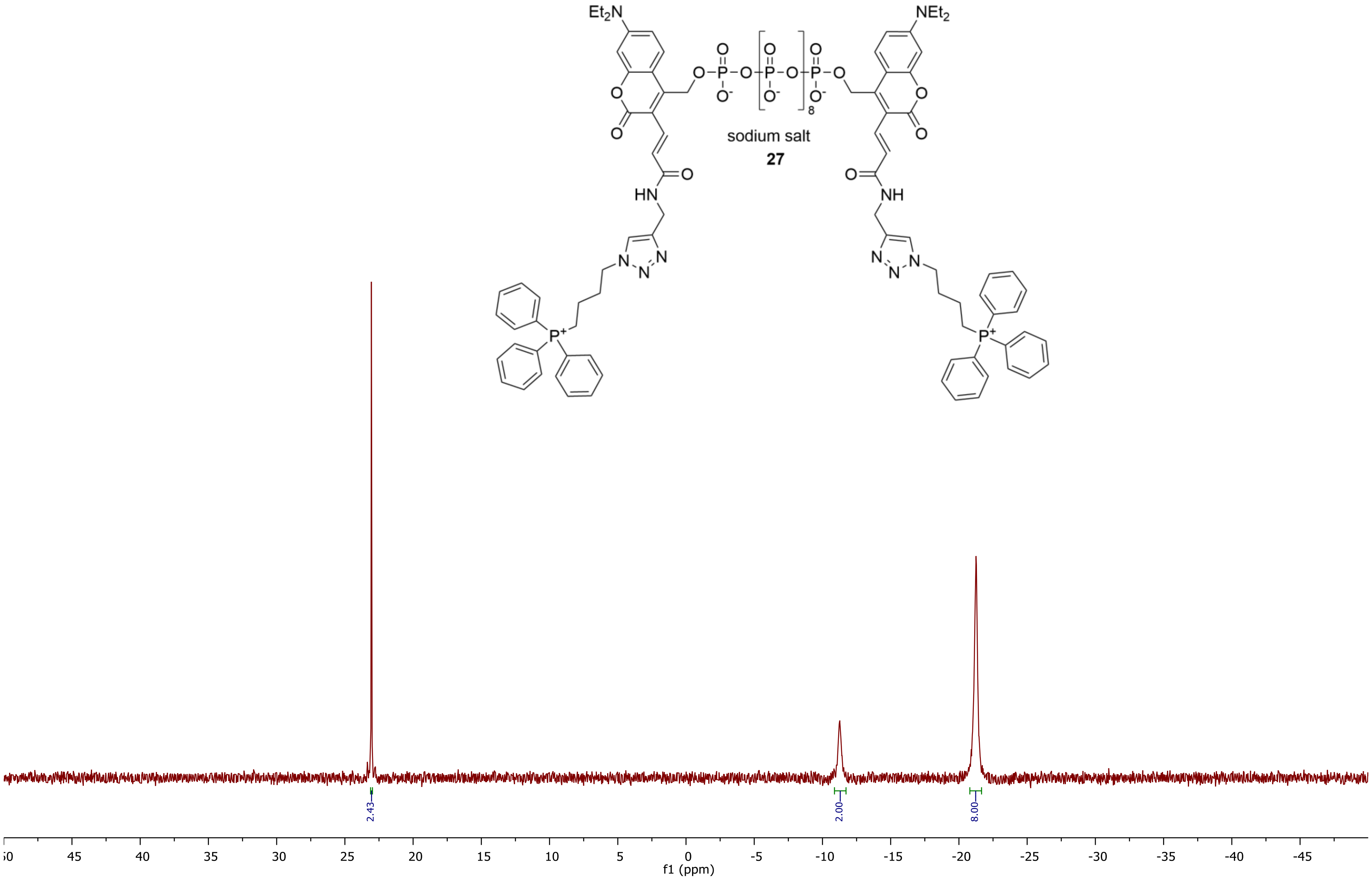

6. HRMS (ESI) Data

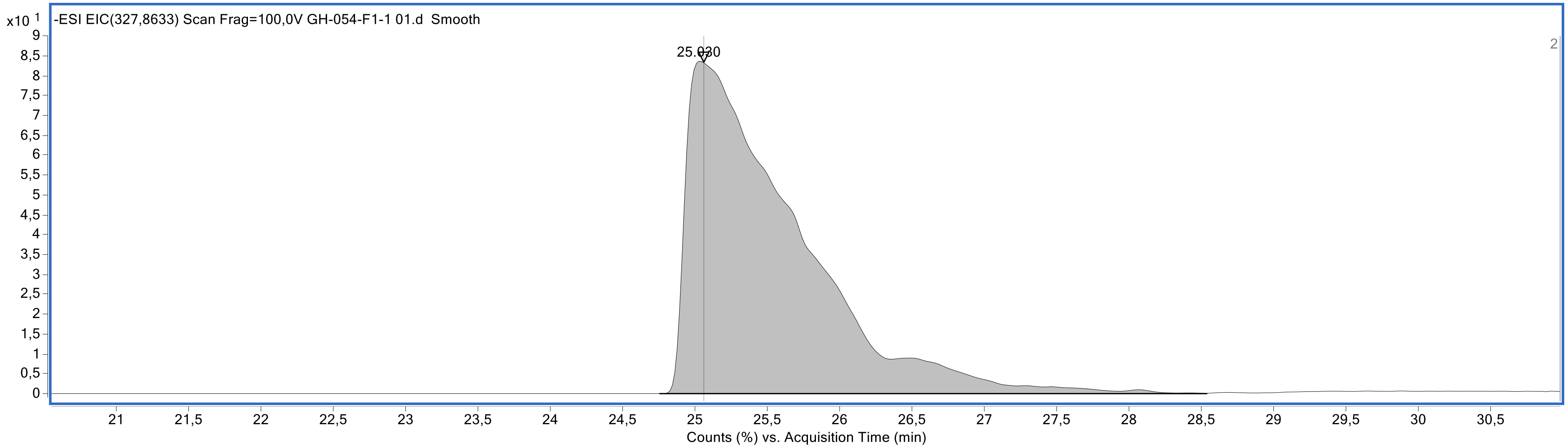

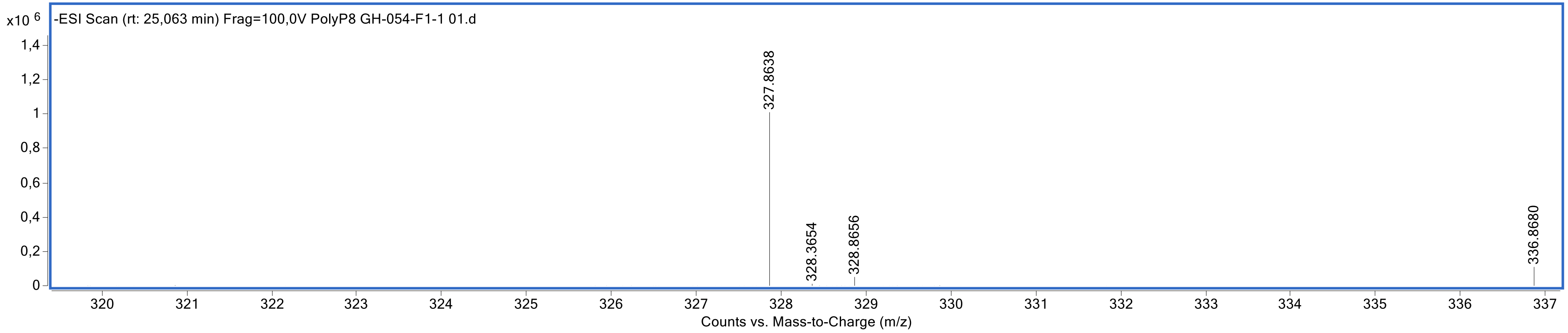

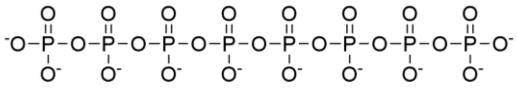

6

sodium salt

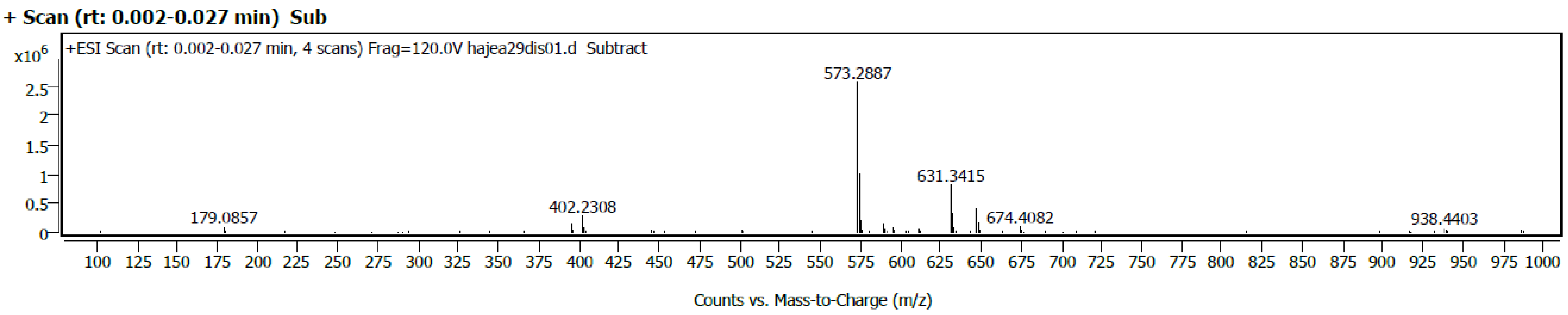
+ Scan (rt: 0.002-0.027 min) Sub
+ESI Scan (rt: 0.002-0.027 min, 4 scans) Frag=120.0V hajea29dis01.d Subtract
x10^6
573.2887
631.3415
402.2308
179.0857
674.4082
938.4403
Counts vs. Mass-to-Charge (m/z)

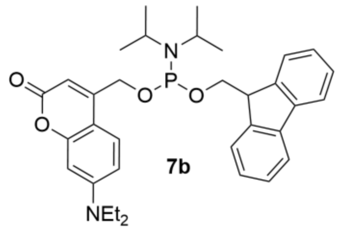
NEt2
O
N
P

7b

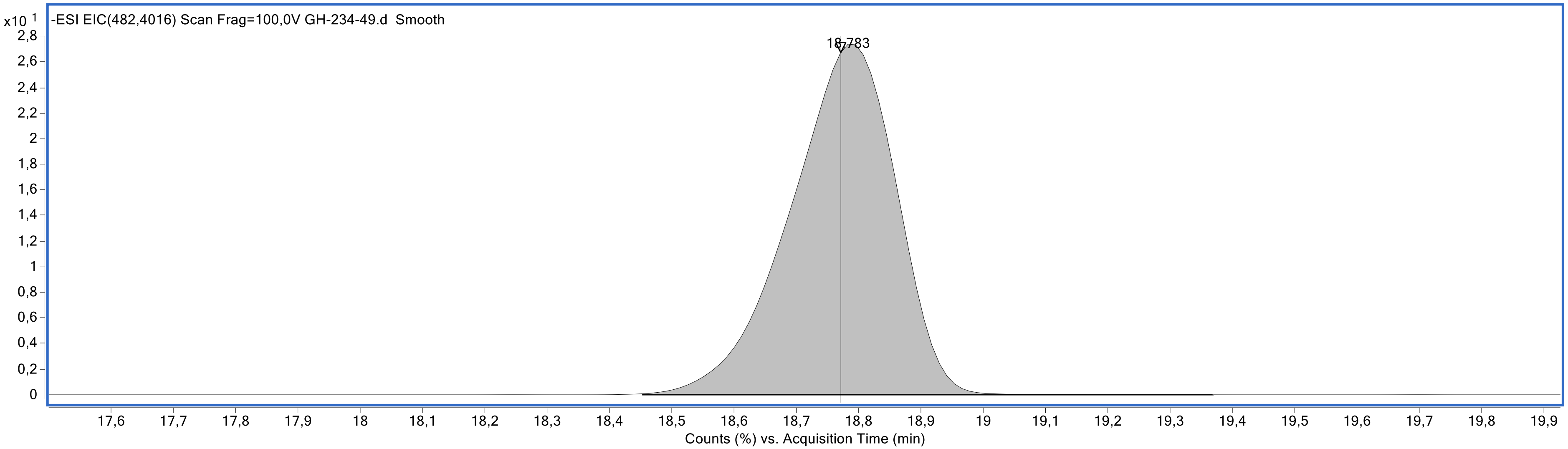

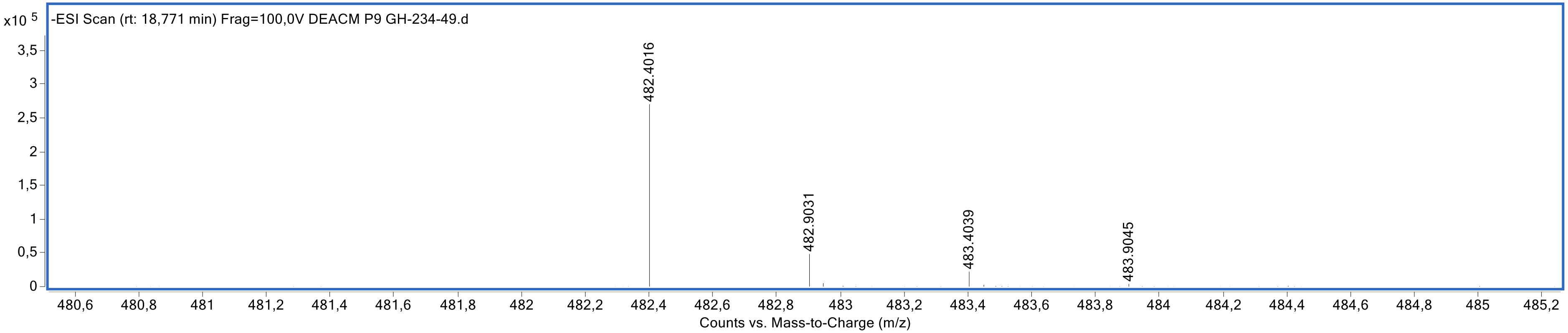

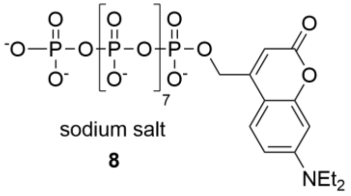

sodium salt

8

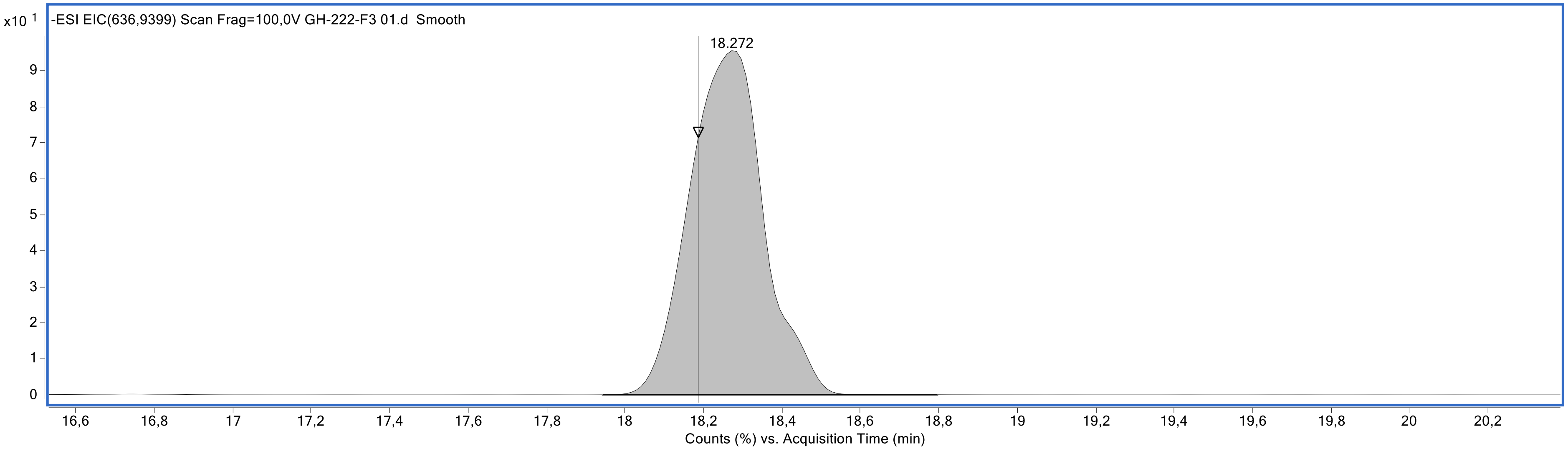

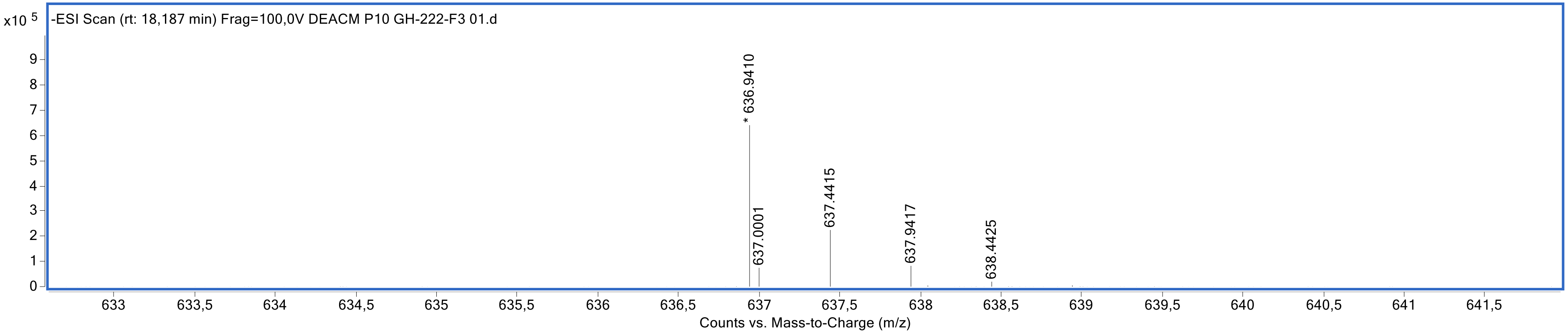

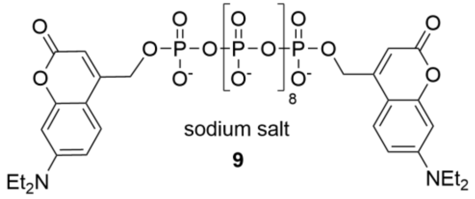

sodium salt

9

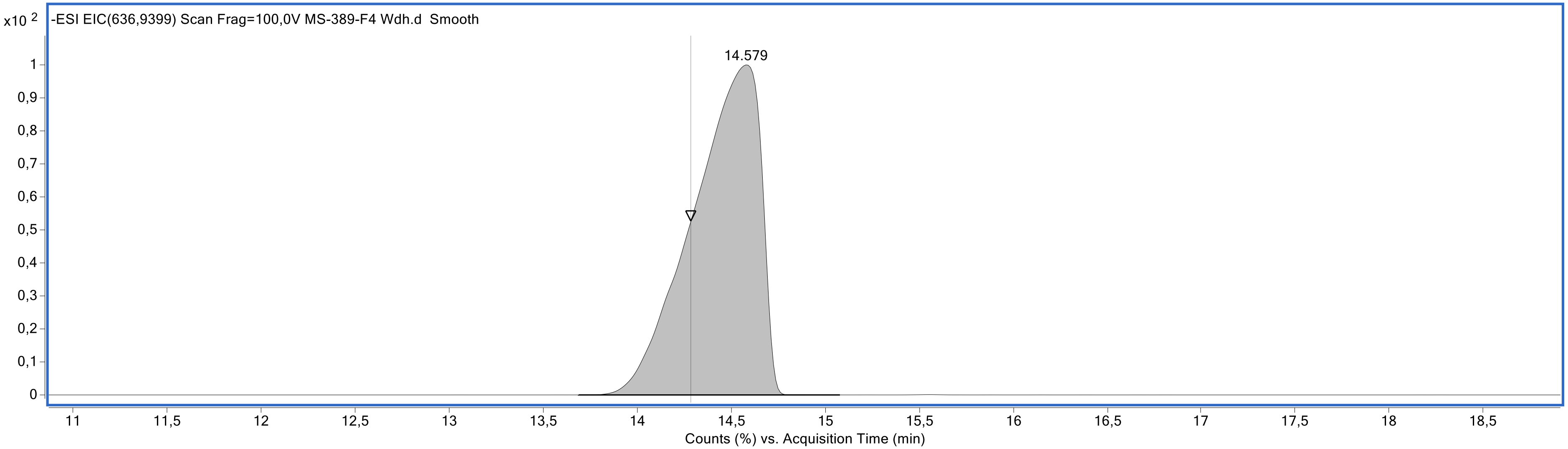
x10 2
-ESI EIC(636,9399) Scan Frag=100,0V MS-389-F4 Wdh.d Smooth
14.579
Counts (%) vs. Acquisition Time (min)

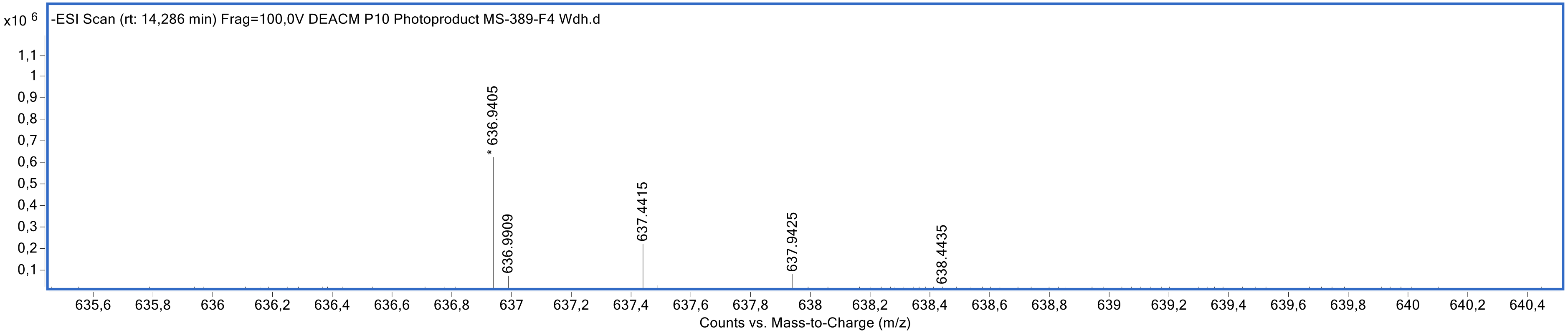
x10 6
-ESI Scan (rt: 14,286 min) Frag=100,0V DEACM P10 Photoproduct MS-389-F4 Wdh.d
* 636.9405
636.9909
637.4415
637.9425
638.4435
Counts vs. Mass-to-Charge (m/z)

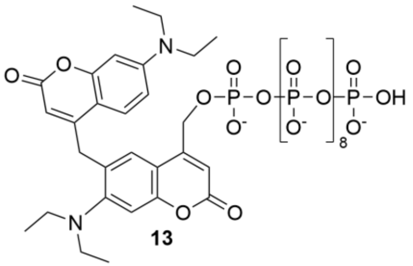

13

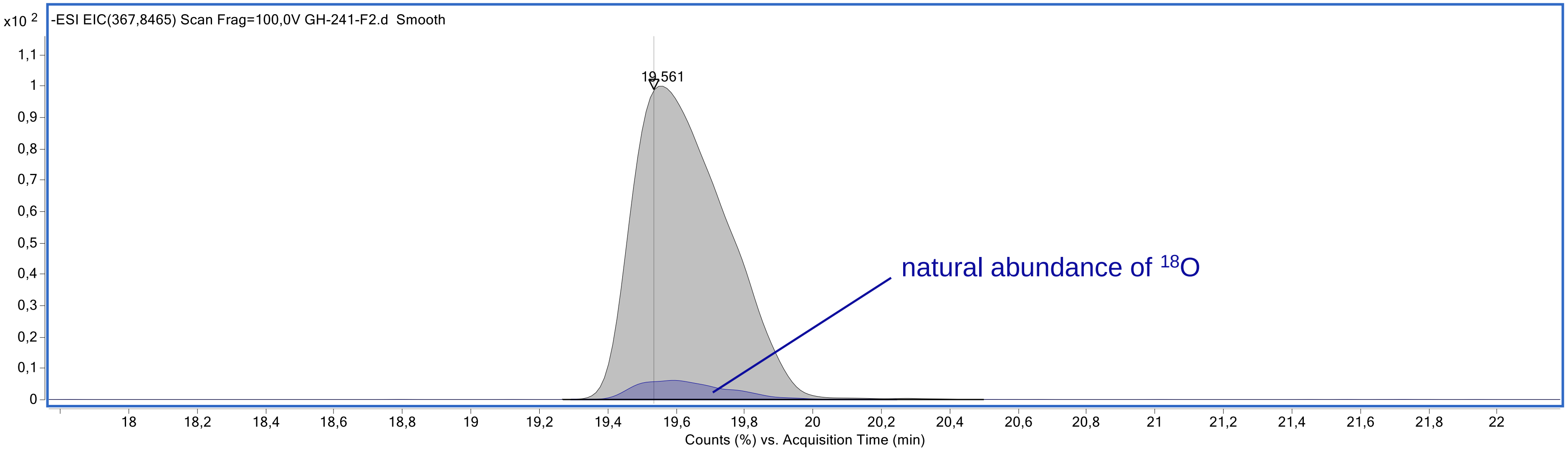

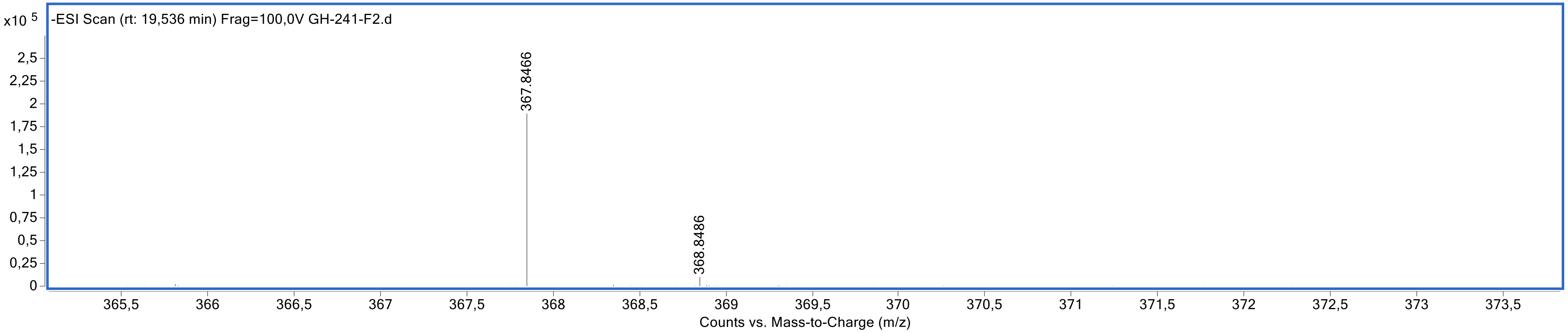

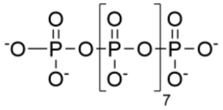

sodium salt

10

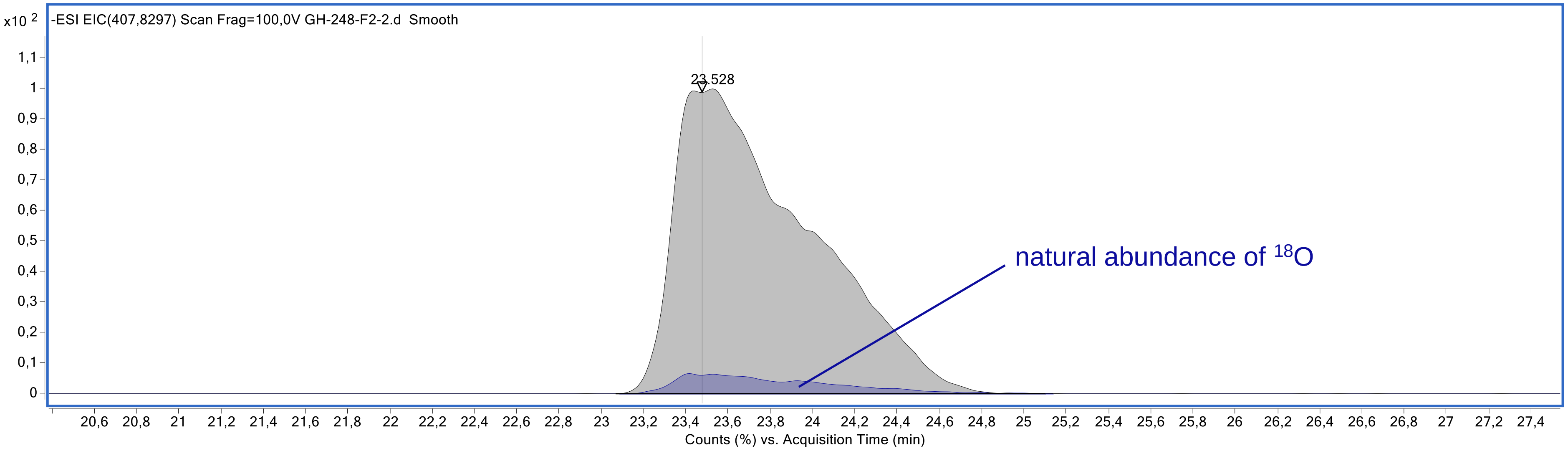

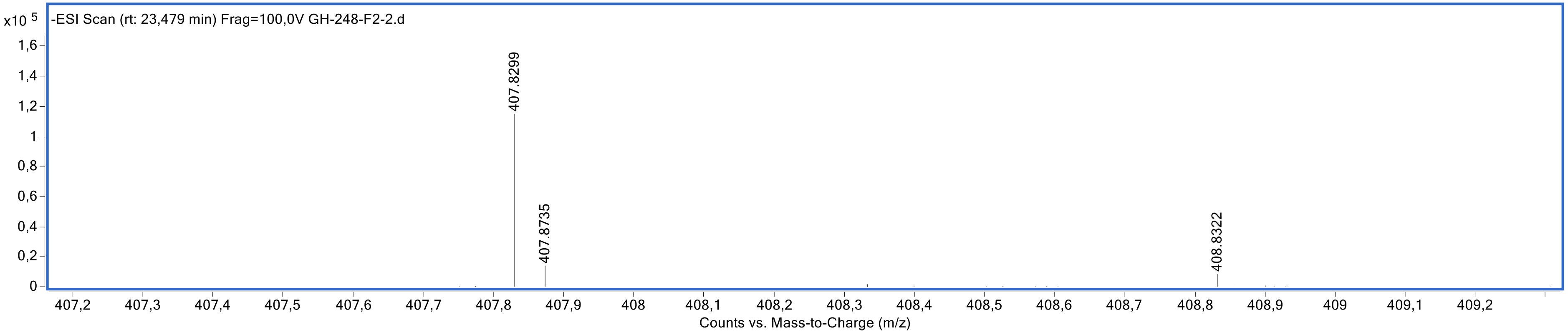

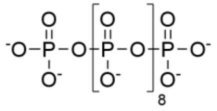

sodium salt

11

D:\data_2022\mujec10shr2 1/12/2022 2:57:03 PM sm.iv.73

mujec10shr2 #1 RT: 0.02 AV: 1 NL: 2.81E6
T: FTMS + p ESI Full lock ms [50.00-900.00]

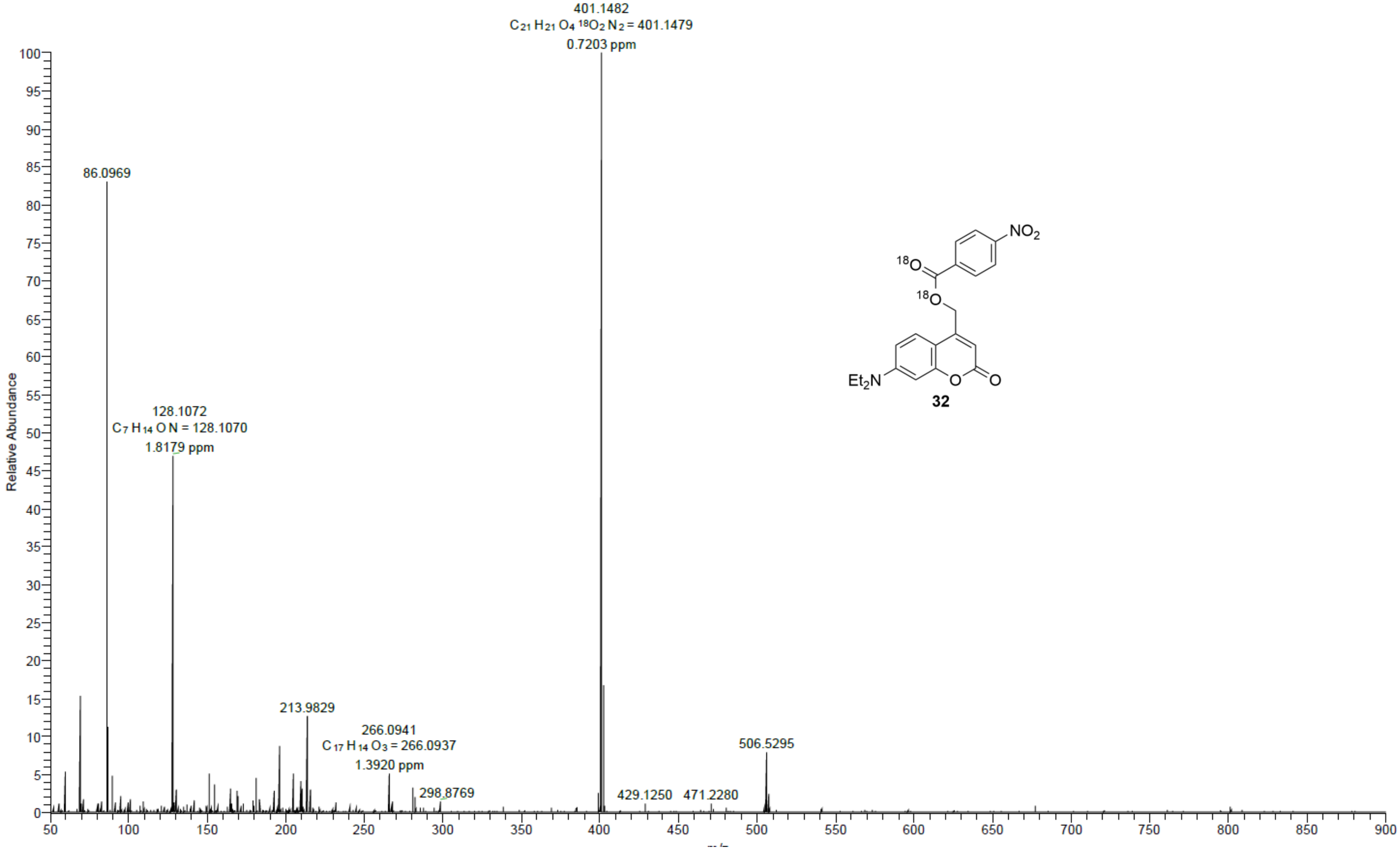

D:\data_2022\mujec11shr1 1/12/2022 3:01:16 PM sm.iv.76

mujec11shr1 #1 RT: 0.02 AV: 1 NL: 1.39E7
T: FTMS + p ESI Full lock ms [50.00-700.00]

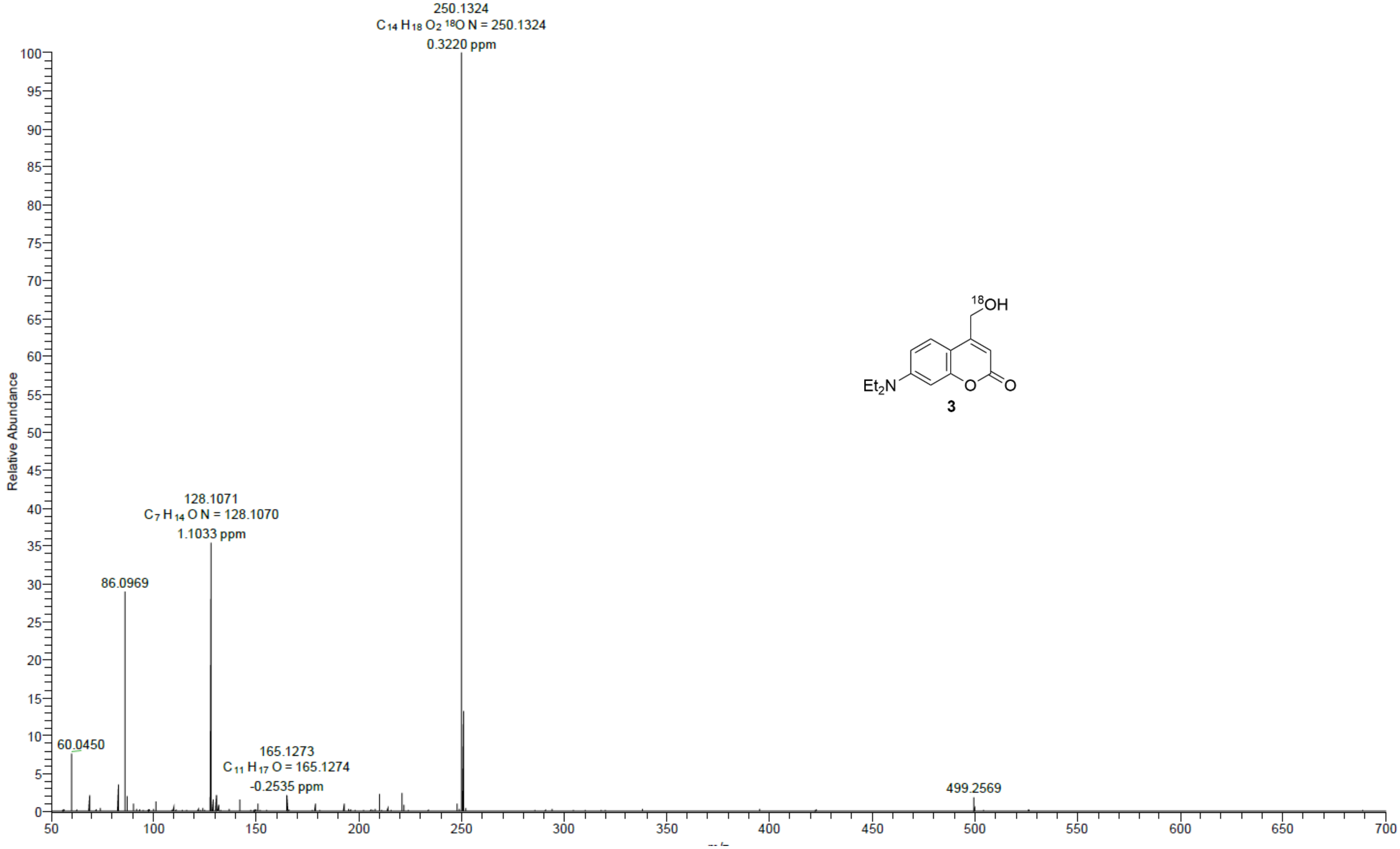

3

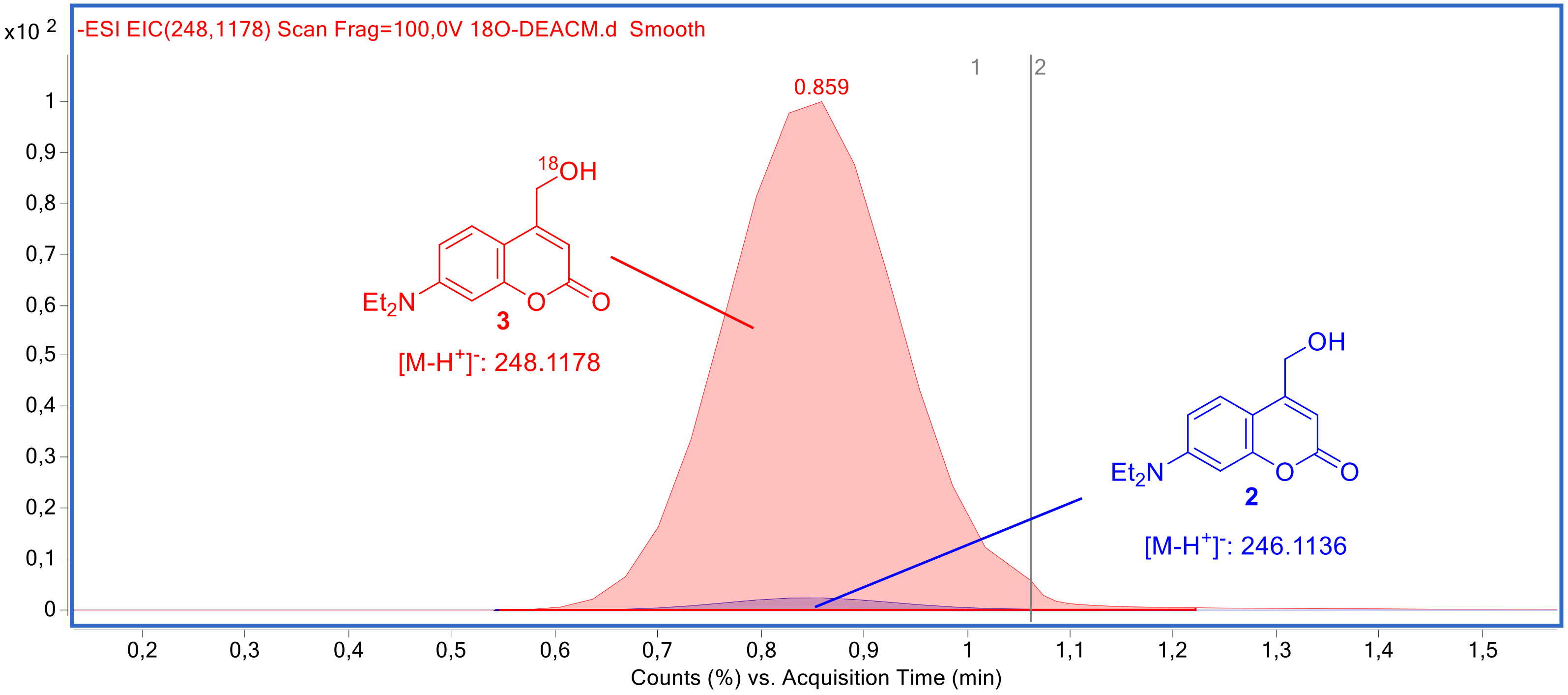

Isotope enrichment (CE-ESI-QTOF, $[M-H]^-$, 248.1178 ($^{18}O_1$) / 246.1136 ($^{18}O_0$)): 98:2.

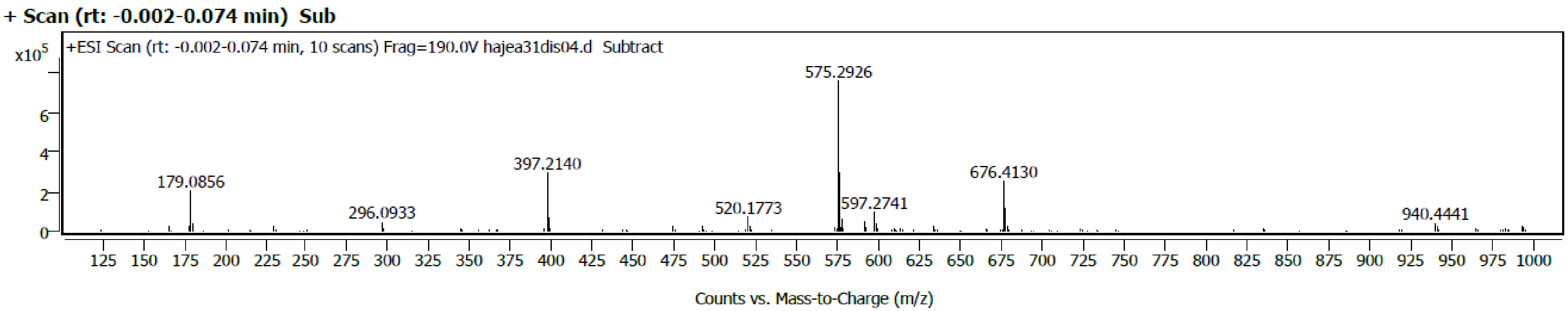
+ Scan (rt: -0.002-0.074 min) Sub
x10^5
+ESI Scan (rt: -0.002-0.074 min, 10 scans) Frag=190.0V hajea31dis04.d Subtract
575.2926
397.2140
179.0856
296.0933
520.1773
597.2741
676.4130
940.4441
0
2
4
6
125 150 175 200 225 250 275 300 325 350 375 400 425 450 475 500 525 550 575 600 625 650 675 700 725 750 775 800 825 850 875 900 925 950 975 1000
Counts vs. Mass-to-Charge (m/z)

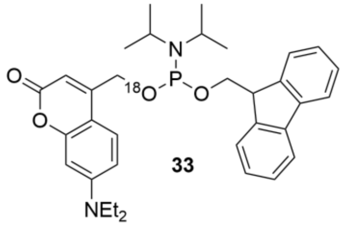
N
P
O
O
O
O
18O
NEt2

33

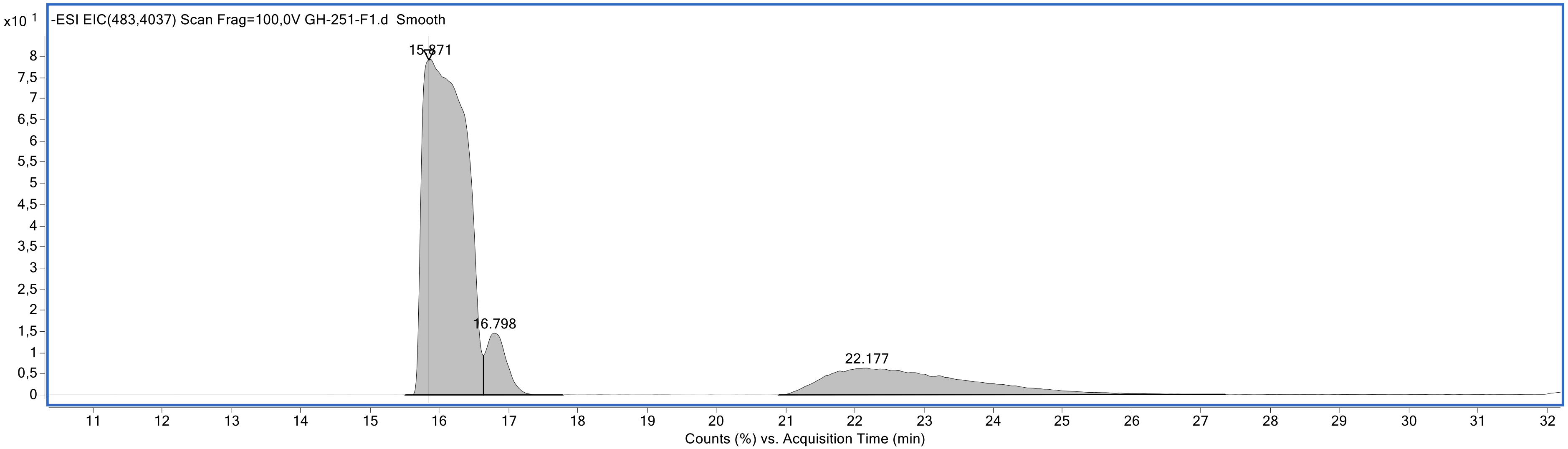

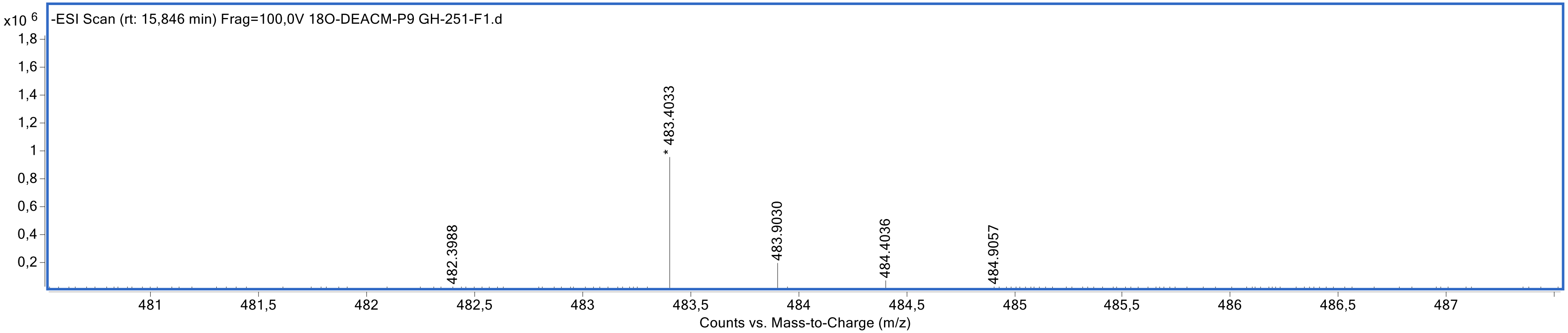

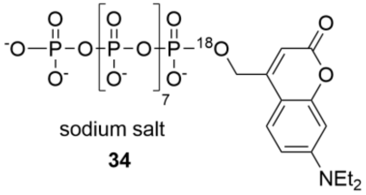

sodium salt

34

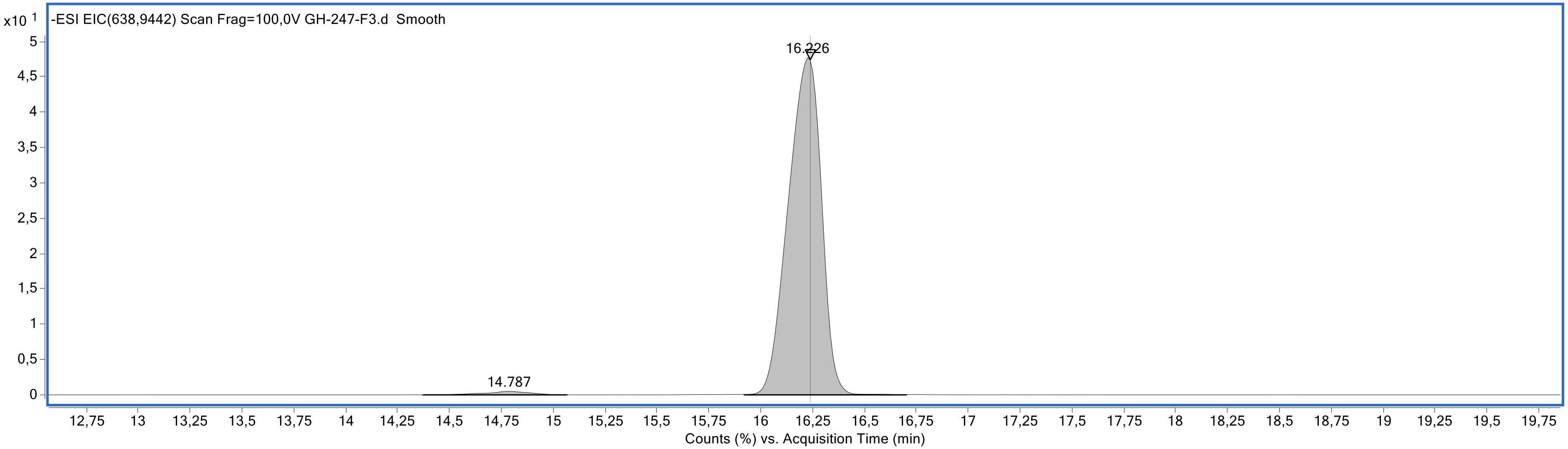

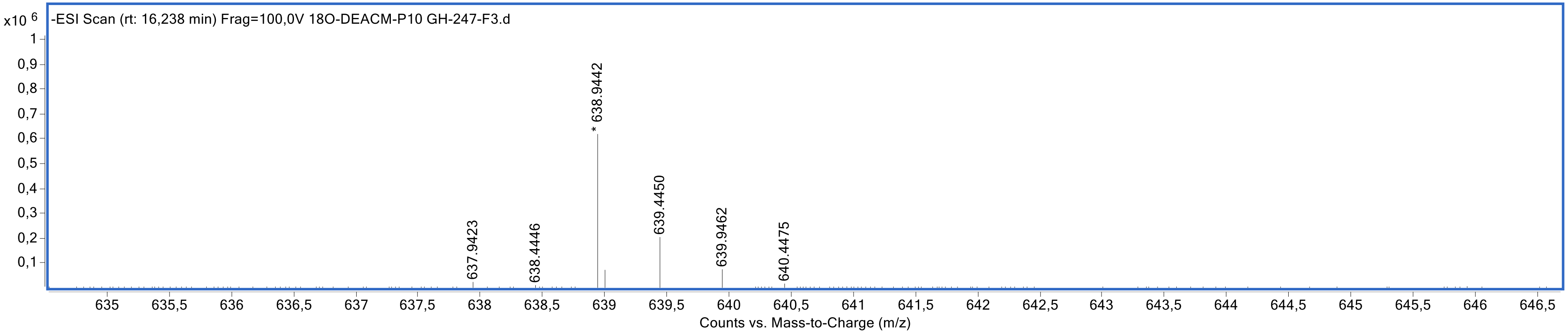

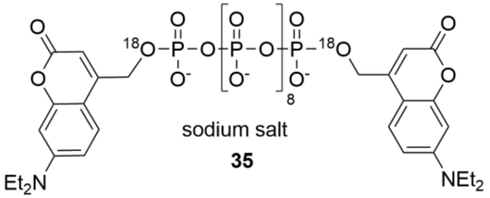

sodium salt

35

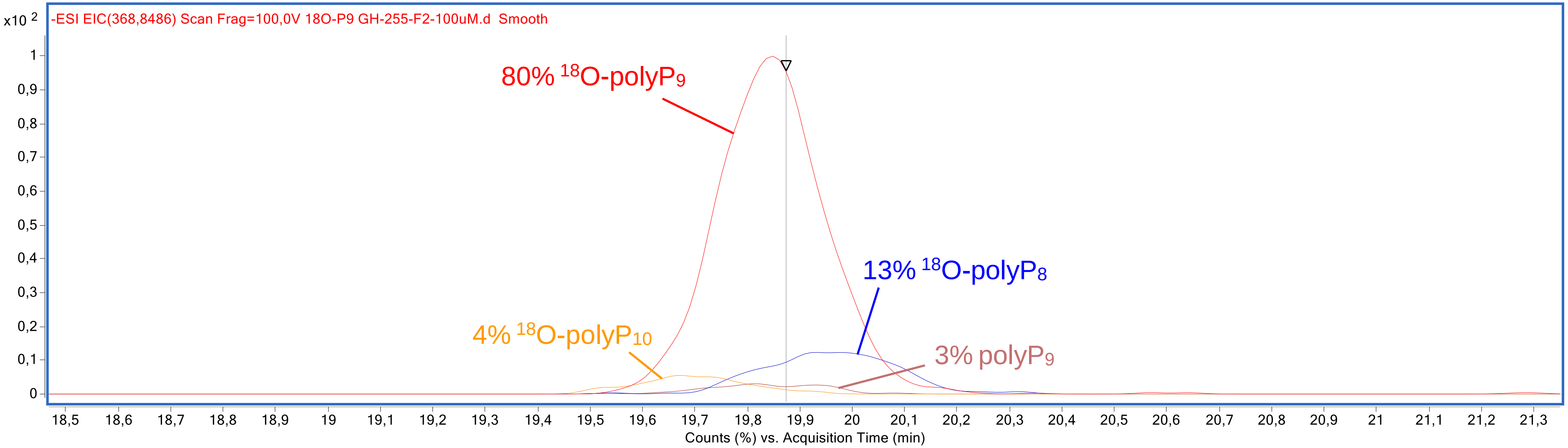

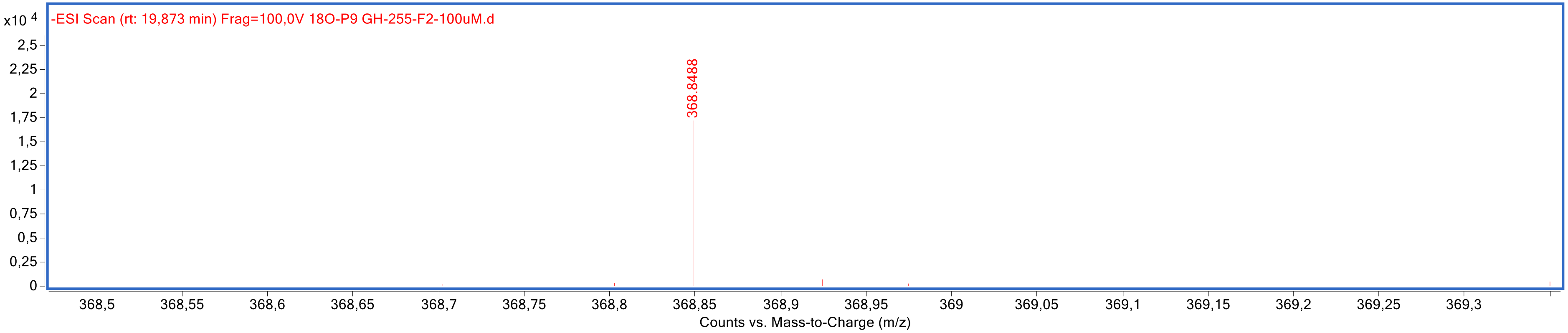

Isotope enrichment of ^{18}O-labeled polyP$_9$ (CE-ESI-QTOF, $[M-2H]^{2-}$, 368.8486 ($^{18}O_1$) / 367.8465 ($^{18}O_0$)): 97:3.

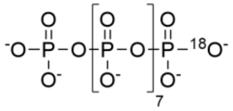

sodium salt

36

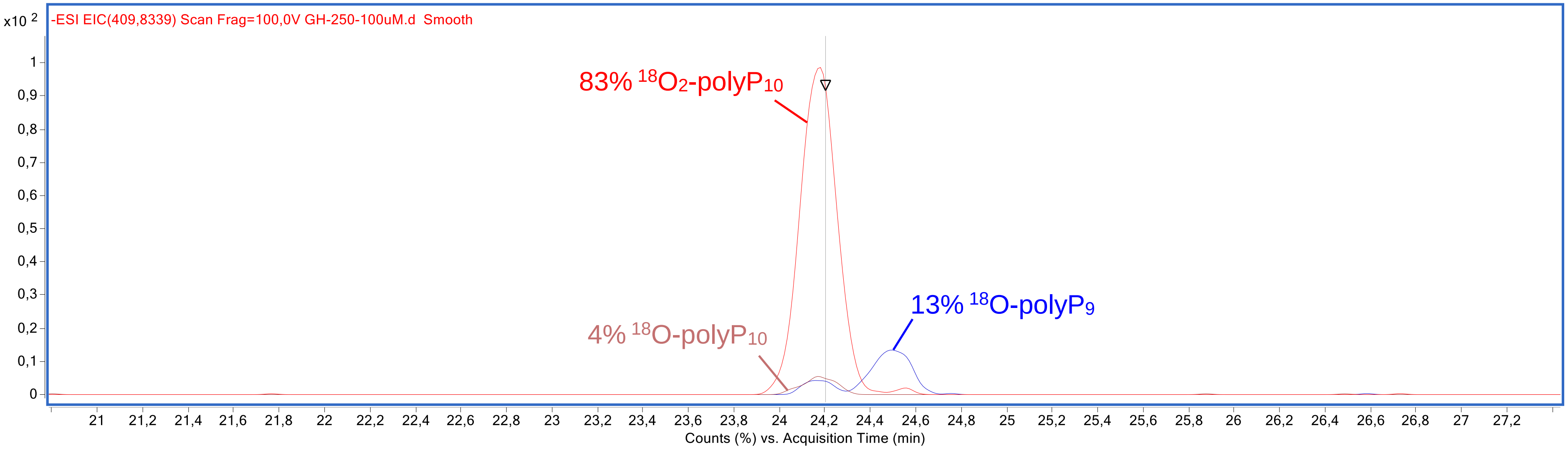

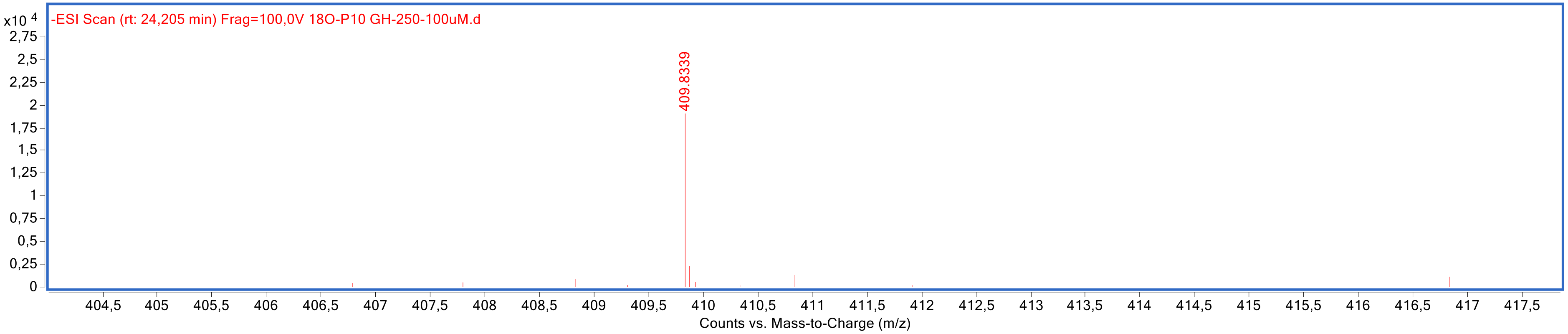

Isotope enrichment of ^{18}O-labeled polyP$_{10}$ (CE-ESI-QTOF, [M-2H]$^{2-}$, 409.8339 ($^{18}O_2$) / 408.8318 ($^{18}O_1$) / 407.8297 ($^{18}O_0$)): 95:5:0

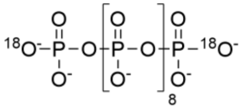

sodium salt

37

D:\data_2022\msjea71thr3 2/2/2022 8:23:08 AM ms101f1
vap350

msjea71thr3 #1 RT: 0.02 AV: 1 NL: 8.12E6
T: FTMS + p APCI corona Full lock ms [100.00-700.00]

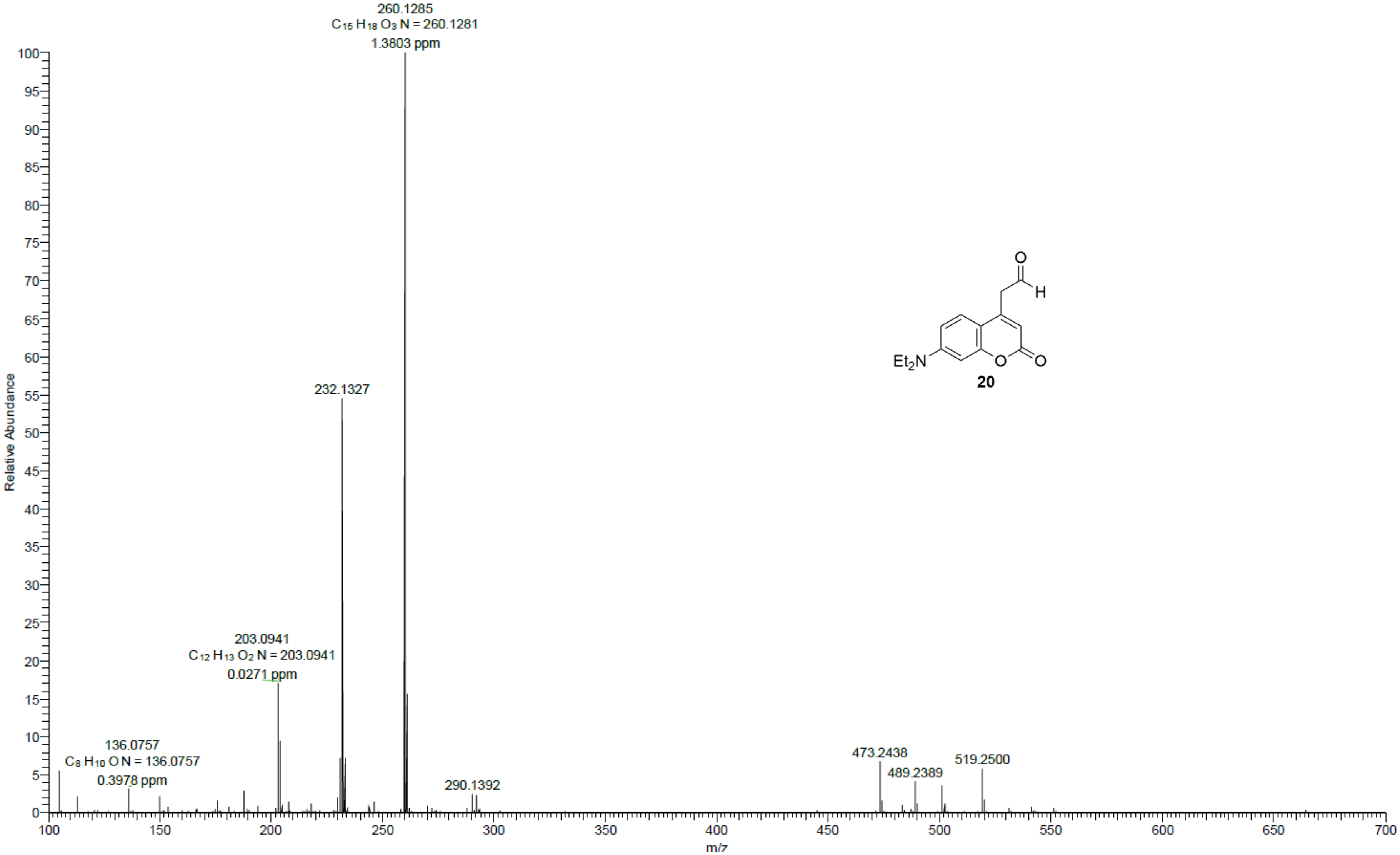

20

D:\data_2022\msjea72shr4 2/11/2022 10:53:34 AM ms111f1

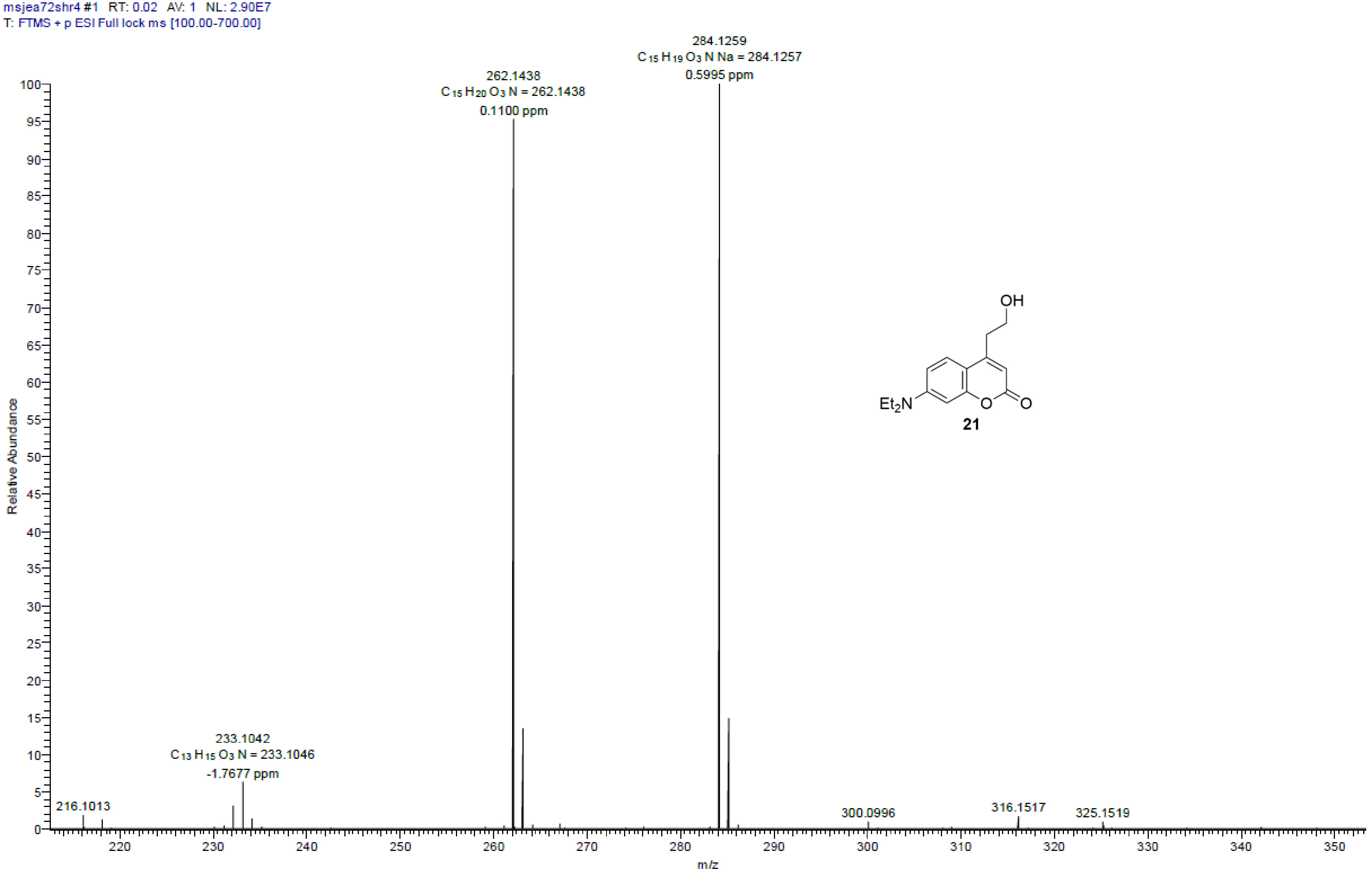

21

D:\data_2022\msjea73shr1 3/22/2022 2:53:23 PM ms119p2f1

msjea73shr1 #1 RT: 0.02 AV: 1 NL: 5.66E6
T: FTMS + p ESI Full lock ms [100.00-1200.00]

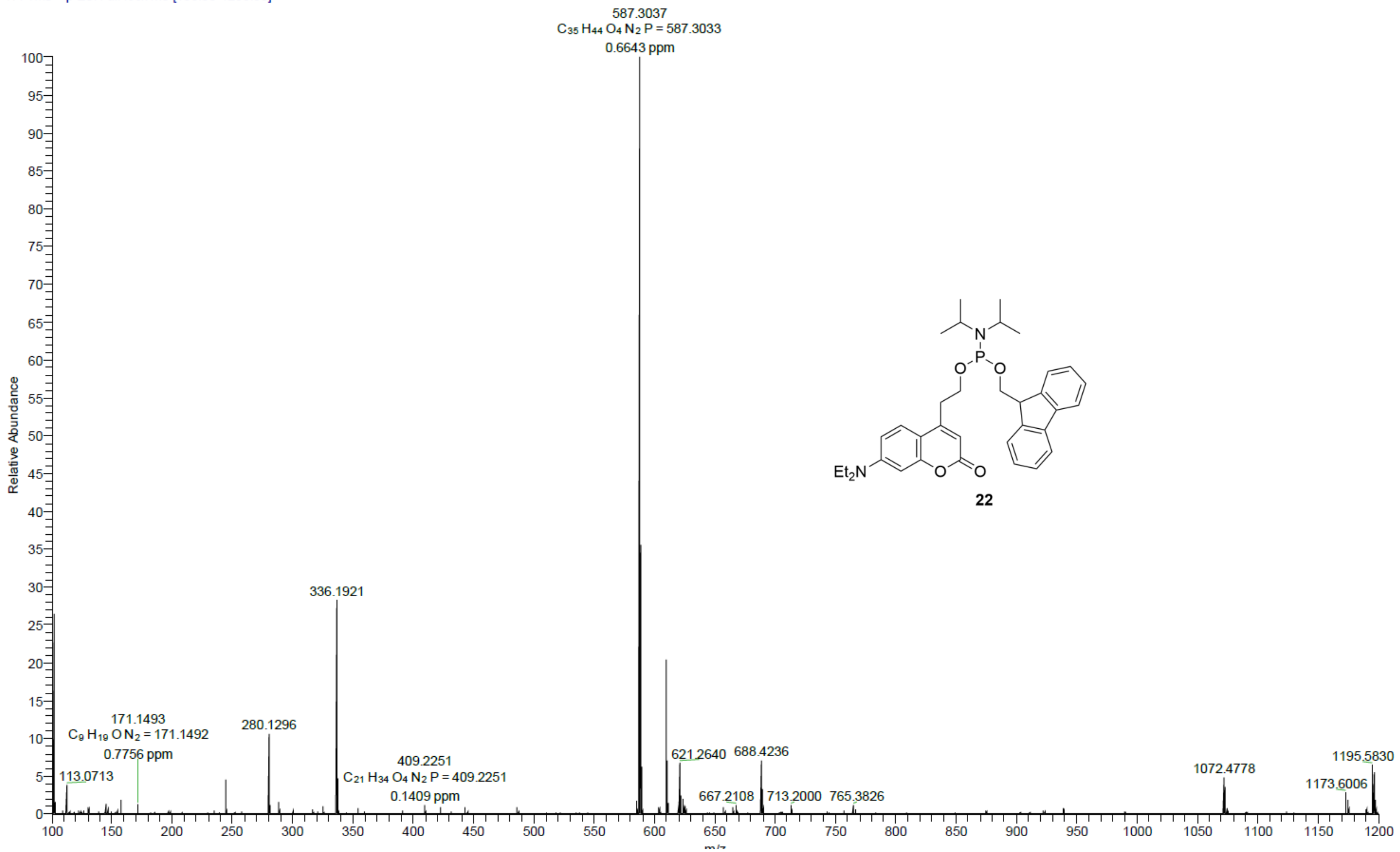

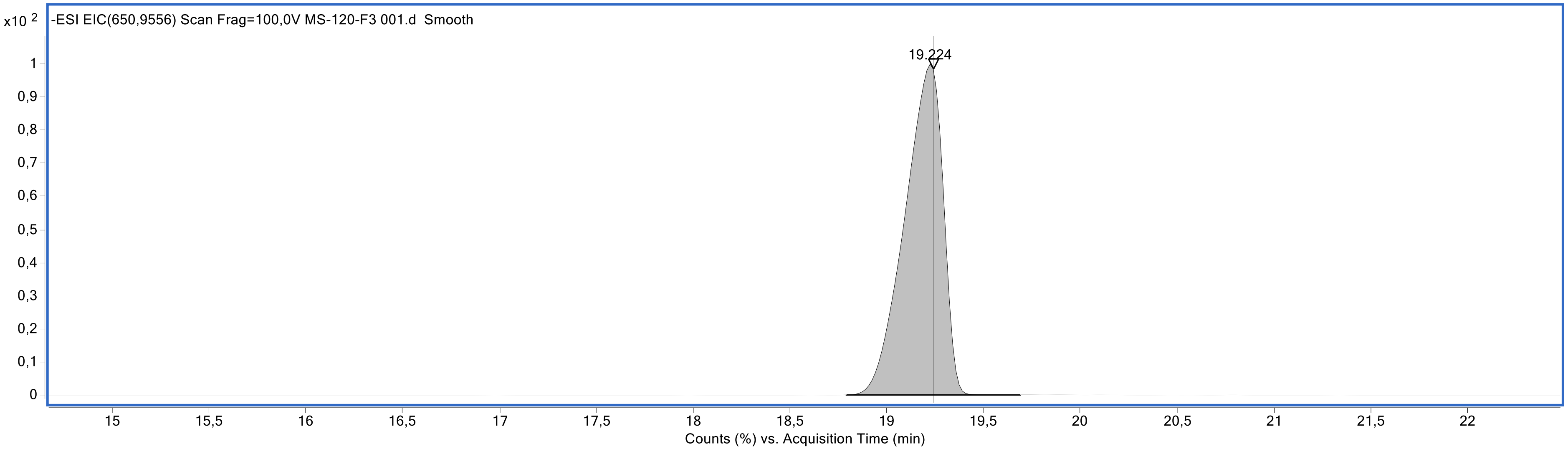

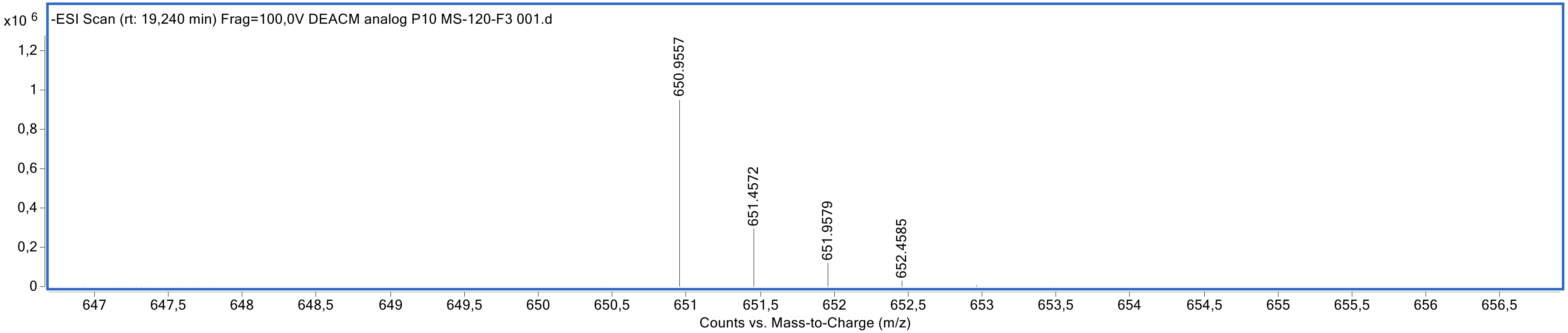

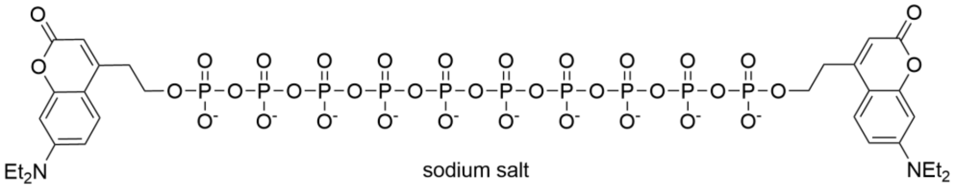

sodium salt

23

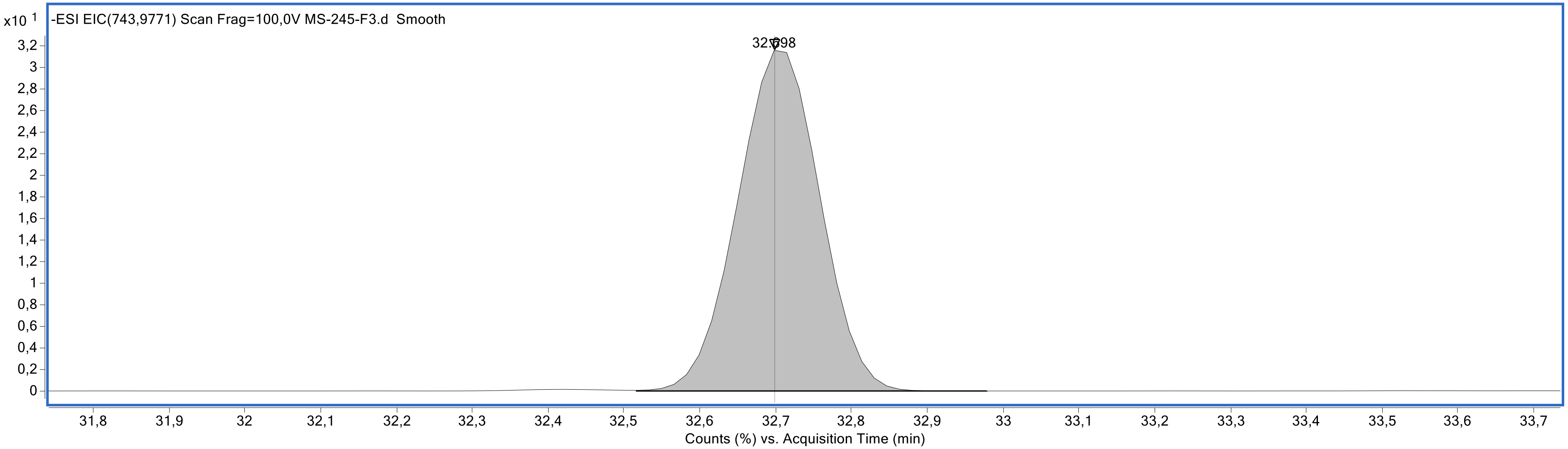
-ESI EIC(743,9771) Scan Frag=100,0V MS-245-F3.d Smooth
x10 1
32,698
Counts (%) vs. Acquisition Time (min)

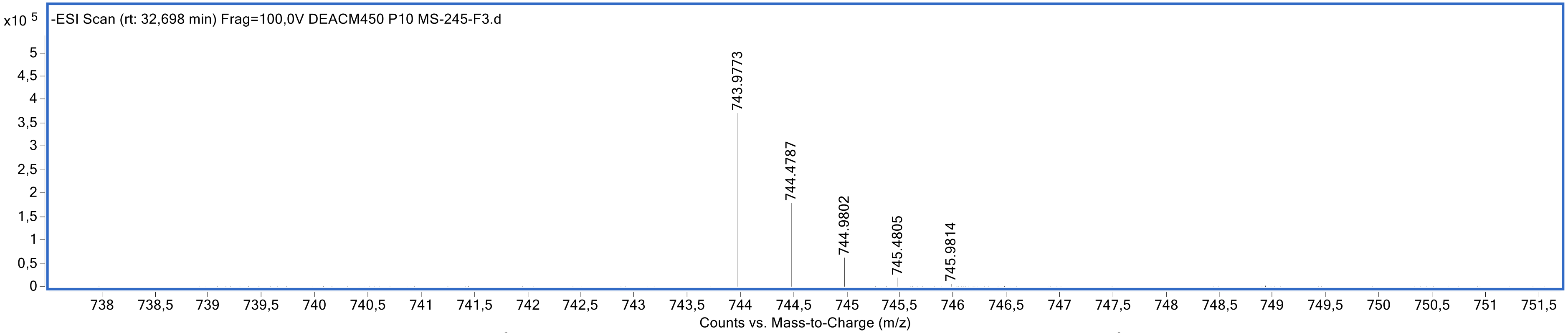
-ESI Scan (rt: 32,698 min) Frag=100,0V DEACM450 P10 MS-245-F3.d
x10 5
743.9773
744.4787
744.9802
745.4805
745.9814
Counts vs. Mass-to-Charge (m/z)

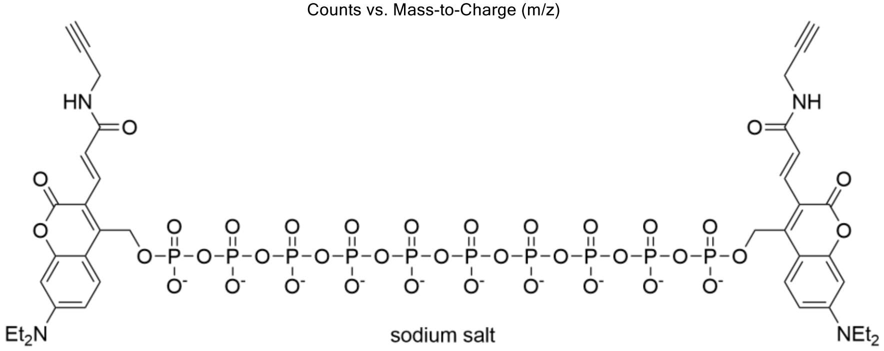
HN
NH
Et2N
NEt2
sodium salt

24

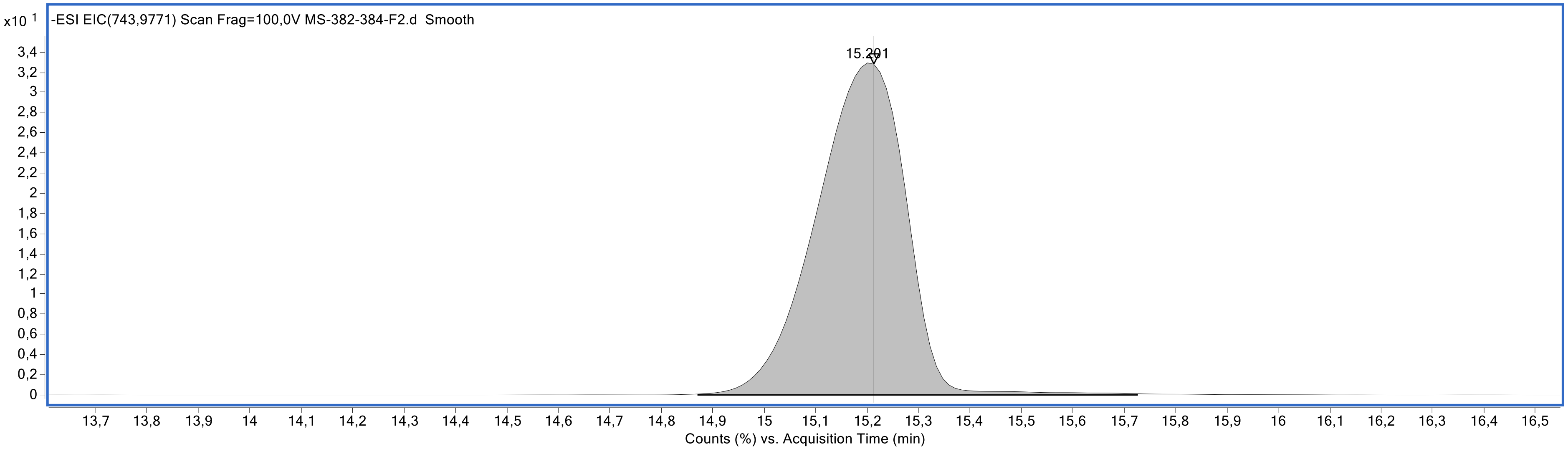
x10 1
-ESI EIC(743,9771) Scan Frag=100,0V MS-382-384-F2.d Smooth
15.201
3,4
3,2
3
2,8
2,6
2,4
2,2
2
1,8
1,6
1,4
1,2
1
0,8
0,6
0,4
0,2
0
13,7 13,8 13,9 14 14,1 14,2 14,3 14,4 14,5 14,6 14,7 14,8 14,9 15 15,1 15,2 15,3 15,4 15,5 15,6 15,7 15,8 15,9 16 16,1 16,2 16,3 16,4 16,5
Counts (%) vs. Acquisition Time (min)

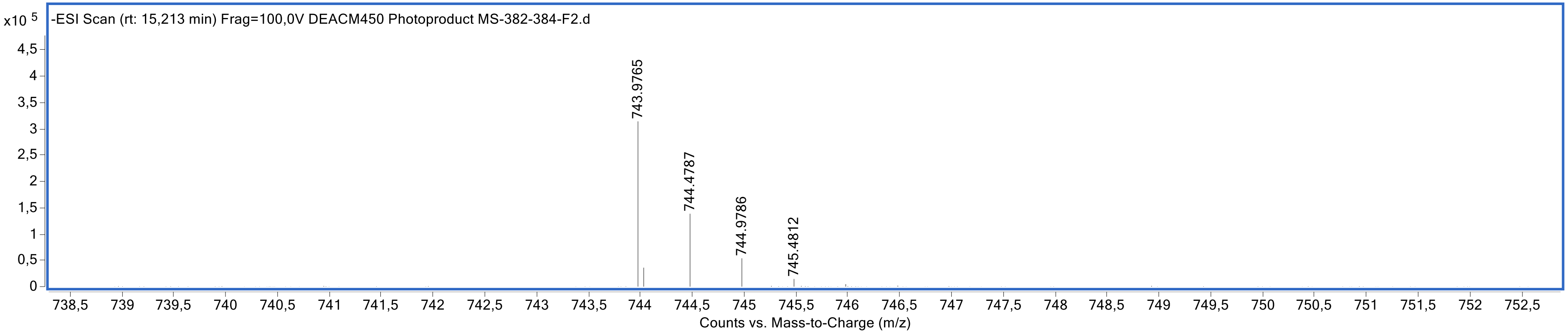
x10 5
-ESI Scan (rt: 15,213 min) Frag=100,0V DEACM450 Photoproduct MS-382-384-F2.d
743.9765
744.4787
744.9786
745.4812
4,5
4
3,5
3
2,5
2
1,5
1
0,5
0
738,5 739 739,5 740 740,5 741 741,5 742 742,5 743 743,5 744 744,5 745 745,5 746 746,5 747 747,5 748 748,5 749 749,5 750 750,5 751 751,5 752 752,5
Counts vs. Mass-to-Charge (m/z)

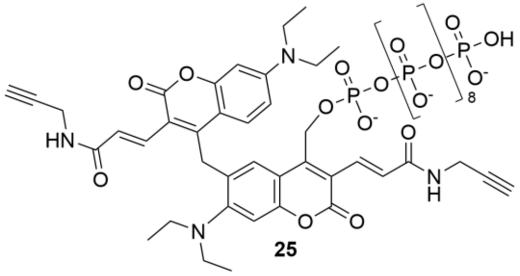
HN
O
N
H
OH
8

25

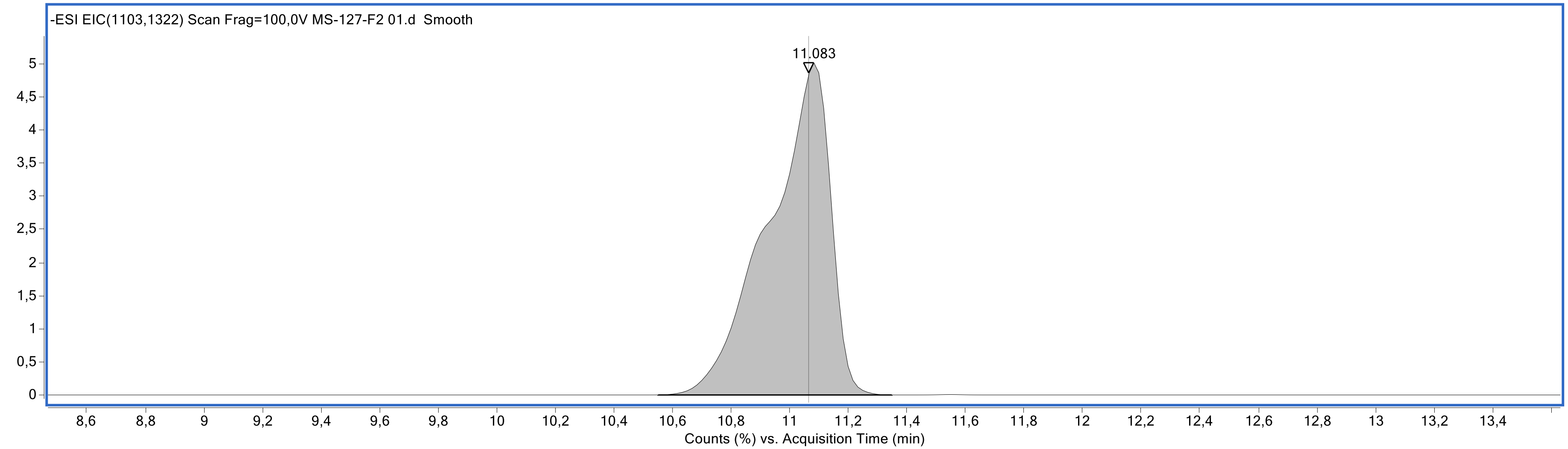

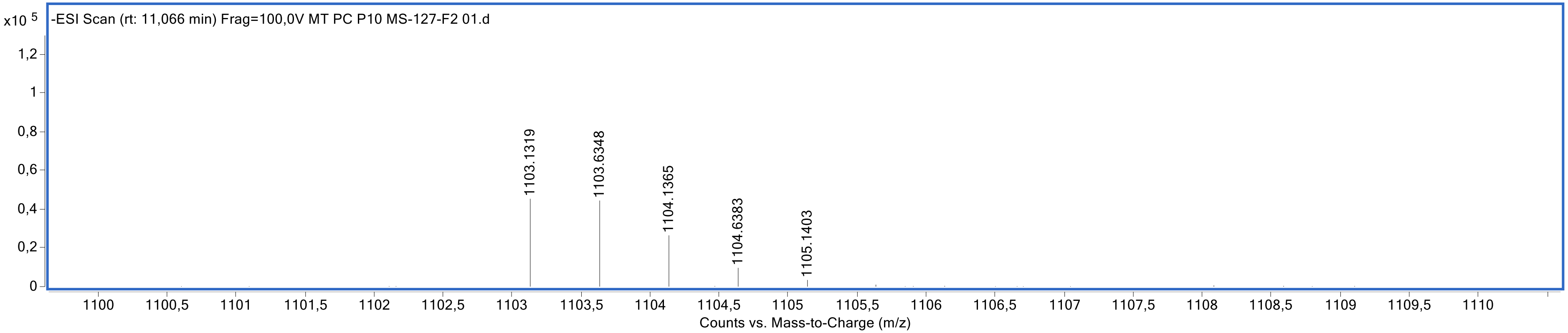

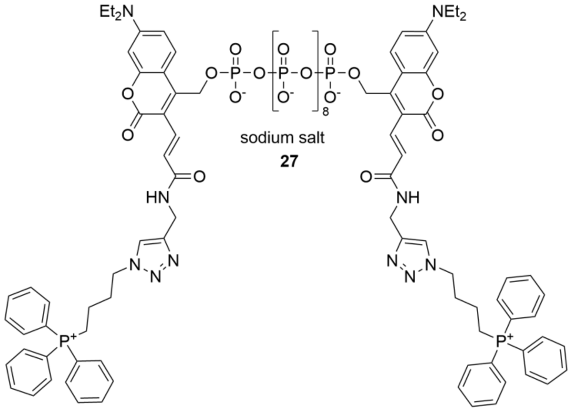

sodium salt

27
